# Hypophosphatemia as a Potential Class Effect of Histone Deacetylase Inhibitors: Evidence from Disproportionality Analysis and Mendelian Randomization Analysis of Drug Targets

**DOI:** 10.3390/ph19050689

**Published:** 2026-04-28

**Authors:** Ruiqi Zhao, Bei Zhang, Mengyao Han, Minling Lv, Jialing Sun, Xiaozhou Zhou

**Affiliations:** 1 The Fourth Clinical Medical College, Guangzhou University of Chinese Medicine, Shenzhen 518033, China; 20252120304@stu.gzucm.edu.cn (R.Z.); 20231121164@stu.gzucm.edu.cn (B.Z.); 2 The Second Clinical Medical College, Guangzhou University of Chinese Medicine, Guangzhou 510000, China; 20252110194@stu.gzucm.edu.cn; 3 Faculty of Chinese Medicine, Macau University of Science and Technology, Taipa, Macao 999078, China; luxlyulyu@163.com

**Keywords:** ADR, disproportionality analysis, FAERS, HDACi, hypophosphatemia, Mendelian randomization

## Abstract

**Background/Objective**: Histone deacetylase inhibitors (HDACi) represent a novel class of antineoplastic agents, yet their comprehensive safety profile warrants further investigation. This study aimed to examine the safety of HDACi using the FDA Adverse Event Reporting System (FAERS) and to explore causal relationships through Mendelian randomization (MR) analysis of drug targets. **Methods**: Adverse drug event (ADE) reports for Vorinostat, Romidepsin, Belinostat, and Panobinostat submitted to the FAERS from their respective market entry dates through 31 December 2023, were analyzed using disproportionality analyses with four algorithms, supplemented by time-to-onset analysis, logistic regression, and MR analysis. **Results**: A total of 1360, 1065, 225, and 1234 ADE reports were documented for Vorinostat, Romidepsin, Belinostat, and Panobinostat, respectively. Eight preferred terms, including decreased white blood cell, platelet, and neutrophil counts, hypophosphatemia, hypocalcemia, QT prolongation, increased aspartate aminotransferase, and anemia, exhibited positive signals across all four HDACi. A temporal decline in the risk of most HDACi-related ADEs was observed, and age, gender, and weight were identified as potential confounding factors for important medical events. Notably, MR analysis revealed a positive correlation between HDAC5 expression and serum phosphate levels. **Conclusions**: This pharmacovigilance study provides hypothesis-generating evidence that hypophosphatemia may represent a potential class effect of HDACi.

## 1. Introduction

Nucleosomes, the fundamental structural units of chromatin, are composed of histones and DNA. The intricate architecture between these components is maintained through ionic interactions between the positively charged histones and the negatively charged DNA backbone. However, excessively compact binding can impede DNA transcription efficiency [1]. Histone post-translational modifications represent a crucial epigenetic mechanism [2]. Since the discovery of histone acetylation in the 1960s, numerous studies have substantiated its pivotal role in chromatin remodeling [3]. Histone acetyltransferases (HATs) and histone deacetylases (HDACs) are enzymes that perform contrasting biological roles. HATs neutralize the positive charges on histone lysine residues through acetylation, resulting in a relaxed nucleosome structure that enhances gene expression. Conversely, HDACs restore the positive charges on histone lysine residues via deacetylation, leading to a more compact nucleosome structure that represses gene expression [4]. The dynamic equilibrium between HATs and HDACs plays an indispensable role in maintaining chromatin structural stability. Consequently, any disruption in the activity balance between these enzymes can lead to unstable chromatin structures, resulting in aberrant gene expression and ultimately manifesting as epigenetic disorders [5,6].

The activity equilibrium between HATs and HDACs plays a pivotal role in tumor initiation and progression. Numerous malignancies exhibit HDAC overexpression, resulting in elevated histone deacetylation levels [7,8,9]. Furthermore, studies have demonstrated that genetic deletion of HDACs in dendritic cells can influence their developmental trajectory, thereby enhancing tumor surveillance and facilitating a functional shift towards anti-tumor immunity [10]. Moreover, a complex interplay exists between HDACs and the tumor microenvironment, encompassing immune cell composition, cytokine signaling, and immune checkpoint expression, ultimately culminating in immune evasion [11].

Epigenetic immunotherapy, as an emerging approach in cancer treatment, has garnered increasing attention from oncologists and molecular biologists [12,13]. Numerous studies have demonstrated the efficacy of histone deacetylase inhibitors (HDACi) across various malignancies [14,15,16,17]. To date, 18 HDAC subtypes have been identified in humans, categorized into Class I, Class IIa, Class IIb, Class IV, and Sirtuins III [18]. Correspondingly, over 20 HDACi have been developed, with the majority being pan-HDACi. However, the non-selective nature of pan-HDACi necessitates careful consideration of their potential adverse effects on normal cellular physiology, which may lead to off-target effects and severe toxicities [19]. While reports of HDACi-related adverse events exist [11], a more comprehensive data-driven systematic analysis and mechanistic investigation are warranted. Our research aims to conduct a pharmacoepidemiological study utilizing the FDA Adverse Event Reporting System (FAERS) and validate causal relationships through drug target Mendelian randomization (MR). This methodology will yield significant insights for the clinical implementation of HDACi as well as for future experimental research.

## 2. Results

### 2.1. Descriptive Characteristics

Table 1 presents detailed patient information for adverse drug reaction reports associated with the four HDACi. Vorinostat-related adverse reactions were most frequently reported (1360 patients), followed by Panobinostat (1234), Romidepsin (1065), and Belinostat (225).

**Figure 1:**
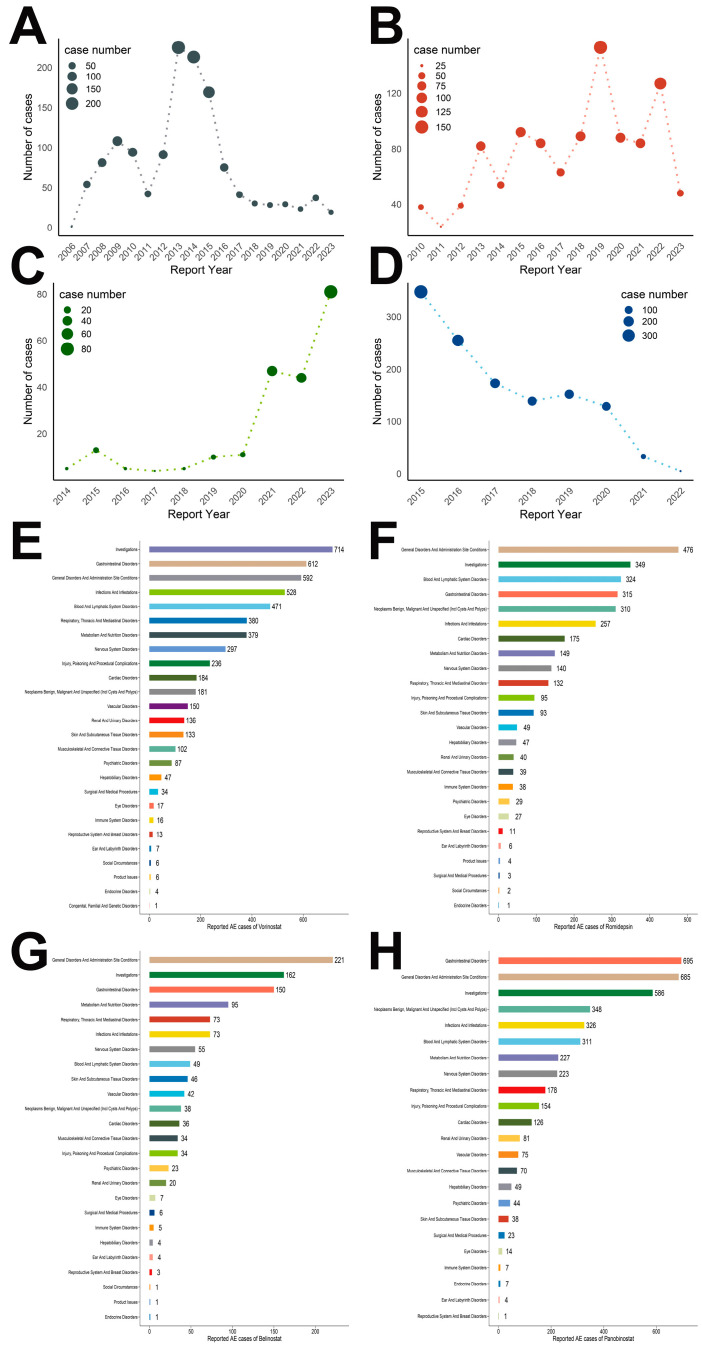
Line charts of the number of ADRs reported for (**A**) vorinostat; (**B**) romidepsin; (**C**) belinostat; and (**D**) panobinostat. Bar charts of the number of ADRs reported for (**E**) vorinostat; (**F**) romidepsin; (**G**) belinostat; and (**H**) panobinostat at the SOC level.

Among reports with specified gender, male patients consistently outnumbered females. The mean age for all groups exceeded 50 years (Vorinostat: 56; Romidepsin: 63; Belinostat: 60; Panobinostat: 64), with average body weights approximating 70 kg (Vorinostat: 71; Romidepsin: 71; Belinostat: 80; Panobinostat: 72). It is important to highlight that most cases originated from the United States and were reported by healthcare professionals, including physicians, pharmacists, and other practitioners. This emphasizes the credibility of our data sources. It is noteworthy that death and hospitalization constituted a significant proportion of patient outcomes. Trend analysis of HDACi-related ADR reports (Figure 1A–D) revealed distinct patterns: Vorinostat-related ADRs showed a declining trend in recent years, Romidepsin exhibited fluctuations, Belinostat demonstrated a consistent increase, while Panobinostat maintained a downward trajectory.

### 2.2. Signal Detects at the SOC Level

The comprehensive quantification and signal detection values for all HDACi-associated ADRs across SOC levels are meticulously documented in Table S3. To enhance visual comprehension, we have also generated histograms illustrating the frequency of ADR reports for each SOC (Figure 1E–H). Notably, General Disorders and Administration Site Conditions, Investigations, and Gastrointestinal Disorders consistently exhibited high reporting frequencies across all four HDACi agents. Vorinostat-related ADRs were most prominently reported in the Investigations category, comprising 714 cases (Figure 1E). For Romidepsin, General Disorders and Administration Site Conditions predominated with 476 reports (Figure 1F). Similarly, Belinostat showed a preponderance of General Disorders and Administration Site Conditions, accounting for 221 reports (Figure 1G). Panobinostat-associated ADRs were primarily manifested in Gastrointestinal Disorders, with 695 reported cases (Figure 1H).

**Figure 2:**
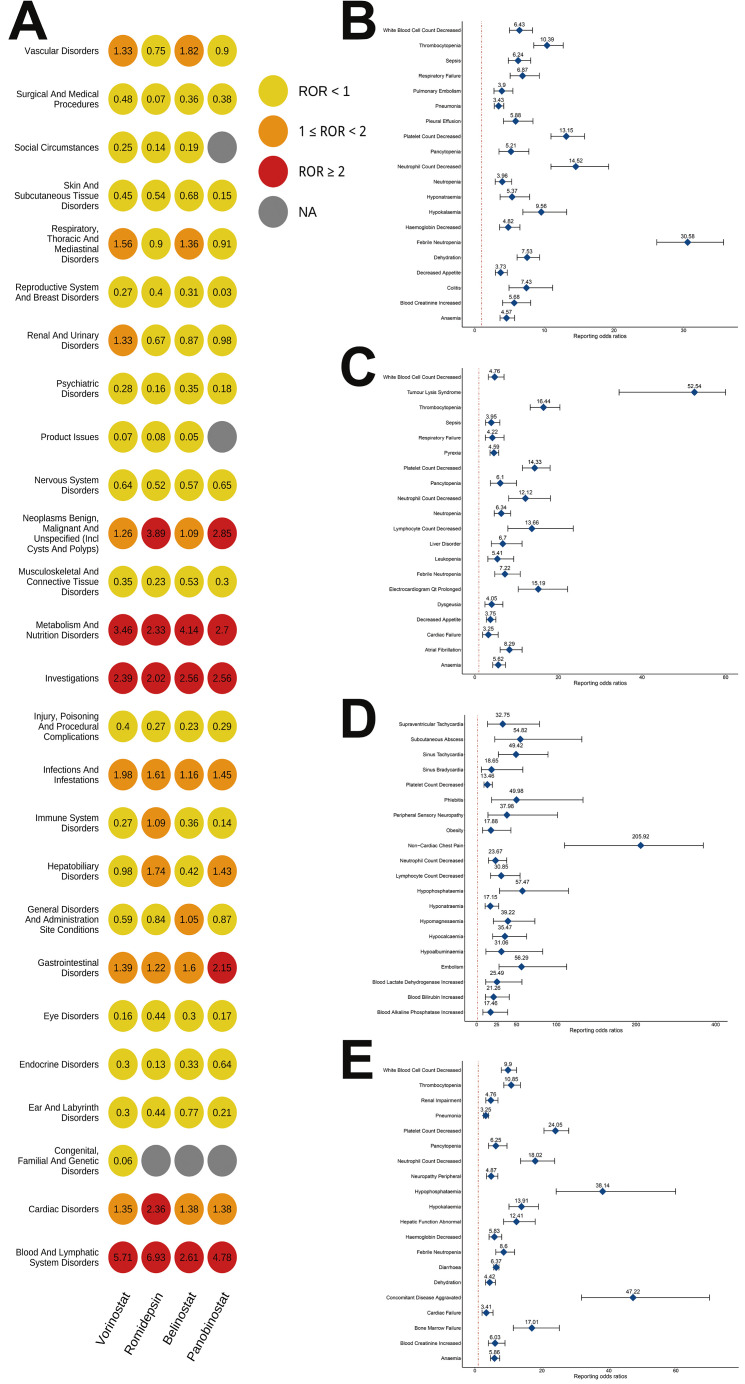
(**A**) Heat map showing ADRs signal detection at the SOC level for four HDACi. Forest plots depicting ADRs signal detection at the PT level for (**B**) vorinostat; (**C**) romidepsin; (**D**) belinostat; and (**E**) panobinostat.

Figure 2A elucidates the ROR algorithm results across 26 SOCs. Of particular significance, Metabolism and Nutrition Disorders, Investigations, and Blood and Lymphatic System Disorders consistently demonstrated ROR values ≥ 2 for all four HDACi agents. Blood and Lymphatic System Disorders exhibited notably elevated ADR signals across all four HDACis, with ROR values and 95% CIs as follows: Vorinostat 5.71 (5.19–6.28), Romidepsin 6.93 (6.17–7.77), Belinostat 2.61 (1.96–3.48), and Panobinostat 4.78 (4.26–5.36).

### 2.3. Signal Detects at the PT Level

**Figure 3:**
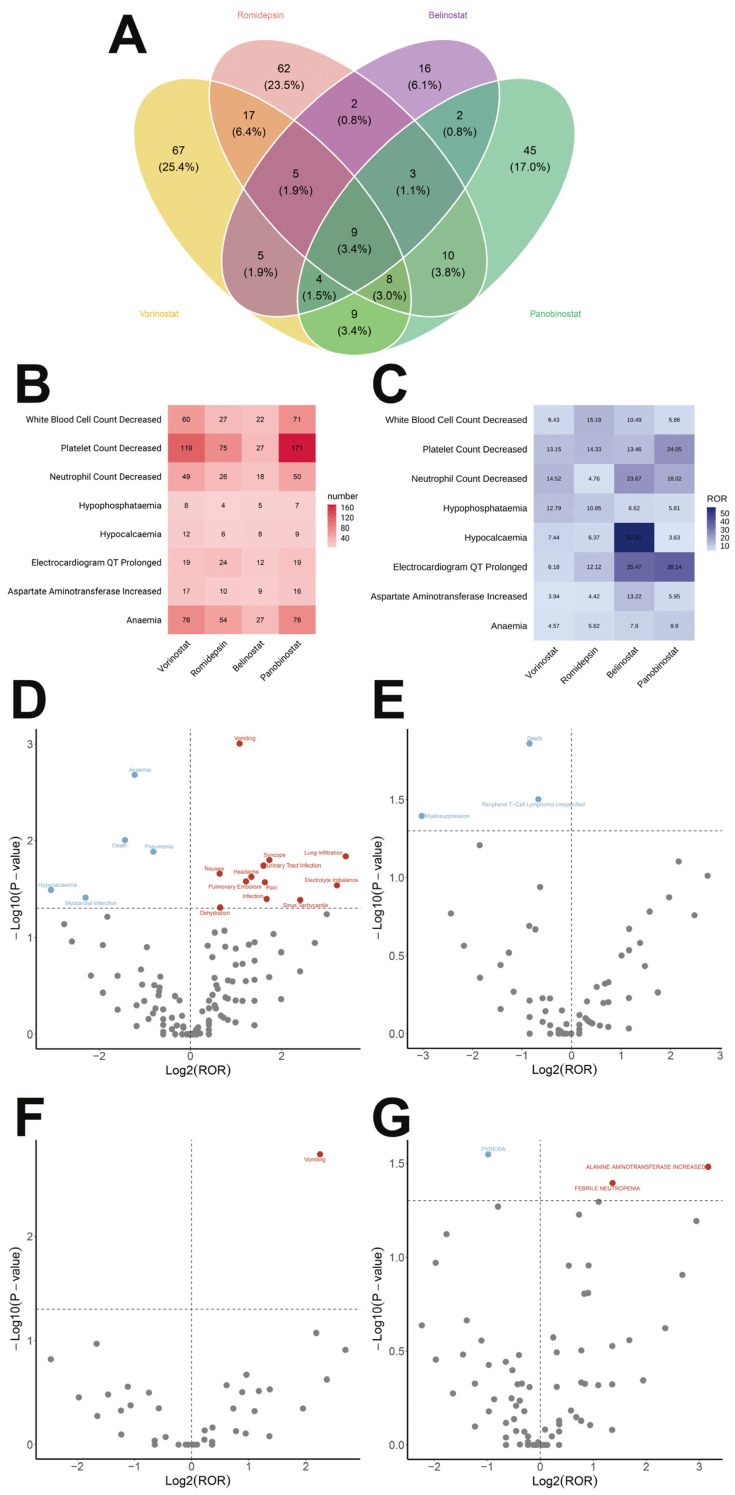
(**A**) Venn diagram showing the significant PTs common to four HDACi. (**B**) Heat map depicting the number of reported significant PTs shared by the four HDACi. (**C**) Heat map illustrating the signal intensity of significant PTs common to all four HDACi. Volcano plots depicting (**D**) vorinostat; (**E**) romidepsin; (**F**) belinostat; and (**G**) panobinostat, showing gender-differentiated risk signals.

Table S4 provides a detailed record of the number and signal detection values of ADRs associated with HDACi at the PT level. We have comprehensively considered the number of reports and the results of signal detection, and plotted the ROR algorithm results of the top 20 PTs in a forest plot. It can be seen that the signal for Febrile Neutropenia associated with Vorinostat (Figure 2B) is the strongest, with an ROR of 30.58 and a 95% CI of (26.15–35.74); the signal for Tumor Lysis Syndrome associated with Romidepsin (Figure 2C) is the strongest, with an ROR of 52.54 and a 95% CI of (34.52–79.97); the signal for Non-Cardiac Chest Pain associated with Belinostat (Figure 2D) is the strongest, with an ROR of 205.92 and a 95% CI of (110.3–384.46); and the signal for Concomitant Disease Aggravated associated with Panobinostat (Figure 2E) is the strongest, with an ROR of 47.22 and a 95% CI of (31.83–70.03). Subsequently, we used a Venn diagram to show the PTs that were significantly associated with all four HDACi (Figure 3A), and obtained 9 PTs that were common to the four HDACi. After excluding Disease Progression, we plotted heatmaps of the number of reports (Figure 3B) and the signal strength (Figure 3C) for the remaining 8 PTs. It can be clearly seen that the number of reports of Platelet Count Decreased was relatively high for all four HDACi, with Panobinostat having the most reports (171 cases); and the signal strength for Hypocalcemia associated with Belinostat was the strongest (ROR = 57.47).

### 2.4. Gender Difference Analysis

**Figure 4:**
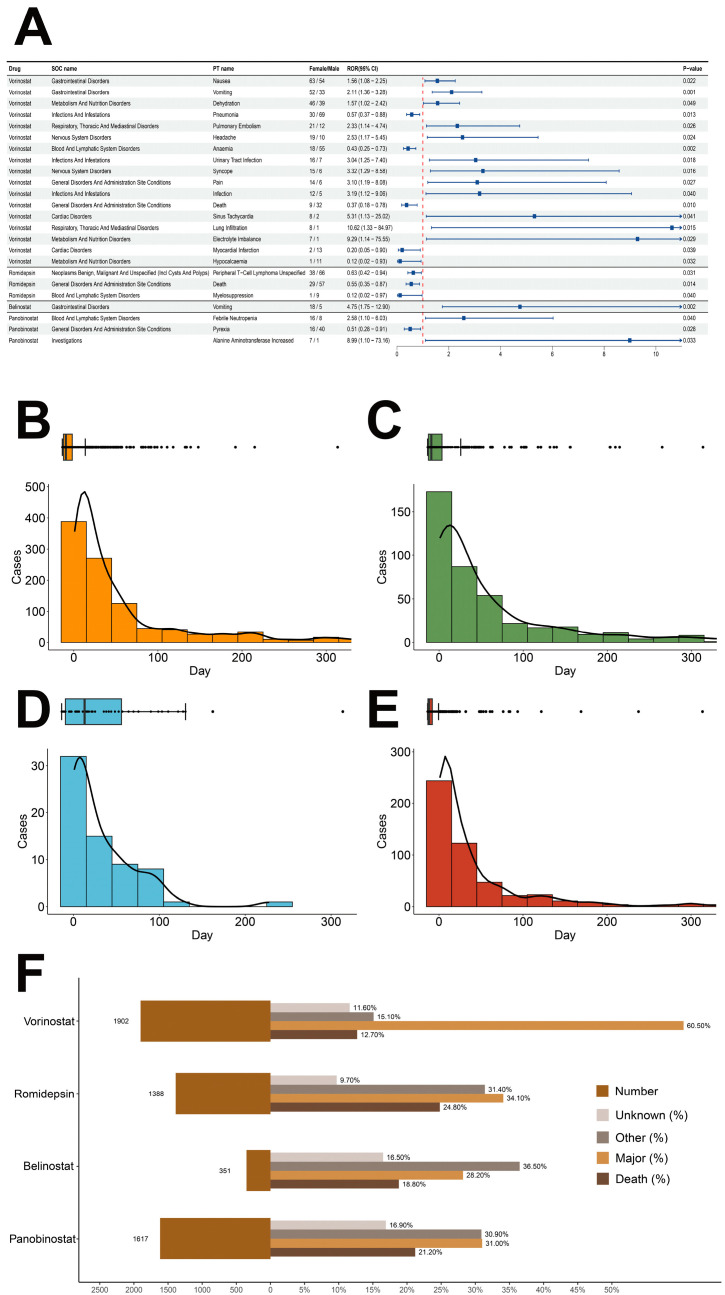
(**A**) Forest plot depicting gender-differentiated risk signals associated with HDACi. Box plot depicting distribution of the time of occurrence of ADRs associated with (**B**) vorinostat; (**C**) romidepsin; (**D**) belinostat; and (**E**) panobinostat. (**F**) Histogram of outcome events related to HDACi.

We also conducted a gender-stratified analysis and generated volcano plots (Figure 3D,E), in which each dot represents a PT, with high-risk PTs for females shown in red and high-risk PTs for males shown in blue. We then plotted a forest plot (Figure 4A) for the PTs with significant differences. The results indicate that, compared to females, males were more prone to the following PTs associated with Vorinostat administration: Pneumonia (ROR, 95% CI, *p*-value: 0.57, 0.37–0.88, 0.013), Anemia (0.43, 0.25–0.73, 0.002), Death (0.37, 0.18–0.78, 0.010), Myocardial Infarction (0.20, 0.05–0.90, 0.039), and Hypocalcemia (0.12, 0.02–0.93, 0.032); the PTs associated with Romidepsin administration were Peripheral T-Cell Lymphoma Unspecified (0.63, 0.42–0.94, 0.031), Death (0.55, 0.35–0.87, 0.014), and Myelosuppression (0.12, 0.02–0.97, 0.040); and the PT associated with Panobinostat administration was Pyrexia (0.51, 0.28–0.91, 0.028).

### 2.5. Time to Onset (TTO) Analysis

To investigate the timing of HDACi-related ADRs, we generated histograms, density plots, and box plots that represent the TTO of these events. (Figure 4B–E). The data indicate that the incidence of ADRs related to the four HDACi declined over time, supporting the hypothesis that the risk of HDACi-related ADRs decreases as time progresses. To verify this hypothesis, we conducted Weibull distribution tests, with the detailed findings presented in Table 2. The study findings confirmed our hypothesis, with the median time to occurrence of ADRs being 28 days for Vorinostat, 29 days for Romidepsin, 19.5 days for Belinostat, and 18 days for Panobinostat. Moreover, the α values for all four HDACi were below 1, and the 95% CIs were similarly less than 1. This suggests an early failure-type curve, indicating that the risk of associated ADRs diminishes over time.

### 2.6. Logistic Regression for the Occurrence of Important Medical Events (IMEs)

As shown in Figure 4F, the proportions of fatal and life-threatening outcomes for Vorinostat, Romidepsin, Belinostat, and Panobinostat were 73.2%, 58.9%, 47.0%, and 52.2%, respectively, suggesting the potential for serious ADRs associated with HDACi. Consequently, we developed both univariate and multivariate logistic regression models to examine the potential relationships among gender, age, and weight in relation to the occurrence of HDACi-related important medical events (IMEs), with the detailed parameters of the models provided in Table 3. The findings reveal that gender (OR = 1.49, 95% CI: 1.15–1.95, *p* = 0.003) and age (treated as a continuous variable) (OR = 1.01, 95% CI: 1.01–1.02, *p* < 0.001) were significantly linked to the occurrence of Vorinostat-related IMEs. In contrast, age (as a continuous variable) (OR = 0.97, 95% CI: 0.95–0.99, *p* = 0.004) demonstrated a negative association with the incidence of Romidepsin-related IMEs. Additionally, gender (OR = 0.24, 95% CI: 0.08–0.67, *p* = 0.009) and weight (considered as a categorical variable) (OR = 0.30, 95% CI: 0.11–0.80, *p* = 0.018) were significantly negatively associated with the occurrence of Belinostat-related IMEs.

### 2.7. Drug Target Mendelian Randomization (MR) Analysis

**Figure 5:**
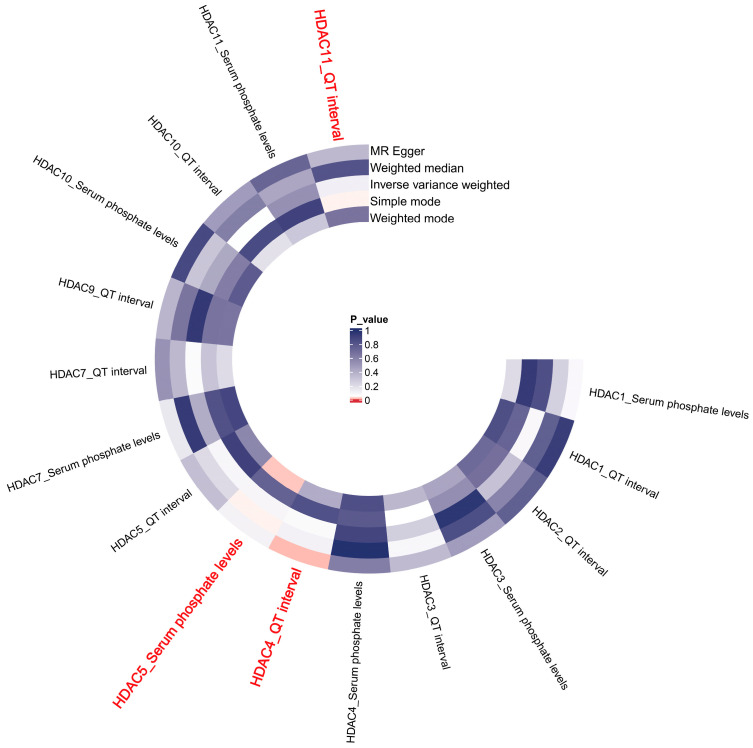
Heat map of Mendelian randomization analysis results.

Figure 5 presents the results of the MR analysis on the drug target genes. The study found a positive correlation between the expression of HDAC4 and QT interval (MR Egger: Beta = 0.273, *p* = 0.017), suggesting that HDACi may shorten the QT interval. Interestingly, our research also provided evidence of a negative correlation between HDAC11 and QT interval (Simple mode: Beta = −0.571, *p* = 0.048), indicating that the QT interval may be regulated by multiple HDAC isoforms. In this study, HDAC4 and HDAC11 had opposing effects on the QT interval. Moreover, the expression of HDAC5 exhibited a significant positive correlation with serum phosphate levels, as determined by both the weighted median and weighted mode methods (Weighted median: Beta = 0.018, *p* = 0.044; Weighted mode: Beta = 0.018, *p* = 0.023). This finding suggests that the administration of HDACi may reduce serum phosphate levels, potentially resulting in hypophosphatemia.

## 3. Discussion

HDACi are an emerging class of antineoplastic agents, often utilized in the treatment of hematological malignancies. They have received FDA approval for the treatment of CTCL, PTCL, and MM, while their application in solid tumors is currently under exploration [5,18]. Although reports of HDACi-related ADRs have been made, post-marketing ADR signal detection holds great value in identifying and characterizing ADRs that may not have occurred during clinical trials, thereby contributing to the assessment of post-marketing drug safety [20,21]. The results of signal detection provide important reference for clinical practitioners in their medication use and serve as a basis for further mechanistic investigations. Therefore, our study employed pharmacoepidemiological analysis of the FAERS database to examine HDACi-related ADRs, and leveraged MR analysis of GWAS data to provide evidence for the causal relationships between HDACi and the associated ADRs.

Significant discrepancies were observed in the number of adverse event reports among the four HDACi, which likely reflect differences in market availability and prescribing patterns rather than solely toxicity profiles. Vorinostat (approved in 2006) has a significantly longer market presence compared to Belinostat (approved in 2014) and Panobinostat (approved in 2015), contributing to its higher cumulative report volume. Furthermore, the indicated patient populations differ; Panobinostat is indicated for Multiple Myeloma, a condition with a higher prevalence than the rare T-cell lymphomas treated by Romidepsin and Belinostat, which partly explains the high report volume for Panobinostat despite its later approval. The declining trend in Vorinostat-related ADRs may reflect clinical familiarity and improved management over time, whereas the increasing trend for Belinostat aligns with its growing adoption following its more recent market entry.

Our study reveals that HDACi-related ADRs tend to occur more frequently during the early stages of administration, with the risk of such events diminishing over time. The potential for serious outcomes underscores the importance of close clinical observation and frequent monitoring of relevant parameters for oncologists in the safe clinical use of HDACi. However, “death” was frequently reported across all drugs. It is crucial to interpret this with caution; given that these agents are approved for relapsed or refractory malignancies, the high incidence of death reports is likely confounded by the severity of the underlying disease and disease progression, rather than being solely attributable to drug toxicity. This phenomenon is a known limitation in pharmacovigilance of oncological therapies. Notably, ADRs classified under the SOCs of General Disorders and Administration Site Conditions, Investigations, and Gastrointestinal Disorders were prevalent across the four HDACi. The former included events such as Asthenia, Fatigue, and Edema; the Investigations category encompassed common biochemical tests like Platelet Count Decreased, White Blood Cell Count Decreased, Neutrophil Count Decreased, Hemoglobin Decreased, and electrocardiographic changes; while the Gastrointestinal Disorders comprised Nausea, Vomiting, and Diarrhea. These findings align with the “most common adverse reactions (incidence >20%)” reported in the FDA drug labels (https://www.accessdata.fda.gov/scripts/cder/daf/index.cfm, accessed on 10 December 2024), corroborating the reliability of our study. Furthermore, our research has identified certain PTs that may exhibit gender-specific predispositions, providing valuable insights for future clinical individualized treatment and adverse event monitoring. Additionally, our study has confirmed the significant associations between the use of HDACi and the prolongation of QT interval, as well as the development of hypophosphatemia. These findings were consistently observed across the disproportionality analyses of the four HDACi and were further supported by the MR analysis of the drug target genes.

The isoform-specific association between HDAC5 expression and serum phosphate levels observed in our Mendelian randomization (MR) analysis should be interpreted with caution, as direct experimental evidence linking HDAC5 to phosphate homeostasis remains limited in the current literature. This finding may represent a novel observation that warrants further prospective validation. The isoform-specific relationship between HDAC5 expression and serum phosphate levels also calls for detailed mechanistic investigation. Among the class IIa HDAC family members (HDAC4, HDAC5, HDAC7, and HDAC9), HDAC5 possesses distinct regulatory functions that may underlie its particular relevance to phosphate homeostasis. HDAC5 plays a critical role in transcriptional regulation and has been closely implicated in the pathogenesis of multiple cancer types [22]. Moreover, previous studies have suggested that microRNA miR-134-5p may enhance inorganic phosphate-induced calcium deposition in vascular smooth muscle cells by suppressing HDAC5 expression [23]. This finding indirectly supports a potential link between HDAC5 and serum phosphate levels.

In drug labels, severe electrocardiographic changes and electrolyte abnormalities are included in the black box warnings. Hypophosphatemia is one of the most common non-hematological laboratory abnormalities (incidence >20%). A large phase I study has shown that in patients with relapsed and refractory multiple myeloma receiving a treatment regimen that included Panobinostat, 80% experienced grade 3–4 ADRs, with hypophosphatemia accounting for 25% of these [24]. Similarly, a study on the treatment of relapsed pediatric acute lymphoblastic leukemia with Decitabine and Vorinostat revealed that hypophosphatemia (43%) was the most common grade 3–4 ADR [25]. The use of Romidepsin has also been associated with grade 3–4 asymptomatic hypophosphatemia [26]. Notably, the research by Lia Gore [27] et al. has indicated that hypophosphatemia is a dose-limiting toxicity in HDACi treatment regimens. A key point is that our study found positive signals for hypophosphatemia across all four HDACi, and this ADR is mentioned in the drug labels. In clinical trial settings, hypophosphatemia has frequently occurred as a dose-limiting toxicity, suggesting that it may be a potential class effect of HDACi. While the underlying mechanisms remain incompletely understood, our MR analysis of the drug targets has offered evidence supporting a causal relationship between HDACi and hypophosphatemia. In summary, for patients receiving HDACi, urine and serum electrolyte analyses should be performed, and oral phosphate supplementation may be necessary, with regular monitoring to maintain serum phosphate levels within the normal range.

The apprehension regarding the potential of HDACi to prolong the ventricular repolarization, or QT interval, has been a subject of considerable debate. With the exception of Belinostat, the prescribing information for the remaining three HDACi carries a warning concerning QT interval prolongation. For patients, a prolonged QT interval on an electrocardiogram may portend severe arrhythmias, such as torsades de pointes, which can be life-threatening. However, whether the concern over QT prolongation induced by HDACi can be definitively concluded remains unclear. Mutations in the hERG gene are known to cause long QT syndrome [28], rendering it an anti-target in drug development. Cellular studies have shown that inhibition of HDAC6 induces acetylation of hERG, counteracting its ubiquitination and thereby stabilizing it, suggesting a potential novel therapeutic approach for long QT syndrome [29]. Conversely, HDACi may impact hERG maturation and consequently prolong the QT interval through transcriptional mechanisms involving altered gene expression required for ion channel trafficking and localization to the sarcolemma [30]. While early clinical studies observed QT prolongation in patients treated with HDACi [31,32], with reports of torsades de pointes [33], recent investigations have refuted this notion [34,35], attributing the QT prolongation to concomitant use of antiemetics, electrolyte imbalances, and potential false positives due to increased heart rate [36,37,38]. Intriguingly, our MR analysis identified HDAC4 and HDAC11 as potential antagonists of QT prolongation. Although a causal relationship cannot be definitively established, our findings suggest that the impact of HDACi on the QT interval may involve multiple pathways, targets, and potential mediators. Moreover, the apparently contradictory MR findings regarding QT interval—with HDAC4 showing positive correlation (suggesting potential QT shortening upon inhibition) and HDAC11 showing negative correlation (suggesting potential QT prolongation upon inhibition)—require careful interpretation and should not be viewed as definitive evidence of causal effects. Several critical considerations temper these genetic associations. First, the MR analysis employed blood-derived eQTL data, which may not adequately capture HDAC expression and function in cardiac myocytes, where QT interval regulation physiologically occurs. Second, the effect sizes were modest and detected by only single MR methods rather than showing consistency across all five analytical approaches, suggesting potential methodological artifacts or weak instruments. In summary, while our genetic and pharmacovigilance data suggest potential associations between HDAC inhibition and cardiac repolarization, the contradictory isoform-specific effects, methodological limitations, and extensive confounding preclude definitive causal conclusions. The prudent clinical approach remains vigilant ECG monitoring with meticulous correction of electrolyte abnormalities, rather than categorical avoidance of HDACi based on QT concerns alone.

The time-to-onset analysis provides clear evidence for risk stratification and monitoring protocols in clinical practice. The consistent early failure pattern (Weibull α < 1) across all four HDACi, with median TTO ranging from 18 to 29 days, indicates that the critical window for intensive monitoring encompasses the first 2–4 weeks of therapy. Based on these findings, we recommend the following evidence-based monitoring protocol: (1) baseline comprehensive metabolic panel including serum phosphate, calcium, and magnesium prior to HDACi initiation; (2) weekly electrolyte monitoring during the first month of therapy; (3) biweekly monitoring during weeks 5–8; and (4) monthly monitoring thereafter in stable patients. For hypophosphatemia specifically, which demonstrated consistent signals across all four agents, prophylactic oral phosphate supplementation may be considered in high-risk patients (elderly, baseline low-normal phosphate, concurrent nephrotoxic agents). Similarly, baseline and weekly ECG monitoring during the first month is advisable given QT prolongation signals, particularly in patients with additional risk factors (hypokalemia, hypomagnesemia, concomitant QT-prolonging medications). The declining risk profile after the initial treatment period should not preclude ongoing vigilance, as our analysis also identified late-onset events, albeit at lower frequency.

In addition, the gender-stratified analyses should be interpreted with caution. Given the large number of PT-level comparisons performed, the absence of formal multiple-testing correction may increase the risk of type I error inflation. However, applying stringent correction methods such as Bonferroni or FDR adjustment in this exploratory pharmacovigilance context would markedly reduce statistical power and could obscure potentially meaningful weak-to-moderate safety signals. Therefore, these sex-specific findings should be considered hypothesis-generating rather than confirmatory. Future studies with larger sample sizes and prospective designs are warranted to validate and further characterize potential sex-related differences in HDACi-associated adverse drug reactions.

Despite the advantages conferred by the pharmacoepidemiological analysis based on the FAERS database and the drug target MR analysis leveraging large-scale GWAS studies, certain limitations persist as challenges to be addressed. Firstly, incomplete or selective reporting is an inherent limitation of the FAERS database, introducing an unavoidable degree of selection bias to our study [39,40]. Specifically, the FAERS database does not fully account for patients’ comprehensive medical history, such as comorbidities and the presence of concomitant medications, which could serve as significant confounding factors. Secondly, owing to the unavailability of the total number of patients receiving HDACi treatment, we could only quantify the occurrence of ADRs and perform disproportionality analysis, precluding the calculation of incidence rates for HDACi-associated ADRs [41]. Confounding by cancer severity and disease progression represents a fundamental challenge in interpreting our findings and warrants explicit discussion. Patients receiving HDACi typically have relapsed or refractory hematological malignancies—clinical contexts associated with extensive prior chemotherapy exposure, advanced disease burden, and compromised organ function. Many of the ADRs identified in our analysis, including cytopenias, fatigue, hypophosphatemia, electrolyte disturbances, and even death, are well-established manifestations of progressive malignancy itself, independent of treatment. The FAERS database does not capture disease severity metrics, precluding adjustment for this critical confounder. Another important source of confounding arises from concomitant medications. Patients treated with HDAC inhibitors frequently receive combination regimens or supportive therapies, including corticosteroids, immunomodulatory agents, proteasome inhibitors, antiemetics, antibiotics, bisphosphonates, and electrolyte supplements. Many of these agents are independently associated with hematologic toxicity, electrolyte disturbances, QT interval changes, or renal dysfunction. On the other hand, our methodological approach of requiring concordance across all four disproportionality algorithms (ROR, PRR, BCPNN, and MGPS) represents a conservative strategy that prioritizes specificity over sensitivity. While this stringent criterion substantially reduces false-positive signals and enhances the reliability of identified ADRs, we acknowledge an inherent trade-off: weaker but potentially clinically meaningful signals, particularly those associated with rare events or emerging toxicities, may be excluded from our primary analysis. Previous comparative studies have demonstrated that Bayesian algorithms (BCPNN and MGPS) exhibit superior performance for detecting signals among infrequently reported events, whereas frequentist methods (ROR and PRR) demonstrate greater sensitivity for early signal detection but with higher false-positive rates [42]. Furthermore, the paucity of detailed clinical data necessitated the omission of certain covariates [43], hindering our ability to control for confounding factors such as smoking and alcohol consumption. Lastly, while disproportionality analysis is limited in quantifying risk and establishing causality [44], a limitation partially mitigated by the drug target MR analysis, additional experimental research is warranted to elucidate the potential mechanisms that underlie the relationship between HDACi and their associated ADRs. In the FAERS context, collider bias occurs because only patients who experience ADRs sufficiently severe to trigger reporting enter the database, potentially creating spurious associations between drug exposure and specific ADRs if reporting propensity differs by patient characteristics or co-exposures. This bias may partially explain some observed associations and cannot be fully addressed through analytical adjustments. Second, the absence of denominator data fundamentally precludes calculation of true incidence rates, absolute risk measures, or number-needed-to-harm estimates. Our disproportionality metrics (ROR, PRR) quantify relative reporting patterns rather than actual occurrence rates, and high ROR values may reflect either genuinely elevated risk or differential reporting propensities. Additionally, the voluntary nature of FAERS reporting introduces unmeasured confounding by indication, disease severity, and healthcare utilization patterns that cannot be adequately controlled through conventional adjustment methods. To overcome these inherent limitations of spontaneous reporting systems, future research should prioritize prospective registry-based cohort studies that capture all exposed patients regardless of outcome status, such as linkage of electronic health records with pharmacy dispensing data.

## 4. Materials and Methods

### 4.1. FAERS Data Curation and Refinement

We carried out a retrospective pharmacoepidemiological study based on real-world data, utilizing the FAERS database, focusing on four FDA-approved HDACi: Vorinostat, which received approval in 2006 for cutaneous T-cell lymphoma (CTCL); Romidepsin, approved in 2009 for CTCL; Belinostat, granted approval in 2014 for peripheral T-cell lymphoma (PTCL); and Panobinostat, authorized in 2015 for multiple myeloma (MM). The drug names used for database queries are detailed in Table S1. Our analysis encompassed all data from the respective approval dates of these HDACi through 31 December 2023. Because FAERS is based on spontaneous reporting, the database may contain duplicate reports or cases that have been withdrawn or deleted. The FDA provides official guidance on data deduplication procedures as well as lists of reports that should be excluded. In this study, data cleaning was conducted in strict accordance with the FDA’s publicly available recommendations. Specifically, duplicate reports were removed following the FDA-recommended deduplication strategy. We extracted the variables PRIMARYID, CASEID, and FDA_DT from the DEMO table and sorted records by CASEID, FDA_DT, and PRIMARYID. For reports sharing the same CASEID, only the record with the most recent FDA_DT was retained. If both CASEID and FDA_DT were identical, the report with the largest PRIMARYID was preserved [45,46,47]. Ultimately, we identified adverse drug reactions (ADRs) with HDACi as the primary suspect (PS) as follows: Vorinostat (1360 reports), Romidepsin (1065 reports), Belinostat (225 reports), and Panobinostat (1234 reports). It is important to note that while reports where the HDACi was listed as the PS were selected to minimize confounding, these reports may still include cases where patients were using concomitant medications. The detailed screening process is illustrated in Figure S1 For standardized medical terminology, we adhered to the Medical Dictionary for Regulatory Activities (MedDRA) [48,49]. Our analysis employed two hierarchical levels of MedDRA (version 26.0): System Organ Class (SOC), the highest level of terminology, and Preferred Term (PT) [50], the most commonly used terminology.

### 4.2. Disproportionality Analysis

To identify and detect adverse drug reactions (ADRs) associated with the four HDACi, we employed disproportionality analysis [51]. This methodology includes both non-Bayesian algorithms, such as the reporting odds ratio (ROR) and the proportional reporting ratio (PRR), alongside Bayesian algorithms like the Bayesian Confidence Propagation Neural Network (BCPNN) and the multi-item gamma Poisson shrinker (MGPS). Notably, ROR and PRR algorithms demonstrate superior efficacy in early signal detection, while BCPNN and MGPS algorithms exhibit enhanced detection capabilities for less frequently reported ADRs [52]. In pharmacovigilance terminology, a ‘signal’ represents a reported association between a drug and an adverse event that warrants further investigation, detected when disproportionality metrics exceed pre-defined thresholds. Signal strength is quantified by the magnitude of the disproportionality measure: higher values indicate that the adverse event is reported more frequently with the drug of interest compared to all other drugs in the database, suggesting a potential causal association requiring clinical attention and further validation. However, signal detection does not establish causation or quantify absolute risk; it identifies associations deserving heightened surveillance and mechanistic investigation. All four algorithms are based on 2 × 2 contingency tables, with detailed formulas and thresholds documented in Table 4. Signals deemed significant by all four algorithms were considered positive. The magnitude of the signal detection value correlates positively with signal strength. To elucidate gender-specific differences in HDACi-related ADRs, we conducted chi-square tests based on the ROR algorithm, utilizing 2 × 2 contingency tables, and calculated *p*-values [53]. A *p*-value of less than 0.05 signifies a noteworthy disparity between genders. In such cases, a PT with an ROR greater than 1 suggested a higher susceptibility to the ADR in females, while values below 1 indicated a higher susceptibility in males.

### 4.3. TTO Analysis

Time to onset (TTO) is defined as the duration between the commencement of drug administration and the subsequent occurrence of ADRs. We included cases with accurate and complete date information for TTO analysis, employing median, quartiles, and extreme values for quantitative description. Furthermore, we utilized Weibull distribution analysis to evaluate the temporal evolution of HDACi-related ADR incidence rates. The Weibull distribution is defined by two essential parameters: the shape parameter (α) and the scale parameter (β). The α parameter is particularly effective in detecting signals that emerge shortly after treatment initiation. Our study focused primarily on the α parameter. A α < 1 with a 95% CI < 1 suggests a declining risk of adverse reactions over time, representing an early failure curve. Conversely, a α > 1 with a 95% CI > 1 suggests an increasing risk over time, characteristic of a wear-out failure curve. Finally, a α approximately equal to 1 with a 95% CI encompassing 1 denotes a random failure curve.

### 4.4. Logistics Regression

To reduce the impact of confounding variables on our results, we utilized both univariate and multivariate logistic regression analyses to examine the potential relationships between gender, age, and weight and the occurrence of significant medical events (IMEs). These IMEs included death, life-threatening conditions, hospitalization, disability, and congenital anomalies. In the multivariate logistic regression model, when one variable served as the independent variable, the remaining two were incorporated as covariates. Age and weight were treated as continuous variables when used as covariates. Statistical significance was determined using a two-sided *p*-value of less than 0.05.

### 4.5. Drug Target MR Analysis

In this study, we utilized drug target Mendelian randomization analysis to further explore the relationship between the administration of HDAC inhibitors and adverse reactions. Exposure-related genome-wide association studies (GWAS) data were sourced from significant cis-eQTLs regulating blood gene expression, provided by the eQTLGen Consortium [54]. The targets of Vorinostat, Belinostat, and Panobinostat include Class I (HDACs 1, 2, 3, and 8), Class IIa (HDACs 4, 5, 7, and 9), Class IIb (HDACs 6 and 10), and Class IV (HDAC 11), whereas Romidepsin specifically targets Class I HDACs. We selected single nucleotide polymorphisms (SNPs) within ± 500 kb of these gene loci as instrumental variables (IVs). The *F*-statistic for each (IV) was computed using the formula *F* = BETA^2^exposure/SE^2^exposure [55], with IVs yielding *F* < 10 being excluded. Additionally, we eliminated IVs in linkage disequilibrium (distance of 500 kb, r^2^ < 0.001 [56]). Outcome-related GWAS data were extracted from the GWAS Catalog [57], including white blood cell leukocyte count (GWAS ID: GCST90078978), platelet count (GCST90078987), neutrophil count (GCST90078993), serum phosphate levels (GCST90025948), calcium levels (GCST90079023), QT interval (GCST90165290), aspartate aminotransferase levels (GCST90079020), and pernicious anemia (GCST90077789). Palindromic SNPs were also excluded from the analysis. Table S2 provides comprehensive details regarding all SNPs included in the final analysis. We performed MR analysis employing five distinct methods: MR Egger, weighted median, inverse variance weighted (IVW), simple mode, and weighted mode. A *p*-value of less than 0.05 was regarded as indicative of a significant causal relationship.

All data analyses and visualizations were performed using Microsoft Excel 2016 and R software (Version 4.2.2).

## 5. Conclusions

In summary, this study, leveraging large-scale real-world safety data, provides an updated overview of HDACi-associated ADRs, with findings generally consistent with existing clinical observations. Our gender-stratified and temporal analyses further identified potential differences in reporting patterns, while age, gender, and weight emerged as possible confounding factors for IMEs. The observed disproportionality signals—particularly for hypophosphatemia—together with supportive Mendelian randomization evidence, should be interpreted as hypothesis-generating rather than definitive proof of causality. Given the inherent limitations of spontaneous reporting systems and genetic proxy analyses, these results are best viewed as signals warranting heightened clinical awareness and further validation. Future large-scale prospective studies and mechanistic investigations are required to clarify the magnitude, causality, and biological basis of these associations. Taken together, these findings suggest that routine monitoring of serum electrolytes—particularly phosphate levels—may be warranted during HDACi therapy, especially in the early treatment phase. Such proactive surveillance could facilitate timely intervention and risk mitigation in clinical practice. These findings bear significant clinical implications, offering valuable insights to clinicians for mitigating HDACi-associated ADRs through post-marketing safety evaluation. Moreover, this study lays the groundwork for further pharmacological investigations.

## Figures and Tables

**Table 1:** Demographic characteristics of ADRs reported in the FAERS database with HDACi as the primary suspect drug.

Characteristics	Vorinostat N = 1360 *n* (%)	Romidepsin N = 1065 *n* (%)	Belinostat N = 225 *n* (%)	Panobinostat N = 1234 *n* (%)
Gender				
Male	699 (51.40%)	519 (48.73%)	116 (51.56%)	490 (39.71%)
Female	558 (41.03%)	388 (36.43%)	82 (36.44%)	414 (33.55%)
Unknown	103 (7.57%)	158 (14.84%)	27 (12.00%)	330 (26.74%)
Age	56 (20) *	63 (15) *	60 (14) *	64 (16) *
<18 years	102 (7.50%)	4 (0.38%)	1 (0.44%)	28 (2.27%)
18–64 years	570 (41.91%)	336 (31.55%)	88 (39.11%)	231 (18.72%)
>64 years	471 (34.63%)	353 (33.15%)	60 (26.67%)	367 (29.74%)
Unknown	217 (15.96%)	372 (34.93%)	76 (33.78%)	608 (49.27%)
Weight	71 (24) *	71 (28) *	80 (22) *	72 (21) *
<80 kg	437 (32.13%)	310 (29.11%)	52 (23.11%)	160 (12.97%)
80–100 kg	153 (11.25%)	75 (7.04%)	26 (11.56%)	58 (4.70%)
>100 kg	55 (4.04%)	38 (3.57%)	13 (5.78%)	21 (1.70%)
Unknown	715 (52.57%)	642 (60.28%)	134 (59.56%)	995 (80.63%)
Reported person				
Physician	340 (25.00%)	591 (55.49%)	130 (57.78%)	509 (41.25%)
Other health-professional	398 (29.26%)	328 (30.80%)	33 (14.67%)	295 (23.91%)
Pharmacist	81 (5.96%)	98 (9.20%)	19 (8.44%)	63 (5.11%)
Consumer	350 (25.74%)	41 (3.85%)	43 (19.11%)	289 (23.42%)
Unknown	191 (14.04%)	7 (0.66%)	0 (0.00%)	78 (6.32%)
Reported country				
United States	940 (69.12%)	418 (39.25%)	138 (61.33%)	551 (44.65%)
Other country	420 (30.88%)	647 (60.75%)	87 (38.67%)	683 (55.35%)
Outcome				
Death	242 (17.79%)	344 (32.30%)	66 (29.33%)	343 (27.80%)
Life-threatening	185 (13.60%)	56 (5.26%)	9 (4.00%)	43 (3.48%)
Hospitalization	558 (41.03%)	276 (25.92%)	41 (18.22%)	289 (23.42%)
Disability	10 (0.74%)	7 (0.66%)	1 (0.44%)	6 (0.49%)
Other	145 (10.66%)	248 (23.29%)	50 (22.22%)	279 (22.61%)
Unknown	220 (16.18%)	134 (12.58%)	58 (25.78%)	274 (22.20%)

ADRs, adverse drug reactions; HDACi, histone deacetylase inhibitors. * Mean (SD).

**Table 2:** Time-to-onset analysis for signals.

Group	Number	TTO (Days)		Weibull Distribution				Failure Type
				Scale Parameter		Shape Parameter		
		Median (IQR)	Min-Max	β	95% CI	α	95% CI	
Vorinostat	1044	28.0 (10.0–75.0)	1–2052	60.17	54.65–65.69	0.70	0.67–0.73	Early failure
Romidepsin	451	29.0 (7.0–109.0)	1–2072	70.46	58.24–82.68	0.56	0.53–0.60	Early failure
Belinostat	66	19.5 (4.0–49.3)	1–228	28.34	18.87–37.81	0.76	0.62–0.91	Early failure
Panobinostat	515	18.0 (6.0–56.5)	1–3254	43.16	36.43–49.88	0.59	0.55–0.62	Early failure

TTO, Time-to-onset.

**Table 3:** Logistic regression models of IME.

Drug	Variable	Logistics Regression					
		Univariate			Multivariate ^†^		
		OR	95% CI	*p*	OR	95% CI	*p*
Vorinostat	Gender (Reference female)	1.49	1.15, 1.95	0.003 **	1.14	0.64, 2.04	0.7
	Age	1.01	1.01, 1.02	<0.001 **	0.99	0.97, 1.01	0.3
	Age (Reference < 18 years)						
	18–64 years	3.05	1.94, 4.77	<0.001	0.76	0.19, 2.63	0.7
	>64 years	2.63	1.66, 4.13	<0.001	0.80	0.20, 2.73	0.7
	Weight	1.01	1.00, 1.02	0.2	1.01	1.00, 1.03	0.15
	Weight (Reference < 80 KG)						
	80–100 kg	2.22	1.04, 5.50	0.058	2.27	1.04, 5.68	0.055
	>100 kg	1.84	0.64, 7.79	0.3	1.72	0.59, 7.28	0.4
Romidepsin	Gender (Reference female)	1.08	0.81, 1.43	0.6	0.98	0.61, 1.56	>0.9
	Age	1.00	0.99, 1.01	0.9	0.97	0.95, 0.99	0.004 **
	Age (Reference < 18 years)						
	18–64 years	9.60	1.21, 196	0.052	NA	NA	NA
	>64 years	7.38	0.93, 150	0.085	NA	NA	NA
	Weight	1.00	0.99, 1.01	0.8	1.00	0.99, 1.01	0.8
	Weight (Reference < 80 KG)						
	80–100 kg	1.37	0.76, 2.58	0.3	1.37	0.71, 2.82	0.4
	>100 kg	0.81	0.40, 1.72	0.6	0.75	0.34, 1.73	0.5
Belinostat	Gender (Reference female)	0.66	0.37, 1.17	0.2	0.24	0.08, 0.67	0.009 **
	Age	1.02	1.00, 1.05	0.072	1.03	0.98, 1.08	0.3
	Age (Reference < 18 years)						
	18–64 years	NA	NA	>0.9	NA	NA	NA
	>64 years	NA	NA	>0.9	2.00	0.71, 5.85	0.2
	Weight	0.99	0.96, 1.00	0.2	1.00	0.97, 1.02	0.8
	Weight (Reference < 80 KG)						
	80–100 kg	0.30	0.11, 0.80	0.018 *	0.46	0.14, 1.43	0.2
	>100 kg	0.67	0.19, 2.36	0.5	1.56	0.31, 9.53	0.6
Panobinostat	Gender (Reference female)	1.23	0.94, 1.61	0.13	0.96	0.44, 2.04	>0.9
	Age	1.00	0.98, 1.01	0.4	0.99	0.97, 1.01	0.4
	Age (Reference < 18 years)						
	18–64 years	0.92	0.37, 2.12	0.9	0.82	0.10, 4.57	0.8
	>64 years	0.88	0.35, 1.98	0.8	0.74	0.09, 3.93	0.7
	Weight	1.00	0.99, 1.02	0.5	1.01	0.99, 1.03	0.4
	Weight (Reference < 80 KG)						
	80–100 kg	1.92	0.80, 5.36	0.2	1.87	0.75, 5.39	0.2
	>100 kg	0.44	0.17, 1.26	0.11	0.62	0.21, 2.10	0.4

IME, important medical event. NA indicates failure of model convergence due to sparse data or quasi-complete separation in certain age-by-gender strata. Complete-case analysis was performed; no imputation methods were employed. ^†^ Two variables in addition to the independent variables are used as covariates to adjust. When age and weight are used as covariates, we include continuous variables. * *p*-value < 0.05, ** *p*-value < 0.01.

**Table 4:** Two-by-two contingency table and detailed formulas.

	Number of Target ADRs	Number of Other ADRs	Total
HDACi	a	b	a + b
Others	c	d	c + d
Total	a + c	b + d	a + b + c +d
Algorithm	Formula		Threshold
ROR	$ROR=\frac{\left(a∕c\right)}{\left(b∕d\right)}=\frac{ad}{bc}\;95\%\;CI=e\ln\left(ROR\right)\pm 1.96\sqrt{\left(\frac{1}{a}+\frac{1}{b}+\frac{1}{c}+\frac{1}{d}\right)}$		a ≥ 3 & lower limit of 95% CI > 1
PRR	$PRR=\frac{a∕\left(a+b\right)}{c∕\left(c+d\right)}\;\chi^{2}=\frac{\left(a+b+c+d\right){\left(ad-bc\right)}^{2}}{\left(a+b)(c+d)(a+c)(b+d\right)}\;95\%\;CI=e\ln\left(PRR\right)\pm 1.96\sqrt{\left(\frac{1}{a}-\frac{1}{a+b}+\frac{1}{c}-\frac{1}{c+d}\right)}$		a ≥ 3 & χ^2^ ≥ 4 & lower limit of 95% CI > 1
BCPNN	$95\%\;CI=E(IC)\pm 2\sqrt{V(IC)}$		a ≥ 3 & IC025 > 0
MGPS	$EBGM=\frac{a\left(a+b+c+d\right)}{\left(a+c\right)\left(a+b\right)}\;EBGM05=e\sqrt[{ln\left(EBGM\right)-1.64}]{\frac{1}{a}+\frac{1}{b}+\frac{1}{c}+\frac{1}{d}}$		a > 0 & EBGM05 > 2

ADRs, adverse drug reactions; HDACi, histone deacetylase inhibitors; a, number of reports containing both HDACi and the target ADRs; b, number of reports containing other ADRs of HDACi; c, number of reports containing the target ADRs of other drugs; d, number of reports containing other drugs and other ADRs. CI, confidence interval; χ^2^, chi-squared; ROR, reporting odds ratio; PRR, proportional reporting ratio; BCPNN, Bayesian confidence propagation neutral network; MGPS, multi-item gamma Poisson shrinker; IC, information component; IC025, the lower limit of the 95% CI of the IC; E (IC), the IC expectations; V (IC), the variance of IC; EBGM, empirical Bayesian geometric mean; EBGM05, the lower limit of the 95% CI of EBGM.

## Data Availability

The data presented in this study are openly available in the official FAERS website at https://open.fda.gov/data/faers/ (accessed on 7 September 2024) and the GWAS Catalog at https://www.ebi.ac.uk/gwas/home (accessed on 19 November 2024).

## Supplementary Materials

The following supporting information can be downloaded at: https://www.mdpi.com/article/10.3390/ph19050689/s1, Figure S1: The flowchart of the Pharmacoepidemiological Study; Table S1: Detailed list of drug names used for searching; Table S2: The information for finally enrolled SNPs; Table S3: Reported cases and signal strengths of HDACi at the SOC level; Table S4: Reported cases and signal strengths of HDACi at the PT level.

## Abbreviations

The following abbreviations are used in this manuscript:

ADR	adverse drug reaction
CTCL	cutaneous T-cell lymphoma
FAERS	FDA Adverse Event Reporting System
BCPNN	Bayesian Confidence Propagation Neural Network
HAT	histone acetyltransferases
IME	important medical events
PS	primary suspect
HDAC	histone deacetylases
HDACi	histone deacetylase inhibitors
IV	instrumental variable
IVW	Inverse variance weighted
TTO	Time to onset
MedDRA	Medical Dictionary for Regulatory Activities
PT	preferred term
GWAS	Genome-Wide Association Studies
MGPS	multi-item gamma Poisson shrinker
MM	multiple myeloma
MR	Mendelian randomization
PRR	proportional reporting ratio
SOC	system organ class
PTCL	peripheral T-cell lymphoma
ROR	reporting odds ratio
SNPs	single nucleotide polymorphisms
