# Supplementary material for: Hypophosphatemia as a Potential Class Effect of Histone Deacetylase Inhibitors: Evidence from Disproportionality Analysis and Mendelian Randomization Analysis of Drug Targets
Source: Pharmaceuticals (Basel). 2026 Apr 28;19(5):689. doi: 10.3390/ph19050689 (PMC13209236; doi:10.3390/ph19050689)
Supplement: Supplementary file 1 [file pharmaceuticals-19-00689-s001.zip › Table S1. Detailed list of drug names used for searching.pdf]

Table S1. Detailed list of drug names used for searching.

Vorinostat: 'VORINOSTAT', 'BLINDED VORINOSTAT', 'BLINDED VORINOSTAT', 'VORINOSTAT', 'ZOLINZA VORINOSTAT UNKNOWN ', 'VORINOSTAT VORINOSTAT ', 'VORINOSTAT MYCOSIS FUNGOIDES', 'VORINOSTAT MYCOSIS FUNGOIDES', 'ZOLINZA VORINOSTAT ', 'VORINOSTAT', 'VORINOSTAT 400MG', 'VORINOSTAT 400MG MERCK', 'VORINOSTAT 400MG PO', 'VORINOSTAT THERAPY', 'TN UNSPECIFIED VORINOSTAT ', 'CAP VORINOSTAT', 'CAP VORINOSTAT UNK', 'CAP VORINOSTAT', 'CAP VORINOSTAT 100 MG', 'VORINOSTAT SUBEROYLANILIDE HYDROXAMIC ACID ORAL DRUG UNSPECIFIED FOR', 'CAP VORINOSTAT UNK','VORINOSTAT ANTINEOPLASTIC AGENTS UNSPECIFIED', 'CAP VORINOSTAT 300 MG', 'VORINOSTAT UNK', 'VORINOSTAT ANTINEOPLASTIC AGENTS ', 'VORINOSTAT ANTINEOPLASTIC AGENTS CAPSULE', 'CAP VORINOSTAT 100MG', 'VORINOSTAT 400MG', 'VORINOSTAT 100MG CAPS', 'VORINOSTAT 100MG CAPSULE', 'CAP VORINOSTAT 400 MG', 'VORINOSTAT VORINOSTAT ', 'VORINOSTAT SUBEROYLANILIDE HYDROXAMIC ACID ORAL DRUG UNSPECIFIED FORM', 'CAP VORINOSTATE', 'CAP VORINOSTAT 200 MG', 'SAHA VORINOSTAT', 'CAP VORINOSTAT 400 MG 200 MG', 'VORINOSTAT 100 MG','VORINOSTAT SUBEROYLANILIDE HYDROXAMIC ACID ', 'VORINOSTAT SUBEROYLANILIDE HYDROXAMIC ACID ', 'VORINOSTAT VORINOSTAT UNSPECIFIED', 'VORINOSTAT VORINOSTAT CAPSULE', 'CAP VORINOSTAT 200 MG DAILY', 'VORINOSTAT 100 MG CAPSULE MERCK', 'SAHA VORINOSTAT 400MG D1 14 Q DAY', 'VORINOSTAT 100MG CAPSULE MERCK', 'SAHA VORINOSTAT 400MG DAY 1 14 QDAY', 'VORINOSTAT VORINOSTAT ORAL DRUG UNSPECIFIED FORM', 'SAHA VORINOSTAT ', 'VORINOSTAT PO', 'VORINOSTAT 100MG MERCK', 'ZAP ZOLINZA VORINOSTAT ', 'VORINOSTAT 300 MG MERCK', 'VORINOSTAT 400 MG','VORINOSTAT VORINOSTAT UNSPECIFIED', 'VORINOSTAT SAHA 300MG MERCK CO INC', 'VORINOSTAT 100MG', 'VORINOSTAT 300MG DAILY X 14 Q 21 DAYS ORAL', 'VORINOSTAT STUDY MED NOT GIVEN ', 'SAHA VORINOSTAT 400MG PO QD', 'INJ VORINOSTAT UNK','VORINOSTAT 400MG PO QD', 'VORINOSTAT 300MG PO DAYS 1 8', 'VORINOSTAT 100MG MERCK', 'VORINOSTAT SAHA 300MG MERCK CO ', 'VORINOSTAT 300MG DAILY DAYS 1 14 OF EACH CYCLE', 'VORINOSTAT 300 MG DAILY DAYS 1 14 OF EACH CYCLE', 'VORINOSTAT 300 MG PO BID DAYS 1 3 EACH WEEK', 'BLINDED VORINOSTAT', 'VORINOSTAT MERCK', 'VORINOSTAT 300MG PO', 'VORINOSTAT 300 MG PO', 'VORINOSTAT SAHA 400MG MERCK CO INC ', 'VORINOSTAT 200 MG','VORINOSTAT SAHA 400MG MERCK CO INC', 'ZOLINZA VORINOSTAT ', 'VORINOSTAT VORINOSTAT VORINOSTAT ', 'SAHA VORINOSTAT ', 'SUBEROYLANILIDE HYDROXAMIC ACID', 'VPROMPSTAT SUBEROYLANILIDE HYDROXAMIC ACID ORAL DRUG UNSPECIFIED FOR', 'VORINOSTAT SUBEROYLANILIDE HYDROXAMIC ACID ORAL DRUG UNSPECIFIED FOR', 'VORINOSTAT SUBEROYLANILIDE HYDROXAMIC ACID ORAL DRUG UNSPECIFIED FORM','VORINOSTAT SUBEROYLANILIDE HYDROXAMIC ACID ', 'VORINOSTAT SUBEROYLANILIDE HYDROXAMIC ACID ', 'MK 0683','MK 0683', 'MK 0683', 'ZOLINZA', 'ZOLINZA', 'ZOLINZA', 'ZOLINZA VORINOSTAT UNKNOWN ', 'ZOLINZA VORINOSTAT ', 'ZAP ZOLINZA VORINOSTAT ', 'ZOLINZA CAPSULES 100MG', 'ZOLINZA CAPSULES 100MG', 'ZOLINZA', 'ZOLINZA VORINOSTAT ', 'ZOLINZA', 'ZOLINZA', 'ZOLINZA', 'ZOLINZA VORINOSTAT UNKNOWN ', 'ZOLINZA VORINOSTAT ', 'ZAP ZOLINZA VORINOSTAT ', 'ZOLINZA CAPSULES 100MG', 'ZOLINZA CAPSULES 100MG', 'ZOLINZA', 'ZOLINZA VORINOSTAT '

Romidepsin: 'ROMIDEPSIN', 'ROMIDEPSIN', 'ROMIDEPSIN', 'ROMIDEPSINE', 'ROMIDEPSIN ', 'ROMIDEPSIN ', 'ROMIDEPSIN ', 'ROMIDEPSIN PFIZER ', 'ROMIDEPSIN PFIZER ', 'ROMIDEPSIN TEVA ',

'HYDROXYZINE MAGNESIUM RANITIDINE ROMIDEPSIN', 'ROMIDEPSIN DEPSIPEPTIDE ', 'ROMIDEPSIN DEPSIPEPTIDE POWDER EXCEPT DPO ', 'ROMIDEPSIN DEPSIPEPTIDE POWDER EXCEPT DPO ', 'ROMIDEPSIN ROMIDEPSIN UNKNOWN ', 'ROMIDEPSIN 10MG M 2 CELGENE ', 'ROMIDEPSIN 7MG M2', 'ROMIDEPSIN 8MG M 2', 'ISTODAX', 'ISTODAX', 'ISTODAX', 'DEPSIPEPTIDE FR901228 '

Belinostat: 'BELINOSTAT', 'BELINOSTAT', 'BELINOSTAT', 'BELINOSTAT INJECTION', 'BELINOSTAT IND', 'PXD101 ', 'PXD101 PXD101 ', 'PXD101 ', 'PXD101', 'BELEODAQ', 'BELEODAQ'

Panobinostat: 'PANOBINOSTAT', 'PANOBINOSTAT', 'PANOBINOSTAT PANOBINOSTAT UNKNOWN ', 'PANOBINOSTAT', 'PANOBINOSTAT 20 MG CAPSULE', 'PANOBINOSTAT 20 MG PO', 'PANOBINOSTAT LACTATE', 'PANOBINOSTAT 15 MG NOVARTIS PHARMACEUTICALS','PANOBINOSTAT', 'PANOBINOSTAT LACTATE', 'LBH589 PANOBINOSTAT ', 'PANOBINOSTAT CARFILZOMIB', 'LBH 589 PANOBINOSTAT ', 'PANOBINOSTAT FORMULATION UNKNOWN ', 'LABH 589 PANOBINOSTAT ', 'PANOBINOSTAT TABLET ', 'PANOBINOSTAT 10 MG', 'PANOBINOSTAT PANOBINOSTAT ', 'PANOBINOSTAT CAPS', 'PANOBINOSTAT PANOBINOSTAT PANOBINOSTAT ', 'PANOBINOSTAT 20 MG PO', 'LBH589', 'LBH589', 'BTZLBH589', 'LBH589 PANOBINOSTAT ', 'LBH589', 'LBH589 LBH CAP', 'LBH589 LBH ', 'BLINDED LBH589', 'LBH589B', 'LBH 589B', 'LBH 589 PANOBINOSTAT ', 'LBH 589B', 'LBH 589', 'FARYDAK', 'FARYDAK', 'FARYDAK', 'FARYDAK PAP', 'FARYDAK', 'FARYDAK', 'FARYDAK', 'FARYDAK', 'FARYDAK PAP', 'FARYDAK'
