# Supplementary material for: Hypophosphatemia as a Potential Class Effect of Histone Deacetylase Inhibitors: Evidence from Disproportionality Analysis and Mendelian Randomization Analysis of Drug Targets
Source: Pharmaceuticals (Basel). 2026 Apr 28;19(5):689. doi: 10.3390/ph19050689 (PMC13209236; doi:10.3390/ph19050689)
Supplement: Supplementary file 1 [file pharmaceuticals-19-00689-s001.zip › Table S2. The informations for finally enrolled SNPs.pdf]

Table S2. The informations for finally enrolled SNPs.

Exposure	Outcome	SNPs	EA-E	NEA-E	EAF	Beta-E	SE-E	*P*-E	*F*	EA-O	NEA-O	Beta-O	SE-O	*P*-O
HDAC1	Serum phosphate levels	rs114940035	T	C	0.033	0.357	0.063	2E-08	31.792	T	C	-0.009	0.006	0.088
HDAC1	Serum phosphate levels	rs11584199	C	T	0.055	0.101	0.018	2E-08	32.001	C	T	-0.004	0.005	0.240
HDAC1	Serum phosphate levels	rs16834897	G	A	0.027	-0.354	0.031	9E-30	128.455	G	A	-0.017	0.011	0.130
HDAC1	Serum phosphate levels	rs2273376	G	A	0.029	0.570	0.041	6E-45	197.830	G	A	0.005	0.006	0.580
HDAC1	Serum phosphate levels	rs2275434	T	C	0.037	0.153	0.025	2E-09	35.976	T	C	-0.002	0.006	0.700
HDAC1	Serum phosphate levels	rs34320757	C	A	0.043	-0.131	0.022	3E-09	35.303	C	A	0.008	0.004	0.048
HDAC1	Serum phosphate levels	rs35901955	A	G	0.038	-0.152	0.023	7E-11	42.516	A	G	0.005	0.007	0.490
HDAC1	Serum phosphate levels	rs35976845	T	C	0.091	0.115	0.017	8E-12	46.868	T	C	-0.007	0.004	0.051
HDAC1	Serum phosphate levels	rs3738007	T	C	0.066	0.139	0.017	4E-16	66.071	T	C	-0.005	0.004	0.250
HDAC1	Serum phosphate levels	rs3765153	G	A	0.021	-0.782	0.124	3E-10	39.826	G	A	-0.030	0.076	0.800
HDAC1	Serum phosphate levels	rs41263973	A	G	0.031	0.256	0.026	4E-23	98.212	A	G	0.002	0.006	0.600
HDAC1	Serum phosphate levels	rs479014	A	G	0.015	0.374	0.039	3E-22	93.985	A	G	0.005	0.010	0.730
HDAC1	Serum phosphate levels	rs563978	C	T	0.287	0.108	0.009	1E-34	151.065	C	T	0.003	0.002	0.260
HDAC1	Serum phosphate levels	rs587707	T	C	0.042	-0.161	0.020	3E-15	62.530	T	C	0.001	0.005	0.730
HDAC1	Serum phosphate levels	rs72662960	A	G	0.076	0.423	0.043	4E-23	98.236	A	G	0.007	0.004	0.100
HDAC1	Serum phosphate levels	rs74886700	T	C	0.043	0.240	0.024	8E-24	101.340	T	C	-0.008	0.005	0.087
HDAC1	Serum phosphate levels	rs77934418	T	C	0.023	0.197	0.030	4E-11	43.748	T	C	0.000	0.007	0.890
HDAC1	Serum phosphate levels	rs78230117	A	C	0.061	0.155	0.018	2E-18	76.314	A	C	-0.004	0.005	0.420
HDAC1	Serum phosphate levels	rs80272918	T	C	0.033	-0.354	0.064	3E-08	30.619	T	C	0.002	0.006	0.690
HDAC1	Serum phosphate levels	rs8176781	C	T	0.023	0.348	0.044	3E-15	62.315	C	T	0.013	0.006	0.021

HDAC1	QT interval	rs1004420	T	G	0.163	0.155	0.014	1E-26	114.344	T	G	0.175	0.149	0.240
HDAC1	QT interval	rs10914368	A	G	0.379	-0.168	0.009	2E-78	351.309	A	G	0.040	0.106	0.710
HDAC1	QT interval	rs10914443	G	T	0.306	-0.178	0.009	5E-92	413.788	G	T	-0.050	0.116	0.660
HDAC1	QT interval	rs10914470	T	C	0.259	-0.065	0.010	5E-11	43.328	T	C	-0.164	0.137	0.230
HDAC1	QT interval	rs1107550	G	A	0.261	-0.053	0.010	4E-08	30.373	G	A	0.104	0.128	0.410
HDAC1	QT interval	rs111977222	A	G	0.026	0.217	0.030	3E-13	53.562	A	G	0.771	0.487	0.110
HDAC1	QT interval	rs113715735	A	G	0.246	0.115	0.010	3E-30	130.901	A	G	0.034	0.145	0.810
HDAC1	QT interval	rs113724490	T	C	0.047	-0.142	0.021	6E-12	47.211	T	C	-0.244	0.248	0.330
HDAC1	QT interval	rs114487668	T	G	0.016	0.244	0.037	3E-11	43.883	T	G	-0.565	0.494	0.250
HDAC1	QT interval	rs114940035	T	C	0.033	0.357	0.063	2E-08	31.792	T	C	-0.205	0.353	0.560
HDAC1	QT interval	rs11584199	C	T	0.055	0.101	0.018	2E-08	32.001	C	T	0.001	0.270	1.000
HDAC1	QT interval	rs11589125	T	C	0.066	-0.543	0.016	1E-200	1176.496	T	C	-0.312	0.223	0.160
HDAC1	QT interval	rs116076876	T	C	0.028	0.696	0.068	1E-24	104.792	T	C	0.243	0.361	0.500
HDAC1	QT interval	rs116080631	A	G	0.040	-0.205	0.022	7E-20	83.332	A	G	-0.180	0.309	0.560
HDAC1	QT interval	rs11799701	G	A	0.062	0.671	0.072	2E-20	86.041	G	A	-0.751	0.513	0.140
HDAC1	QT interval	rs11806010	G	A	0.120	0.098	0.012	3E-15	62.546	G	A	0.135	0.161	0.400
HDAC1	QT interval	rs12039509	A	G	0.296	-0.118	0.010	9E-35	151.341	A	G	-0.134	0.116	0.250
HDAC1	QT interval	rs12117354	G	A	0.022	0.189	0.031	2E-09	36.424	G	A	0.102	0.452	0.820
HDAC1	QT interval	rs12734726	T	C	0.110	-0.238	0.013	1E-77	348.104	T	C	-0.325	0.164	0.047
HDAC1	QT interval	rs12737504	A	G	0.017	-0.359	0.043	4E-17	70.940	A	G	0.456	0.443	0.300
HDAC1	QT interval	rs13376569	C	T	0.062	0.426	0.057	9E-14	55.592	C	T	-0.265	0.372	0.480
HDAC1	QT interval	rs139268405	G	A	0.015	0.458	0.037	5E-36	157.001	G	A	0.554	0.402	0.170

HDAC1	QT interval	rs141766713	T	C	0.017	-0.250	0.036	2E-12	49.382	T	C	-0.071	0.510	0.890
HDAC1	QT interval	rs143641181	A	G	0.015	0.514	0.070	2E-13	53.632	A	G	1.240	0.478	0.010
HDAC1	QT interval	rs144058376	T	C	0.047	-0.269	0.021	1E-38	169.042	T	C	-0.398	0.324	0.220
HDAC1	QT interval	rs144924250	C	A	0.019	-0.314	0.033	5E-21	88.368	C	A	-0.445	0.500	0.370
HDAC1	QT interval	rs145550750	G	A	0.022	-0.230	0.031	2E-13	54.376	G	A	0.081	0.423	0.850
HDAC1	QT interval	rs148719434	C	T	0.033	-0.161	0.023	6E-12	47.219	C	T	0.440	0.296	0.140
HDAC1	QT interval	rs149734285	C	T	0.041	-0.354	0.060	3E-09	35.191	C	T	0.556	0.627	0.380
HDAC1	QT interval	rs150255180	C	A	0.019	0.212	0.034	5E-10	38.808	C	A	0.069	0.488	0.890
HDAC1	QT interval	rs150378436	G	A	0.022	-0.211	0.029	1E-13	54.831	G	A	-0.622	0.373	0.096
HDAC1	QT interval	rs150763245	G	A	0.020	0.904	0.103	1E-18	77.735	G	A	-0.609	0.563	0.280
HDAC1	QT interval	rs1536130	G	C	0.283	-0.114	0.009	2E-38	167.687	G	C	-0.055	0.111	0.620
HDAC1	QT interval	rs16834392	G	A	0.015	-0.568	0.084	1E-11	45.590	G	A	0.187	0.523	0.720
HDAC1	QT interval	rs16834897	G	A	0.027	-0.354	0.031	9E-30	128.455	G	A	0.049	0.349	0.890
HDAC1	QT interval	rs180696576	T	C	0.017	-0.347	0.038	5E-20	83.858	T	C	0.536	0.470	0.250
HDAC1	QT interval	rs187794144	A	G	0.014	0.497	0.085	5E-09	34.054	A	G	-0.178	0.838	0.830
HDAC1	QT interval	rs191520785	T	C	0.019	-0.274	0.033	5E-17	70.191	T	C	0.195	0.431	0.650
HDAC1	QT interval	rs192721084	G	A	0.012	0.550	0.078	2E-12	49.719	G	A	-0.862	0.940	0.360
HDAC1	QT interval	rs1934287	G	A	0.397	-0.141	0.008	5E-68	303.650	G	A	-0.024	0.104	0.820
HDAC1	QT interval	rs2184203	G	C	0.446	-0.106	0.008	6E-40	174.979	G	C	0.148	0.104	0.150
HDAC1	QT interval	rs2273376	G	A	0.029	0.570	0.041	6E-45	197.830	G	A	0.051	0.295	0.860
HDAC1	QT interval	rs2275434	T	C	0.037	0.153	0.025	2E-09	35.976	T	C	0.042	0.256	0.870
HDAC1	QT interval	rs2297681	A	G	0.459	0.073	0.009	2E-15	63.037	A	G	-0.096	0.123	0.430
HDAC1	QT interval	rs2377862	C	A	0.073	0.159	0.016	1E-22	95.884	C	A	0.016	0.189	0.930

HDAC1	QT interval	rs2475833	G	A	0.122	-0.128	0.012	9E-26	110.133	G	A	-0.034	0.157	0.830
HDAC1	QT interval	rs34320757	C	A	0.043	-0.131	0.022	3E-09	35.303	C	A	-0.139	0.267	0.600
HDAC1	QT interval	rs35049993	T	C	0.018	-0.305	0.038	1E-15	63.850	T	C	0.830	0.742	0.260
HDAC1	QT interval	rs35132510	G	A	0.131	0.092	0.013	2E-12	49.635	G	A	-0.071	0.189	0.710
HDAC1	QT interval	rs35901955	A	G	0.038	-0.152	0.023	7E-11	42.516	A	G	0.034	0.460	0.940
HDAC1	QT interval	rs35976845	T	C	0.091	0.115	0.017	8E-12	46.868	T	C	-0.069	0.241	0.770
HDAC1	QT interval	rs36212119	C	T	0.070	-0.091	0.016	2E-08	31.497	C	T	-0.358	0.220	0.100
HDAC1	QT interval	rs3738007	T	C	0.066	0.139	0.017	4E-16	66.071	T	C	-0.112	0.212	0.600
HDAC1	QT interval	rs41263973	A	G	0.031	0.256	0.026	4E-23	98.212	A	G	0.536	0.352	0.130
HDAC1	QT interval	rs4245638	T	G	0.213	-0.075	0.010	2E-14	58.958	T	G	0.060	0.126	0.640
HDAC1	QT interval	rs4448564	A	C	0.249	0.363	0.026	5E-45	198.224	A	C	0.005	0.121	0.960
HDAC1	QT interval	rs45611233	A	G	0.174	-0.158	0.011	4E-46	203.529	A	G	-0.186	0.146	0.200
HDAC1	QT interval	rs479014	A	G	0.015	0.374	0.039	3E-22	93.985	A	G	-0.345	0.563	0.540
HDAC1	QT interval	rs4949489	T	C	0.023	0.175	0.031	2E-08	31.214	T	C	0.418	0.402	0.300
HDAC1	QT interval	rs55767537	C	T	0.183	-0.244	0.010	1E-122	555.083	C	T	-0.116	0.133	0.390
HDAC1	QT interval	rs563978	C	T	0.287	0.108	0.009	1E-34	151.065	C	T	-0.001	0.115	0.990
HDAC1	QT interval	rs57677732	A	G	0.036	-0.317	0.023	9E-42	183.385	A	G	0.053	0.283	0.850
HDAC1	QT interval	rs587707	T	C	0.042	-0.161	0.020	3E-15	62.530	T	C	0.038	0.277	0.890
HDAC1	QT interval	rs59532938	G	A	0.049	-0.295	0.020	2E-50	222.685	G	A	-0.387	0.231	0.094
HDAC1	QT interval	rs59756989	T	C	0.056	0.379	0.049	1E-14	59.158	T	C	0.126	0.222	0.570
HDAC1	QT interval	rs60441581	G	T	0.318	0.219	0.008	4E-148	671.919	G	T	-0.075	0.112	0.500

HDAC1	QT interval	rs60694879	A	G	0.072	0.088	0.015	1E-08	32.222	A	G	-0.160	0.196	0.410
HDAC1	QT interval	rs61099507	G	A	0.181	-0.078	0.010	5E-14	56.903	G	A	0.121	0.131	0.360
HDAC1	QT interval	rs61780805	T	C	0.039	-0.305	0.021	7E-48	211.430	T	C	0.040	0.266	0.880
HDAC1	QT interval	rs61781671	G	A	0.017	-0.413	0.070	4E-09	34.664	G	A	0.227	0.839	0.790
HDAC1	QT interval	rs6425781	G	A	0.133	0.086	0.012	1E-13	54.706	G	A	-0.007	0.170	0.970
HDAC1	QT interval	rs646689	A	G	0.115	0.206	0.017	4E-34	148.557	A	G	0.164	0.176	0.350
HDAC1	QT interval	rs6680160	A	G	0.377	-0.101	0.008	1E-34	150.314	A	G	-0.109	0.107	0.310
HDAC1	QT interval	rs673495	T	G	0.349	0.052	0.009	5E-09	34.394	T	G	0.088	0.114	0.440
HDAC1	QT interval	rs680328	G	A	0.166	0.115	0.015	3E-15	62.354	G	A	0.141	0.134	0.290
HDAC1	QT interval	rs72660842	A	G	0.102	0.273	0.014	4E-83	372.996	A	G	0.000	0.200	1.000
HDAC1	QT interval	rs72660893	G	C	0.091	0.495	0.039	6E-37	161.356	G	C	0.031	0.185	0.870
HDAC1	QT interval	rs72662920	A	G	0.014	0.292	0.042	3E-12	48.894	A	G	-0.398	0.533	0.450
HDAC1	QT interval	rs72662960	A	G	0.076	0.423	0.043	4E-23	98.236	A	G	-0.007	0.204	0.970
HDAC1	QT interval	rs72666724	A	G	0.047	0.125	0.021	2E-09	36.515	A	G	0.016	0.249	0.950
HDAC1	QT interval	rs72666752	G	A	0.081	-0.323	0.015	1E-99	449.304	G	A	0.031	0.195	0.870
HDAC1	QT interval	rs74578012	G	A	0.014	0.288	0.045	2E-10	40.750	G	A	0.302	0.497	0.540
HDAC1	QT interval	rs74886700	T	C	0.043	0.240	0.024	8E-24	101.340	T	C	-0.238	0.229	0.300
HDAC1	QT interval	rs7544140	A	G	0.451	0.064	0.009	2E-13	54.565	A	G	0.037	0.116	0.750
HDAC1	QT interval	rs76583934	C	A	0.047	0.187	0.020	2E-20	85.393	C	A	-0.061	0.272	0.820
HDAC1	QT interval	rs77934418	T	C	0.023	0.197	0.030	4E-11	43.748	T	C	-0.158	0.369	0.670
HDAC1	QT interval	rs78230117	A	C	0.061	0.155	0.018	2E-18	76.314	A	C	0.195	0.254	0.440
HDAC1	QT interval	rs78621080	A	G	0.115	-0.069	0.013	3E-08	30.433	A	G	-0.413	0.157	0.009
HDAC1	QT interval	rs79815286	T	G	0.017	0.284	0.044	9E-11	41.944	T	G	0.214	0.397	0.590

HDAC1	QT interval	rs80272918	T	C	0.033	-0.354	0.064	3E-08	30.619	T	C	0.046	0.374	0.900
HDAC1	QT interval	rs8176781	C	T	0.023	0.348	0.044	3E-15	62.315	C	T	-0.226	0.424	0.590
HDAC1	QT interval	rs951545	A	G	0.138	-0.177	0.013	1E-44	196.464	A	G	0.074	0.147	0.620
HDAC1	QT interval	rs9728705	G	T	0.069	-0.163	0.018	7E-19	78.702	G	T	-0.211	0.302	0.480
HDAC2	QT interval	rs147081792	A	G	0.016	-0.340	0.062	5E-08	29.882	A	G	-0.975	0.517	0.059
HDAC2	QT interval	rs149003802	A	G	0.033	0.203	0.033	1E-09	37.066	A	G	-0.145	0.315	0.650
HDAC2	QT interval	rs17772683	T	C	0.171	0.151	0.016	5E-22	93.112	T	C	-0.304	0.135	0.024
HDAC2	QT interval	rs55677919	G	A	0.445	0.075	0.012	5E-10	38.828	G	A	-0.082	0.103	0.430
HDAC2	QT interval	rs7341241	C	A	0.133	0.096	0.017	4E-08	30.107	C	A	-0.028	0.153	0.850
HDAC2	QT interval	rs73543855	A	G	0.102	0.208	0.019	1E-26	114.216	A	G	-0.086	0.176	0.620
HDAC2	QT interval	rs78382519	T	C	0.106	-0.151	0.019	4E-15	61.757	T	C	0.011	0.171	0.950
HDAC2	QT interval	rs9285411	T	A	0.323	0.197	0.013	2E-55	245.668	T	A	0.014	0.112	0.900
HDAC2	QT interval	rs9384867	A	G	0.352	-0.175	0.012	7E-46	202.060	A	G	0.027	0.107	0.800
HDAC2	QT interval	rs9400641	A	G	0.213	-0.090	0.014	5E-10	38.713	A	G	-0.038	0.121	0.750
HDAC3	Serum phosphate levels	rs111260438	A	G	0.040	0.139	0.023	8E-10	37.741	A	G	-0.008	0.005	0.079
HDAC3	Serum phosphate levels	rs114831305	T	C	0.021	0.309	0.035	1E-18	77.433	T	C	-0.002	0.006	0.600
HDAC3	Serum phosphate levels	rs11542702	T	C	0.071	0.126	0.019	2E-11	45.328	T	C	0.002	0.004	0.420
HDAC3	Serum phosphate levels	rs115436949	C	T	0.030	-0.163	0.024	1E-11	45.803	C	T	-0.001	0.006	0.870
HDAC3	Serum phosphate levels	rs116642057	T	C	0.021	-0.255	0.035	2E-13	53.729	T	C	0.005	0.007	0.600
HDAC3	Serum phosphate levels	rs11750870	G	A	0.025	-0.285	0.027	2E-25	108.897	G	A	0.016	0.007	0.014
HDAC3	Serum phosphate levels	rs12189346	G	A	0.180	0.218	0.011	1E-90	407.712	G	A	0.009	0.003	0.004
HDAC3	Serum phosphate levels	rs12658441	C	T	0.232	0.104	0.009	1E-28	123.286	C	T	-0.001	0.002	0.610
HDAC3	Serum phosphate levels	rs14449	T	C	0.035	-0.184	0.023	1E-15	64.083	T	C	-0.003	0.006	0.550

HDAC3	Serum phosphate levels	rs147402888	C	A	0.017	-0.311	0.040	1E-14	59.991	C	A	-0.002	0.009	0.740
HDAC3	Serum phosphate levels	rs164078	G	T	0.395	0.060	0.010	7E-10	37.899	G	T	0.001	0.002	0.730
HDAC3	Serum phosphate levels	rs164080	A	G	0.467	-0.079	0.008	2E-22	95.445	A	G	0.001	0.002	0.660
HDAC3	Serum phosphate levels	rs17706123	G	A	0.049	0.102	0.018	3E-08	30.612	G	A	0.010	0.006	0.086
HDAC3	Serum phosphate levels	rs1898679	C	T	0.413	0.109	0.011	1E-24	105.129	C	T	0.001	0.002	0.800
HDAC3	Serum phosphate levels	rs2232195	G	A	0.251	0.052	0.009	2E-08	31.259	G	A	0.001	0.003	0.720
HDAC3	Serum phosphate levels	rs252103	G	A	0.024	0.590	0.029	1E-90	407.379	G	A	-0.012	0.008	0.082
HDAC3	Serum phosphate levels	rs252109	T	C	0.136	-0.102	0.012	2E-18	76.612	T	C	-0.002	0.003	0.500
HDAC3	Serum phosphate levels	rs32928	T	C	0.178	-0.111	0.010	1E-26	113.874	T	C	0.001	0.003	0.760
HDAC3	Serum phosphate levels	rs34636888	T	C	0.014	-0.522	0.041	6E-38	165.854	T	C	0.003	0.010	0.800
HDAC3	Serum phosphate levels	rs364287	G	C	0.224	-0.082	0.010	4E-15	61.626	G	C	0.000	0.002	0.930
HDAC3	Serum phosphate levels	rs41290613	A	G	0.019	-0.370	0.034	9E-28	119.229	A	G	0.005	0.008	0.610
HDAC3	Serum phosphate levels	rs445430	C	A	0.025	-0.247	0.028	3E-18	75.989	C	A	-0.005	0.007	0.460
HDAC3	Serum phosphate levels	rs58900279	T	C	0.057	-0.117	0.020	3E-09	34.923	T	C	0.001	0.004	0.820
HDAC3	Serum phosphate levels	rs62380004	G	A	0.082	0.107	0.015	5E-13	52.273	G	A	-0.002	0.004	0.670
HDAC3	Serum phosphate levels	rs72640104	C	A	0.033	-0.144	0.025	7E-09	33.552	C	A	-0.001	0.008	0.970
HDAC3	Serum phosphate levels	rs73288917	G	A	0.106	-0.131	0.013	5E-24	102.038	G	A	-0.003	0.004	0.500
HDAC3	Serum phosphate levels	rs756537	A	C	0.053	0.114	0.018	1E-10	41.101	A	C	-0.004	0.005	0.600
HDAC3	Serum phosphate levels	rs75774668	A	G	0.020	-0.274	0.033	9E-17	69.189	A	G	0.000	0.007	0.900
HDAC3	Serum phosphate levels	rs76814825	G	A	0.040	0.157	0.021	2E-13	54.055	G	A	-0.003	0.006	0.670
HDAC3	Serum phosphate levels	rs76933395	C	T	0.020	-0.226	0.032	2E-12	49.090	C	T	-0.003	0.007	0.780
HDAC3	Serum phosphate levels	rs7705576	C	G	0.235	-0.105	0.009	5E-29	124.904	C	G	0.002	0.002	0.420
HDAC3	Serum phosphate levels	rs78547804	T	C	0.110	0.080	0.013	6E-10	38.190	T	C	0.001	0.003	0.710

HDAC3	Serum phosphate levels	rs78950299	T	C	0.083	-0.201	0.016	2E-37	163.984	T	C	0.000	0.004	0.900
HDAC3	Serum phosphate levels	rs80178004	A	G	0.024	0.214	0.026	6E-16	65.435	A	G	0.010	0.007	0.059
HDAC3	Serum phosphate levels	rs80318397	A	G	0.073	-0.097	0.015	4E-10	39.365	A	G	0.003	0.004	0.530
HDAC3	QT interval	rs10075594	A	G	0.251	0.085	0.009	4E-20	84.596	A	G	-0.132	0.118	0.260
HDAC3	QT interval	rs111260438	A	G	0.040	0.139	0.023	8E-10	37.741	A	G	-0.423	0.292	0.150
HDAC3	QT interval	rs111543038	T	C	0.022	0.181	0.030	3E-09	35.357	T	C	-0.235	0.375	0.530
HDAC3	QT interval	rs11167766	C	G	0.494	-0.052	0.008	2E-10	40.689	C	G	-0.117	0.106	0.270
HDAC3	QT interval	rs112784197	T	C	0.022	0.278	0.033	9E-17	69.239	T	C	0.176	0.328	0.590
HDAC3	QT interval	rs113351491	G	A	0.043	0.143	0.023	4E-10	39.090	G	A	-0.202	0.281	0.470
HDAC3	QT interval	rs113629976	T	C	0.066	0.163	0.016	4E-23	98.196	T	C	-0.182	0.208	0.380
HDAC3	QT interval	rs114117766	T	C	0.017	0.316	0.033	2E-22	94.505	T	C	-0.752	0.441	0.088
HDAC3	QT interval	rs114269378	T	C	0.016	0.359	0.056	2E-10	40.646	T	C	0.490	0.480	0.310
HDAC3	QT interval	rs114831305	T	C	0.021	0.309	0.035	1E-18	77.433	T	C	0.240	0.327	0.460
HDAC3	QT interval	rs114968533	A	G	0.020	0.208	0.035	2E-09	35.667	A	G	-0.071	0.355	0.840
HDAC3	QT interval	rs115034640	G	A	0.015	0.588	0.040	6E-49	216.290	G	A	0.100	0.428	0.820
HDAC3	QT interval	rs11542702	T	C	0.071	0.126	0.019	2E-11	45.328	T	C	0.079	0.187	0.670
HDAC3	QT interval	rs115436949	C	T	0.030	-0.163	0.024	1E-11	45.803	C	T	0.162	0.296	0.580
HDAC3	QT interval	rs115459895	T	C	0.031	0.214	0.025	3E-18	75.961	T	C	0.437	0.354	0.220
HDAC3	QT interval	rs115634191	A	G	0.018	-0.224	0.034	2E-11	44.847	A	G	-0.288	0.440	0.510
HDAC3	QT interval	rs115986265	T	C	0.030	-0.156	0.024	4E-11	43.780	T	C	0.163	0.312	0.600
HDAC3	QT interval	rs116490745	G	T	0.016	-0.559	0.035	9E-57	252.061	G	T	0.140	0.465	0.760
HDAC3	QT interval	rs116642057	T	C	0.021	-0.255	0.035	2E-13	53.729	T	C	-0.276	0.423	0.510
HDAC3	QT interval	rs116760964	C	A	0.015	-0.255	0.042	1E-09	36.838	C	A	-0.135	0.403	0.740

HDAC3	QT interval	rs11740826	T	C	0.020	0.175	0.032	3E-08	30.459	T	C	0.754	0.414	0.069
HDAC3	QT interval	rs11749747	A	G	0.488	0.097	0.008	2E-33	145.058	A	G	-0.021	0.105	0.840
HDAC3	QT interval	rs11750870	G	A	0.025	-0.285	0.027	2E-25	108.897	G	A	-0.377	0.373	0.310
HDAC3	QT interval	rs12108935	A	G	0.101	-0.159	0.014	2E-30	131.680	A	G	-0.083	0.176	0.640
HDAC3	QT interval	rs12189346	G	A	0.180	0.218	0.011	1E-90	407.712	G	A	0.083	0.136	0.540
HDAC3	QT interval	rs12518743	T	C	0.120	-0.143	0.012	2E-31	136.306	T	C	0.235	0.156	0.130
HDAC3	QT interval	rs12658441	C	T	0.232	0.104	0.009	1E-28	123.286	C	T	-0.029	0.128	0.820
HDAC3	QT interval	rs13158321	A	G	0.073	-0.166	0.015	1E-27	118.470	A	G	-0.063	0.192	0.740
HDAC3	QT interval	rs13184739	A	G	0.226	0.113	0.010	5E-32	138.951	A	G	-0.159	0.123	0.200
HDAC3	QT interval	rs140491105	A	G	0.026	-0.192	0.028	1E-11	46.004	A	G	0.394	0.353	0.260
HDAC3	QT interval	rs140806892	A	G	0.024	-0.638	0.029	3E-105	474.498	A	G	0.251	0.443	0.570
HDAC3	QT interval	rs141627459	A	G	0.030	-0.214	0.027	3E-15	62.461	A	G	0.098	0.276	0.720
HDAC3	QT interval	rs141815925	G	T	0.012	-0.409	0.062	4E-11	43.795	G	T	0.117	0.697	0.870
HDAC3	QT interval	rs144268375	C	T	0.028	-0.147	0.027	4E-08	30.200	C	T	0.441	0.330	0.180
HDAC3	QT interval	rs144459785	T	C	0.016	0.243	0.041	2E-09	35.880	T	C	-0.848	0.472	0.073
HDAC3	QT interval	rs14449	T	C	0.035	-0.184	0.023	1E-15	64.083	T	C	-0.629	0.325	0.053
HDAC3	QT interval	rs145239159	A	G	0.018	0.251	0.033	2E-14	58.302	A	G	0.615	0.387	0.110
HDAC3	QT interval	rs146130495	C	A	0.015	0.336	0.041	2E-16	67.608	C	A	-0.002	0.459	1.000
HDAC3	QT interval	rs147402888	C	A	0.017	-0.311	0.040	1E-14	59.991	C	A	-0.067	0.489	0.890
HDAC3	QT interval	rs148365217	A	G	0.013	0.558	0.093	2E-09	36.284	A	G	-2.440	1.320	0.065
HDAC3	QT interval	rs149883072	C	A	0.026	0.262	0.029	1E-19	81.841	C	A	-0.216	0.336	0.520
HDAC3	QT interval	rs149970548	T	G	0.025	-0.293	0.031	5E-21	88.544	T	G	0.205	0.344	0.550

HDAC3	QT interval	rs150900700	A	G	0.013	-0.728	0.069	3E-26	112.305	A	G	0.559	0.673	0.410
HDAC3	QT interval	rs153154	T	C	0.108	-0.108	0.013	1E-16	68.235	T	C	0.003	0.172	0.990
HDAC3	QT interval	rs164078	G	T	0.395	0.060	0.010	7E-10	37.899	G	T	0.071	0.114	0.540
HDAC3	QT interval	rs164080	A	G	0.467	-0.079	0.008	2E-22	95.445	A	G	0.053	0.102	0.600
HDAC3	QT interval	rs164082	T	C	0.478	-0.104	0.008	2E-36	158.835	T	C	-0.075	0.110	0.500
HDAC3	QT interval	rs164498	T	C	0.391	-0.057	0.008	1E-11	46.023	T	C	0.062	0.113	0.580
HDAC3	QT interval	rs164501	T	G	0.138	0.117	0.013	4E-20	84.272	T	G	-0.020	0.169	0.910
HDAC3	QT interval	rs164508	T	C	0.057	-0.134	0.018	6E-14	56.220	T	C	0.048	0.225	0.830
HDAC3	QT interval	rs166040	A	G	0.125	-0.072	0.012	2E-09	35.791	A	G	0.226	0.144	0.120
HDAC3	QT interval	rs168331	C	A	0.331	-0.282	0.009	1E-200	1089.022	C	A	-0.003	0.118	0.980
HDAC3	QT interval	rs17097553	T	C	0.267	0.089	0.009	5E-22	93.018	T	C	-0.095	0.122	0.440
HDAC3	QT interval	rs17098224	T	G	0.282	0.158	0.009	2E-71	319.001	T	G	0.014	0.113	0.900
HDAC3	QT interval	rs17208586	A	G	0.024	-0.285	0.030	6E-21	88.078	A	G	0.196	0.422	0.640
HDAC3	QT interval	rs17610129	T	G	0.051	-0.146	0.018	7E-16	65.034	T	G	0.069	0.239	0.770
HDAC3	QT interval	rs17611767	A	G	0.046	0.161	0.019	3E-17	71.571	A	G	0.795	0.245	0.001
HDAC3	QT interval	rs17706123	G	A	0.049	0.102	0.018	3E-08	30.612	G	A	0.137	0.239	0.570
HDAC3	QT interval	rs182595515	A	G	0.013	-0.636	0.103	6E-10	38.306	A	G	1.470	0.800	0.066
HDAC3	QT interval	rs183508516	T	C	0.044	0.138	0.020	1E-12	50.081	T	C	-0.153	0.242	0.530
HDAC3	QT interval	rs188340329	A	G	0.027	-0.295	0.028	5E-26	111.269	A	G	0.284	0.308	0.360
HDAC3	QT interval	rs1898679	C	T	0.413	0.109	0.011	1E-24	105.129	C	T	-0.073	0.117	0.540
HDAC3	QT interval	rs191427148	A	C	0.035	0.373	0.027	3E-44	194.910	A	C	-0.422	0.375	0.260
HDAC3	QT interval	rs192436476	C	T	0.027	0.467	0.081	1E-08	32.865	C	T	1.550	2.170	0.480

HDAC3	QT interval	rs2232195	G	A	0.251	0.052	0.009	2E-08	31.259	G	A	-0.035	0.125	0.780
HDAC3	QT interval	rs2438594	C	T	0.174	0.138	0.011	2E-39	172.870	C	T	0.234	0.135	0.083
HDAC3	QT interval	rs248478	A	T	0.083	0.084	0.014	8E-09	33.238	A	T	-0.113	0.196	0.560
HDAC3	QT interval	rs248539	G	A	0.384	0.059	0.008	4E-13	52.624	G	A	-0.009	0.104	0.930
HDAC3	QT interval	rs249652	C	T	0.140	0.120	0.011	8E-26	110.401	C	T	0.056	0.136	0.680
HDAC3	QT interval	rs249658	T	C	0.264	0.073	0.009	9E-16	64.597	T	C	0.002	0.113	0.990
HDAC3	QT interval	rs252094	G	A	0.390	-0.058	0.010	4E-09	34.471	G	A	-0.169	0.118	0.150
HDAC3	QT interval	rs252103	G	A	0.024	0.590	0.029	1E-90	407.379	G	A	-0.159	0.417	0.700
HDAC3	QT interval	rs252109	T	C	0.136	-0.102	0.012	2E-18	76.612	T	C	-0.101	0.155	0.510
HDAC3	QT interval	rs252141	C	T	0.330	0.467	0.008	1E-200	3261.746	C	T	-0.131	0.109	0.230
HDAC3	QT interval	rs254437	C	T	0.280	0.056	0.009	4E-10	39.268	C	T	-0.067	0.113	0.550
HDAC3	QT interval	rs2906080	A	G	0.458	-0.131	0.008	1E-59	265.117	A	G	0.131	0.107	0.220
HDAC3	QT interval	rs2906082	C	T	0.214	0.063	0.010	1E-10	41.716	C	T	0.267	0.122	0.029
HDAC3	QT interval	rs2907295	T	C	0.027	0.185	0.027	3E-12	48.631	T	C	-0.241	0.385	0.530
HDAC3	QT interval	rs2908533	T	C	0.120	-0.096	0.012	7E-15	60.578	T	C	-0.241	0.160	0.130
HDAC3	QT interval	rs2913920	C	T	0.233	-0.064	0.009	1E-11	46.120	C	T	0.256	0.124	0.038
HDAC3	QT interval	rs2961709	C	T	0.449	-0.098	0.008	3E-34	148.664	C	T	0.086	0.103	0.400
HDAC3	QT interval	rs32928	T	C	0.178	-0.111	0.010	1E-26	113.874	T	C	-0.124	0.136	0.360
HDAC3	QT interval	rs33947258	A	C	0.251	0.082	0.009	5E-19	79.634	A	C	0.067	0.121	0.580
HDAC3	QT interval	rs34578382	A	G	0.042	-0.151	0.022	4E-12	48.064	A	G	-0.075	0.268	0.780
HDAC3	QT interval	rs34636888	T	C	0.014	-0.522	0.041	6E-38	165.854	T	C	-0.396	0.482	0.410
HDAC3	QT interval	rs34802011	T	C	0.021	0.176	0.029	1E-09	36.811	T	C	-0.123	0.412	0.760

HDAC3	QT interval	rs351259	C	T	0.323	-0.224	0.009	1E-131	596.042	C	T	-0.073	0.117	0.530
HDAC3	QT interval	rs35817396	G	T	0.114	-0.164	0.012	3E-39	171.578	G	T	0.174	0.168	0.300
HDAC3	QT interval	rs35896457	C	T	0.077	0.130	0.016	1E-15	63.732	C	T	0.244	0.190	0.200
HDAC3	QT interval	rs364287	G	C	0.224	-0.082	0.010	4E-15	61.626	G	C	-0.182	0.131	0.170
HDAC3	QT interval	rs3761762	A	G	0.118	0.250	0.013	1E-88	398.531	A	G	0.132	0.173	0.440
HDAC3	QT interval	rs3815355	C	T	0.254	0.061	0.009	5E-11	43.005	C	T	0.031	0.121	0.800
HDAC3	QT interval	rs3910203	G	A	0.397	0.058	0.008	9E-13	51.028	G	A	0.060	0.102	0.560
HDAC3	QT interval	rs39991	C	T	0.271	0.073	0.009	3E-16	66.782	C	T	-0.106	0.115	0.360
HDAC3	QT interval	rs41290611	A	G	0.016	-0.307	0.039	2E-15	63.314	A	G	0.200	0.442	0.650
HDAC3	QT interval	rs41290613	A	G	0.019	-0.370	0.034	9E-28	119.229	A	G	-0.248	0.480	0.600
HDAC3	QT interval	rs445310	G	A	0.179	-0.088	0.010	2E-17	72.594	G	A	-0.038	0.140	0.790
HDAC3	QT interval	rs445430	C	A	0.025	-0.247	0.028	3E-18	75.989	C	A	0.412	0.338	0.220
HDAC3	QT interval	rs4912797	T	C	0.347	0.131	0.008	3E-55	245.330	T	C	0.187	0.107	0.079
HDAC3	QT interval	rs4912810	T	C	0.074	0.089	0.015	6E-09	33.917	T	C	-0.105	0.173	0.540
HDAC3	QT interval	rs55725639	G	A	0.102	-0.171	0.014	4E-32	139.235	G	A	-0.018	0.185	0.920
HDAC3	QT interval	rs58900279	T	C	0.057	-0.117	0.020	3E-09	34.923	T	C	0.222	0.211	0.290
HDAC3	QT interval	rs59646836	T	G	0.306	-0.081	0.009	6E-21	88.217	T	G	-0.064	0.116	0.580
HDAC3	QT interval	rs617384	C	A	0.500	-0.064	0.008	4E-15	61.692	C	A	0.181	0.101	0.073
HDAC3	QT interval	rs62379435	C	T	0.053	-0.101	0.018	2E-08	31.277	C	T	0.184	0.220	0.400
HDAC3	QT interval	rs62380004	G	A	0.082	0.107	0.015	5E-13	52.273	G	A	0.158	0.192	0.410
HDAC3	QT interval	rs62381448	A	C	0.021	0.235	0.036	4E-11	43.589	A	C	0.197	0.484	0.680

HDAC3	QT interval	rs62381978	G	A	0.366	-0.223	0.008	2E-164	747.043	G	A	-0.046	0.107	0.670
HDAC3	QT interval	rs658868	G	C	0.233	-0.087	0.010	5E-17	70.308	G	C	0.034	0.132	0.800
HDAC3	QT interval	rs71587207	G	C	0.078	0.101	0.016	2E-10	40.815	G	C	0.508	0.180	0.005
HDAC3	QT interval	rs72640104	C	A	0.033	-0.144	0.025	7E-09	33.552	C	A	-0.217	0.294	0.460
HDAC3	QT interval	rs72794023	C	T	0.143	0.068	0.011	2E-09	36.091	C	T	-0.235	0.151	0.120
HDAC3	QT interval	rs72796006	A	C	0.046	-0.133	0.019	2E-12	49.202	A	C	-0.129	0.232	0.580
HDAC3	QT interval	rs72796010	G	A	0.044	-0.163	0.023	4E-12	48.236	G	A	-0.157	0.294	0.590
HDAC3	QT interval	rs72796024	A	G	0.062	-0.225	0.018	3E-35	153.706	A	G	-0.593	0.202	0.003
HDAC3	QT interval	rs72796079	G	A	0.084	0.309	0.014	4E-101	455.751	G	A	-0.254	0.179	0.150
HDAC3	QT interval	rs72799938	G	A	0.058	-0.124	0.018	6E-12	47.280	G	A	0.091	0.256	0.720
HDAC3	QT interval	rs72801932	A	G	0.064	0.123	0.016	5E-14	56.776	A	G	0.189	0.202	0.350
HDAC3	QT interval	rs73288917	G	A	0.106	-0.131	0.013	5E-24	102.038	G	A	-0.329	0.170	0.054
HDAC3	QT interval	rs735683	T	G	0.246	0.168	0.009	5E-75	335.966	T	G	0.064	0.115	0.580
HDAC3	QT interval	rs74326323	T	C	0.075	-0.109	0.015	3E-13	52.969	T	C	-0.151	0.197	0.440
HDAC3	QT interval	rs74446496	A	G	0.067	-0.151	0.016	4E-21	88.944	A	G	0.182	0.200	0.360
HDAC3	QT interval	rs7448461	A	G	0.051	0.119	0.018	6E-11	42.766	A	G	0.091	0.233	0.690
HDAC3	QT interval	rs74612838	G	C	0.102	0.147	0.013	1E-28	123.726	G	C	0.040	0.169	0.810
HDAC3	QT interval	rs74709227	G	T	0.137	-0.345	0.012	3E-168	764.689	G	T	0.099	0.158	0.530
HDAC3	QT interval	rs74968207	G	A	0.017	0.322	0.035	3E-20	84.721	G	A	-0.643	0.403	0.110
HDAC3	QT interval	rs75154750	T	C	0.015	0.468	0.040	2E-31	136.263	T	C	0.232	0.516	0.650
HDAC3	QT interval	rs756537	A	C	0.053	0.114	0.018	1E-10	41.101	A	C	-0.160	0.223	0.470

HDAC3	QT interval	rs75774668	A	G	0.020	-0.274	0.033	9E-17	69.189	A	G	0.402	0.412	0.330
HDAC3	QT interval	rs76814825	G	A	0.040	0.157	0.021	2E-13	54.055	G	A	-0.530	0.311	0.089
HDAC3	QT interval	rs76933395	C	T	0.020	-0.226	0.032	2E-12	49.090	C	T	0.004	0.340	0.990
HDAC3	QT interval	rs7702909	T	C	0.426	-0.082	0.009	7E-21	87.767	T	C	0.080	0.107	0.450
HDAC3	QT interval	rs7705576	C	G	0.235	-0.105	0.009	5E-29	124.904	C	G	-0.032	0.119	0.790
HDAC3	QT interval	rs7722894	T	C	0.051	-0.243	0.018	1E-41	182.753	T	C	0.593	0.214	0.006
HDAC3	QT interval	rs77241684	G	A	0.031	0.183	0.024	9E-15	60.091	G	A	0.023	0.273	0.930
HDAC3	QT interval	rs77300336	T	C	0.013	0.340	0.051	3E-11	44.230	T	C	-0.572	0.740	0.440
HDAC3	QT interval	rs78547804	T	C	0.110	0.080	0.013	6E-10	38.190	T	C	0.177	0.176	0.310
HDAC3	QT interval	rs78950299	T	C	0.083	-0.201	0.016	2E-37	163.984	T	C	0.404	0.212	0.056
HDAC3	QT interval	rs79970685	G	T	0.069	0.241	0.018	7E-43	188.376	G	T	-0.172	0.214	0.420
HDAC3	QT interval	rs80178004	A	G	0.024	0.214	0.026	6E-16	65.435	A	G	0.839	0.346	0.015
HDAC3	QT interval	rs80318397	A	G	0.073	-0.097	0.015	4E-10	39.365	A	G	0.305	0.203	0.130
HDAC3	QT interval	rs891991	A	T	0.213	0.187	0.010	2E-83	374.276	A	T	0.126	0.126	0.320
HDAC4	Serum phosphate levels	rs10172440	C	T	0.350	-0.274	0.012	5E-109	492.092	C	T	0.000	0.002	0.920
HDAC4	Serum phosphate levels	rs10192044	T	C	0.113	0.162	0.013	5E-38	166.316	T	C	0.006	0.003	0.054
HDAC4	Serum phosphate levels	rs10462023	A	G	0.351	-0.278	0.013	3E-105	474.524	A	G	0.003	0.002	0.260
HDAC4	Serum phosphate levels	rs112673616	G	A	0.082	0.347	0.020	2E-69	310.007	G	A	-0.007	0.004	0.063
HDAC4	Serum phosphate levels	rs113627235	C	T	0.020	0.618	0.049	5E-36	156.931	C	T	0.000	0.006	0.970
HDAC4	Serum phosphate levels	rs114069421	T	C	0.034	0.218	0.038	6E-09	33.903	T	C	-0.006	0.005	0.380
HDAC4	Serum phosphate levels	rs114604346	A	G	0.017	-0.484	0.067	4E-13	52.644	A	G	0.002	0.008	0.810

HDAC4	Serum phosphate levels	rs114715181	T	C	0.022	0.219	0.032	4E-12	48.102	T	C	0.011	0.007	0.099
HDAC4	Serum phosphate levels	rs115227879	A	G	0.035	0.462	0.038	2E-33	144.995	A	G	0.002	0.006	0.610
HDAC4	Serum phosphate levels	rs115452859	T	C	0.027	0.430	0.043	2E-23	99.892	T	C	0.001	0.006	0.940
HDAC4	Serum phosphate levels	rs115522175	A	G	0.019	0.445	0.059	6E-14	56.319	A	G	0.002	0.007	0.640
HDAC4	Serum phosphate levels	rs115661111	A	G	0.028	0.156	0.025	4E-10	39.132	A	G	0.003	0.006	0.870
HDAC4	Serum phosphate levels	rs116078872	T	G	0.045	0.112	0.020	2E-08	31.749	T	G	-0.005	0.004	0.420
HDAC4	Serum phosphate levels	rs116298301	T	C	0.029	-0.218	0.034	9E-11	42.099	T	C	-0.003	0.007	0.860
HDAC4	Serum phosphate levels	rs11688385	C	T	0.101	0.098	0.014	5E-13	52.175	C	T	-0.004	0.003	0.260
HDAC4	Serum phosphate levels	rs11693209	C	T	0.032	0.210	0.024	2E-18	77.025	C	T	0.007	0.006	0.250
HDAC4	Serum phosphate levels	rs1198823	G	A	0.324	0.159	0.013	1E-36	159.789	G	A	-0.001	0.002	0.690
HDAC4	Serum phosphate levels	rs12615308	A	G	0.131	-0.210	0.017	3E-33	144.504	A	G	-0.003	0.003	0.360
HDAC4	Serum phosphate levels	rs12618020	C	A	0.160	0.112	0.011	2E-22	95.038	C	A	0.003	0.003	0.320
HDAC4	Serum phosphate levels	rs12692216	C	T	0.348	-0.334	0.009	1E-200	1277.551	C	T	0.000	0.002	0.720
HDAC4	Serum phosphate levels	rs13013945	A	C	0.463	0.112	0.012	1E-20	86.985	A	C	-0.001	0.002	0.670
HDAC4	Serum phosphate levels	rs137993186	C	T	0.023	0.463	0.082	2E-08	31.783	C	T	-0.011	0.012	0.390
HDAC4	Serum phosphate levels	rs146086850	G	A	0.015	0.463	0.065	1E-12	50.129	G	A	-0.002	0.010	0.960
HDAC4	Serum phosphate levels	rs2278739	G	A	0.126	0.121	0.013	8E-22	92.164	G	A	-0.004	0.003	0.080
HDAC4	Serum phosphate levels	rs28668986	T	C	0.016	-0.244	0.038	1E-10	41.214	T	C	-0.002	0.008	0.860
HDAC4	Serum phosphate levels	rs34743088	T	C	0.050	-0.220	0.019	8E-30	128.601	T	C	-0.004	0.005	0.260
HDAC4	Serum phosphate levels	rs35752423	T	C	0.208	-0.165	0.016	1E-24	104.747	T	C	0.001	0.003	0.460
HDAC4	Serum phosphate levels	rs3754705	C	G	0.241	0.118	0.010	3E-34	148.675	C	G	-0.002	0.003	0.480
HDAC4	Serum phosphate levels	rs3769100	G	A	0.231	-0.250	0.014	3E-72	322.995	G	A	-0.004	0.003	0.110

HDAC4	Serum phosphate levels	rs3820808	G	C	0.047	0.167	0.029	1E-08	32.467	G	C	-0.002	0.005	0.480
HDAC4	Serum phosphate levels	rs3934971	G	T	0.422	0.047	0.008	1E-08	32.682	G	T	0.002	0.002	0.420
HDAC4	Serum phosphate levels	rs4349294	C	T	0.021	0.223	0.038	3E-09	35.175	C	T	0.002	0.010	0.780
HDAC4	Serum phosphate levels	rs55912241	G	A	0.017	0.465	0.057	2E-16	67.506	G	A	-0.012	0.007	0.110
HDAC4	Serum phosphate levels	rs62194932	A	G	0.109	-0.239	0.018	9E-40	174.114	A	G	-0.003	0.003	0.190
HDAC4	Serum phosphate levels	rs62194948	C	G	0.244	0.200	0.013	3E-56	249.855	C	G	-0.003	0.002	0.140
HDAC4	Serum phosphate levels	rs62196722	A	G	0.036	0.247	0.031	2E-15	63.409	A	G	0.000	0.006	0.950
HDAC4	Serum phosphate levels	rs6431555	T	C	0.021	0.356	0.041	3E-18	75.896	T	C	-0.007	0.007	0.310
HDAC4	Serum phosphate levels	rs72988350	T	C	0.098	-0.079	0.013	5E-09	34.129	T	C	-0.001	0.004	0.840
HDAC4	Serum phosphate levels	rs74922901	T	C	0.038	-0.265	0.031	9E-18	73.699	T	C	-0.009	0.005	0.086
HDAC4	Serum phosphate levels	rs75960565	G	A	0.025	0.222	0.028	1E-15	64.391	G	A	-0.012	0.007	0.063
HDAC4	Serum phosphate levels	rs7601275	A	G	0.347	0.064	0.009	5E-12	47.853	A	G	-0.005	0.002	0.018
HDAC4	Serum phosphate levels	rs76188121	A	C	0.023	0.292	0.039	3E-14	57.568	A	C	0.008	0.007	0.310
HDAC4	Serum phosphate levels	rs76664545	A	G	0.049	-0.200	0.023	2E-18	76.426	A	G	-0.008	0.005	0.130
HDAC4	Serum phosphate levels	rs76758152	T	C	0.022	0.528	0.074	1E-12	50.917	T	C	0.006	0.008	0.460
HDAC4	Serum phosphate levels	rs77070298	A	G	0.022	0.360	0.041	3E-18	76.168	A	G	0.009	0.007	0.130
HDAC4	Serum phosphate levels	rs77169642	A	G	0.055	-0.203	0.018	9E-31	133.019	A	G	0.000	0.005	0.890
HDAC4	Serum phosphate levels	rs78632873	A	G	0.016	0.583	0.081	6E-13	51.800	A	G	-0.005	0.009	0.770
HDAC4	Serum phosphate levels	rs78856938	G	A	0.013	0.435	0.069	4E-10	39.373	G	A	-0.009	0.011	0.450
HDAC4	Serum phosphate levels	rs79039501	A	G	0.027	-0.252	0.036	1E-12	50.367	A	G	-0.001	0.006	0.960
HDAC4	Serum phosphate levels	rs79110832	G	T	0.045	0.128	0.021	6E-10	38.279	G	T	0.004	0.005	0.430
HDAC4	Serum phosphate levels	rs80029919	C	T	0.022	0.415	0.047	1E-18	77.384	C	T	0.014	0.006	0.013
HDAC4	Serum phosphate levels	rs80167884	T	C	0.030	0.361	0.041	3E-18	75.833	T	C	-0.003	0.005	0.540

HDAC4	Serum phosphate levels	rs821806	G	A	0.347	0.180	0.008	2E-104	470.681	G	A	-0.005	0.002	0.034
HDAC4	Serum phosphate levels	rs934945	T	C	0.187	0.190	0.010	1E-77	348.055	T	C	-0.002	0.003	0.590
HDAC4	Serum phosphate levels	rs935411	T	C	0.107	-0.200	0.014	9E-46	201.588	T	C	-0.002	0.004	0.660
HDAC4	QT interval	rs10172440	C	T	0.350	-0.274	0.012	5E-109	492.092	C	T	0.176	0.120	0.140
HDAC4	QT interval	rs10181746	T	C	0.320	0.158	0.014	1E-29	128.251	T	C	0.040	0.126	0.750
HDAC4	QT interval	rs10192044	T	C	0.113	0.162	0.013	5E-38	166.316	T	C	-0.089	0.159	0.580
HDAC4	QT interval	rs10208760	T	G	0.329	0.137	0.008	7E-59	261.806	T	G	-0.098	0.112	0.380
HDAC4	QT interval	rs1027928	A	G	0.208	-0.127	0.010	2E-38	167.687	A	G	0.097	0.129	0.450
HDAC4	QT interval	rs10462023	A	G	0.351	-0.278	0.013	3E-105	474.524	A	G	-0.197	0.113	0.082
HDAC4	QT interval	rs10929273	A	G	0.356	0.094	0.014	1E-11	46.023	A	G	0.099	0.118	0.400
HDAC4	QT interval	rs111243161	G	A	0.180	-0.532	0.043	8E-35	151.546	G	A	-0.192	0.137	0.160
HDAC4	QT interval	rs112084035	G	A	0.137	0.121	0.012	5E-25	106.759	G	A	-0.224	0.153	0.140
HDAC4	QT interval	rs112548587	T	C	0.047	-0.422	0.045	3E-21	89.340	T	C	-0.136	0.279	0.630
HDAC4	QT interval	rs112612485	T	C	0.042	0.332	0.030	3E-29	125.992	T	C	0.382	0.312	0.220
HDAC4	QT interval	rs112673616	G	A	0.082	0.347	0.020	2E-69	310.007	G	A	0.135	0.199	0.500
HDAC4	QT interval	rs113627235	C	T	0.020	0.618	0.049	5E-36	156.931	C	T	-0.394	0.383	0.300
HDAC4	QT interval	rs113656277	A	G	0.016	0.251	0.038	6E-11	42.782	A	G	0.120	0.458	0.790
HDAC4	QT interval	rs114069421	T	C	0.034	0.218	0.038	6E-09	33.903	T	C	0.281	0.323	0.380
HDAC4	QT interval	rs114390286	T	C	0.043	0.198	0.024	4E-16	66.491	T	C	0.393	0.296	0.180
HDAC4	QT interval	rs114436503	A	G	0.018	0.414	0.051	4E-16	66.064	A	G	-0.025	0.430	0.950
HDAC4	QT interval	rs114454255	G	C	0.033	-0.252	0.032	2E-15	62.714	G	C	0.094	0.297	0.750

HDAC4	QT interval	rs114489460	T	C	0.023	-0.195	0.028	1E-12	50.103	T	C	-0.384	0.383	0.320
HDAC4	QT interval	rs114534473	A	G	0.020	0.309	0.037	1E-16	68.628	A	G	-0.023	0.384	0.950
HDAC4	QT interval	rs114574280	T	C	0.014	0.378	0.063	2E-09	36.392	T	C	-0.176	0.671	0.790
HDAC4	QT interval	rs114604346	A	G	0.017	-0.484	0.067	4E-13	52.644	A	G	0.282	0.529	0.590
HDAC4	QT interval	rs114615836	G	A	0.014	0.721	0.113	2E-10	40.888	G	A	0.926	0.731	0.200
HDAC4	QT interval	rs114629133	A	G	0.019	0.233	0.033	3E-12	48.785	A	G	-0.480	0.461	0.300
HDAC4	QT interval	rs114715181	T	C	0.022	0.219	0.032	4E-12	48.102	T	C	0.151	0.474	0.750
HDAC4	QT interval	rs114794746	A	G	0.054	0.250	0.046	4E-08	30.118	A	G	0.002	0.276	0.990
HDAC4	QT interval	rs114862906	T	C	0.016	0.238	0.037	7E-11	42.489	T	C	-0.480	0.436	0.270
HDAC4	QT interval	rs114870822	A	G	0.019	0.191	0.031	4E-10	38.998	A	G	-0.018	0.444	0.970
HDAC4	QT interval	rs114995139	T	C	0.036	0.168	0.022	3E-14	57.988	T	C	-0.131	0.293	0.650
HDAC4	QT interval	rs115135110	T	C	0.044	0.431	0.052	9E-17	69.192	T	C	0.448	0.240	0.062
HDAC4	QT interval	rs1151795	G	A	0.027	0.447	0.043	9E-26	110.124	G	A	0.527	0.366	0.150
HDAC4	QT interval	rs115227879	A	G	0.035	0.462	0.038	2E-33	144.995	A	G	0.058	0.373	0.880
HDAC4	QT interval	rs115403835	G	A	0.031	-0.233	0.031	9E-14	55.631	G	A	-0.927	0.396	0.019
HDAC4	QT interval	rs115452859	T	C	0.027	0.430	0.043	2E-23	99.892	T	C	-0.780	0.440	0.076
HDAC4	QT interval	rs115522175	A	G	0.019	0.445	0.059	6E-14	56.319	A	G	0.206	0.405	0.610
HDAC4	QT interval	rs115661111	A	G	0.028	0.156	0.025	4E-10	39.132	A	G	-0.363	0.326	0.270
HDAC4	QT interval	rs116078872	T	G	0.045	0.112	0.020	2E-08	31.749	T	G	-0.028	0.230	0.900
HDAC4	QT interval	rs116152447	A	G	0.021	0.220	0.032	3E-12	48.478	A	G	-0.294	0.387	0.450
HDAC4	QT interval	rs116298301	T	C	0.029	-0.218	0.034	9E-11	42.099	T	C	-0.912	0.353	0.010
HDAC4	QT interval	rs11673847	A	G	0.220	0.066	0.010	9E-12	46.466	A	G	0.013	0.129	0.920
HDAC4	QT interval	rs11675917	G	C	0.062	-0.175	0.018	8E-23	96.759	G	C	-0.035	0.207	0.870

HDAC4	QT interval	rs11679732	A	G	0.242	0.442	0.013	1E-200	1078.114	A	G	0.108	0.122	0.380
HDAC4	QT interval	rs11680171	G	A	0.357	0.094	0.012	4E-14	57.184	G	A	0.005	0.137	0.970
HDAC4	QT interval	rs11686911	A	G	0.107	-0.231	0.014	1E-65	292.970	A	G	-0.103	0.183	0.570
HDAC4	QT interval	rs11688385	C	T	0.101	0.098	0.014	5E-13	52.175	C	T	-0.170	0.174	0.330
HDAC4	QT interval	rs11688820	A	G	0.024	0.190	0.029	3E-11	44.350	A	G	-0.759	0.424	0.073
HDAC4	QT interval	rs11693209	C	T	0.032	0.210	0.024	2E-18	77.025	C	T	-0.639	0.334	0.056
HDAC4	QT interval	rs11884933	A	G	0.438	-0.105	0.009	1E-34	150.760	A	G	0.105	0.111	0.340
HDAC4	QT interval	rs11888775	G	A	0.174	0.149	0.014	2E-25	108.672	G	A	0.089	0.136	0.510
HDAC4	QT interval	rs11894086	T	C	0.354	-0.183	0.012	3E-49	217.403	T	C	0.027	0.105	0.800
HDAC4	QT interval	rs11894535	T	C	0.208	0.283	0.013	2E-101	457.021	T	C	0.054	0.128	0.670
HDAC4	QT interval	rs1198814	C	T	0.161	0.227	0.016	9E-46	201.669	C	T	0.098	0.136	0.470
HDAC4	QT interval	rs1198819	T	G	0.487	0.078	0.008	2E-22	95.234	T	G	-0.017	0.102	0.860
HDAC4	QT interval	rs1198821	C	T	0.202	0.104	0.010	1E-24	104.613	C	T	0.045	0.129	0.730
HDAC4	QT interval	rs1198823	G	A	0.324	0.159	0.013	1E-36	159.789	G	A	0.037	0.107	0.730
HDAC4	QT interval	rs12053552	G	A	0.091	0.131	0.015	2E-19	80.882	G	A	-0.416	0.195	0.033
HDAC4	QT interval	rs1210881	G	T	0.486	0.129	0.008	2E-59	264.533	G	T	0.224	0.111	0.045
HDAC4	QT interval	rs12474237	C	T	0.097	-0.230	0.036	2E-10	40.361	C	T	-0.082	0.182	0.650
HDAC4	QT interval	rs12474573	C	T	0.176	-0.058	0.010	3E-08	30.876	C	T	-0.132	0.137	0.340
HDAC4	QT interval	rs12611445	C	T	0.502	-0.101	0.009	5E-29	125.109	C	T	-0.043	0.126	0.730
HDAC4	QT interval	rs12613388	T	G	0.074	0.176	0.023	6E-15	60.921	T	G	-0.120	0.188	0.520
HDAC4	QT interval	rs12614428	G	A	0.175	-0.123	0.016	3E-15	62.319	G	A	-0.006	0.132	0.960

HDAC4	QT interval	rs12615308	A	G	0.131	-0.210	0.017	3E-33	144.504	A	G	0.032	0.145	0.820
HDAC4	QT interval	rs12616232	T	C	0.219	0.343	0.023	4E-52	230.968	T	C	-0.003	0.121	0.980
HDAC4	QT interval	rs12618020	C	A	0.160	0.112	0.011	2E-22	95.038	C	A	0.158	0.160	0.320
HDAC4	QT interval	rs12618510	A	G	0.395	-0.313	0.012	6E-151	684.964	A	G	-0.253	0.112	0.024
HDAC4	QT interval	rs12692216	C	T	0.348	-0.334	0.009	1E-200	1277.551	C	T	-0.247	0.124	0.047
HDAC4	QT interval	rs12692217	G	A	0.019	-0.422	0.076	3E-08	30.610	G	A	-1.600	0.509	0.002
HDAC4	QT interval	rs12692237	T	C	0.173	-0.066	0.012	1E-08	32.613	T	C	-0.043	0.131	0.740
HDAC4	QT interval	rs12992035	A	T	0.204	0.269	0.015	1E-76	342.940	A	T	0.278	0.132	0.035
HDAC4	QT interval	rs12999111	T	C	0.205	0.123	0.010	5E-35	152.376	T	C	0.163	0.137	0.230
HDAC4	QT interval	rs13010511	G	C	0.268	-0.072	0.009	3E-15	62.489	G	C	0.024	0.117	0.840
HDAC4	QT interval	rs13013232	C	T	0.371	-0.152	0.008	3E-73	327.697	C	T	0.148	0.113	0.190
HDAC4	QT interval	rs13013945	A	C	0.463	0.112	0.012	1E-20	86.985	A	C	-0.112	0.118	0.340
HDAC4	QT interval	rs13020667	A	G	0.315	0.090	0.009	6E-22	92.598	A	G	-0.167	0.123	0.170
HDAC4	QT interval	rs13035956	T	C	0.153	-0.112	0.011	1E-23	100.365	T	C	-0.005	0.138	0.970
HDAC4	QT interval	rs13392396	C	T	0.079	-0.166	0.021	5E-16	65.727	C	T	0.000	0.175	1.000
HDAC4	QT interval	rs137993186	C	T	0.023	0.463	0.082	2E-08	31.783	C	T	-0.319	0.867	0.710
HDAC4	QT interval	rs140857117	T	C	0.016	-0.992	0.128	1E-14	59.988	T	C	-1.610	1.190	0.170
HDAC4	QT interval	rs141069794	G	A	0.067	0.364	0.028	1E-37	164.630	G	A	-0.078	0.225	0.730
HDAC4	QT interval	rs141070815	A	G	0.046	-0.319	0.032	7E-23	96.897	A	G	0.234	0.253	0.360
HDAC4	QT interval	rs141583457	A	G	0.014	-0.251	0.044	1E-08	32.078	A	G	-0.367	0.568	0.520
HDAC4	QT interval	rs142777664	A	G	0.017	-0.320	0.038	4E-17	70.926	A	G	-1.180	0.559	0.035

HDAC4	QT interval	rs145140451	G	A	0.013	0.459	0.074	5E-10	38.708	G	A	-0.374	0.643	0.560
HDAC4	QT interval	rs145224375	A	G	0.035	0.158	0.023	4E-12	48.302	A	G	-0.277	0.315	0.380
HDAC4	QT interval	rs145390497	A	G	0.017	-0.425	0.041	8E-25	105.773	A	G	-0.740	0.768	0.340
HDAC4	QT interval	rs145470986	G	A	0.016	0.760	0.097	5E-15	61.152	G	A	-0.404	0.659	0.540
HDAC4	QT interval	rs145748353	T	C	0.017	-0.271	0.046	3E-09	35.035	T	C	0.843	0.407	0.038
HDAC4	QT interval	rs145852943	A	G	0.018	0.374	0.034	1E-28	123.281	A	G	0.400	0.481	0.410
HDAC4	QT interval	rs146086850	G	A	0.015	0.463	0.065	1E-12	50.129	G	A	-0.012	0.652	0.980
HDAC4	QT interval	rs146120064	A	G	0.017	0.224	0.035	3E-10	39.836	A	G	0.051	0.453	0.910
HDAC4	QT interval	rs148542110	G	A	0.019	-0.318	0.054	5E-09	34.029	G	A	-1.370	1.920	0.480
HDAC4	QT interval	rs148953838	C	T	0.016	0.388	0.055	2E-12	49.598	C	T	0.394	0.419	0.350
HDAC4	QT interval	rs150838863	G	T	0.014	0.871	0.095	5E-20	83.909	G	T	-0.036	0.598	0.950
HDAC4	QT interval	rs1562339	C	A	0.442	-0.340	0.012	4E-188	856.006	C	A	0.125	0.108	0.250
HDAC4	QT interval	rs1665283	A	G	0.099	-0.236	0.018	1E-38	169.473	A	G	0.188	0.192	0.330
HDAC4	QT interval	rs180793087	A	G	0.017	-0.217	0.035	7E-10	37.985	A	G	0.542	0.408	0.180
HDAC4	QT interval	rs185110866	A	C	0.014	-0.610	0.100	1E-09	37.317	A	C	-0.822	0.547	0.130
HDAC4	QT interval	rs186249944	C	A	0.035	-0.145	0.024	9E-10	37.476	C	A	-0.083	0.309	0.790
HDAC4	QT interval	rs186695350	T	C	0.016	0.600	0.083	5E-13	52.334	T	C	0.206	0.475	0.660
HDAC4	QT interval	rs1867933	A	G	0.347	-0.210	0.013	7E-60	266.519	A	G	-0.087	0.109	0.430
HDAC4	QT interval	rs1867934	G	A	0.127	0.146	0.012	3E-34	148.654	G	A	-0.024	0.162	0.880
HDAC4	QT interval	rs188589030	C	T	0.013	0.865	0.145	3E-09	35.376	C	T	-0.110	0.735	0.880
HDAC4	QT interval	rs191717602	G	T	0.012	-0.821	0.145	2E-08	31.925	G	T	-0.409	0.780	0.600
HDAC4	QT interval	rs1982339	T	C	0.179	0.166	0.016	1E-26	113.862	T	C	-0.067	0.147	0.650

HDAC4	QT interval	rs2119022	T	C	0.020	0.629	0.046	7E-42	183.738	T	C	0.699	0.509	0.170
HDAC4	QT interval	rs2264132	T	C	0.206	-0.313	0.014	9E-105	472.546	T	C	0.049	0.123	0.690
HDAC4	QT interval	rs2278739	G	A	0.126	0.121	0.013	8E-22	92.164	G	A	0.046	0.167	0.780
HDAC4	QT interval	rs28497373	G	A	0.067	-0.091	0.016	2E-08	31.128	G	A	0.004	0.192	0.980
HDAC4	QT interval	rs28668986	T	C	0.016	-0.244	0.038	1E-10	41.214	T	C	0.042	0.482	0.930
HDAC4	QT interval	rs303039	A	C	0.265	0.091	0.009	2E-23	99.684	A	C	-0.028	0.120	0.820
HDAC4	QT interval	rs34590157	A	G	0.053	0.112	0.019	4E-09	34.836	A	G	-0.183	0.256	0.480
HDAC4	QT interval	rs34743088	T	C	0.050	-0.220	0.019	8E-30	128.601	T	C	-0.185	0.281	0.510
HDAC4	QT interval	rs35047785	G	A	0.067	-0.185	0.028	3E-11	44.225	G	A	0.043	0.231	0.850
HDAC4	QT interval	rs35277801	T	G	0.373	-0.269	0.012	1E-107	485.481	T	G	0.039	0.111	0.730
HDAC4	QT interval	rs35578309	A	G	0.143	-0.171	0.017	3E-24	103.197	A	G	0.170	0.160	0.290
HDAC4	QT interval	rs35752423	T	C	0.208	-0.165	0.016	1E-24	104.747	T	C	0.086	0.143	0.550
HDAC4	QT interval	rs3739064	G	A	0.207	0.453	0.013	1E-200	1243.733	G	A	0.157	0.130	0.230
HDAC4	QT interval	rs3754705	C	G	0.241	0.118	0.010	3E-34	148.675	C	G	0.339	0.131	0.010
HDAC4	QT interval	rs3769100	G	A	0.231	-0.250	0.014	3E-72	322.995	G	A	-0.006	0.121	0.960
HDAC4	QT interval	rs3820808	G	C	0.047	0.167	0.029	1E-08	32.467	G	C	0.208	0.246	0.400
HDAC4	QT interval	rs3891424	A	G	0.046	-0.181	0.020	6E-20	83.481	A	G	0.251	0.253	0.320
HDAC4	QT interval	rs3934971	G	T	0.422	0.047	0.008	1E-08	32.682	G	T	0.050	0.115	0.660
HDAC4	QT interval	rs4349294	C	T	0.021	0.223	0.038	3E-09	35.175	C	T	-0.126	0.538	0.820
HDAC4	QT interval	rs4663298	A	G	0.096	0.290	0.018	1E-56	251.438	A	G	-0.117	0.182	0.520
HDAC4	QT interval	rs4663805	A	C	0.137	-0.096	0.012	1E-16	68.347	A	C	-0.059	0.154	0.700

HDAC4	QT interval	rs4663874	G	C	0.321	-0.170	0.011	6E-50	220.848	G	C	0.099	0.111	0.370
HDAC4	QT interval	rs4663908	G	A	0.145	0.064	0.011	2E-08	31.414	G	A	0.251	0.151	0.097
HDAC4	QT interval	rs55694679	T	C	0.060	0.192	0.017	3E-29	125.844	T	C	0.196	0.229	0.390
HDAC4	QT interval	rs55775132	A	G	0.045	0.194	0.020	1E-22	96.099	A	G	0.368	0.271	0.170
HDAC4	QT interval	rs55869445	A	G	0.134	0.122	0.012	6E-25	106.553	A	G	-0.148	0.147	0.310
HDAC4	QT interval	rs55912241	G	A	0.017	0.465	0.057	2E-16	67.506	G	A	0.410	0.478	0.390
HDAC4	QT interval	rs559686	A	G	0.239	-0.150	0.013	1E-32	142.044	A	G	0.103	0.118	0.390
HDAC4	QT interval	rs56001807	C	T	0.173	-0.200	0.016	1E-37	164.696	C	T	-0.234	0.137	0.087
HDAC4	QT interval	rs56011431	C	T	0.021	-0.293	0.050	5E-09	34.279	C	T	-0.306	0.372	0.410
HDAC4	QT interval	rs56067485	T	C	0.071	0.274	0.016	1E-69	311.235	T	C	0.020	0.203	0.920
HDAC4	QT interval	rs56100702	T	G	0.239	-0.118	0.016	3E-13	53.002	T	G	-0.142	0.124	0.250
HDAC4	QT interval	rs56201071	A	G	0.016	0.799	0.086	1E-20	86.870	A	G	0.471	0.563	0.400
HDAC4	QT interval	rs56266341	C	T	0.159	0.103	0.013	1E-15	64.093	C	T	-0.043	0.156	0.780
HDAC4	QT interval	rs56408852	C	G	0.350	0.127	0.008	8E-53	233.883	C	G	0.096	0.111	0.390
HDAC4	QT interval	rs57328854	G	A	0.191	0.114	0.010	5E-29	125.193	G	A	-0.079	0.135	0.560
HDAC4	QT interval	rs57455455	G	A	0.103	-0.135	0.018	4E-14	57.117	G	A	-0.114	0.166	0.490
HDAC4	QT interval	rs60634320	T	G	0.066	-0.199	0.016	1E-33	146.464	T	G	0.031	0.206	0.880
HDAC4	QT interval	rs62189869	A	G	0.205	0.075	0.010	3E-14	57.894	A	G	-0.262	0.126	0.038
HDAC4	QT interval	rs62189875	G	A	0.093	0.140	0.014	1E-23	100.898	G	A	0.211	0.186	0.260
HDAC4	QT interval	rs62194461	A	G	0.046	-0.241	0.021	2E-31	135.942	A	G	0.217	0.242	0.370
HDAC4	QT interval	rs62194469	G	T	0.108	-0.332	0.021	5E-54	239.562	G	T	0.139	0.161	0.390
HDAC4	QT interval	rs62194471	A	G	0.390	0.440	0.012	1E-200	1414.491	A	G	-0.068	0.118	0.570

HDAC4	QT interval	rs62194932	A	G	0.109	-0.239	0.018	9E-40	174.114	A	G	-0.074	0.179	0.680
HDAC4	QT interval	rs62194936	C	T	0.065	0.121	0.017	4E-12	48.080	C	T	-0.115	0.214	0.590
HDAC4	QT interval	rs62194948	C	G	0.244	0.200	0.013	3E-56	249.855	C	G	-0.027	0.127	0.830
HDAC4	QT interval	rs62196722	A	G	0.036	0.247	0.031	2E-15	63.409	A	G	0.405	0.311	0.190
HDAC4	QT interval	rs6431555	T	C	0.021	0.356	0.041	3E-18	75.896	T	C	0.529	0.379	0.160
HDAC4	QT interval	rs6431582	C	T	0.366	0.146	0.014	3E-24	103.392	C	T	-0.264	0.132	0.046
HDAC4	QT interval	rs6431599	C	A	0.272	0.127	0.022	8E-09	33.272	C	A	0.142	0.121	0.240
HDAC4	QT interval	rs6719491	A	G	0.167	-0.141	0.011	8E-40	174.325	A	G	0.162	0.137	0.240
HDAC4	QT interval	rs6721411	A	G	0.048	-0.293	0.028	2E-26	113.073	A	G	-0.180	0.270	0.500
HDAC4	QT interval	rs6723015	T	C	0.045	0.189	0.032	3E-09	35.405	T	C	-0.074	0.288	0.800
HDAC4	QT interval	rs6727736	C	T	0.147	-0.116	0.011	2E-24	104.403	C	T	0.133	0.145	0.360
HDAC4	QT interval	rs6728338	A	G	0.053	-0.182	0.027	7E-12	46.909	A	G	-0.187	0.197	0.340
HDAC4	QT interval	rs6740741	C	T	0.498	0.203	0.009	9E-125	564.462	C	T	0.121	0.112	0.280
HDAC4	QT interval	rs6741923	C	G	0.287	0.224	0.009	3E-124	562.088	C	G	0.205	0.113	0.070
HDAC4	QT interval	rs6746771	G	A	0.111	0.101	0.013	9E-16	64.574	G	A	0.166	0.176	0.350
HDAC4	QT interval	rs6753456	G	A	0.350	-0.095	0.013	4E-13	52.862	G	A	0.119	0.113	0.290
HDAC4	QT interval	rs6758509	A	G	0.118	0.263	0.018	2E-47	209.646	A	G	-0.160	0.154	0.300
HDAC4	QT interval	rs71425002	C	T	0.027	-0.479	0.066	5E-13	52.235	C	T	-0.692	0.481	0.150
HDAC4	QT interval	rs71426536	T	C	0.018	-0.440	0.079	2E-08	31.445	T	C	0.245	0.587	0.680
HDAC4	QT interval	rs72978050	G	A	0.057	0.244	0.017	2E-44	195.458	G	A	0.061	0.243	0.800
HDAC4	QT interval	rs72979292	A	G	0.045	0.409	0.033	7E-35	151.797	A	G	0.214	0.275	0.440

HDAC4	QT interval	rs72981964	G	A	0.033	-0.147	0.023	3E-10	39.950	G	A	0.273	0.300	0.360
HDAC4	QT interval	rs72988350	T	C	0.098	-0.079	0.013	5E-09	34.129	T	C	-0.130	0.178	0.470
HDAC4	QT interval	rs72989053	T	C	0.041	0.143	0.022	6E-11	42.986	T	C	0.156	0.311	0.620
HDAC4	QT interval	rs72996319	T	A	0.260	0.071	0.009	6E-15	61.034	T	A	-0.185	0.118	0.120
HDAC4	QT interval	rs72998394	G	T	0.412	-0.050	0.008	9E-10	37.425	G	T	-0.072	0.103	0.490
HDAC4	QT interval	rs73091785	C	T	0.169	0.098	0.016	5E-10	38.820	C	T	-0.030	0.131	0.820
HDAC4	QT interval	rs73098386	T	C	0.035	0.311	0.037	1E-16	68.628	T	C	-0.538	0.297	0.070
HDAC4	QT interval	rs7340253	G	A	0.424	0.101	0.011	4E-20	84.644	G	A	-0.074	0.102	0.470
HDAC4	QT interval	rs7355613	A	G	0.213	0.116	0.016	1E-12	50.891	A	G	0.021	0.146	0.880
HDAC4	QT interval	rs744837	C	T	0.035	-0.152	0.025	2E-09	35.856	C	T	-0.170	0.314	0.590
HDAC4	QT interval	rs74513214	A	G	0.313	0.188	0.013	1E-49	219.883	A	G	0.040	0.114	0.730
HDAC4	QT interval	rs74922901	T	C	0.038	-0.265	0.031	9E-18	73.699	T	C	-0.057	0.307	0.850
HDAC4	QT interval	rs75537083	G	T	0.015	-0.253	0.042	2E-09	35.737	G	T	-0.416	0.758	0.580
HDAC4	QT interval	rs75563491	G	A	0.014	0.592	0.070	4E-17	70.880	G	A	0.178	0.462	0.700
HDAC4	QT interval	rs7560313	C	G	0.506	-0.140	0.008	9E-69	307.287	C	G	0.125	0.103	0.230
HDAC4	QT interval	rs7562977	G	A	0.292	0.115	0.009	7E-39	169.990	G	A	0.073	0.114	0.520
HDAC4	QT interval	rs7563369	C	T	0.329	0.338	0.011	1E-200	917.592	C	T	-0.078	0.115	0.500
HDAC4	QT interval	rs7567226	A	C	0.260	-0.190	0.013	1E-45	201.533	A	C	0.130	0.122	0.290
HDAC4	QT interval	rs75782737	T	G	0.105	0.090	0.013	4E-12	47.903	T	G	-0.015	0.167	0.930
HDAC4	QT interval	rs7579382	T	C	0.400	-0.089	0.011	7E-16	65.277	T	C	-0.038	0.104	0.720
HDAC4	QT interval	rs7582777	A	G	0.175	-0.147	0.011	7E-44	192.948	A	G	0.052	0.140	0.710
HDAC4	QT interval	rs75845245	T	C	0.033	0.201	0.024	9E-17	69.103	T	C	0.103	0.290	0.720

HDAC4	QT interval	rs7590387	C	G	0.490	0.131	0.012	6E-28	120.192	C	G	-0.011	0.107	0.920
HDAC4	QT interval	rs75960565	G	A	0.025	0.222	0.028	1E-15	64.391	G	A	0.128	0.383	0.740
HDAC4	QT interval	rs7597345	T	C	0.248	0.152	0.009	3E-61	272.574	T	C	-0.092	0.122	0.450
HDAC4	QT interval	rs7601275	A	G	0.347	0.064	0.009	5E-12	47.853	A	G	-0.012	0.122	0.920
HDAC4	QT interval	rs76188121	A	C	0.023	0.292	0.039	3E-14	57.568	A	C	-0.358	0.426	0.400
HDAC4	QT interval	rs76554193	C	T	0.050	-0.300	0.049	9E-10	37.472	C	T	-0.549	0.258	0.034
HDAC4	QT interval	rs76664545	A	G	0.049	-0.200	0.023	2E-18	76.426	A	G	-0.379	0.277	0.170
HDAC4	QT interval	rs76731390	T	C	0.027	0.202	0.026	9E-15	60.171	T	C	-0.326	0.331	0.320
HDAC4	QT interval	rs76758152	T	C	0.022	0.528	0.074	1E-12	50.917	T	C	0.377	0.525	0.470
HDAC4	QT interval	rs77070298	A	G	0.022	0.360	0.041	3E-18	76.168	A	G	-0.538	0.340	0.110
HDAC4	QT interval	rs77169642	A	G	0.055	-0.203	0.018	9E-31	133.019	A	G	-0.155	0.238	0.520
HDAC4	QT interval	rs77504293	G	A	0.152	0.135	0.011	2E-33	145.656	G	A	0.175	0.153	0.250
HDAC4	QT interval	rs77815260	T	C	0.035	0.168	0.023	5E-13	52.362	T	C	0.018	0.286	0.950
HDAC4	QT interval	rs77985050	T	C	0.022	-0.318	0.044	8E-13	51.240	T	C	0.401	0.420	0.340
HDAC4	QT interval	rs78076311	T	C	0.034	0.220	0.025	6E-19	79.231	T	C	-0.260	0.312	0.400
HDAC4	QT interval	rs78098900	A	G	0.034	-0.206	0.024	2E-17	72.471	A	G	0.254	0.331	0.440
HDAC4	QT interval	rs78179953	G	A	0.027	0.192	0.026	1E-13	54.988	G	A	-0.288	0.338	0.390
HDAC4	QT interval	rs78323382	A	G	0.021	-0.234	0.040	6E-09	33.991	A	G	-0.449	0.399	0.260
HDAC4	QT interval	rs78632873	A	G	0.016	0.583	0.081	6E-13	51.800	A	G	0.158	0.640	0.800
HDAC4	QT interval	rs78856938	G	A	0.013	0.435	0.069	4E-10	39.373	G	A	1.170	0.718	0.100
HDAC4	QT interval	rs78865534	C	T	0.123	0.184	0.030	8E-10	37.793	C	T	0.253	0.157	0.110
HDAC4	QT interval	rs78991032	G	C	0.125	-0.130	0.012	5E-26	111.325	G	C	0.260	0.156	0.095
HDAC4	QT interval	rs79039501	A	G	0.027	-0.252	0.036	1E-12	50.367	A	G	0.490	0.321	0.130

HDAC4	QT interval	rs79110832	G	T	0.045	0.128	0.021	6E-10	38.279	G	T	-0.043	0.279	0.880
HDAC4	QT interval	rs79676909	T	C	0.041	0.153	0.021	9E-14	55.497	T	C	-0.221	0.269	0.410
HDAC4	QT interval	rs79749461	G	C	0.035	0.262	0.032	3E-16	66.514	G	C	0.119	0.292	0.680
HDAC4	QT interval	rs79784164	G	A	0.143	0.212	0.011	1E-76	343.090	G	A	-0.106	0.155	0.500
HDAC4	QT interval	rs79996022	A	T	0.083	-0.232	0.021	2E-27	117.723	A	T	-0.017	0.179	0.930
HDAC4	QT interval	rs80029919	C	T	0.022	0.415	0.047	1E-18	77.384	C	T	-0.469	0.410	0.250
HDAC4	QT interval	rs80167884	T	C	0.030	0.361	0.041	3E-18	75.833	T	C	-0.142	0.330	0.670
HDAC4	QT interval	rs821806	G	A	0.347	0.180	0.008	2E-104	470.681	G	A	-0.161	0.108	0.130
HDAC4	QT interval	rs895572	C	T	0.459	-0.208	0.008	5E-154	698.928	C	T	0.079	0.105	0.450
HDAC4	QT interval	rs903373	T	C	0.249	0.204	0.014	4E-51	226.430	T	C	0.199	0.118	0.092
HDAC4	QT interval	rs934945	T	C	0.187	0.190	0.010	1E-77	348.055	T	C	0.101	0.138	0.470
HDAC4	QT interval	rs935411	T	C	0.107	-0.200	0.014	9E-46	201.588	T	C	-0.564	0.212	0.008
HDAC5	Serum phosphate levels	rs117166686	C	T	0.018	-0.568	0.094	2E-09	36.436	C	T	0.012	0.007	0.060
HDAC5	Serum phosphate levels	rs117199550	T	C	0.038	0.124	0.022	1E-08	32.413	T	C	0.016	0.005	0.001
HDAC5	Serum phosphate levels	rs117267254	A	G	0.037	0.339	0.022	3E-51	226.851	A	G	0.004	0.006	0.360
HDAC5	Serum phosphate levels	rs117379709	A	G	0.013	-0.334	0.046	6E-13	51.999	A	G	-0.021	0.009	0.016
HDAC5	Serum phosphate levels	rs117389699	T	C	0.027	0.502	0.068	1E-13	55.074	T	C	-0.007	0.008	0.590
HDAC5	Serum phosphate levels	rs117499775	C	T	0.036	-0.169	0.025	6E-12	47.420	C	T	-0.018	0.005	0.000
HDAC5	Serum phosphate levels	rs117615688	A	G	0.052	-0.274	0.021	3E-40	176.496	A	G	-0.029	0.004	0.000
HDAC5	Serum phosphate levels	rs117641629	A	G	0.020	-0.300	0.042	1E-12	50.132	A	G	0.022	0.007	0.001
HDAC5	Serum phosphate levels	rs118185397	T	C	0.060	0.366	0.061	2E-09	36.380	T	C	0.000	0.004	0.870

HDAC5	Serum phosphate levels	rs12950522	C	A	0.503	0.339	0.025	2E-43	191.435	C	A	0.012	0.002	0.000
HDAC5	QT interval	rs111288789	C	T	0.017	-1.400	0.108	2E-38	168.226	C	T	0.449	0.629	0.480
HDAC5	QT interval	rs111878933	A	C	0.205	-0.278	0.012	9E-117	527.666	G	A	-0.377	0.131	0.004
HDAC5	QT interval	rs113193302	T	C	0.093	-0.248	0.018	6E-44	193.217	T	C	-0.685	1.870	0.710
HDAC5	QT interval	rs113369483	A	G	0.017	-0.331	0.050	6E-11	42.905	A	G	0.306	0.425	0.470
HDAC5	QT interval	rs114469358	C	T	0.111	0.137	0.013	3E-25	107.503	C	T	-0.409	0.174	0.019
HDAC5	QT interval	rs116096269	T	A	0.023	0.253	0.031	4E-16	66.110	T	A	-0.808	0.349	0.020
HDAC5	QT interval	rs116843903	A	G	0.071	0.180	0.016	4E-29	125.618	A	G	-0.017	0.196	0.930
HDAC5	QT interval	rs116876049	G	A	0.020	-0.253	0.038	2E-11	45.516	G	A	-0.171	0.479	0.720
HDAC5	QT interval	rs116897693	A	G	0.018	0.266	0.042	2E-10	40.503	A	G	-0.717	0.508	0.160
HDAC5	QT interval	rs116987516	T	C	0.017	0.422	0.053	2E-15	63.269	T	C	0.768	0.649	0.240
HDAC5	QT interval	rs117053640	C	T	0.019	-0.287	0.037	6E-15	60.921	C	T	-0.271	0.421	0.520
HDAC5	QT interval	rs117100342	T	C	0.035	0.272	0.023	2E-32	140.689	T	C	0.345	0.311	0.270
HDAC5	QT interval	rs117149222	T	A	0.093	0.163	0.014	8E-32	137.869	T	A	-0.093	0.193	0.630
HDAC5	QT interval	rs117166686	C	T	0.018	-0.568	0.094	2E-09	36.436	C	T	0.013	0.332	0.970
HDAC5	QT interval	rs117199550	T	C	0.038	0.124	0.022	1E-08	32.413	T	C	0.160	0.267	0.550
HDAC5	QT interval	rs117224340	T	C	0.022	-0.321	0.037	2E-18	76.938	T	C	-0.104	0.402	0.790
HDAC5	QT interval	rs117267254	A	G	0.037	0.339	0.022	3E-51	226.851	A	G	-0.181	0.285	0.520
HDAC5	QT interval	rs117319569	T	C	0.053	0.337	0.056	2E-09	36.077	T	C	0.168	0.278	0.550
HDAC5	QT interval	rs117346071	A	C	0.072	0.110	0.016	3E-11	44.114	A	C	-0.014	0.194	0.940
HDAC5	QT interval	rs117372337	A	G	0.031	-0.286	0.033	7E-18	74.098	A	G	-0.352	0.307	0.250
HDAC5	QT interval	rs117379709	A	G	0.013	-0.334	0.046	6E-13	51.999	A	G	0.461	0.581	0.430

HDAC5	QT interval	rs117389699	T	C	0.027	0.502	0.068	1E-13	55.074	T	C	0.439	0.478	0.360
HDAC5	QT interval	rs117499775	C	T	0.036	-0.169	0.025	6E-12	47.420	C	T	-0.100	0.356	0.780
HDAC5	QT interval	rs117615688	A	G	0.052	-0.274	0.021	3E-40	176.496	A	G	0.415	0.209	0.047
HDAC5	QT interval	rs117641629	A	G	0.020	-0.300	0.042	1E-12	50.132	A	G	0.091	0.466	0.850
HDAC5	QT interval	rs118011309	T	G	0.056	-0.239	0.021	1E-30	132.210	T	G	-0.480	0.290	0.097
HDAC5	QT interval	rs118020479	T	C	0.029	0.413	0.065	3E-10	39.975	T	C	0.607	0.418	0.150
HDAC5	QT interval	rs118039425	A	G	0.034	-0.271	0.029	1E-20	86.508	A	G	0.068	0.342	0.840
HDAC5	QT interval	rs118077335	C	T	0.038	0.390	0.053	2E-13	53.723	C	T	-0.654	0.266	0.014
HDAC5	QT interval	rs118185397	T	C	0.060	0.366	0.061	2E-09	36.380	T	C	-0.077	0.213	0.720
HDAC5	QT interval	rs12940065	A	G	0.152	0.227	0.011	4E-93	419.171	A	G	-0.341	0.143	0.017
HDAC5	QT interval	rs12942300	A	T	0.148	0.397	0.034	7E-31	133.411	A	T	-0.236	0.147	0.110
HDAC5	QT interval	rs12950522	C	A	0.503	0.339	0.025	2E-43	191.435	C	A	0.080	0.105	0.450
HDAC5	QT interval	rs138449302	A	G	0.017	0.606	0.111	4E-08	29.989	A	G	-0.809	0.738	0.270
HDAC5	QT interval	rs138728946	T	C	0.014	-1.509	0.124	3E-34	149.221	T	C	1.030	0.473	0.029
HDAC5	QT interval	rs139566238	A	C	0.014	-0.612	0.088	4E-12	48.358	A	C	0.428	0.465	0.360
HDAC5	QT interval	rs140161293	A	G	0.027	-0.238	0.034	5E-12	47.768	A	G	0.228	0.460	0.620
HDAC5	QT interval	rs141013430	G	A	0.020	0.191	0.031	1E-09	37.354	G	A	0.432	0.439	0.330
HDAC5	QT interval	rs141455452	T	G	0.511	0.259	0.018	7E-48	211.390	T	G	0.131	0.145	0.370
HDAC5	QT interval	rs141461397	G	A	0.024	-0.274	0.035	4E-15	61.777	G	A	1.120	0.500	0.025
HDAC5	QT interval	rs143125392	A	G	0.027	-0.254	0.034	5E-14	56.815	A	G	-0.196	0.334	0.560
HDAC5	QT interval	rs144442307	A	G	0.019	-0.302	0.039	1E-14	59.930	A	G	-0.726	0.430	0.092
HDAC5	QT interval	rs144452577	A	G	0.032	-0.260	0.029	5E-19	79.478	A	G	-0.093	0.260	0.720

HDAC5	QT interval	rs144753516	G	T	0.068	0.446	0.019	8E-126	569.287	G	T	-0.135	0.236	0.570
HDAC5	QT interval	rs144766143	A	G	0.018	0.824	0.113	3E-13	52.958	A	G	0.039	0.516	0.940
HDAC5	QT interval	rs144817232	A	G	0.017	0.378	0.058	9E-11	41.983	A	G	0.374	0.582	0.520
HDAC5	QT interval	rs144997692	G	A	0.024	-0.237	0.039	1E-09	37.315	G	A	0.049	0.395	0.900
HDAC5	QT interval	rs145127226	A	G	0.027	0.246	0.026	6E-21	88.330	A	G	0.107	0.361	0.770
HDAC5	QT interval	rs146674965	G	A	0.038	0.185	0.024	4E-15	61.874	G	A	0.205	0.248	0.410
HDAC5	QT interval	rs147047792	C	T	0.084	-0.243	0.020	4E-33	143.823	C	T	-0.232	0.257	0.370
HDAC5	QT interval	rs147691494	A	G	0.047	-0.225	0.027	1E-16	68.814	A	G	-0.021	0.276	0.940
HDAC5	QT interval	rs148889170	A	G	0.227	-0.266	0.017	1E-54	242.088	A	G	-0.339	0.160	0.035
HDAC5	QT interval	rs151314932	C	T	0.017	0.322	0.035	2E-20	85.770	C	T	-0.204	0.427	0.630
HDAC5	QT interval	rs151325721	T	C	0.014	0.364	0.059	7E-10	38.049	T	C	1.150	0.945	0.220
HDAC5	QT interval	rs181088092	A	G	0.014	-0.393	0.060	5E-11	43.081	A	G	0.378	0.649	0.560
HDAC5	QT interval	rs182630309	T	C	0.013	0.686	0.071	4E-22	93.575	T	C	-0.323	0.984	0.740
HDAC5	QT interval	rs182656281	T	C	0.013	0.846	0.128	4E-11	43.518	T	C	-0.207	0.666	0.760
HDAC5	QT interval	rs187738091	G	A	0.015	-0.622	0.105	4E-09	34.827	G	A	1.080	0.594	0.068
HDAC5	QT interval	rs189151015	A	G	0.020	0.555	0.100	3E-08	30.891	A	G	-2.590	1.050	0.013
HDAC5	QT interval	rs191237882	A	G	0.022	-0.728	0.090	6E-16	65.594	A	G	-0.095	0.405	0.810
HDAC5	QT interval	rs192922438	T	C	0.018	0.292	0.037	4E-15	61.827	T	C	0.033	0.506	0.950
HDAC5	QT interval	rs2023861	T	C	0.063	0.377	0.040	3E-21	89.840	T	C	0.293	0.291	0.310
HDAC5	QT interval	rs2139893	G	A	0.441	0.287	0.011	2E-153	696.570	G	A	-0.227	0.160	0.160
HDAC5	QT interval	rs2458218	C	T	0.146	-0.209	0.013	2E-55	245.555	C	T	0.393	0.161	0.015

HDAC5	QT interval	rs28364026	A	G	0.031	-0.299	0.029	3E-24	103.332	A	G	0.468	0.277	0.091
HDAC5	QT interval	rs28364032	A	G	0.069	0.124	0.016	1E-14	59.830	A	G	-0.377	0.203	0.063
HDAC5	QT interval	rs67155855	A	G	0.044	-0.253	0.029	2E-18	76.794	A	G	0.255	0.397	0.520
HDAC5	QT interval	rs71213817	T	C	0.066	0.177	0.019	6E-21	88.026	T	C	-0.337	0.293	0.250
HDAC5	QT interval	rs7220056	G	A	0.041	0.216	0.031	7E-12	46.944	G	A	0.657	0.376	0.080
HDAC7	Serum phosphate levels	rs1011951	T	C	0.447	-0.066	0.008	2E-16	67.526	T	C	-0.002	0.002	0.550
HDAC7	Serum phosphate levels	rs10783218	A	G	0.028	-0.194	0.030	2E-10	41.011	A	G	-0.010	0.006	0.054
HDAC7	Serum phosphate levels	rs111716416	T	C	0.015	0.453	0.042	2E-27	117.866	T	C	-0.003	0.008	0.640
HDAC7	Serum phosphate levels	rs111925910	A	G	0.021	0.225	0.029	3E-15	61.975	A	G	-0.011	0.008	0.180
HDAC7	Serum phosphate levels	rs11574044	C	A	0.108	0.104	0.014	2E-13	54.205	C	A	-0.005	0.003	0.050
HDAC7	Serum phosphate levels	rs11610206	C	T	0.081	-0.334	0.035	4E-22	93.435	C	T	0.008	0.004	0.042
HDAC7	Serum phosphate levels	rs11611678	A	C	0.015	0.250	0.041	1E-09	36.991	A	C	0.001	0.008	0.910
HDAC7	Serum phosphate levels	rs116973148	T	C	0.020	0.428	0.030	2E-45	199.668	T	C	0.003	0.007	0.840
HDAC7	Serum phosphate levels	rs116982199	T	C	0.025	0.264	0.033	3E-15	62.107	T	C	0.017	0.008	0.069
HDAC7	Serum phosphate levels	rs117126275	G	A	0.020	0.607	0.073	6E-17	69.856	G	A	-0.008	0.007	0.310
HDAC7	Serum phosphate levels	rs117307872	A	C	0.028	0.372	0.058	1E-10	41.173	A	C	-0.009	0.007	0.150
HDAC7	Serum phosphate levels	rs117379012	A	G	0.017	0.280	0.040	2E-12	49.846	A	G	-0.011	0.008	0.260
HDAC7	Serum phosphate levels	rs117693832	C	A	0.019	0.435	0.078	3E-08	31.007	C	A	0.003	0.007	0.830
HDAC7	Serum phosphate levels	rs117804725	A	G	0.023	0.740	0.065	8E-30	128.632	A	G	-0.005	0.007	0.520
HDAC7	Serum phosphate levels	rs117860837	C	T	0.021	0.235	0.033	1E-12	50.740	C	T	0.004	0.007	0.750
HDAC7	Serum phosphate levels	rs118006188	T	C	0.031	-0.423	0.060	1E-12	50.240	T	C	0.002	0.006	0.680
HDAC7	Serum phosphate levels	rs12721404	A	G	0.017	-0.447	0.035	2E-36	158.916	A	G	0.006	0.008	0.430
HDAC7	Serum phosphate levels	rs17721101	C	A	0.054	0.163	0.018	3E-19	80.544	C	A	-0.003	0.004	0.570

HDAC7	Serum phosphate levels	rs17885398	T	C	0.015	-0.309	0.051	1E-09	36.637	T	C	-0.001	0.008	0.920
HDAC7	Serum phosphate levels	rs1859281	A	G	0.062	-0.260	0.017	6E-55	243.604	A	G	0.009	0.004	0.075
HDAC7	Serum phosphate levels	rs2107301	A	G	0.299	0.074	0.009	2E-17	72.710	A	G	0.003	0.002	0.240
HDAC7	Serum phosphate levels	rs214695	G	A	0.053	0.212	0.021	3E-24	103.490	G	A	-0.003	0.004	0.480
HDAC7	Serum phosphate levels	rs214732	C	T	0.111	0.553	0.029	5E-79	354.435	C	T	0.001	0.003	0.760
HDAC7	Serum phosphate levels	rs2189480	T	G	0.372	0.057	0.008	5E-12	47.588	T	G	-0.001	0.002	0.700
HDAC7	Serum phosphate levels	rs2408876	C	T	0.355	0.081	0.011	2E-13	54.294	C	T	0.001	0.002	0.530
HDAC7	Serum phosphate levels	rs2936558	A	G	0.045	0.232	0.022	1E-25	109.420	A	G	0.001	0.005	0.800
HDAC7	Serum phosphate levels	rs34739581	A	G	0.018	0.244	0.035	4E-12	48.239	A	G	-0.009	0.008	0.280
HDAC7	Serum phosphate levels	rs34974869	A	C	0.060	-0.279	0.017	2E-58	260.260	A	C	-0.003	0.005	0.690
HDAC7	Serum phosphate levels	rs3782905	C	G	0.311	-0.073	0.009	2E-17	71.951	C	G	0.003	0.002	0.330
HDAC7	Serum phosphate levels	rs3815131	T	C	0.306	0.074	0.009	7E-18	74.080	T	C	0.005	0.002	0.046
HDAC7	Serum phosphate levels	rs4141223	A	G	0.048	-0.183	0.019	1E-22	95.613	A	G	0.008	0.005	0.160
HDAC7	Serum phosphate levels	rs56304982	C	G	0.274	0.089	0.009	2E-23	99.644	C	G	-0.002	0.002	0.470
HDAC7	Serum phosphate levels	rs61927719	A	G	0.149	-0.164	0.028	3E-09	35.265	A	G	0.003	0.003	0.440
HDAC7	Serum phosphate levels	rs6580637	T	C	0.269	0.062	0.009	5E-12	47.751	T	C	0.002	0.002	0.680
HDAC7	Serum phosphate levels	rs6580639	C	T	0.037	-0.145	0.024	9E-10	37.560	C	T	-0.011	0.006	0.091
HDAC7	Serum phosphate levels	rs685558	A	C	0.053	0.239	0.019	2E-37	163.923	A	C	0.001	0.005	0.710
HDAC7	Serum phosphate levels	rs72644833	T	C	0.035	0.217	0.024	3E-19	80.188	T	C	0.003	0.006	0.560
HDAC7	Serum phosphate levels	rs73093150	G	A	0.079	-0.144	0.015	8E-21	87.554	G	A	-0.003	0.004	0.490
HDAC7	Serum phosphate levels	rs73107414	T	C	0.016	-0.326	0.040	8E-16	64.860	T	C	-0.003	0.009	0.790
HDAC7	Serum phosphate levels	rs73109829	A	G	0.026	0.241	0.027	5E-19	79.271	A	G	-0.002	0.006	0.830
HDAC7	Serum phosphate levels	rs74917912	T	C	0.069	0.239	0.037	2E-10	40.717	T	C	0.004	0.004	0.330

HDAC7	Serum phosphate levels	rs77290820	A	G	0.042	0.162	0.022	1E-13	54.609	A	G	-0.007	0.005	0.110
HDAC7	Serum phosphate levels	rs78953588	C	T	0.022	0.240	0.033	3E-13	52.972	C	T	-0.001	0.007	0.870
HDAC7	Serum phosphate levels	rs7972177	G	A	0.299	-0.130	0.009	6E-51	225.323	G	A	-0.003	0.002	0.170
HDAC7	Serum phosphate levels	rs7975232	C	A	0.475	0.136	0.009	7E-56	248.171	C	A	0.000	0.002	0.810
HDAC7	Serum phosphate levels	rs7975604	T	C	0.055	-0.115	0.018	6E-11	42.769	T	C	0.000	0.006	0.750
HDAC7	Serum phosphate levels	rs80009138	G	A	0.023	-0.496	0.064	1E-14	59.496	G	A	-0.010	0.007	0.270
HDAC7	Serum phosphate levels	rs80083409	A	C	0.038	0.319	0.056	1E-08	32.863	A	C	0.000	0.005	0.920
HDAC7	Serum phosphate levels	rs9645810	T	C	0.039	-0.196	0.022	2E-19	80.950	T	C	0.014	0.005	0.009
HDAC7	QT interval	rs1011951	T	C	0.447	-0.066	0.008	2E-16	67.526	T	C	-0.056	0.103	0.590
HDAC7	QT interval	rs10783218	A	G	0.028	-0.194	0.030	2E-10	41.011	A	G	0.080	0.377	0.830
HDAC7	QT interval	rs10785672	C	A	0.281	0.073	0.010	9E-13	51.143	C	A	0.041	0.116	0.730
HDAC7	QT interval	rs10785676	G	A	0.279	-0.260	0.011	1E-131	595.828	G	A	0.045	0.110	0.690
HDAC7	QT interval	rs10875650	C	A	0.322	-0.104	0.010	2E-27	117.996	C	A	-0.071	0.110	0.520
HDAC7	QT interval	rs10875667	G	A	0.157	-0.401	0.012	1E-200	1140.029	G	A	-0.060	0.145	0.680
HDAC7	QT interval	rs10881014	A	G	0.259	0.067	0.009	1E-13	54.961	A	G	0.037	0.123	0.770
HDAC7	QT interval	rs10881060	T	C	0.052	0.178	0.020	2E-18	76.899	T	C	-0.122	0.238	0.610
HDAC7	QT interval	rs10881087	G	A	0.475	-0.078	0.009	3E-18	76.028	G	A	0.022	0.101	0.820
HDAC7	QT interval	rs10881091	T	C	0.048	0.449	0.044	2E-24	103.632	T	C	0.048	0.242	0.840
HDAC7	QT interval	rs10881092	G	T	0.463	-0.072	0.009	2E-15	63.012	G	T	-0.017	0.101	0.860
HDAC7	QT interval	rs10881101	T	C	0.212	0.150	0.011	4E-44	193.974	T	C	-0.235	0.125	0.060
HDAC7	QT interval	rs10881107	A	G	0.135	0.165	0.013	1E-37	164.337	A	G	0.031	0.151	0.840

HDAC7	QT interval	rs1101816	T	C	0.300	0.784	0.019	1E-200	1736.085	T	C	0.017	0.111	0.880
HDAC7	QT interval	rs111678931	A	G	0.046	0.128	0.021	7E-10	38.041	A	G	-0.064	0.271	0.810
HDAC7	QT interval	rs11168184	A	G	0.445	0.076	0.010	1E-15	64.288	A	G	0.100	0.113	0.380
HDAC7	QT interval	rs11168220	A	G	0.261	-0.224	0.009	7E-137	620.249	A	G	0.040	0.118	0.740
HDAC7	QT interval	rs11168228	G	A	0.301	-0.135	0.009	8E-55	243.168	G	A	0.045	0.112	0.690
HDAC7	QT interval	rs11168262	T	C	0.136	0.080	0.012	2E-11	45.460	T	C	0.075	0.144	0.610
HDAC7	QT interval	rs11168274	T	C	0.074	-0.100	0.017	4E-09	34.673	T	C	-0.028	0.154	0.850
HDAC7	QT interval	rs11168275	C	T	0.240	0.065	0.010	3E-10	39.806	C	T	0.294	0.129	0.023
HDAC7	QT interval	rs11168284	G	A	0.344	0.093	0.009	5E-25	106.746	G	A	-0.133	0.116	0.250
HDAC7	QT interval	rs11168286	A	G	0.111	0.121	0.014	2E-17	72.315	A	G	-0.269	0.177	0.130
HDAC7	QT interval	rs111687147	G	A	0.061	-0.190	0.019	1E-24	104.785	G	A	-0.074	0.213	0.730
HDAC7	QT interval	rs111716416	T	C	0.015	0.453	0.042	2E-27	117.866	T	C	-0.154	0.453	0.730
HDAC7	QT interval	rs11183643	C	T	0.491	0.131	0.020	2E-11	44.652	C	T	0.076	0.103	0.460
HDAC7	QT interval	rs11183762	G	A	0.147	-0.111	0.011	9E-23	96.417	G	A	-0.077	0.154	0.620
HDAC7	QT interval	rs11183802	A	G	0.094	0.086	0.014	3E-10	39.539	A	G	0.294	0.174	0.091
HDAC7	QT interval	rs11183810	A	G	0.014	0.775	0.115	1E-11	45.692	A	G	-0.356	0.470	0.450
HDAC7	QT interval	rs11183848	A	G	0.059	-0.127	0.018	2E-12	49.050	A	G	-0.278	0.220	0.210
HDAC7	QT interval	rs11183956	A	G	0.103	0.267	0.031	9E-18	73.702	A	G	0.261	0.167	0.120
HDAC7	QT interval	rs111925910	A	G	0.021	0.225	0.029	3E-15	61.975	A	G	-0.250	0.410	0.540
HDAC7	QT interval	rs112335632	T	C	0.050	0.167	0.021	7E-15	60.678	T	C	-0.192	0.254	0.450
HDAC7	QT interval	rs1123746	A	G	0.277	0.094	0.010	3E-21	89.696	A	G	0.056	0.118	0.640

HDAC7	QT interval	rs112726671	G	A	0.017	0.202	0.032	4E-10	39.215	G	A	-0.068	0.362	0.850
HDAC7	QT interval	rs112859712	T	C	0.023	-0.832	0.095	2E-18	76.832	T	C	1.110	1.230	0.370
HDAC7	QT interval	rs113104382	A	G	0.037	-0.489	0.065	4E-14	57.290	A	G	-0.437	1.010	0.670
HDAC7	QT interval	rs113111572	T	G	0.100	0.414	0.031	7E-40	174.725	T	G	-0.136	0.176	0.440
HDAC7	QT interval	rs113958599	C	T	0.026	0.170	0.027	3E-10	39.720	C	T	-0.671	0.392	0.087
HDAC7	QT interval	rs11520333	T	C	0.175	0.348	0.025	1E-42	187.043	T	C	0.054	0.141	0.700
HDAC7	QT interval	rs1153977	T	A	0.481	-0.146	0.008	1E-75	339.026	T	A	-0.136	0.101	0.180
HDAC7	QT interval	rs11574024	T	G	0.078	-0.177	0.015	3E-31	135.005	T	G	-0.239	0.211	0.260
HDAC7	QT interval	rs11574027	A	C	0.021	0.248	0.032	1E-14	59.874	A	C	0.828	0.348	0.017
HDAC7	QT interval	rs11574044	C	A	0.108	0.104	0.014	2E-13	54.205	C	A	-0.033	0.163	0.840
HDAC7	QT interval	rs11574052	T	C	0.040	0.142	0.020	4E-12	48.067	T	C	0.213	0.301	0.480
HDAC7	QT interval	rs11574143	T	C	0.103	-0.164	0.013	5E-36	157.080	T	C	-0.131	0.162	0.420
HDAC7	QT interval	rs11610206	C	T	0.081	-0.334	0.035	4E-22	93.435	C	T	0.184	0.172	0.290
HDAC7	QT interval	rs11611678	A	C	0.015	0.250	0.041	1E-09	36.991	A	C	-0.688	0.512	0.180
HDAC7	QT interval	rs11611892	A	C	0.028	0.592	0.064	3E-20	85.256	A	C	-0.622	0.340	0.067
HDAC7	QT interval	rs116858826	C	T	0.016	-0.671	0.096	2E-12	49.238	C	T	0.548	0.554	0.320
HDAC7	QT interval	rs116973148	T	C	0.020	0.428	0.030	2E-45	199.668	T	C	-0.203	0.359	0.570
HDAC7	QT interval	rs116982199	T	C	0.025	0.264	0.033	3E-15	62.107	T	C	0.239	0.419	0.570
HDAC7	QT interval	rs117126275	G	A	0.020	0.607	0.073	6E-17	69.856	G	A	0.256	0.395	0.520
HDAC7	QT interval	rs117267013	T	C	0.057	0.254	0.018	1E-43	192.300	T	C	0.153	0.225	0.500
HDAC7	QT interval	rs117307872	A	C	0.028	0.372	0.058	1E-10	41.173	A	C	0.139	0.366	0.710
HDAC7	QT interval	rs117379012	A	G	0.017	0.280	0.040	2E-12	49.846	A	G	0.058	0.483	0.900
HDAC7	QT interval	rs117397073	A	G	0.015	-0.504	0.078	1E-10	41.340	A	G	0.622	0.684	0.360

HDAC7	QT interval	rs117499636	A	G	0.022	0.218	0.032	1E-11	45.746	A	G	-0.223	0.447	0.620
HDAC7	QT interval	rs117693832	C	A	0.019	0.435	0.078	3E-08	31.007	C	A	-0.318	0.362	0.380
HDAC7	QT interval	rs117703725	A	G	0.015	0.448	0.046	2E-22	95.371	A	G	0.509	0.555	0.360
HDAC7	QT interval	rs117709065	T	C	0.018	-0.284	0.033	2E-17	72.002	T	C	0.576	0.466	0.220
HDAC7	QT interval	rs117733247	T	C	0.022	-0.203	0.031	1E-10	41.874	T	C	-0.043	0.412	0.920
HDAC7	QT interval	rs117804725	A	G	0.023	0.740	0.065	8E-30	128.632	A	G	-0.483	0.381	0.200
HDAC7	QT interval	rs117860837	C	T	0.021	0.235	0.033	1E-12	50.740	C	T	-0.402	0.448	0.370
HDAC7	QT interval	rs117867199	C	T	0.025	-0.252	0.030	8E-17	69.402	C	T	0.115	0.373	0.760
HDAC7	QT interval	rs117961330	A	G	0.026	0.459	0.068	2E-11	45.024	A	G	-0.014	0.355	0.970
HDAC7	QT interval	rs117965278	C	T	0.020	0.438	0.033	3E-40	176.301	C	T	0.447	0.437	0.310
HDAC7	QT interval	rs118006188	T	C	0.031	-0.423	0.060	1E-12	50.240	T	C	0.006	0.298	0.980
HDAC7	QT interval	rs12231051	T	C	0.161	0.235	0.012	2E-88	397.859	T	C	-0.020	0.124	0.870
HDAC7	QT interval	rs12301106	A	G	0.128	-0.090	0.013	1E-11	46.118	A	G	-0.179	0.145	0.220
HDAC7	QT interval	rs12301925	C	T	0.328	-0.164	0.008	1E-83	375.590	C	T	-0.219	0.108	0.043
HDAC7	QT interval	rs1232978	T	C	0.158	-0.195	0.011	5E-72	322.236	T	C	-0.094	0.138	0.500
HDAC7	QT interval	rs1236637	G	A	0.470	-0.126	0.008	1E-56	251.547	G	A	0.015	0.101	0.880
HDAC7	QT interval	rs12369526	A	G	0.231	-0.264	0.022	3E-32	140.086	A	G	0.098	0.119	0.410
HDAC7	QT interval	rs12425772	T	C	0.037	0.518	0.050	4E-25	107.292	T	C	0.126	0.283	0.660
HDAC7	QT interval	rs12721375	A	G	0.124	-0.157	0.021	2E-13	53.592	A	G	-0.062	0.169	0.710
HDAC7	QT interval	rs12721404	A	G	0.017	-0.447	0.035	2E-36	158.916	A	G	-0.438	0.509	0.390
HDAC7	QT interval	rs12813049	A	G	0.091	0.206	0.015	1E-43	191.678	A	G	-0.110	0.182	0.550
HDAC7	QT interval	rs12826679	T	G	0.270	-0.244	0.021	1E-30	132.895	T	G	0.250	0.114	0.029
HDAC7	QT interval	rs12830810	C	T	0.231	0.116	0.010	3E-34	149.046	C	T	0.172	0.124	0.170

HDAC7	QT interval	rs1357935	T	G	0.312	0.131	0.009	2E-43	191.136	T	G	-0.052	0.108	0.630
HDAC7	QT interval	rs138126224	T	G	0.022	-0.354	0.064	4E-08	30.072	T	G	-0.123	0.429	0.770
HDAC7	QT interval	rs138337034	T	C	0.018	0.299	0.032	1E-20	86.527	T	C	1.650	0.580	0.004
HDAC7	QT interval	rs138409438	T	C	0.042	0.304	0.047	1E-10	41.142	T	C	0.067	0.252	0.790
HDAC7	QT interval	rs139004597	A	C	0.021	-0.206	0.030	5E-12	47.833	A	C	-0.485	0.376	0.200
HDAC7	QT interval	rs141596925	T	G	0.014	0.299	0.046	8E-11	42.271	T	G	-0.191	0.628	0.760
HDAC7	QT interval	rs142011798	A	G	0.045	0.400	0.051	6E-15	60.831	A	G	0.035	0.270	0.900
HDAC7	QT interval	rs142012346	T	C	0.013	1.361	0.237	1E-08	32.823	T	C	-0.232	0.573	0.690
HDAC7	QT interval	rs142096752	T	C	0.017	-0.272	0.048	1E-08	32.483	T	C	-0.117	0.634	0.850
HDAC7	QT interval	rs142180605	A	G	0.015	-0.861	0.128	1E-11	45.535	A	G	0.170	0.628	0.790
HDAC7	QT interval	rs143040286	G	A	0.018	0.520	0.072	6E-13	51.696	G	A	0.107	0.486	0.830
HDAC7	QT interval	rs143512419	A	G	0.013	0.280	0.045	5E-10	38.659	A	G	-0.205	0.457	0.650
HDAC7	QT interval	rs144201754	T	C	0.036	0.504	0.065	9E-15	60.081	T	C	0.618	0.866	0.480
HDAC7	QT interval	rs144377854	A	G	0.022	-0.594	0.085	2E-12	49.331	A	G	0.868	0.895	0.330
HDAC7	QT interval	rs144856064	A	G	0.040	0.144	0.021	1E-11	46.414	A	G	-0.265	0.262	0.310
HDAC7	QT interval	rs145242567	T	C	0.055	-0.144	0.018	4E-15	61.484	T	C	-0.256	0.257	0.320
HDAC7	QT interval	rs145426754	C	A	0.020	-0.233	0.029	2E-15	62.843	C	A	0.415	0.373	0.270
HDAC7	QT interval	rs146604675	C	T	0.014	-0.895	0.063	7E-46	202.055	C	T	1.130	0.469	0.016
HDAC7	QT interval	rs146681026	T	C	0.015	-0.255	0.044	7E-09	33.464	T	C	-0.638	0.556	0.250
HDAC7	QT interval	rs147037634	T	C	0.012	-0.631	0.077	2E-16	67.904	T	C	-1.710	0.794	0.031
HDAC7	QT interval	rs147500985	A	G	0.041	0.667	0.053	7E-36	156.325	A	G	0.505	0.271	0.063
HDAC7	QT interval	rs148620323	T	C	0.015	-0.585	0.058	3E-24	102.945	T	C	-0.267	0.597	0.650
HDAC7	QT interval	rs148774106	G	T	0.014	-0.778	0.044	2E-70	314.787	G	T	-1.010	0.689	0.140

HDAC7	QT interval	rs149188661	T	C	0.014	0.379	0.060	3E-10	39.559	T	C	-0.994	0.416	0.017
HDAC7	QT interval	rs149870309	T	C	0.020	-0.326	0.045	4E-13	52.600	T	C	1.050	0.520	0.042
HDAC7	QT interval	rs150129738	A	C	0.012	-0.622	0.107	6E-09	33.966	A	C	-0.806	0.682	0.240
HDAC7	QT interval	rs1623199	T	C	0.084	0.168	0.016	1E-25	109.604	T	C	0.192	0.187	0.300
HDAC7	QT interval	rs17097720	G	C	0.065	-0.355	0.038	3E-20	85.318	G	C	-0.006	0.206	0.980
HDAC7	QT interval	rs17121947	T	C	0.056	-0.250	0.019	4E-38	166.483	T	C	-0.068	0.229	0.770
HDAC7	QT interval	rs17721101	C	A	0.054	0.163	0.018	3E-19	80.544	C	A	-0.107	0.215	0.620
HDAC7	QT interval	rs17880972	C	T	0.046	0.320	0.019	1E-63	283.485	C	T	0.200	0.250	0.420
HDAC7	QT interval	rs17885398	T	C	0.015	-0.309	0.051	1E-09	36.637	T	C	-0.351	0.537	0.510
HDAC7	QT interval	rs1808208	G	A	0.195	-0.092	0.010	2E-19	81.029	G	A	0.031	0.130	0.810
HDAC7	QT interval	rs181052866	C	T	0.015	-0.871	0.085	2E-24	103.852	C	T	-1.140	0.815	0.160
HDAC7	QT interval	rs183860398	A	G	0.014	-0.516	0.086	2E-09	36.012	A	G	0.091	0.576	0.870
HDAC7	QT interval	rs184145049	A	G	0.017	0.527	0.093	2E-08	31.993	A	G	-0.069	0.680	0.920
HDAC7	QT interval	rs184639345	T	G	0.023	0.222	0.040	3E-08	31.009	T	G	-0.091	0.572	0.870
HDAC7	QT interval	rs1859281	A	G	0.062	-0.260	0.017	6E-55	243.604	A	G	-0.022	0.213	0.920
HDAC7	QT interval	rs185952996	T	C	0.016	0.634	0.105	1E-09	36.690	T	C	0.535	0.483	0.270
HDAC7	QT interval	rs186641960	G	A	0.012	0.908	0.159	1E-08	32.396	G	A	0.080	0.572	0.890
HDAC7	QT interval	rs188612729	A	G	0.010	0.734	0.114	1E-10	41.489	A	G	0.015	0.647	0.980
HDAC7	QT interval	rs190848850	T	C	0.018	-0.284	0.032	2E-18	77.120	T	C	0.624	0.439	0.160
HDAC7	QT interval	rs191340575	T	C	0.019	-0.417	0.069	2E-09	36.465	T	C	-0.208	0.769	0.790
HDAC7	QT interval	rs1919729	C	G	0.466	-0.084	0.009	3E-21	89.745	C	G	-0.058	0.102	0.570
HDAC7	QT interval	rs193072227	A	G	0.013	1.562	0.152	8E-25	105.835	A	G	0.646	0.509	0.200
HDAC7	QT interval	rs193280568	T	G	0.025	0.590	0.095	5E-10	38.626	T	G	0.571	1.220	0.640

HDAC7	QT interval	rs1949861	A	T	0.028	-0.865	0.056	2E-53	236.329	A	T	-0.230	0.276	0.400
HDAC7	QT interval	rs2011637	C	A	0.264	0.074	0.010	1E-13	54.929	C	A	-0.079	0.115	0.490
HDAC7	QT interval	rs2025	T	C	0.094	0.113	0.014	1E-16	68.491	T	C	-0.103	0.174	0.560
HDAC7	QT interval	rs2040643	A	G	0.444	0.074	0.008	1E-20	86.535	A	G	-0.052	0.102	0.610
HDAC7	QT interval	rs2107301	A	G	0.299	0.074	0.009	2E-17	72.710	A	G	-0.025	0.117	0.830
HDAC7	QT interval	rs214649	G	A	0.067	0.244	0.018	4E-43	189.410	G	A	0.096	0.207	0.640
HDAC7	QT interval	rs214659	A	G	0.240	0.148	0.024	9E-10	37.550	A	G	-0.077	0.123	0.530
HDAC7	QT interval	rs214671	A	G	0.200	0.193	0.011	5E-68	303.832	A	G	-0.251	0.126	0.047
HDAC7	QT interval	rs214675	T	C	0.362	0.158	0.020	1E-15	64.305	T	C	0.088	0.104	0.400
HDAC7	QT interval	rs214684	A	G	0.254	-0.241	0.022	9E-29	123.766	A	G	-0.133	0.118	0.260
HDAC7	QT interval	rs214695	G	A	0.053	0.212	0.021	3E-24	103.490	G	A	-0.208	0.224	0.350
HDAC7	QT interval	rs214699	A	G	0.343	0.108	0.009	1E-30	132.435	A	G	-0.148	0.106	0.170
HDAC7	QT interval	rs214732	C	T	0.111	0.553	0.029	5E-79	354.435	C	T	0.364	0.160	0.023
HDAC7	QT interval	rs215332	T	C	0.343	0.053	0.008	2E-10	40.572	T	C	-0.140	0.108	0.200
HDAC7	QT interval	rs215338	A	G	0.018	0.259	0.038	6E-12	47.456	A	G	0.225	0.427	0.600
HDAC7	QT interval	rs215349	A	T	0.255	-0.132	0.010	6E-39	170.500	A	T	-0.043	0.117	0.720
HDAC7	QT interval	rs215355	G	C	0.322	0.222	0.010	2E-103	466.198	G	C	-0.085	0.111	0.440
HDAC7	QT interval	rs2189480	T	G	0.372	0.057	0.008	5E-12	47.588	T	G	-0.011	0.105	0.910
HDAC7	QT interval	rs2238136	T	C	0.252	-0.129	0.009	5E-42	184.336	T	C	-0.021	0.131	0.880
HDAC7	QT interval	rs2239181	C	A	0.107	-0.118	0.013	4E-20	84.530	C	A	-0.198	0.166	0.230
HDAC7	QT interval	rs2240109	T	C	0.227	0.079	0.010	2E-16	68.033	T	C	0.145	0.129	0.260
HDAC7	QT interval	rs2408635	T	G	0.131	0.154	0.028	4E-08	29.984	T	G	0.176	0.145	0.230

HDAC7	QT interval	rs2408876	C	T	0.355	0.081	0.011	2E-13	54.294	C	T	-0.226	0.120	0.060
HDAC7	QT interval	rs2465618	G	A	0.123	-0.199	0.029	7E-12	46.928	G	A	-0.032	0.152	0.840
HDAC7	QT interval	rs2525055	G	T	0.287	-0.121	0.009	1E-42	187.669	G	T	-0.128	0.118	0.280
HDAC7	QT interval	rs2543740	G	T	0.242	-0.069	0.010	3E-11	44.161	G	T	0.138	0.115	0.230
HDAC7	QT interval	rs2544028	T	A	0.370	-0.058	0.008	1E-12	50.333	T	A	-0.119	0.110	0.280
HDAC7	QT interval	rs2698790	T	C	0.056	0.182	0.019	3E-21	89.363	T	C	0.203	0.223	0.360
HDAC7	QT interval	rs2698792	T	C	0.390	-0.179	0.021	5E-17	70.537	T	C	-0.037	0.103	0.720
HDAC7	QT interval	rs2698794	A	G	0.348	-0.061	0.009	3E-11	43.901	A	G	-0.093	0.105	0.380
HDAC7	QT interval	rs28458307	G	T	0.044	0.182	0.025	7E-13	51.512	G	T	-0.659	0.319	0.038
HDAC7	QT interval	rs2853563	T	C	0.038	0.115	0.021	4E-08	30.184	T	C	0.067	0.267	0.800
HDAC7	QT interval	rs2853566	A	G	0.477	-0.093	0.008	5E-31	134.217	A	G	-0.032	0.111	0.770
HDAC7	QT interval	rs2936558	A	G	0.045	0.232	0.022	1E-25	109.420	A	G	-0.115	0.254	0.650
HDAC7	QT interval	rs34692456	T	A	0.087	-0.125	0.016	2E-15	63.473	T	A	-0.244	0.177	0.170
HDAC7	QT interval	rs34739581	A	G	0.018	0.244	0.035	4E-12	48.239	A	G	-0.719	0.412	0.081
HDAC7	QT interval	rs34785353	C	T	0.135	-0.116	0.016	5E-13	52.192	C	T	-0.176	0.178	0.320
HDAC7	QT interval	rs34974869	A	C	0.060	-0.279	0.017	2E-58	260.260	A	C	0.099	0.230	0.670
HDAC7	QT interval	rs3782905	C	G	0.311	-0.073	0.009	2E-17	71.951	C	G	0.052	0.110	0.640
HDAC7	QT interval	rs3809316	A	G	0.508	-0.062	0.008	8E-15	60.252	A	G	-0.081	0.107	0.450
HDAC7	QT interval	rs3815131	T	C	0.306	0.074	0.009	7E-18	74.080	T	C	-0.079	0.116	0.500
HDAC7	QT interval	rs3815137	T	C	0.116	-0.185	0.012	2E-50	223.092	T	C	0.003	0.157	0.980
HDAC7	QT interval	rs4141223	A	G	0.048	-0.183	0.019	1E-22	95.613	A	G	0.469	0.255	0.066
HDAC7	QT interval	rs4503624	A	G	0.290	0.059	0.010	1E-09	37.266	A	G	-0.034	0.117	0.770
HDAC7	QT interval	rs4570672	A	G	0.374	-0.125	0.010	8E-36	156.155	A	G	-0.136	0.104	0.190

HDAC7	QT interval	rs4760603	T	A	0.350	-0.096	0.009	3E-26	112.500	T	A	-0.084	0.106	0.430
HDAC7	QT interval	rs4760626	A	G	0.182	0.182	0.011	7E-67	298.579	A	G	0.241	0.145	0.096
HDAC7	QT interval	rs4760650	G	T	0.496	0.109	0.011	6E-25	106.329	G	T	0.054	0.120	0.660
HDAC7	QT interval	rs4760671	G	C	0.462	0.126	0.009	1E-47	210.337	G	C	0.113	0.106	0.290
HDAC7	QT interval	rs4768158	T	C	0.063	-0.302	0.016	1E-76	343.488	T	C	-0.099	0.188	0.600
HDAC7	QT interval	rs4768804	C	T	0.041	-0.124	0.022	2E-08	31.421	C	T	-0.128	0.250	0.610
HDAC7	QT interval	rs4768814	T	C	0.298	0.071	0.010	2E-13	54.432	T	C	0.136	0.112	0.230
HDAC7	QT interval	rs55713656	A	G	0.144	0.170	0.012	2E-49	218.029	A	G	-0.251	0.151	0.096
HDAC7	QT interval	rs55900360	G	A	0.134	-0.172	0.028	7E-10	37.955	G	A	-0.118	0.163	0.470
HDAC7	QT interval	rs55958137	T	A	0.281	0.098	0.009	4E-28	121.062	T	A	-0.005	0.112	0.970
HDAC7	QT interval	rs56047798	T	G	0.064	-0.098	0.017	2E-08	31.190	T	G	-0.005	0.203	0.980
HDAC7	QT interval	rs56050793	A	G	0.137	-0.093	0.013	3E-13	52.955	A	G	0.081	0.155	0.600
HDAC7	QT interval	rs56304982	C	G	0.274	0.089	0.009	2E-23	99.644	C	G	0.027	0.118	0.820
HDAC7	QT interval	rs56411627	G	A	0.096	-0.373	0.036	3E-25	107.492	G	A	-0.153	0.175	0.380
HDAC7	QT interval	rs57845861	A	C	0.073	-0.265	0.038	2E-12	49.635	A	C	0.026	0.175	0.880
HDAC7	QT interval	rs59330359	T	G	0.047	0.176	0.023	2E-14	58.241	T	G	0.308	0.230	0.180
HDAC7	QT interval	rs61045067	A	G	0.103	0.123	0.015	1E-15	63.757	A	G	-0.141	0.159	0.370
HDAC7	QT interval	rs61751601	G	A	0.016	-0.217	0.034	1E-10	41.435	G	A	-0.717	0.420	0.088
HDAC7	QT interval	rs61917650	A	G	0.150	-0.075	0.011	7E-11	42.594	A	G	0.130	0.164	0.430
HDAC7	QT interval	rs61918136	A	C	0.084	-0.564	0.014	1E-200	1623.440	A	C	-0.107	0.185	0.560
HDAC7	QT interval	rs61927669	T	C	0.024	-0.428	0.073	4E-09	34.593	T	C	-0.041	0.303	0.890
HDAC7	QT interval	rs61927719	A	G	0.149	-0.164	0.028	3E-09	35.265	A	G	-0.024	0.147	0.870

HDAC7	QT interval	rs6580634	T	C	0.417	-0.105	0.008	5E-39	170.685	T	C	0.081	0.104	0.440
HDAC7	QT interval	rs6580637	T	C	0.269	0.062	0.009	5E-12	47.751	T	C	0.050	0.124	0.680
HDAC7	QT interval	rs6580639	C	T	0.037	-0.145	0.024	9E-10	37.560	C	T	-0.329	0.320	0.300
HDAC7	QT interval	rs685558	A	C	0.053	0.239	0.019	2E-37	163.923	A	C	0.478	0.251	0.057
HDAC7	QT interval	rs712094	C	T	0.468	0.151	0.010	1E-54	242.362	C	T	-0.039	0.101	0.700
HDAC7	QT interval	rs71445733	G	A	0.109	0.101	0.013	3E-15	62.438	G	A	0.177	0.158	0.260
HDAC7	QT interval	rs71462977	T	C	0.036	-0.194	0.023	3E-17	71.619	T	C	0.417	0.350	0.230
HDAC7	QT interval	rs72644833	T	C	0.035	0.217	0.024	3E-19	80.188	T	C	0.343	0.247	0.170
HDAC7	QT interval	rs72644839	A	G	0.070	-0.221	0.037	3E-09	35.027	A	G	-0.191	0.188	0.310
HDAC7	QT interval	rs727121	G	A	0.254	-0.274	0.009	1E-200	924.137	G	A	0.108	0.120	0.370
HDAC7	QT interval	rs7299689	T	C	0.049	-0.315	0.018	4E-66	295.077	T	C	0.374	0.247	0.130
HDAC7	QT interval	rs7300569	A	C	0.121	0.074	0.012	2E-09	35.694	A	C	0.131	0.155	0.400
HDAC7	QT interval	rs73093116	T	C	0.042	0.158	0.025	3E-10	39.907	T	C	-0.225	0.285	0.430
HDAC7	QT interval	rs73093150	G	A	0.079	-0.144	0.015	8E-21	87.554	G	A	-0.446	0.209	0.032
HDAC7	QT interval	rs73101751	G	T	0.127	0.187	0.013	2E-45	200.613	G	T	-0.054	0.159	0.730
HDAC7	QT interval	rs73102192	G	T	0.134	0.122	0.012	1E-25	109.888	G	T	0.170	0.150	0.260
HDAC7	QT interval	rs73105356	G	A	0.032	0.251	0.026	5E-22	92.914	G	A	0.157	0.331	0.640
HDAC7	QT interval	rs73105366	T	C	0.073	-0.164	0.015	9E-27	114.684	T	C	-0.120	0.190	0.530
HDAC7	QT interval	rs73105873	T	C	0.021	0.228	0.030	2E-14	58.095	T	C	-0.199	0.461	0.670
HDAC7	QT interval	rs73107414	T	C	0.016	-0.326	0.040	8E-16	64.860	T	C	-0.421	0.505	0.400
HDAC7	QT interval	rs73107953	A	G	0.075	-0.105	0.017	5E-10	38.828	A	G	-0.160	0.221	0.470

HDAC7	QT interval	rs73107964	T	C	0.205	0.213	0.010	3E-102	461.182	T	C	-0.033	0.133	0.800
HDAC7	QT interval	rs73109409	T	C	0.047	0.485	0.045	1E-27	118.435	T	C	0.149	0.267	0.580
HDAC7	QT interval	rs73109829	A	G	0.026	0.241	0.027	5E-19	79.271	A	G	-0.146	0.334	0.660
HDAC7	QT interval	rs73109883	A	G	0.196	0.124	0.010	2E-35	154.275	A	G	0.272	0.133	0.040
HDAC7	QT interval	rs73109892	C	T	0.015	0.574	0.093	7E-10	37.948	C	T	0.589	0.659	0.370
HDAC7	QT interval	rs73111983	T	C	0.036	-0.470	0.055	2E-17	71.771	T	C	-0.024	0.292	0.940
HDAC7	QT interval	rs7313879	T	C	0.085	-0.239	0.034	2E-12	49.562	T	C	-0.166	0.181	0.360
HDAC7	QT interval	rs7313977	C	G	0.081	-0.192	0.015	8E-40	174.366	C	G	-0.339	0.176	0.054
HDAC7	QT interval	rs7314534	T	C	0.117	-0.390	0.029	7E-41	179.217	T	C	-0.096	0.158	0.540
HDAC7	QT interval	rs73302702	C	A	0.102	-0.434	0.013	1E-200	1122.147	C	A	-0.118	0.173	0.490
HDAC7	QT interval	rs734608	C	T	0.208	0.059	0.010	1E-09	36.561	C	T	-0.015	0.126	0.900
HDAC7	QT interval	rs739842	T	C	0.389	0.050	0.008	9E-10	37.619	T	C	0.114	0.116	0.330
HDAC7	QT interval	rs74435373	C	G	0.065	-0.135	0.018	1E-13	55.042	C	G	0.695	0.228	0.002
HDAC7	QT interval	rs74557864	A	G	0.046	0.485	0.051	9E-22	91.968	A	G	-0.254	0.252	0.310
HDAC7	QT interval	rs74917912	T	C	0.069	0.239	0.037	2E-10	40.717	T	C	0.009	0.200	0.970
HDAC7	QT interval	rs75178219	T	G	0.015	0.585	0.093	4E-10	39.093	T	G	-0.090	0.565	0.870
HDAC7	QT interval	rs75422866	T	C	0.029	-0.776	0.024	1E-200	1033.849	T	C	-0.259	0.327	0.430
HDAC7	QT interval	rs757344	T	C	0.521	-0.076	0.009	7E-19	78.812	T	C	-0.075	0.112	0.500
HDAC7	QT interval	rs75912544	A	G	0.079	-0.256	0.016	1E-55	247.118	A	G	0.402	0.198	0.042
HDAC7	QT interval	rs76092831	T	C	0.037	0.489	0.050	1E-22	96.248	T	C	0.121	0.272	0.660
HDAC7	QT interval	rs76247632	C	T	0.020	-0.387	0.045	3E-18	75.770	C	T	0.486	0.649	0.450

HDAC7	QT interval	rs76285358	A	G	0.033	-0.376	0.027	2E-45	200.285	A	G	-0.169	0.371	0.650
HDAC7	QT interval	rs76632275	A	G	0.028	0.325	0.058	2E-08	31.371	A	G	0.489	0.336	0.150
HDAC7	QT interval	rs77190323	T	C	0.018	-0.277	0.034	7E-16	65.274	T	C	0.451	0.454	0.320
HDAC7	QT interval	rs77290820	A	G	0.042	0.162	0.022	1E-13	54.609	A	G	0.164	0.263	0.530
HDAC7	QT interval	rs77316493	T	A	0.043	0.187	0.022	2E-17	72.662	T	A	0.234	0.234	0.320
HDAC7	QT interval	rs77616825	C	A	0.015	0.289	0.051	2E-08	31.900	C	A	0.554	0.753	0.460
HDAC7	QT interval	rs77841370	T	C	0.018	-0.268	0.033	3E-16	66.583	T	C	0.557	0.499	0.260
HDAC7	QT interval	rs78079190	A	G	0.038	0.198	0.021	2E-21	90.589	A	G	-0.153	0.294	0.600
HDAC7	QT interval	rs78643454	A	G	0.060	0.166	0.018	4E-21	88.955	A	G	-0.095	0.213	0.650
HDAC7	QT interval	rs78698597	C	T	0.016	0.537	0.088	1E-09	37.095	C	T	-0.023	0.592	0.970
HDAC7	QT interval	rs78953588	C	T	0.022	0.240	0.033	3E-13	52.972	C	T	-0.419	0.352	0.230
HDAC7	QT interval	rs79171818	A	G	0.027	0.211	0.028	4E-14	57.350	A	G	-0.447	0.358	0.210
HDAC7	QT interval	rs79459945	G	T	0.224	-0.091	0.011	6E-17	70.107	G	T	-0.042	0.123	0.730
HDAC7	QT interval	rs7959280	G	A	0.310	0.067	0.011	5E-10	38.713	G	A	-0.345	0.125	0.006
HDAC7	QT interval	rs7960701	A	T	0.103	-0.335	0.031	9E-27	114.755	A	T	0.078	0.164	0.630
HDAC7	QT interval	rs7960745	A	C	0.367	0.131	0.008	2E-57	255.442	A	C	0.113	0.106	0.280
HDAC7	QT interval	rs7961288	A	G	0.076	-0.128	0.017	7E-15	60.475	A	G	-0.234	0.219	0.280
HDAC7	QT interval	rs7963444	G	C	0.294	0.055	0.009	2E-10	40.310	G	C	0.043	0.110	0.700
HDAC7	QT interval	rs7972177	G	A	0.299	-0.130	0.009	6E-51	225.323	G	A	-0.092	0.116	0.430
HDAC7	QT interval	rs7975232	C	A	0.475	0.136	0.009	7E-56	248.171	C	A	0.199	0.102	0.050
HDAC7	QT interval	rs7975604	T	C	0.055	-0.115	0.018	6E-11	42.769	T	C	0.101	0.202	0.620
HDAC7	QT interval	rs7979072	T	G	0.059	-0.102	0.017	6E-09	33.999	T	G	0.110	0.228	0.630
HDAC7	QT interval	rs79891674	C	T	0.021	-0.265	0.032	8E-17	69.416	C	T	-0.318	0.382	0.400

HDAC7	QT interval	rs79924760	G	A	0.035	0.138	0.025	2E-08	31.083	G	A	0.043	0.281	0.880
HDAC7	QT interval	rs80009138	G	A	0.023	-0.496	0.064	1E-14	59.496	G	A	0.015	0.399	0.970
HDAC7	QT interval	rs80083409	A	C	0.038	0.319	0.056	1E-08	32.863	A	C	-0.047	0.271	0.860
HDAC7	QT interval	rs832741	T	G	0.369	-0.309	0.019	1E-57	256.159	T	G	-0.102	0.105	0.330
HDAC7	QT interval	rs854890	A	G	0.134	0.184	0.028	5E-11	43.281	A	G	0.130	0.146	0.370
HDAC7	QT interval	rs854897	T	C	0.134	0.239	0.031	2E-14	58.135	T	C	-0.220	0.162	0.180
HDAC7	QT interval	rs855135	T	A	0.193	0.074	0.013	3E-09	35.096	T	A	0.124	0.134	0.350
HDAC7	QT interval	rs855137	T	C	0.201	-0.186	0.024	4E-15	61.547	T	C	0.024	0.128	0.850
HDAC7	QT interval	rs855186	C	T	0.021	0.630	0.075	3E-17	71.095	C	T	0.757	0.379	0.046
HDAC7	QT interval	rs929270	C	T	0.248	0.100	0.009	2E-27	117.523	C	T	-0.002	0.121	0.980
HDAC7	QT interval	rs9645810	T	C	0.039	-0.196	0.022	2E-19	80.950	T	C	0.374	0.310	0.230
HDAC9	QT interval	rs112695986	A	G	0.143	-0.088	0.011	2E-14	58.627	A	G	-0.120	0.154	0.440
HDAC9	QT interval	rs117912312	G	A	0.018	-0.203	0.032	2E-10	40.198	G	A	-0.075	0.376	0.840
HDAC9	QT interval	rs12699929	G	C	0.531	-0.052	0.009	2E-09	36.426	G	C	-0.109	0.103	0.290
HDAC9	QT interval	rs2691614	C	T	0.127	0.124	0.012	2E-25	108.797	C	T	0.046	0.150	0.760
HDAC9	QT interval	rs2714872	C	T	0.351	0.054	0.008	1E-10	41.536	C	T	-0.079	0.105	0.450
HDAC9	QT interval	rs624811	A	G	0.251	0.069	0.011	7E-10	38.118	A	G	-0.122	0.115	0.290
HDAC9	QT interval	rs78686616	G	A	0.025	0.149	0.027	2E-08	31.443	G	A	0.103	0.353	0.770
HDAC10	Serum phosphate levels	rs1010282	A	G	0.160	0.132	0.013	1E-23	100.225	A	G	0.002	0.003	0.450
HDAC10	Serum phosphate levels	rs10483249	A	G	0.036	0.224	0.022	4E-24	102.670	A	G	0.004	0.005	0.370
HDAC10	Serum phosphate levels	rs10854870	T	C	0.083	0.083	0.014	1E-08	32.513	T	C	0.005	0.004	0.180
HDAC10	Serum phosphate levels	rs111392589	T	C	0.041	-0.261	0.036	4E-13	52.867	T	C	-0.009	0.005	0.055
HDAC10	Serum phosphate levels	rs11547731	T	C	0.355	0.093	0.013	4E-12	48.008	T	C	0.000	0.002	0.960

HDAC10	Serum phosphate levels	rs11553142	T	C	0.037	-0.454	0.051	1E-18	78.145	T	C	-0.006	0.005	0.160
HDAC10	Serum phosphate levels	rs117000576	A	G	0.033	-0.141	0.024	4E-09	34.843	A	G	0.001	0.006	0.950
HDAC10	Serum phosphate levels	rs117265627	T	C	0.025	-0.196	0.028	6E-12	47.362	T	C	0.006	0.006	0.270
HDAC10	Serum phosphate levels	rs117291362	A	G	0.017	-0.332	0.043	1E-14	59.561	A	G	-0.004	0.009	0.640
HDAC10	Serum phosphate levels	rs117450593	A	G	0.033	-0.341	0.025	2E-42	186.109	A	G	0.008	0.005	0.085
HDAC10	Serum phosphate levels	rs117550993	T	C	0.026	0.269	0.027	3E-23	98.398	T	C	-0.008	0.007	0.210
HDAC10	Serum phosphate levels	rs117613664	T	C	0.024	-0.232	0.036	2E-10	40.927	T	C	0.004	0.006	0.590
HDAC10	Serum phosphate levels	rs117759723	C	T	0.016	0.624	0.065	1E-21	91.593	C	T	-0.017	0.010	0.110
HDAC10	Serum phosphate levels	rs11913406	C	T	0.195	0.130	0.010	3E-37	162.767	C	T	0.002	0.003	0.560
HDAC10	Serum phosphate levels	rs12158007	C	T	0.028	0.250	0.027	1E-19	82.676	C	T	0.004	0.008	0.490
HDAC10	Serum phosphate levels	rs12484907	A	G	0.044	-0.188	0.020	1E-21	91.280	A	G	-0.003	0.005	0.800
HDAC10	Serum phosphate levels	rs138270	T	G	0.497	-0.188	0.008	4E-122	552.474	T	G	-0.001	0.002	0.690
HDAC10	Serum phosphate levels	rs138812	A	G	0.071	0.112	0.016	1E-12	50.560	A	G	0.004	0.004	0.510
HDAC10	Serum phosphate levels	rs138877	G	A	0.062	-0.173	0.019	3E-19	80.694	G	A	-0.001	0.004	0.710
HDAC10	Serum phosphate levels	rs147595191	C	T	0.012	0.657	0.077	1E-17	73.113	C	T	-0.013	0.012	0.240
HDAC10	Serum phosphate levels	rs148864222	T	C	0.015	-0.911	0.120	3E-14	57.842	T	C	-0.007	0.043	0.870
HDAC10	Serum phosphate levels	rs17001272	C	T	0.117	0.096	0.015	7E-11	42.573	C	T	0.003	0.003	0.400
HDAC10	Serum phosphate levels	rs181700711	T	G	0.027	0.412	0.069	3E-09	35.303	T	G	-0.003	0.014	0.640
HDAC10	Serum phosphate levels	rs184639990	C	T	0.016	0.393	0.043	3E-20	84.839	C	T	0.001	0.011	0.920
HDAC10	Serum phosphate levels	rs2076139	T	C	0.231	0.072	0.011	1E-10	41.608	T	C	-0.003	0.003	0.340
HDAC10	Serum phosphate levels	rs2294398	A	G	0.026	-0.353	0.052	2E-11	45.325	A	G	-0.011	0.059	0.610
HDAC10	Serum phosphate levels	rs28372448	A	G	0.055	0.199	0.033	1E-09	36.828	A	G	-0.009	0.005	0.038

HDAC10	Serum phosphate levels	rs28479153	T	C	0.023	0.219	0.035	6E-10	38.373	T	C	0.007	0.006	0.270
HDAC10	Serum phosphate levels	rs28513473	C	T	0.096	0.097	0.015	6E-11	42.725	C	T	-0.004	0.004	0.300
HDAC10	Serum phosphate levels	rs28604596	G	T	0.306	0.102	0.013	7E-16	65.157	G	T	-0.003	0.003	0.370
HDAC10	Serum phosphate levels	rs34296882	T	C	0.158	-0.176	0.011	3E-57	254.383	T	C	-0.001	0.003	0.530
HDAC10	Serum phosphate levels	rs34355047	T	C	0.174	-0.105	0.016	2E-11	45.341	T	C	0.001	0.003	0.640
HDAC10	Serum phosphate levels	rs34402301	T	C	0.049	-0.138	0.024	1E-08	32.101	T	C	-0.008	0.004	0.043
HDAC10	Serum phosphate levels	rs34455105	A	G	0.053	-0.109	0.019	6E-09	33.712	A	G	0.003	0.005	0.570
HDAC10	Serum phosphate levels	rs34617177	T	C	0.022	0.245	0.038	1E-10	41.050	T	C	0.004	0.007	0.650
HDAC10	Serum phosphate levels	rs35381394	A	G	0.085	0.134	0.018	4E-14	57.411	A	G	-0.006	0.003	0.130
HDAC10	Serum phosphate levels	rs35792846	G	A	0.023	-0.305	0.043	9E-13	51.134	G	A	0.002	0.006	0.730
HDAC10	Serum phosphate levels	rs4823954	A	G	0.064	0.123	0.018	5E-12	47.745	A	G	-0.001	0.004	0.870
HDAC10	Serum phosphate levels	rs4824055	T	C	0.310	0.068	0.010	8E-12	46.851	T	C	0.002	0.002	0.390
HDAC10	Serum phosphate levels	rs4824056	G	A	0.170	0.192	0.011	6E-73	326.374	G	A	0.004	0.003	0.350
HDAC10	Serum phosphate levels	rs4838866	A	G	0.142	-0.185	0.012	4E-58	258.244	A	G	0.005	0.003	0.062
HDAC10	Serum phosphate levels	rs55747387	A	G	0.025	0.198	0.034	4E-09	34.598	A	G	-0.017	0.007	0.006
HDAC10	Serum phosphate levels	rs55899818	T	C	0.221	-0.110	0.011	3E-22	94.020	T	C	0.003	0.003	0.160
HDAC10	Serum phosphate levels	rs56139719	T	C	0.043	-0.244	0.034	4E-13	52.571	T	C	0.002	0.005	0.710
HDAC10	Serum phosphate levels	rs5771133	G	A	0.151	0.109	0.014	5E-14	56.575	G	A	-0.001	0.003	0.450
HDAC10	Serum phosphate levels	rs5771204	C	T	0.432	0.048	0.009	5E-08	29.722	C	T	-0.002	0.002	0.270
HDAC10	Serum phosphate levels	rs5771238	G	A	0.081	-0.120	0.017	3E-12	48.537	G	A	-0.006	0.004	0.081
HDAC10	Serum phosphate levels	rs5771271	A	G	0.119	0.132	0.014	4E-20	84.636	A	G	-0.007	0.003	0.035
HDAC10	Serum phosphate levels	rs58422561	A	G	0.029	0.518	0.055	3E-21	89.461	A	G	0.018	0.012	0.100
HDAC10	Serum phosphate levels	rs59650998	A	C	0.061	-0.157	0.018	2E-18	77.060	A	C	0.011	0.004	0.010

HDAC10	Serum phosphate levels	rs61760587	A	G	0.046	0.176	0.025	9E-13	51.108	A	G	0.002	0.005	0.750
HDAC10	Serum phosphate levels	rs73429531	T	C	0.021	-0.244	0.031	5E-15	61.466	T	C	-0.001	0.007	0.870
HDAC10	Serum phosphate levels	rs73445937	A	G	0.033	0.293	0.037	9E-16	64.574	A	G	0.011	0.006	0.035
HDAC10	Serum phosphate levels	rs74624037	T	C	0.017	-0.237	0.039	8E-10	37.668	T	C	0.011	0.007	0.083
HDAC10	Serum phosphate levels	rs75080456	T	C	0.070	-0.172	0.021	3E-16	66.932	T	C	0.009	0.004	0.007
HDAC10	Serum phosphate levels	rs75596977	C	T	0.019	-0.233	0.042	4E-08	30.378	C	T	0.002	0.012	0.960
HDAC10	Serum phosphate levels	rs79431035	T	C	0.027	0.343	0.047	3E-13	53.147	T	C	0.020	0.035	0.570
HDAC10	Serum phosphate levels	rs79947213	G	T	0.032	-0.200	0.031	1E-10	41.486	G	T	0.008	0.007	0.320
HDAC10	Serum phosphate levels	rs79966207	C	T	0.150	-0.091	0.015	8E-10	37.813	C	T	-0.004	0.003	0.130
HDAC10	Serum phosphate levels	rs80045995	T	C	0.024	-0.273	0.042	6E-11	42.845	T	C	0.003	0.006	0.630
HDAC10	Serum phosphate levels	rs8137793	A	G	0.456	0.081	0.009	1E-17	73.459	A	G	0.000	0.002	0.860
HDAC10	Serum phosphate levels	rs916362	A	G	0.406	0.287	0.009	1E-200	1093.036	A	G	-0.002	0.002	0.470
HDAC10	Serum phosphate levels	rs9616730	G	A	0.215	-0.083	0.011	3E-14	57.961	G	A	-0.001	0.003	0.660
HDAC10	Serum phosphate levels	rs9617098	A	G	0.032	-0.345	0.034	5E-24	102.030	A	G	-0.002	0.005	0.560
HDAC10	Serum phosphate levels	rs9628289	T	C	0.037	-0.220	0.022	1E-22	95.949	T	C	-0.013	0.006	0.017
HDAC10	QT interval	rs1010282	A	G	0.160	0.132	0.013	1E-23	100.225	A	G	-0.088	0.198	0.660
HDAC10	QT interval	rs10483249	A	G	0.036	0.224	0.022	4E-24	102.670	A	G	-0.107	0.291	0.710
HDAC10	QT interval	rs10854870	T	C	0.083	0.083	0.014	1E-08	32.513	T	C	-0.002	0.200	0.990
HDAC10	QT interval	rs111375925	T	C	0.016	0.270	0.037	2E-13	53.629	T	C	-0.251	0.648	0.700
HDAC10	QT interval	rs111392589	T	C	0.041	-0.261	0.036	4E-13	52.867	T	C	-0.314	0.353	0.370
HDAC10	QT interval	rs111569990	T	C	0.143	0.127	0.013	1E-23	100.480	T	C	-0.087	0.196	0.660
HDAC10	QT interval	rs112189132	T	C	0.059	-0.145	0.023	6E-10	38.235	T	C	-0.047	0.298	0.870

HDAC10	QT interval	rs113389610	G	A	0.036	0.699	0.023	1E-197	899.858	G	A	-0.153	0.523	0.770
HDAC10	QT interval	rs113491012	C	A	0.051	0.573	0.025	1E-115	522.800	C	A	-0.648	0.964	0.500
HDAC10	QT interval	rs113924912	T	C	0.167	-0.201	0.017	4E-32	139.405	T	C	0.234	0.214	0.270
HDAC10	QT interval	rs11547731	T	C	0.355	0.093	0.013	4E-12	48.008	T	C	0.078	0.167	0.640
HDAC10	QT interval	rs11553142	T	C	0.037	-0.454	0.051	1E-18	78.145	T	C	-0.126	0.406	0.760
HDAC10	QT interval	rs116972251	T	C	0.014	0.562	0.098	9E-09	32.961	T	C	0.098	1.890	0.960
HDAC10	QT interval	rs117000576	A	G	0.033	-0.141	0.024	4E-09	34.843	A	G	0.368	0.365	0.310
HDAC10	QT interval	rs11703673	T	C	0.306	0.119	0.010	6E-33	142.865	T	C	-0.187	0.151	0.220
HDAC10	QT interval	rs11703986	A	G	0.180	0.077	0.011	5E-13	52.030	A	G	-0.119	0.166	0.470
HDAC10	QT interval	rs11704019	T	C	0.052	0.282	0.033	6E-18	74.487	T	C	0.062	0.286	0.830
HDAC10	QT interval	rs11704368	A	G	0.073	0.131	0.016	3E-17	71.099	A	G	0.107	0.214	0.620
HDAC10	QT interval	rs117265627	T	C	0.025	-0.196	0.028	6E-12	47.362	T	C	0.316	0.484	0.510
HDAC10	QT interval	rs117291362	A	G	0.017	-0.332	0.043	1E-14	59.561	A	G	0.460	0.429	0.280
HDAC10	QT interval	rs117309460	G	A	0.012	0.921	0.131	2E-12	49.618	G	A	1.430	1.080	0.190
HDAC10	QT interval	rs117439977	A	G	0.063	-0.361	0.018	2E-88	397.674	A	G	-0.394	0.311	0.210
HDAC10	QT interval	rs117450593	A	G	0.033	-0.341	0.025	2E-42	186.109	A	G	-0.412	0.353	0.240
HDAC10	QT interval	rs117550993	T	C	0.026	0.269	0.027	3E-23	98.398	T	C	0.137	0.448	0.760
HDAC10	QT interval	rs117563943	A	C	0.042	0.170	0.023	6E-14	56.343	A	C	0.235	0.312	0.450
HDAC10	QT interval	rs117613664	T	C	0.024	-0.232	0.036	2E-10	40.927	T	C	0.502	0.537	0.350
HDAC10	QT interval	rs117616637	C	T	0.024	0.369	0.036	3E-24	103.344	C	T	-1.070	1.690	0.530
HDAC10	QT interval	rs117759723	C	T	0.016	0.624	0.065	1E-21	91.593	C	T	-1.450	0.593	0.015

HDAC10	QT interval	rs117826045	T	G	0.016	0.259	0.041	3E-10	39.499	T	G	0.131	1.560	0.930
HDAC10	QT interval	rs117899254	A	G	0.015	0.368	0.041	3E-19	80.691	A	G	-0.576	0.562	0.310
HDAC10	QT interval	rs117957229	G	A	0.024	0.224	0.034	4E-11	43.391	G	A	-0.069	2.010	0.970
HDAC10	QT interval	rs117994696	G	T	0.029	-0.241	0.026	5E-21	88.638	G	T	0.347	0.468	0.460
HDAC10	QT interval	rs118116626	T	C	0.016	0.542	0.087	4E-10	39.085	T	C	0.515	2.000	0.800
HDAC10	QT interval	rs11913406	C	T	0.195	0.130	0.010	3E-37	162.767	C	T	0.071	0.133	0.590
HDAC10	QT interval	rs11913414	G	T	0.119	0.184	0.014	7E-42	183.955	G	T	-0.086	0.192	0.650
HDAC10	QT interval	rs12158007	C	T	0.028	0.250	0.027	1E-19	82.676	C	T	0.480	0.362	0.180
HDAC10	QT interval	rs12484907	A	G	0.044	-0.188	0.020	1E-21	91.280	A	G	0.227	0.256	0.380
HDAC10	QT interval	rs12485055	A	C	0.101	-0.185	0.022	2E-17	72.118	A	C	-0.021	0.270	0.940
HDAC10	QT interval	rs13056136	T	C	0.050	-0.167	0.020	9E-17	69.282	T	C	0.338	0.274	0.220
HDAC10	QT interval	rs13058164	G	A	0.237	0.313	0.011	5E-169	768.011	G	A	-0.048	0.189	0.800
HDAC10	QT interval	rs134453	A	C	0.332	-0.074	0.008	2E-18	76.710	A	C	-0.079	0.109	0.470
HDAC10	QT interval	rs134472	C	T	0.460	0.099	0.014	4E-13	52.803	C	T	0.069	0.116	0.550
HDAC10	QT interval	rs134473	T	C	0.106	0.102	0.014	2E-12	49.951	T	C	-0.237	0.213	0.270
HDAC10	QT interval	rs135856	C	T	0.106	0.385	0.013	2E-200	912.937	C	T	0.139	0.164	0.400
HDAC10	QT interval	rs137868	T	C	0.135	-0.199	0.015	1E-38	168.612	T	C	0.012	0.248	0.960
HDAC10	QT interval	rs137881	A	G	0.021	-0.237	0.040	3E-09	35.054	A	G	0.782	1.170	0.500
HDAC10	QT interval	rs137898	T	C	0.302	0.097	0.013	1E-13	54.780	T	C	0.225	0.275	0.410
HDAC10	QT interval	rs137899	A	G	0.284	-0.087	0.013	6E-12	47.408	A	G	-0.342	0.287	0.230
HDAC10	QT interval	rs137902	A	G	0.138	0.166	0.014	4E-32	139.196	A	G	0.065	0.233	0.780

HDAC10	QT interval	rs13811	C	T	0.283	0.252	0.016	3E-54	240.443	C	T	-0.628	0.200	0.002
HDAC10	QT interval	rs138221	G	T	0.484	0.090	0.009	2E-24	104.179	G	T	-0.079	0.102	0.440
HDAC10	QT interval	rs138231	G	A	0.015	-0.695	0.086	9E-16	64.696	G	A	-0.203	2.230	0.930
HDAC10	QT interval	rs138270	T	G	0.497	-0.188	0.008	4E-122	552.474	T	G	0.204	0.107	0.057
HDAC10	QT interval	rs138273	A	G	0.088	-0.401	0.014	1E-178	812.304	A	G	-0.245	0.236	0.300
HDAC10	QT interval	rs138652799	T	C	0.024	0.814	0.117	3E-12	48.774	T	C	2.740	2.890	0.340
HDAC10	QT interval	rs138812	A	G	0.071	0.112	0.016	1E-12	50.560	A	G	-0.157	0.200	0.430
HDAC10	QT interval	rs138877	G	A	0.062	-0.173	0.019	3E-19	80.694	G	A	0.056	0.198	0.780
HDAC10	QT interval	rs138998389	C	T	0.018	0.298	0.052	1E-08	32.851	C	T	0.700	2.330	0.760
HDAC10	QT interval	rs139527301	T	C	0.019	0.385	0.059	7E-11	42.644	T	C	-1.920	1.950	0.320
HDAC10	QT interval	rs139784	A	G	0.256	0.073	0.010	6E-13	51.993	A	G	-0.043	0.116	0.710
HDAC10	QT interval	rs139793	T	G	0.026	0.604	0.026	1E-122	554.498	T	G	-0.454	0.314	0.150
HDAC10	QT interval	rs139818	C	T	0.099	0.132	0.015	2E-19	81.334	C	T	-0.101	0.205	0.620
HDAC10	QT interval	rs139823	G	A	0.228	0.305	0.010	3E-191	870.348	G	A	-0.147	0.174	0.400
HDAC10	QT interval	rs141499951	C	T	0.018	0.570	0.074	2E-14	58.804	C	T	0.642	0.684	0.350
HDAC10	QT interval	rs141709018	G	A	0.019	0.270	0.048	2E-08	31.641	G	A	-2.340	1.760	0.180
HDAC10	QT interval	rs143657796	T	C	0.023	0.277	0.043	8E-11	42.302	T	C	-0.407	1.860	0.830
HDAC10	QT interval	rs144345128	A	C	0.012	0.268	0.047	9E-09	32.957	A	C	0.666	0.658	0.310
HDAC10	QT interval	rs145474292	G	A	0.013	-0.413	0.066	5E-10	38.868	G	A	1.060	0.779	0.170
HDAC10	QT interval	rs145537167	T	C	0.019	-0.312	0.057	3E-08	30.468	T	C	2.040	1.990	0.310

HDAC10	QT interval	rs147363989	A	G	0.024	0.190	0.032	2E-09	35.787	A	G	-0.670	0.534	0.210
HDAC10	QT interval	rs147595191	C	T	0.012	0.657	0.077	1E-17	73.113	C	T	-1.360	0.725	0.061
HDAC10	QT interval	rs147906724	T	C	0.015	0.248	0.039	3E-10	39.594	T	C	3.030	2.580	0.240
HDAC10	QT interval	rs148208096	T	C	0.028	0.516	0.025	2E-93	420.703	T	C	-0.183	0.411	0.660
HDAC10	QT interval	rs148740866	C	A	0.015	-0.930	0.139	3E-11	44.433	C	A	0.071	0.977	0.940
HDAC10	QT interval	rs149798187	A	G	0.029	-0.392	0.030	4E-39	171.019	A	G	-0.856	1.280	0.500
HDAC10	QT interval	rs149986667	C	T	0.115	-0.159	0.014	1E-30	132.793	C	T	0.059	0.180	0.750
HDAC10	QT interval	rs150309031	T	C	0.026	-0.237	0.035	1E-11	46.112	T	C	0.584	0.336	0.082
HDAC10	QT interval	rs150366244	G	A	0.046	-0.713	0.021	1E-200	1151.118	G	A	-0.082	0.442	0.850
HDAC10	QT interval	rs150755090	T	C	0.032	-0.435	0.041	7E-26	110.717	T	C	0.011	2.210	1.000
HDAC10	QT interval	rs150770587	A	G	0.012	-0.934	0.166	2E-08	31.667	A	G	-2.230	3.150	0.480
HDAC10	QT interval	rs151182861	A	G	0.026	0.252	0.029	5E-18	74.940	A	G	0.234	0.507	0.640
HDAC10	QT interval	rs151201116	G	A	0.015	0.314	0.040	2E-15	62.685	G	A	-0.370	0.538	0.490
HDAC10	QT interval	rs17001272	C	T	0.117	0.096	0.015	7E-11	42.573	C	T	0.001	0.197	1.000
HDAC10	QT interval	rs181700711	T	G	0.027	0.412	0.069	3E-09	35.303	T	G	0.617	0.858	0.470
HDAC10	QT interval	rs182846491	A	G	0.016	1.013	0.150	1E-11	45.933	A	G	-1.820	0.902	0.043
HDAC10	QT interval	rs184639990	C	T	0.016	0.393	0.043	3E-20	84.839	C	T	-0.623	0.712	0.380
HDAC10	QT interval	rs185086762	C	T	0.014	-1.023	0.161	2E-10	40.394	C	T	5.860	3.330	0.079
HDAC10	QT interval	rs186429433	A	G	0.017	-0.960	0.090	2E-26	113.116	A	G	-3.880	3.200	0.220
HDAC10	QT interval	rs189268966	T	C	0.018	0.365	0.033	6E-29	124.649	T	C	0.268	0.384	0.490
HDAC10	QT interval	rs190115008	T	C	0.015	-1.119	0.160	3E-12	48.687	T	C	0.194	0.710	0.780
HDAC10	QT interval	rs190735878	C	A	0.018	0.481	0.086	2E-08	31.125	C	A	0.139	4.910	0.980

HDAC10	QT interval	rs192059871	G	A	0.021	-0.251	0.035	7E-13	51.633	G	A	-0.217	0.551	0.690
HDAC10	QT interval	rs192890491	G	A	0.018	-0.422	0.057	1E-13	55.247	G	A	0.049	1.970	0.980
HDAC10	QT interval	rs1967476	T	C	0.030	0.471	0.026	3E-71	318.887	T	C	0.203	0.572	0.720
HDAC10	QT interval	rs2076136	A	G	0.156	0.067	0.011	2E-09	36.041	A	G	0.142	0.180	0.430
HDAC10	QT interval	rs2076139	T	C	0.231	0.072	0.011	1E-10	41.608	T	C	-0.003	0.172	0.980
HDAC10	QT interval	rs2142537	G	C	0.215	0.101	0.017	1E-09	36.925	G	C	-0.183	0.124	0.140
HDAC10	QT interval	rs2235356	G	A	0.533	0.080	0.012	1E-10	41.745	G	A	-0.093	0.147	0.530
HDAC10	QT interval	rs2272852	A	G	0.028	-0.244	0.025	2E-22	94.630	A	G	0.370	0.611	0.540
HDAC10	QT interval	rs2294379	C	T	0.386	0.107	0.011	5E-23	97.595	C	T	0.063	0.138	0.650
HDAC10	QT interval	rs28372448	A	G	0.055	0.199	0.033	1E-09	36.828	A	G	-0.209	0.804	0.800
HDAC10	QT interval	rs28404597	A	G	0.223	0.109	0.012	8E-20	83.065	A	G	-0.081	0.171	0.640
HDAC10	QT interval	rs28442189	G	C	0.426	-0.124	0.010	8E-33	142.449	G	C	-0.138	0.148	0.350
HDAC10	QT interval	rs28449609	G	A	0.082	0.248	0.022	2E-29	127.405	G	A	0.116	0.262	0.660
HDAC10	QT interval	rs28479153	T	C	0.023	0.219	0.035	6E-10	38.373	T	C	0.965	0.548	0.079
HDAC10	QT interval	rs28513473	C	T	0.096	0.097	0.015	6E-11	42.725	C	T	0.253	0.222	0.250
HDAC10	QT interval	rs28516848	G	T	0.046	-0.158	0.028	2E-08	32.038	G	T	0.276	0.355	0.440
HDAC10	QT interval	rs28534659	G	A	0.027	-0.266	0.030	2E-18	77.032	G	A	0.310	0.333	0.350
HDAC10	QT interval	rs28535173	G	T	0.241	0.097	0.014	4E-12	48.166	G	T	-0.014	0.155	0.930
HDAC10	QT interval	rs28546641	T	C	0.142	0.257	0.013	4E-94	423.619	T	C	0.123	0.166	0.460
HDAC10	QT interval	rs28604596	G	T	0.306	0.102	0.013	7E-16	65.157	G	T	0.032	0.263	0.900
HDAC10	QT interval	rs28634968	A	G	0.118	0.237	0.016	5E-47	207.469	A	G	0.065	0.205	0.750
HDAC10	QT interval	rs28711487	C	T	0.272	0.193	0.014	4E-43	189.607	C	T	0.070	0.148	0.640
HDAC10	QT interval	rs28722130	G	C	0.053	0.410	0.022	2E-77	347.330	G	C	0.040	0.274	0.880

HDAC10	QT interval	rs28753402	G	A	0.451	-0.255	0.014	5E-79	354.442	G	A	-1.240	0.969	0.200
HDAC10	QT interval	rs34296882	T	C	0.158	-0.176	0.011	3E-57	254.383	T	C	0.139	0.141	0.320
HDAC10	QT interval	rs34353679	T	C	0.022	-0.491	0.041	2E-33	145.371	T	C	1.130	0.465	0.015
HDAC10	QT interval	rs34355047	T	C	0.174	-0.105	0.016	2E-11	45.341	T	C	0.095	0.446	0.830
HDAC10	QT interval	rs34394484	A	G	0.041	0.390	0.029	3E-41	180.907	A	G	0.228	0.425	0.590
HDAC10	QT interval	rs34402301	T	C	0.049	-0.138	0.024	1E-08	32.101	T	C	-0.393	0.396	0.320
HDAC10	QT interval	rs34455105	A	G	0.053	-0.109	0.019	6E-09	33.712	A	G	0.562	0.287	0.051
HDAC10	QT interval	rs34617177	T	C	0.022	0.245	0.038	1E-10	41.050	T	C	-1.690	1.040	0.110
HDAC10	QT interval	rs35304733	A	G	0.061	-0.103	0.017	1E-09	37.076	A	G	0.056	0.225	0.800
HDAC10	QT interval	rs35381394	A	G	0.085	0.134	0.018	4E-14	57.411	A	G	-0.178	0.250	0.480
HDAC10	QT interval	rs35792846	G	A	0.023	-0.305	0.043	9E-13	51.134	G	A	1.420	1.230	0.250
HDAC10	QT interval	rs36053713	A	G	0.083	-0.178	0.018	4E-23	97.990	A	G	0.389	0.246	0.110
HDAC10	QT interval	rs3901193	C	T	0.160	-0.176	0.019	6E-21	88.089	C	T	0.179	0.286	0.530
HDAC10	QT interval	rs4084289	A	G	0.248	-0.279	0.010	7E-186	845.392	A	G	-0.011	0.150	0.940
HDAC10	QT interval	rs4823954	A	G	0.064	0.123	0.018	5E-12	47.745	A	G	-0.319	0.263	0.230
HDAC10	QT interval	rs4823962	C	T	0.067	-0.267	0.016	8E-62	275.263	C	T	0.336	0.211	0.110
HDAC10	QT interval	rs4824048	T	G	0.060	-0.132	0.020	2E-11	44.550	T	G	-0.288	0.338	0.390
HDAC10	QT interval	rs4824053	T	C	0.055	0.192	0.018	4E-27	116.148	T	C	0.260	0.220	0.240
HDAC10	QT interval	rs4824055	T	C	0.310	0.068	0.010	8E-12	46.851	T	C	0.186	0.132	0.160
HDAC10	QT interval	rs4824056	G	A	0.170	0.192	0.011	6E-73	326.374	G	A	0.190	0.158	0.230
HDAC10	QT interval	rs4838818	A	G	0.134	0.340	0.014	1E-123	558.947	A	G	-0.355	0.237	0.130

HDAC10	QT interval	rs4838866	A	G	0.142	-0.185	0.012	4E-58	258.244	A	G	-0.062	0.207	0.760
HDAC10	QT interval	rs55747387	A	G	0.025	0.198	0.034	4E-09	34.598	A	G	0.600	0.601	0.320
HDAC10	QT interval	rs55899818	T	C	0.221	-0.110	0.011	3E-22	94.020	T	C	0.097	0.177	0.580
HDAC10	QT interval	rs56139719	T	C	0.043	-0.244	0.034	4E-13	52.571	T	C	-0.410	0.270	0.130
HDAC10	QT interval	rs56150947	A	G	0.464	-0.375	0.012	1E-200	985.915	A	G	0.459	0.176	0.009
HDAC10	QT interval	rs57258598	A	G	0.030	0.222	0.031	1E-12	50.791	A	G	0.043	0.818	0.960
HDAC10	QT interval	rs5770741	A	G	0.215	0.200	0.010	2E-92	416.226	A	G	0.092	0.124	0.460
HDAC10	QT interval	rs5771116	T	A	0.215	0.091	0.013	2E-12	49.573	T	A	-0.083	0.257	0.750
HDAC10	QT interval	rs5771133	G	A	0.151	0.109	0.014	5E-14	56.575	G	A	0.293	0.185	0.110
HDAC10	QT interval	rs5771190	A	G	0.167	0.114	0.012	1E-21	91.624	A	G	-0.060	0.138	0.660
HDAC10	QT interval	rs5771204	C	T	0.432	0.048	0.009	5E-08	29.722	C	T	-0.155	0.133	0.240
HDAC10	QT interval	rs5771209	G	A	0.270	-0.133	0.009	2E-47	209.184	G	A	0.032	0.145	0.820
HDAC10	QT interval	rs5771215	A	C	0.351	-0.090	0.010	5E-21	88.383	A	C	0.059	0.152	0.700
HDAC10	QT interval	rs5771238	G	A	0.081	-0.120	0.017	3E-12	48.537	G	A	0.555	0.287	0.053
HDAC10	QT interval	rs5771245	T	C	0.423	-0.133	0.011	2E-35	154.838	T	C	-0.139	0.177	0.430
HDAC10	QT interval	rs5771271	A	G	0.119	0.132	0.014	4E-20	84.636	A	G	0.228	0.260	0.380
HDAC10	QT interval	rs58422561	A	G	0.029	0.518	0.055	3E-21	89.461	A	G	-0.463	0.416	0.260
HDAC10	QT interval	rs59650998	A	C	0.061	-0.157	0.018	2E-18	77.060	A	C	0.537	0.301	0.074
HDAC10	QT interval	rs6009816	C	T	0.159	-0.110	0.011	2E-23	99.464	C	T	0.166	0.154	0.280
HDAC10	QT interval	rs6009905	C	T	0.030	0.300	0.025	5E-32	138.650	C	T	-0.054	0.278	0.850
HDAC10	QT interval	rs6010161	A	G	0.246	0.073	0.010	6E-13	51.944	A	G	-0.161	0.188	0.390
HDAC10	QT interval	rs6010167	G	A	0.439	-0.121	0.008	7E-50	220.647	G	A	0.136	0.103	0.180

HDAC10	QT interval	rs6010195	G	T	0.142	-0.116	0.014	4E-16	66.198	G	T	0.254	0.190	0.180
HDAC10	QT interval	rs6010197	A	G	0.438	-0.071	0.010	2E-13	54.488	A	G	0.248	0.187	0.180
HDAC10	QT interval	rs60332599	G	A	0.165	-0.250	0.016	1E-54	242.088	G	A	0.662	0.242	0.006
HDAC10	QT interval	rs61760587	A	G	0.046	0.176	0.025	9E-13	51.108	A	G	-0.049	0.354	0.890
HDAC10	QT interval	rs62231924	T	G	0.119	-0.184	0.019	2E-22	94.561	T	G	-0.574	0.470	0.220
HDAC10	QT interval	rs62232138	A	G	0.067	0.261	0.018	3E-48	213.019	A	G	0.447	0.240	0.062
HDAC10	QT interval	rs62239290	T	C	0.044	0.182	0.023	2E-15	63.126	T	C	-0.460	0.372	0.220
HDAC10	QT interval	rs62241209	A	G	0.133	-0.088	0.013	9E-12	46.540	A	G	-0.203	0.187	0.280
HDAC10	QT interval	rs62241220	A	G	0.437	-0.281	0.010	1E-166	756.570	A	G	-0.134	0.128	0.300
HDAC10	QT interval	rs66473461	C	T	0.087	-0.120	0.017	2E-12	50.042	C	T	0.208	0.214	0.330
HDAC10	QT interval	rs728028	T	C	0.340	0.083	0.014	8E-09	33.270	T	C	0.155	0.136	0.260
HDAC10	QT interval	rs7286615	C	G	0.226	-0.082	0.015	3E-08	30.994	C	G	0.409	0.164	0.013
HDAC10	QT interval	rs7291218	C	T	0.037	-0.128	0.021	3E-09	35.414	C	T	0.054	0.273	0.840
HDAC10	QT interval	rs7291940	G	A	0.113	0.293	0.016	3E-77	346.101	G	A	0.197	0.246	0.420
HDAC10	QT interval	rs73176905	C	T	0.033	0.253	0.039	1E-10	41.045	C	T	-0.257	0.487	0.600
HDAC10	QT interval	rs73176983	C	T	0.029	-0.274	0.025	1E-27	118.601	C	T	-0.182	0.374	0.630
HDAC10	QT interval	rs73176996	A	G	0.044	-0.246	0.035	4E-12	48.358	A	G	0.092	0.339	0.790
HDAC10	QT interval	rs73179122	T	C	0.111	-0.223	0.023	8E-23	96.668	T	C	-0.263	0.233	0.260
HDAC10	QT interval	rs73179148	A	G	0.142	-0.186	0.012	5E-58	258.002	A	G	-0.208	0.159	0.190
HDAC10	QT interval	rs73181105	A	G	0.016	-0.240	0.038	4E-10	38.993	A	G	-1.020	0.507	0.044
HDAC10	QT interval	rs73181132	A	G	0.028	1.046	0.162	1E-10	41.892	A	G	0.392	0.952	0.680
HDAC10	QT interval	rs73181183	A	G	0.047	-0.198	0.029	5E-12	47.820	A	G	-0.473	0.403	0.240

HDAC10	QT interval	rs73183392	C	T	0.025	0.292	0.040	2E-13	54.441	C	T	-0.169	0.600	0.780
HDAC10	QT interval	rs73187269	T	C	0.135	-0.193	0.013	6E-49	216.162	T	C	0.127	0.233	0.580
HDAC10	QT interval	rs73429531	T	C	0.021	-0.244	0.031	5E-15	61.466	T	C	0.176	0.459	0.700
HDAC10	QT interval	rs73445933	T	C	0.043	0.177	0.028	1E-10	41.196	T	C	-0.245	0.292	0.400
HDAC10	QT interval	rs73445937	A	G	0.033	0.293	0.037	9E-16	64.574	A	G	-0.212	0.454	0.640
HDAC10	QT interval	rs738418	C	G	0.165	0.208	0.011	1E-81	366.176	C	G	-0.007	0.145	0.960
HDAC10	QT interval	rs73890863	T	C	0.018	-0.460	0.057	9E-16	64.555	T	C	-0.034	0.342	0.920
HDAC10	QT interval	rs74492663	G	A	0.027	-0.202	0.025	2E-15	63.342	G	A	-0.528	0.353	0.130
HDAC10	QT interval	rs74624037	T	C	0.017	-0.237	0.039	8E-10	37.668	T	C	-0.173	0.540	0.750
HDAC10	QT interval	rs74854156	C	T	0.032	-0.221	0.034	4E-11	43.428	C	T	-0.466	0.440	0.290
HDAC10	QT interval	rs75080456	T	C	0.070	-0.172	0.021	3E-16	66.932	T	C	0.651	0.306	0.033
HDAC10	QT interval	rs75440836	T	C	0.044	-0.294	0.020	4E-50	221.718	T	C	-0.035	0.241	0.880
HDAC10	QT interval	rs75596977	C	T	0.019	-0.233	0.042	4E-08	30.378	C	T	-0.062	0.547	0.910
HDAC10	QT interval	rs75649964	A	C	0.084	0.188	0.017	1E-28	123.348	A	C	0.061	0.320	0.850
HDAC10	QT interval	rs760748	A	G	0.025	-0.301	0.052	6E-09	33.749	A	G	1.240	2.860	0.660
HDAC10	QT interval	rs763126	T	G	0.393	0.259	0.008	1E-200	1047.269	T	G	0.132	0.108	0.220
HDAC10	QT interval	rs77078751	A	G	0.045	0.214	0.035	7E-10	38.052	A	G	-0.354	0.276	0.200
HDAC10	QT interval	rs77371730	T	C	0.021	0.217	0.033	5E-11	43.028	T	C	-0.543	0.353	0.120
HDAC10	QT interval	rs77458731	G	A	0.053	0.170	0.031	2E-08	31.090	G	A	-0.509	0.238	0.033
HDAC10	QT interval	rs78212605	T	C	0.016	-0.239	0.042	1E-08	32.235	T	C	0.383	1.490	0.800
HDAC10	QT interval	rs79016563	A	G	0.051	0.252	0.024	3E-26	112.191	A	G	0.641	0.613	0.300
HDAC10	QT interval	rs79142910	T	C	0.015	0.472	0.079	2E-09	35.981	T	C	0.872	0.715	0.220

HDAC10	QT interval	rs79250573	A	G	0.045	-0.122	0.020	1E-09	36.736	A	G	0.400	0.326	0.220
HDAC10	QT interval	rs79947213	G	T	0.032	-0.200	0.031	1E-10	41.486	G	T	-0.817	0.466	0.080
HDAC10	QT interval	rs79966207	C	T	0.150	-0.091	0.015	8E-10	37.813	C	T	0.454	0.242	0.061
HDAC10	QT interval	rs79998483	A	G	0.124	0.130	0.014	1E-19	82.005	A	G	0.048	0.213	0.820
HDAC10	QT interval	rs80045995	T	C	0.024	-0.273	0.042	6E-11	42.845	T	C	0.966	0.456	0.034
HDAC10	QT interval	rs80101768	T	C	0.034	-0.262	0.031	1E-17	73.082	T	C	0.312	0.391	0.430
HDAC10	QT interval	rs8135472	G	A	0.489	0.126	0.011	1E-32	141.710	G	A	-0.079	0.166	0.630
HDAC10	QT interval	rs8137793	A	G	0.456	0.081	0.009	1E-17	73.459	A	G	0.011	0.122	0.930
HDAC10	QT interval	rs8184965	A	G	0.401	-0.081	0.011	2E-14	58.381	A	G	0.030	0.154	0.850
HDAC10	QT interval	rs909691	A	G	0.517	0.100	0.012	1E-16	68.347	A	G	-0.181	0.153	0.230
HDAC10	QT interval	rs916362	A	G	0.406	0.287	0.009	1E-200	1093.036	A	G	0.087	0.115	0.450
HDAC10	QT interval	rs9616695	T	C	0.035	-0.175	0.031	2E-08	31.659	T	C	0.450	0.456	0.320
HDAC10	QT interval	rs9616730	G	A	0.215	-0.083	0.011	3E-14	57.961	G	A	-0.098	0.130	0.450
HDAC10	QT interval	rs9616740	A	G	0.158	0.292	0.012	3E-131	594.478	A	G	-0.188	0.324	0.560
HDAC10	QT interval	rs9616760	G	A	0.313	0.128	0.016	1E-15	64.311	G	A	0.017	0.177	0.920
HDAC10	QT interval	rs9617098	A	G	0.032	-0.345	0.034	5E-24	102.030	A	G	-0.337	0.466	0.470
HDAC10	QT interval	rs9617145	C	T	0.141	0.131	0.012	8E-30	128.613	C	T	0.322	0.146	0.028
HDAC10	QT interval	rs9628275	T	C	0.345	-0.108	0.012	1E-19	82.442	T	C	0.189	0.169	0.260
HDAC10	QT interval	rs9628289	T	C	0.037	-0.220	0.022	1E-22	95.949	T	C	-0.559	0.305	0.067
HDAC10	QT interval	rs9628299	G	C	0.249	-0.250	0.009	4E-159	722.243	G	C	0.081	0.126	0.520
HDAC10	QT interval	rs9628319	T	C	0.122	-0.113	0.015	8E-15	60.457	T	C	0.189	0.213	0.380

HDAC11	Serum phosphate levels	rs168628	A	C	0.043	0.165	0.023	3E-13	53.077	A	C	-0.007	0.005	0.150
HDAC11	Serum phosphate levels	rs2276747	A	G	0.201	0.056	0.010	1E-08	32.178	A	G	0.003	0.003	0.480
HDAC11	Serum phosphate levels	rs360845	A	G	0.367	0.088	0.011	2E-16	67.352	A	G	-0.003	0.002	0.200
HDAC11	Serum phosphate levels	rs360846	C	T	0.062	0.117	0.018	1E-10	41.616	C	T	0.010	0.004	0.024
HDAC11	Serum phosphate levels	rs4684909	T	C	0.342	-0.046	0.008	3E-08	30.532	T	C	0.000	0.002	0.760
HDAC11	Serum phosphate levels	rs62234567	T	G	0.307	-0.189	0.021	3E-20	85.222	T	G	-0.003	0.002	0.310
HDAC11	Serum phosphate levels	rs6442373	C	T	0.129	0.188	0.012	9E-55	243.030	C	T	0.004	0.003	0.180
HDAC11	Serum phosphate levels	rs73014518	T	C	0.216	-0.204	0.010	8E-86	385.500	T	C	-0.001	0.003	0.780
HDAC11	Serum phosphate levels	rs73018516	T	C	0.074	-0.094	0.016	6E-09	33.921	T	C	0.000	0.004	0.980
HDAC11	Serum phosphate levels	rs73033243	G	A	0.038	-0.200	0.022	2E-20	85.926	G	A	0.003	0.005	0.520
HDAC11	Serum phosphate levels	rs74858247	G	A	0.040	-0.168	0.022	4E-14	56.975	G	A	0.006	0.005	0.220
HDAC11	Serum phosphate levels	rs7616054	A	G	0.128	0.160	0.013	2E-35	154.486	A	G	-0.002	0.003	0.400
HDAC11	Serum phosphate levels	rs76427117	C	T	0.041	0.199	0.021	1E-21	90.947	C	T	-0.001	0.005	0.860
HDAC11	Serum phosphate levels	rs79324993	G	A	0.044	0.150	0.020	2E-14	58.897	G	A	0.000	0.005	0.820
HDAC11	QT interval	rs113941744	A	G	0.040	0.134	0.021	5E-10	38.730	A	G	0.304	0.279	0.270
HDAC11	QT interval	rs114480627	T	C	0.012	-0.340	0.054	4E-10	38.995	T	C	0.150	0.620	0.810
HDAC11	QT interval	rs114666678	A	C	0.033	0.143	0.023	8E-10	37.773	A	C	0.463	0.319	0.150
HDAC11	QT interval	rs115258020	T	C	0.017	0.256	0.041	3E-10	39.496	T	C	0.796	0.659	0.230
HDAC11	QT interval	rs116718459	A	C	0.018	-0.293	0.032	2E-20	85.911	A	C	0.333	0.406	0.410
HDAC11	QT interval	rs11707778	G	A	0.231	-0.398	0.023	7E-69	307.589	G	A	0.260	0.125	0.037
HDAC11	QT interval	rs11712537	T	C	0.071	0.130	0.016	3E-16	66.680	T	C	0.286	0.236	0.230
HDAC11	QT interval	rs11721176	A	C	0.119	-0.086	0.013	6E-12	47.268	A	C	-0.027	0.172	0.870
HDAC11	QT interval	rs11921282	T	C	0.359	0.072	0.009	7E-16	65.002	T	C	-0.046	0.125	0.720

HDAC11	QT interval	rs12495241	A	G	0.058	-0.106	0.019	3E-08	30.440	A	G	-0.133	0.284	0.640
HDAC11	QT interval	rs12634816	G	A	0.309	0.136	0.021	1E-10	41.448	G	A	-0.098	0.110	0.370
HDAC11	QT interval	rs13065759	T	C	0.038	0.241	0.023	2E-25	108.698	T	C	-0.155	0.283	0.580
HDAC11	QT interval	rs13069140	A	G	0.108	-0.262	0.031	7E-17	69.606	A	G	0.330	0.169	0.051
HDAC11	QT interval	rs13084488	G	A	0.068	0.110	0.018	2E-09	36.053	G	A	-0.189	0.228	0.410
HDAC11	QT interval	rs13097641	G	A	0.345	0.056	0.008	3E-11	44.481	G	A	-0.075	0.109	0.490
HDAC11	QT interval	rs138789714	A	C	0.024	-0.186	0.033	3E-08	31.007	A	C	0.304	0.483	0.530
HDAC11	QT interval	rs140420201	T	C	0.013	0.763	0.135	1E-08	32.088	T	C	0.345	0.692	0.620
HDAC11	QT interval	rs1433359	A	G	0.499	0.196	0.019	1E-23	100.846	A	G	-0.090	0.104	0.390
HDAC11	QT interval	rs146064643	C	T	0.130	0.128	0.012	7E-25	106.182	C	T	0.107	0.183	0.560
HDAC11	QT interval	rs146547665	T	C	0.025	-0.162	0.027	2E-09	35.610	T	C	0.527	0.412	0.200
HDAC11	QT interval	rs168628	A	C	0.043	0.165	0.023	3E-13	53.077	A	C	-0.096	0.303	0.750
HDAC11	QT interval	rs180897511	A	G	0.016	0.253	0.043	5E-09	34.108	A	G	0.338	2.520	0.890
HDAC11	QT interval	rs1816427	T	C	0.081	-0.253	0.035	4E-13	52.792	T	C	-0.093	0.212	0.660
HDAC11	QT interval	rs185773055	C	T	0.022	0.292	0.041	7E-13	51.538	C	T	0.757	0.496	0.130
HDAC11	QT interval	rs2101448	T	C	0.281	0.204	0.022	5E-21	88.732	T	C	-0.030	0.118	0.800
HDAC11	QT interval	rs2276747	A	G	0.201	0.056	0.010	1E-08	32.178	A	G	-0.003	0.131	0.980
HDAC11	QT interval	rs2449077	T	C	0.501	-0.299	0.008	1E-200	1262.604	T	C	0.083	0.114	0.470
HDAC11	QT interval	rs2569996	C	T	0.232	-0.061	0.011	1E-08	32.499	C	T	0.111	0.155	0.470
HDAC11	QT interval	rs2596896	T	G	0.328	-0.084	0.010	9E-19	78.195	T	G	0.048	0.134	0.720
HDAC11	QT interval	rs2597506	G	A	0.487	-0.118	0.008	6E-50	220.856	G	A	-0.051	0.103	0.620
HDAC11	QT interval	rs2655209	T	C	0.018	0.197	0.033	2E-09	35.942	T	C	-0.237	0.611	0.700

HDAC11	QT interval	rs2733168	T	C	0.164	0.064	0.011	3E-09	35.345	T	C	-0.084	0.131	0.530
HDAC11	QT interval	rs357158	T	C	0.415	-0.182	0.009	6E-97	436.565	T	C	-0.041	0.107	0.710
HDAC11	QT interval	rs35824618	A	G	0.022	-0.222	0.031	1E-12	50.385	A	G	0.134	0.444	0.760
HDAC11	QT interval	rs360746	G	C	0.234	0.059	0.011	5E-08	29.820	G	C	-0.003	0.145	0.980
HDAC11	QT interval	rs360829	A	G	0.333	-0.089	0.010	1E-19	81.950	A	G	0.076	0.133	0.570
HDAC11	QT interval	rs360833	A	G	0.253	0.145	0.011	2E-43	190.664	A	G	0.099	0.138	0.480
HDAC11	QT interval	rs360843	A	G	0.442	0.091	0.011	4E-17	70.724	A	G	0.047	0.126	0.710
HDAC11	QT interval	rs360844	G	A	0.100	0.131	0.014	7E-20	83.215	G	A	-0.145	0.184	0.430
HDAC11	QT interval	rs360845	A	G	0.367	0.088	0.011	2E-16	67.352	A	G	-0.124	0.128	0.330
HDAC11	QT interval	rs360846	C	T	0.062	0.117	0.018	1E-10	41.616	C	T	0.228	0.242	0.350
HDAC11	QT interval	rs4478060	T	C	0.434	0.087	0.008	6E-27	115.670	T	C	-0.151	0.107	0.160
HDAC11	QT interval	rs458459	T	C	0.242	0.092	0.011	3E-17	71.153	T	C	0.142	0.143	0.320
HDAC11	QT interval	rs462825	C	G	0.272	0.060	0.009	2E-11	44.767	C	G	-0.214	0.123	0.081
HDAC11	QT interval	rs468279	C	G	0.378	-0.086	0.008	1E-25	109.818	C	G	-0.064	0.105	0.540
HDAC11	QT interval	rs4684151	A	G	0.075	-0.104	0.015	4E-12	48.241	A	G	-0.058	0.174	0.740
HDAC11	QT interval	rs4684157	A	G	0.288	0.056	0.009	2E-10	40.605	A	G	-0.065	0.114	0.570
HDAC11	QT interval	rs4684909	T	C	0.342	-0.046	0.008	3E-08	30.532	T	C	-0.010	0.121	0.940
HDAC11	QT interval	rs4684974	T	C	0.203	0.061	0.010	6E-10	38.338	T	C	0.129	0.130	0.320
HDAC11	QT interval	rs522491	G	A	0.277	0.159	0.010	8E-62	275.387	G	A	-0.070	0.131	0.590
HDAC11	QT interval	rs557593	C	T	0.315	0.047	0.009	3E-08	30.727	C	T	-0.266	0.108	0.014
HDAC11	QT interval	rs55809373	T	C	0.175	-0.124	0.010	3E-32	140.097	T	C	0.153	0.138	0.270
HDAC11	QT interval	rs56144878	T	C	0.014	-0.341	0.039	4E-18	75.135	T	C	0.233	0.500	0.640
HDAC11	QT interval	rs56347393	C	T	0.382	-0.188	0.019	5E-22	92.948	C	T	-0.036	0.107	0.730

HDAC11	QT interval	rs56925963	T	C	0.090	-0.187	0.033	2E-08	31.789	T	C	-0.078	0.175	0.660
HDAC11	QT interval	rs59799759	C	T	0.024	0.177	0.027	4E-11	43.447	C	T	-0.080	0.329	0.810
HDAC11	QT interval	rs62234567	T	G	0.307	-0.189	0.021	3E-20	85.222	T	G	0.062	0.109	0.570
HDAC11	QT interval	rs6442373	C	T	0.129	0.188	0.012	9E-55	243.030	C	T	0.127	0.167	0.450
HDAC11	QT interval	rs6442417	T	C	0.272	-0.323	0.022	2E-50	223.002	T	C	0.161	0.122	0.180
HDAC11	QT interval	rs67111631	C	T	0.146	0.103	0.011	1E-19	81.815	C	T	-0.008	0.178	0.960
HDAC11	QT interval	rs71306030	T	C	0.022	0.287	0.034	2E-17	71.711	T	C	-0.420	0.447	0.350
HDAC11	QT interval	rs724442	C	G	0.152	0.101	0.012	3E-17	71.670	C	G	-0.082	0.147	0.580
HDAC11	QT interval	rs73014518	T	C	0.216	-0.204	0.010	8E-86	385.500	T	C	0.024	0.153	0.880
HDAC11	QT interval	rs73017526	G	C	0.024	0.167	0.027	4E-10	39.245	G	C	0.469	0.357	0.190
HDAC11	QT interval	rs73018516	T	C	0.074	-0.094	0.016	6E-09	33.921	T	C	-0.067	0.217	0.760
HDAC11	QT interval	rs73018875	A	G	0.020	0.543	0.067	4E-16	66.279	A	G	0.444	0.563	0.430
HDAC11	QT interval	rs73028763	C	T	0.071	-0.182	0.018	1E-23	100.762	C	T	-0.220	0.224	0.330
HDAC11	QT interval	rs73033212	G	T	0.120	0.077	0.013	1E-09	37.359	G	T	-0.052	0.180	0.770
HDAC11	QT interval	rs73033243	G	A	0.038	-0.200	0.022	2E-20	85.926	G	A	0.141	0.268	0.600
HDAC11	QT interval	rs73033955	T	C	0.037	-0.166	0.024	2E-12	49.455	T	C	-0.089	0.405	0.830
HDAC11	QT interval	rs73813518	A	C	0.063	-0.110	0.017	7E-11	42.442	A	C	0.309	0.221	0.160
HDAC11	QT interval	rs74782020	T	C	0.139	0.074	0.012	1E-10	41.173	T	C	-0.109	0.164	0.510
HDAC11	QT interval	rs74858247	G	A	0.040	-0.168	0.022	4E-14	56.975	G	A	0.172	0.301	0.570
HDAC11	QT interval	rs75262025	T	C	0.081	0.203	0.018	2E-29	126.964	T	C	-0.043	0.209	0.840
HDAC11	QT interval	rs75741112	T	C	0.051	-0.124	0.018	2E-11	45.301	T	C	0.272	0.275	0.320
HDAC11	QT interval	rs7611704	A	G	0.174	-0.063	0.010	2E-09	36.053	A	G	0.198	0.147	0.180
HDAC11	QT interval	rs7616054	A	G	0.128	0.160	0.013	2E-35	154.486	A	G	-0.290	0.165	0.079

HDAC11	QT interval	rs7630488	C	A	0.466	-0.609	0.045	1E-42	186.979	C	A	-0.156	0.104	0.130
HDAC11	QT interval	rs7632509	T	A	0.318	-0.085	0.009	1E-22	96.185	T	A	0.134	0.109	0.220
HDAC11	QT interval	rs7634734	A	G	0.066	0.194	0.017	5E-29	124.916	A	G	-0.009	0.197	0.960
HDAC11	QT interval	rs76427117	C	T	0.041	0.199	0.021	1E-21	90.947	C	T	0.186	0.316	0.560
HDAC11	QT interval	rs7647863	G	A	0.084	-0.147	0.014	1E-24	105.183	G	A	0.001	0.173	0.990
HDAC11	QT interval	rs77474099	A	G	0.149	-0.070	0.011	1E-09	37.283	A	G	0.068	0.144	0.640
HDAC11	QT interval	rs78514578	A	G	0.016	0.359	0.059	1E-09	37.261	A	G	-0.688	0.503	0.170
HDAC11	QT interval	rs79324993	G	A	0.044	0.150	0.020	2E-14	58.897	G	A	0.149	0.255	0.560
HDAC11	QT interval	rs80260618	T	C	0.045	-0.278	0.019	8E-47	206.530	T	C	0.389	0.244	0.110
HDAC11	QT interval	rs900087	G	A	0.042	0.184	0.020	2E-20	85.629	G	A	0.138	0.281	0.620
HDAC11	QT interval	rs923981	C	T	0.340	0.067	0.008	3E-15	62.189	C	T	-0.059	0.109	0.590
HDAC11	QT interval	rs9310431	G	T	0.302	-0.129	0.009	3E-50	222.052	G	T	0.080	0.111	0.470
HDAC11	QT interval	rs9813525	T	C	0.339	-0.095	0.008	2E-29	127.220	T	C	0.035	0.117	0.770
HDAC11	QT interval	rs9819997	G	A	0.423	-0.083	0.009	2E-21	90.246	G	A	0.020	0.124	0.870
HDAC11	QT interval	rs9823655	T	G	0.478	-0.046	0.008	5E-09	34.108	T	G	0.067	0.103	0.520
HDAC11	QT interval	rs9825306	A	G	0.086	0.096	0.014	2E-11	44.877	A	G	-0.280	0.180	0.120
HDAC11	QT interval	rs9840696	A	G	0.376	0.058	0.008	2E-12	49.514	A	G	-0.057	0.109	0.600
HDAC11	QT interval	rs9870638	T	G	0.170	-0.265	0.026	5E-24	102.212	T	G	-0.086	0.130	0.510

SNPs, single nucleotide polymorphisms; EA-E, effect allele of exposure; NEA-E, non-effect allele of exposure; EAF, effect allele frequency; Beta-E, beta of exposure; SE-E, standard error of exposure; *P*-E, the significance level of SNPs in exposure; EA-O, effect allele of outcome; NEA-O, non-effect allele of outcome; Beta-O, beta of outcome; SE-O, standard error of outcome; *P*-O, the significance level of SNPs in outcome. The *F* statistic for each SNP was calculated as follows: $F = Beta^2 exposure/SE^2 exposure$.

In total, 1,381 SNPs were included in the study. For serum phosphate levels, 248 SNPs were selected, distributed as follows: HDAC1 (20), HDAC3 (35), HDAC4 (55), HDAC5 (10), HDAC7 (49), HDAC10 (65), and HDAC11 (14). For QT interval, 1,133 SNPs were included: HDAC1 (93), HDAC2 (10), HDAC3 (142), HDAC4 (226), HDAC5 (69), HDAC7 (269), HDAC9 (7), HDAC10 (222), and HDAC11 (95).
