# Supplementary material for: Hypophosphatemia as a Potential Class Effect of Histone Deacetylase Inhibitors: Evidence from Disproportionality Analysis and Mendelian Randomization Analysis of Drug Targets
Source: Pharmaceuticals (Basel). 2026 Apr 28;19(5):689. doi: 10.3390/ph19050689 (PMC13209236; doi:10.3390/ph19050689)
Supplement: Supplementary file 1 [file pharmaceuticals-19-00689-s001.zip › Table S3.pdf]

Table S3. Reported cases and signal strengths of HDACi at the SOC level.

Drug	SOC name	Cases	ROR (95%CI)	PRR (χ^2)	IC (IC025)	EBGM (EBGM05)
Vorinostat	Investigations	714	2.39 (2.21 - 2.59)	2.2 (500.16)	1.14 (-0.53)	2.2 (2.06)
Vorinostat	Gastrointestinal Disorders	612	1.39 (1.28 - 1.51)	1.35 (59.55)	0.43 (-1.24)	1.35 (1.25)
Vorinostat	General Disorders And Administration Site Conditions	592	0.59 (0.54 - 0.64)	0.63 (152.69)	-0.66 (-2.33)	0.63 (0.59)
Vorinostat	Infections And Infestations	528	1.98 (1.81 - 2.17)	1.88 (231.43)	0.91 (-0.75)	1.88 (1.75)
Vorinostat	Blood And Lymphatic System Disorders	471	5.71 (5.19 - 6.28)	5.29 (1667.12)	2.4 (0.74)	5.29 (4.89)
Vorinostat	Respiratory, Thoracic And Mediastinal Disorders	380	1.56 (1.4 - 1.73)	1.52 (70.43)	0.6 (-1.06)	1.52 (1.39)
Vorinostat	Metabolism And Nutrition Disorders	379	3.46 (3.12 - 3.85)	3.29 (617.05)	1.72 (0.05)	3.29 (3.01)
Vorinostat	Nervous System Disorders	297	0.64 (0.57 - 0.72)	0.66 (58.05)	-0.61 (-2.27)	0.66 (0.6)
Vorinostat	Injury, Poisoning And Procedural Complications	236	0.4 (0.36 - 0.46)	0.43 (197.62)	-1.21 (-2.88)	0.43 (0.39)
Vorinostat	Cardiac Disorders	184	1.35 (1.16 - 1.56)	1.34 (15.98)	0.42 (-1.25)	1.34 (1.18)
Vorinostat	Neoplasms Benign, Malignant And Unspecified (Incl Cysts And Polyps)	181	1.26 (1.09 - 1.46)	1.25 (9.57)	0.33 (-1.34)	1.25 (1.11)
Vorinostat	Vascular Disorders	150	1.33 (1.13 - 1.56)	1.32 (11.84)	0.4 (-1.27)	1.32 (1.15)
Vorinostat	Renal And Urinary Disorders	136	1.33 (1.13 - 1.58)	1.33 (11.11)	0.41 (-1.26)	1.33 (1.15)
Vorinostat	Skin And Subcutaneous Tissue Disorders	133	0.45 (0.38 - 0.53)	0.46 (88.08)	-1.11 (-2.78)	0.46 (0.4)
Vorinostat	Musculoskeletal And Connective Tissue Disorders	102	0.35 (0.29 - 0.43)	0.36 (120.71)	-1.46 (-3.13)	0.36 (0.31)
Vorinostat	Psychiatric Disorders	87	0.28 (0.22 - 0.34)	0.29 (162.65)	-1.8 (-3.46)	0.29 (0.24)
Vorinostat	Hepatobiliary Disorders	47	0.98 (0.74 - 1.31)	0.99 (0.01)	-0.02 (-1.69)	0.99 (0.77)
Vorinostat	Surgical And Medical Procedures	34	0.48 (0.34 - 0.67)	0.48 (19.09)	-1.05 (-2.72)	0.48 (0.36)
Vorinostat	Eye Disorders	17	0.16 (0.1 - 0.26)	0.16 (75.48)	-2.63 (-4.3)	0.16 (0.11)
Vorinostat	Immune System Disorders	16	0.27 (0.16 - 0.44)	0.27 (31.79)	-1.89 (-3.55)	0.27 (0.18)
Vorinostat	Reproductive System And Breast Disorders	13	0.27 (0.16 - 0.47)	0.27 (25.14)	-1.86 (-3.53)	0.27 (0.17)
Vorinostat	Ear And Labyrinth Disorders	7	0.3 (0.14 - 0.63)	0.3 (11.39)	-1.73 (-3.4)	0.3 (0.16)
Vorinostat	Product Issues	6	0.07 (0.03 - 0.16)	0.07 (74.14)	-3.82 (-5.48)	0.07 (0.04)
Vorinostat	Social Circumstances	6	0.25 (0.11 - 0.55)	0.25 (13.91)	-2.02 (-3.69)	0.25 (0.13)
Vorinostat	Endocrine Disorders	4	0.3 (0.11 - 0.79)	0.3 (6.7)	-1.76 (-3.42)	0.3 (0.13)
Vorinostat	Congenital, Familial And Genetic Disorders	1	0.06 (0.01 - 0.42)	0.06 (14.91)	-4.07 (-5.74)	0.06 (0.01)
Romidepsin	General Disorders And Administration Site Conditions	476	0.84 (0.76 - 0.93)	0.86 (12.46)	-0.21 (-1.88)	0.86 (0.8)
Romidepsin	Investigations	349	2.02 (1.8 - 2.25)	1.9 (158.82)	0.93 (-0.74)	1.9 (1.73)
Romidepsin	Blood And Lymphatic System Disorders	324	6.93 (6.17 - 7.77)	6.31 (1471.66)	2.66 (0.99)	6.31 (5.73)
Romidepsin	Gastrointestinal Disorders	315	1.22 (1.08 - 1.37)	1.19 (10.85)	0.26 (-1.41)	1.19 (1.08)

Romidepsin	Neoplasms Benign, Malignant And Unspecified (Incl Cysts And Polyps)	310	3.89 (3.46 - 4.38)	3.6 (599.66)	1.85 (0.18)	3.6 (3.27)
Romidepsin	Infections And Infestations	257	1.61 (1.42 - 1.83)	1.56 (54.31)	0.64 (-1.03)	1.56 (1.4)
Romidepsin	Cardiac Disorders	175	2.36 (2.03 - 2.75)	2.29 (130.05)	1.19 (-0.47)	2.29 (2.01)
Romidepsin	Metabolism And Nutrition Disorders	149	2.33 (1.97 - 2.74)	2.26 (107.45)	1.18 (-0.49)	2.26 (1.97)
Romidepsin	Nervous System Disorders	140	0.52 (0.44 - 0.61)	0.54 (60.39)	-0.89 (-2.56)	0.54 (0.47)
Romidepsin	Respiratory, Thoracic And Mediastinal Disorders	132	0.9 (0.76 - 1.08)	0.91 (1.29)	-0.14 (-1.81)	0.91 (0.78)
Romidepsin	Injury, Poisoning And Procedural Complications	95	0.27 (0.22 - 0.33)	0.29 (185.53)	-1.79 (-3.46)	0.29 (0.24)
Romidepsin	Skin And Subcutaneous Tissue Disorders	93	0.54 (0.44 - 0.66)	0.55 (36.41)	-0.86 (-2.53)	0.55 (0.46)
Romidepsin	Vascular Disorders	49	0.75 (0.56 - 0.99)	0.75 (4.12)	-0.41 (-2.08)	0.75 (0.59)
Romidepsin	Hepatobiliary Disorders	47	1.74 (1.31 - 2.32)	1.73 (14.6)	0.79 (-0.88)	1.73 (1.36)
Romidepsin	Renal And Urinary Disorders	40	0.67 (0.49 - 0.91)	0.67 (6.63)	-0.58 (-2.24)	0.67 (0.52)
Romidepsin	Musculoskeletal And Connective Tissue Disorders	39	0.23 (0.17 - 0.31)	0.24 (101.73)	-2.08 (-3.75)	0.24 (0.18)
Romidepsin	Immune System Disorders	38	1.09 (0.79 - 1.5)	1.09 (0.28)	0.12 (-1.54)	1.09 (0.83)
Romidepsin	Psychiatric Disorders	29	0.16 (0.11 - 0.23)	0.17 (127.98)	-2.58 (-4.25)	0.17 (0.12)
Romidepsin	Eye Disorders	27	0.44 (0.3 - 0.64)	0.44 (19.51)	-1.18 (-2.85)	0.44 (0.32)
Romidepsin	Reproductive System And Breast Disorders	11	0.4 (0.22 - 0.72)	0.4 (9.91)	-1.32 (-2.98)	0.4 (0.24)
Romidepsin	Ear And Labyrinth Disorders	6	0.44 (0.2 - 0.98)	0.44 (4.23)	-1.18 (-2.84)	0.44 (0.23)
Romidepsin	Product Issues	4	0.08 (0.03 - 0.21)	0.08 (43.73)	-3.66 (-5.33)	0.08 (0.03)
Romidepsin	Surgical And Medical Procedures	3	0.07 (0.02 - 0.22)	0.07 (36.12)	-3.79 (-5.45)	0.07 (0.03)
Romidepsin	Social Circumstances	2	0.14 (0.04 - 0.57)	0.14 (10.41)	-2.82 (-4.48)	0.14 (0.04)
Romidepsin	Endocrine Disorders	1	0.13 (0.02 - 0.9)	0.13 (6.04)	-2.98 (-4.65)	0.13 (0.02)
Belinostat	General Disorders And Administration Site Conditions	221	1.05 (0.91 - 1.22)	1.04 (0.46)	0.06 (-1.61)	1.04 (0.92)
Belinostat	Investigations	162	2.56 (2.17 - 3.02)	2.35 (133.17)	1.23 (-0.44)	2.35 (2.04)
Belinostat	Gastrointestinal Disorders	150	1.6 (1.34 - 1.89)	1.52 (29.07)	0.6 (-1.06)	1.52 (1.32)
Belinostat	Metabolism And Nutrition Disorders	95	4.14 (3.36 - 5.11)	3.89 (208.28)	1.96 (0.29)	3.89 (3.26)
Belinostat	Infections And Infestations	73	1.16 (0.92 - 1.47)	1.15 (1.51)	0.2 (-1.47)	1.15 (0.94)
Belinostat	Respiratory, Thoracic And Mediastinal Disorders	73	1.36 (1.07 - 1.72)	1.34 (6.55)	0.42 (-1.25)	1.34 (1.1)
Belinostat	Nervous System Disorders	55	0.57 (0.43 - 0.74)	0.59 (17.5)	-0.77 (-2.44)	0.59 (0.47)
Belinostat	Blood And Lymphatic System Disorders	49	2.61 (1.96 - 3.48)	2.55 (46.83)	1.35 (-0.32)	2.55 (2.01)
Belinostat	Skin And Subcutaneous Tissue Disorders	46	0.68 (0.51 - 0.92)	0.7 (6.41)	-0.52 (-2.19)	0.7 (0.54)
Belinostat	Vascular Disorders	42	1.82 (1.34 - 2.47)	1.79 (14.9)	0.84 (-0.83)	1.79 (1.38)
Belinostat	Neoplasms Benign, Malignant And Unspecified (Incl Cysts And Polyps)	38	1.09 (0.79 - 1.51)	1.09 (0.3)	0.13 (-1.54)	1.09 (0.83)
Belinostat	Cardiac Disorders	36	1.38 (0.99 - 1.92)	1.37 (3.6)	0.45 (-1.22)	1.37 (1.03)

Belinostat	Musculoskeletal And Connective Tissue Disorders	34	0.53 (0.38 - 0.75)	0.55 (13.37)	-0.87 (-2.54)	0.55 (0.41)
Belinostat	Injury, Poisoning And Procedural Complications	34	0.23 (0.16 - 0.33)	0.25 (84.17)	-1.98 (-3.65)	0.25 (0.19)
Belinostat	Psychiatric Disorders	23	0.35 (0.23 - 0.52)	0.36 (27.64)	-1.47 (-3.14)	0.36 (0.25)
Belinostat	Renal And Urinary Disorders	20	0.87 (0.56 - 1.36)	0.88 (0.36)	-0.19 (-1.86)	0.88 (0.61)
Belinostat	Eye Disorders	7	0.3 (0.14 - 0.63)	0.3 (11.32)	-1.71 (-3.38)	0.3 (0.16)
Belinostat	Surgical And Medical Procedures	6	0.36 (0.16 - 0.81)	0.37 (6.65)	-1.45 (-3.11)	0.37 (0.19)
Belinostat	Immune System Disorders	5	0.36 (0.15 - 0.86)	0.36 (5.81)	-1.48 (-3.15)	0.36 (0.17)
Belinostat	Ear And Labyrinth Disorders	4	0.77 (0.29 - 2.06)	0.77 (0.27)	-0.37 (-2.04)	0.77 (0.34)
Belinostat	Hepatobiliary Disorders	4	0.42 (0.16 - 1.11)	0.42 (3.28)	-1.26 (-2.93)	0.42 (0.18)
Belinostat	Reproductive System And Breast Disorders	3	0.31 (0.1 - 0.96)	0.31 (4.63)	-1.69 (-3.36)	0.31 (0.12)
Belinostat	Product Issues	1	0.05 (0.01 - 0.34)	0.05 (18.74)	-4.35 (-6.02)	0.05 (0.01)
Belinostat	Endocrine Disorders	1	0.33 (0.05 - 2.34)	0.33 (1.36)	-1.6 (-3.27)	0.33 (0.06)
Belinostat	Social Circumstances	1	0.19 (0.03 - 1.36)	0.19 (3.42)	-2.38 (-4.05)	0.19 (0.04)
Panobinostat	Gastrointestinal Disorders	695	2.15 (1.98 - 2.33)	1.96 (356.21)	0.97 (-0.7)	1.96 (1.83)
Panobinostat	General Disorders And Administration Site Conditions	685	0.87 (0.81 - 0.95)	0.89 (10.35)	-0.16 (-1.83)	0.89 (0.84)
Panobinostat	Investigations	586	2.56 (2.35 - 2.8)	2.35 (482.11)	1.23 (-0.43)	2.35 (2.18)
Panobinostat	Neoplasms Benign, Malignant And Unspecified (Incl Cysts And Polyps)	348	2.85 (2.55 - 3.18)	2.7 (383.84)	1.43 (-0.23)	2.7 (2.46)
Panobinostat	Infections And Infestations	326	1.45 (1.3 - 1.63)	1.42 (42.48)	0.5 (-1.16)	1.42 (1.29)
Panobinostat	Blood And Lymphatic System Disorders	311	4.78 (4.26 - 5.36)	4.5 (860.91)	2.17 (0.5)	4.5 (4.09)
Panobinostat	Metabolism And Nutrition Disorders	227	2.7 (2.36 - 3.08)	2.6 (229.07)	1.38 (-0.29)	2.6 (2.33)
Panobinostat	Nervous System Disorders	223	0.65 (0.56 - 0.74)	0.66 (41.17)	-0.59 (-2.26)	0.66 (0.59)
Panobinostat	Respiratory, Thoracic And Mediastinal Disorders	178	0.91 (0.78 - 1.05)	0.91 (1.67)	-0.14 (-1.8)	0.91 (0.8)
Panobinostat	Injury, Poisoning And Procedural Complications	154	0.29 (0.24 - 0.34)	0.31 (263.14)	-1.68 (-3.34)	0.31 (0.27)
Panobinostat	Cardiac Disorders	126	1.38 (1.16 - 1.65)	1.37 (12.92)	0.46 (-1.21)	1.37 (1.18)
Panobinostat	Renal And Urinary Disorders	81	0.98 (0.78 - 1.22)	0.98 (0.04)	-0.03 (-1.7)	0.98 (0.81)
Panobinostat	Vascular Disorders	75	0.9 (0.72 - 1.13)	0.9 (0.78)	-0.15 (-1.81)	0.9 (0.75)
Panobinostat	Musculoskeletal And Connective Tissue Disorders	70	0.3 (0.24 - 0.38)	0.31 (110.1)	-1.67 (-3.33)	0.31 (0.26)
Panobinostat	Hepatobiliary Disorders	49	1.43 (1.08 - 1.9)	1.43 (6.38)	0.52 (-1.15)	1.43 (1.13)
Panobinostat	Psychiatric Disorders	44	0.18 (0.14 - 0.25)	0.19 (159.34)	-2.39 (-4.05)	0.19 (0.15)
Panobinostat	Skin And Subcutaneous Tissue Disorders	38	0.15 (0.11 - 0.21)	0.16 (180.75)	-2.66 (-4.33)	0.16 (0.12)
Panobinostat	Surgical And Medical Procedures	23	0.38 (0.26 - 0.58)	0.39 (22.51)	-1.37 (-3.03)	0.39 (0.28)
Panobinostat	Eye Disorders	14	0.17 (0.1 - 0.28)	0.17 (58.31)	-2.56 (-4.23)	0.17 (0.11)
Panobinostat	Immune System Disorders	7	0.14 (0.06 - 0.29)	0.14 (38.27)	-2.86 (-4.53)	0.14 (0.07)

Panobinostat	Endocrine Disorders	7	0.64 (0.3 - 1.34)	0.64 (1.44)	-0.65 (-2.31)	0.64 (0.34)
Panobinostat	Ear And Labyrinth Disorders	4	0.21 (0.08 - 0.57)	0.21 (11.65)	-2.23 (-3.9)	0.21 (0.09)
Panobinostat	Reproductive System And Breast Disorders	1	0.03 (0 - 0.21)	0.03 (31.94)	-5.07 (-6.74)	0.03 (0.01)

HDACi, histone deacetylase inhibitors; SOC, System Organ Class; ROR, reporting odds ratio; CI, confidence interval; PRR, proportional reporting ratio; χ^2, chi-squared; IC, information component; IC025, the lower limit of the 95% CI of the IC; EBGM, empirical Bayesian geometric mean; EBGM05, the lower limit of the 95% CI of EBGM.
