# Supplementary material for: Hypophosphatemia as a Potential Class Effect of Histone Deacetylase Inhibitors: Evidence from Disproportionality Analysis and Mendelian Randomization Analysis of Drug Targets
Source: Pharmaceuticals (Basel). 2026 Apr 28;19(5):689. doi: 10.3390/ph19050689 (PMC13209236; doi:10.3390/ph19050689)
Supplement: Supplementary file 1 [file pharmaceuticals-19-00689-s001.zip › Table S4.pdf]

Table S4. Reported cases and signal strengths of HDACi at the PT level.

Drug	PT name	Cases	ROR (95%CI)	PRR (χ^2)	IC (IC025)	EBGM (EBGM05)
Vorinostat	Febrile Neutropenia	163	30.58 (26.15 - 35.74)	29.67 (4506.49)	4.89 (3.22)	29.58 (25.96)
Vorinostat	Platelet Count Decreased	119	13.15 (10.96 - 15.77)	12.88 (1304.18)	3.68 (2.02)	12.86 (11.05)
Vorinostat	Nausea	118	1.75 (1.46 - 2.1)	1.74 (37.31)	0.8 (-0.87)	1.74 (1.49)
Vorinostat	Diarrhoea	103	1.89 (1.56 - 2.3)	1.88 (42.54)	0.91 (-0.76)	1.88 (1.59)
Vorinostat	Pneumonia	100	3.43 (2.81 - 4.18)	3.38 (168.64)	1.76 (0.09)	3.38 (2.87)
Vorinostat	Thrombocytopenia	96	10.39 (8.49 - 12.71)	10.22 (798.69)	3.35 (1.68)	10.21 (8.62)
Vorinostat	Fatigue	87	1.29 (1.05 - 1.6)	1.29 (5.66)	0.36 (-1.3)	1.29 (1.08)
Vorinostat	Dehydration	86	7.53 (6.09 - 9.32)	7.43 (478.89)	2.89 (1.23)	7.42 (6.21)
Vorinostat	Vomiting	86	2.18 (1.76 - 2.7)	2.16 (54.04)	1.11 (-0.55)	2.16 (1.81)
Vorinostat	Decreased Appetite	77	3.73 (2.98 - 4.68)	3.7 (151.91)	1.89 (0.22)	3.69 (3.06)
Vorinostat	Anaemia	76	4.57 (3.64 - 5.73)	4.52 (208.68)	2.17 (0.51)	4.52 (3.74)
Vorinostat	Pyrexia	73	2.44 (1.94 - 3.08)	2.42 (61.34)	1.28 (-0.39)	2.42 (2)
Vorinostat	White Blood Cell Count Decreased	60	6.43 (4.99 - 8.3)	6.37 (272.05)	2.67 (1)	6.37 (5.15)
Vorinostat	Sepsis	60	6.24 (4.84 - 8.05)	6.18 (260.91)	2.63 (0.96)	6.18 (4.99)
Vorinostat	Dyspnoea	54	1.1 (0.84 - 1.44)	1.1 (0.49)	0.14 (-1.53)	1.1 (0.88)
Vorinostat	Product Use In Unapproved Indication	50	2.7 (2.04 - 3.57)	2.69 (53.06)	1.42 (-0.24)	2.69 (2.13)
Vorinostat	Neutrophil Count Decreased	49	14.52 (10.96 - 19.24)	14.39 (610.19)	3.85 (2.18)	14.37 (11.36)
Vorinostat	Asthenia	48	1.49 (1.12 - 1.97)	1.48 (7.54)	0.57 (-1.1)	1.48 (1.17)
Vorinostat	Neutropenia	45	3.96 (2.95 - 5.31)	3.94 (98.74)	1.98 (0.31)	3.94 (3.08)
Vorinostat	Death	45	0.6 (0.45 - 0.81)	0.61 (11.69)	-0.72 (-2.39)	0.61 (0.47)
Vorinostat	Respiratory Failure	43	6.87 (5.09 - 9.28)	6.82 (213.86)	2.77 (1.1)	6.82 (5.31)
Vorinostat	Haemoglobin Decreased	43	4.82 (3.57 - 6.5)	4.79 (128.93)	2.26 (0.59)	4.78 (3.72)
Vorinostat	Abdominal Pain	42	2.1 (1.55 - 2.85)	2.09 (24.07)	1.07 (-0.6)	2.09 (1.62)

Vorinostat	Weight Decreased	39	1.6 (1.17 - 2.19)	1.59 (8.66)	0.67 (-0.99)	1.59 (1.22)
Vorinostat	Disease Progression	38	3.89 (2.83 - 5.36)	3.87 (81.09)	1.95 (0.29)	3.87 (2.96)
Vorinostat	Hypokalaemia	37	9.56 (6.92 - 13.21)	9.5 (281.35)	3.25 (1.58)	9.49 (7.24)
Vorinostat	Hypotension	35	2.02 (1.45 - 2.82)	2.02 (17.99)	1.01 (-0.65)	2.02 (1.53)
Vorinostat	Off Label Use	34	0.49 (0.35 - 0.69)	0.5 (17.6)	-1.01 (-2.68)	0.5 (0.37)
Vorinostat	Acute Kidney Injury	33	1.94 (1.37 - 2.73)	1.93 (14.82)	0.95 (-0.72)	1.93 (1.45)
Vorinostat	Pulmonary Embolism	33	3.9 (2.77 - 5.49)	3.88 (70.7)	1.96 (0.29)	3.88 (2.91)
Vorinostat	Blood Creatinine Increased	32	5.68 (4.01 - 8.04)	5.65 (122.56)	2.5 (0.83)	5.65 (4.22)
Vorinostat	Constipation	32	1.78 (1.26 - 2.52)	1.78 (10.89)	0.83 (-0.84)	1.78 (1.33)
Vorinostat	Pleural Effusion	31	5.88 (4.13 - 8.37)	5.85 (124.79)	2.55 (0.88)	5.85 (4.35)
Vorinostat	Headache	29	0.52 (0.36 - 0.75)	0.53 (12.46)	-0.92 (-2.59)	0.53 (0.39)
Vorinostat	Hyponatraemia	26	5.37 (3.65 - 7.89)	5.35 (91.91)	2.42 (0.75)	5.34 (3.87)
Vorinostat	Dizziness	25	0.58 (0.39 - 0.86)	0.58 (7.66)	-0.79 (-2.45)	0.58 (0.42)
Vorinostat	Drug Ineffective	24	0.2 (0.14 - 0.31)	0.21 (73.72)	-2.26 (-3.93)	0.21 (0.15)
Vorinostat	Pancytopenia	24	5.21 (3.49 - 7.78)	5.19 (81.26)	2.38 (0.71)	5.19 (3.71)
Vorinostat	Urinary Tract Infection	24	1.63 (1.09 - 2.44)	1.63 (5.84)	0.7 (-0.96)	1.63 (1.16)
Vorinostat	Cutaneous T-Cell Lymphoma	24	159.81 (106.66 - 239.44)	159.09 (3708.29)	7.29 (5.62)	156.48 (111.57)
Vorinostat	Colitis	23	7.43 (4.93 - 11.19)	7.4 (127.31)	2.89 (1.22)	7.4 (5.25)
Vorinostat	Muscular Weakness	23	2.3 (1.52 - 3.46)	2.29 (16.74)	1.2 (-0.47)	2.29 (1.63)
Vorinostat	Malignant Neoplasm Progression	23	2.73 (1.81 - 4.11)	2.72 (25.05)	1.44 (-0.22)	2.72 (1.93)
Vorinostat	Renal Failure	23	1.89 (1.25 - 2.84)	1.88 (9.56)	0.91 (-0.75)	1.88 (1.34)
Vorinostat	Pain	22	0.39 (0.26 - 0.6)	0.39 (20.69)	-1.34 (-3.01)	0.39 (0.28)
Vorinostat	Hyperglycaemia	22	6.98 (4.59 - 10.6)	6.95 (112.06)	2.8 (1.13)	6.95 (4.89)
Vorinostat	Dysgeusia	22	3.28 (2.16 - 4.99)	3.28 (34.8)	1.71 (0.04)	3.27 (2.31)
Vorinostat	Confusional State	22	1.58 (1.04 - 2.39)	1.57 (4.6)	0.65 (-1.01)	1.57 (1.11)
Vorinostat	Syncope	21	2.39 (1.56 - 3.67)	2.38 (16.87)	1.25 (-0.41)	2.38 (1.66)

Vorinostat	Embolism	21	30.02 (19.54 - 46.11)	29.9 (584.91)	4.9 (3.23)	29.81 (20.82)
Vorinostat	Chills	21	2.05 (1.33 - 3.14)	2.04 (11.2)	1.03 (-0.64)	2.04 (1.43)
Vorinostat	Rash	21	0.54 (0.35 - 0.83)	0.54 (8.3)	-0.89 (-2.56)	0.54 (0.38)
Vorinostat	Fall	20	0.68 (0.44 - 1.06)	0.69 (2.9)	-0.54 (-2.21)	0.69 (0.47)
Vorinostat	Infection	19	1.57 (1 - 2.46)	1.56 (3.87)	0.64 (-1.02)	1.56 (1.07)
Vorinostat	Malaise	19	0.47 (0.3 - 0.74)	0.48 (11.08)	-1.07 (-2.74)	0.48 (0.33)
Vorinostat	Atrial Fibrillation	19	2.25 (1.43 - 3.53)	2.25 (13.14)	1.17 (-0.5)	2.24 (1.54)
Vorinostat	Electrocardiogram Qt Prolonged	19	6.18 (3.94 - 9.7)	6.16 (82.2)	2.62 (0.96)	6.16 (4.23)
Vorinostat	General Physical Health Deterioration	19	2.08 (1.32 - 3.26)	2.07 (10.6)	1.05 (-0.61)	2.07 (1.42)
Vorinostat	Hypoxia	18	6.22 (3.92 - 9.89)	6.21 (78.62)	2.63 (0.97)	6.2 (4.21)
Vorinostat	Hypertension	18	0.99 (0.62 - 1.57)	0.99 (0)	-0.02 (-1.69)	0.99 (0.67)
Vorinostat	Cellulitis	18	3.99 (2.51 - 6.34)	3.98 (40.19)	1.99 (0.33)	3.98 (2.7)
Vorinostat	Oedema Peripheral	18	1.67 (1.05 - 2.65)	1.66 (4.78)	0.73 (-0.93)	1.66 (1.13)
Vorinostat	Alanine Aminotransferase Increased	18	3.56 (2.24 - 5.65)	3.55 (32.94)	1.83 (0.16)	3.55 (2.41)
Vorinostat	Deep Vein Thrombosis	18	3.04 (1.91 - 4.82)	3.03 (24.49)	1.6 (-0.07)	3.03 (2.06)
Vorinostat	Seizure	17	1.16 (0.72 - 1.86)	1.16 (0.36)	0.21 (-1.46)	1.16 (0.78)
Vorinostat	Renal Impairment	17	2.38 (1.48 - 3.82)	2.37 (13.49)	1.25 (-0.42)	2.37 (1.59)
Vorinostat	Aspartate Aminotransferase Increased	17	3.94 (2.45 - 6.34)	3.93 (37.13)	1.97 (0.31)	3.93 (2.64)
Vorinostat	Toxicity To Various Agents	17	1.08 (0.67 - 1.73)	1.08 (0.09)	0.11 (-1.56)	1.08 (0.72)
Vorinostat	Hypophagia	17	7.13 (4.43 - 11.48)	7.11 (89.25)	2.83 (1.16)	7.11 (4.77)
Vorinostat	Epistaxis	16	2.42 (1.48 - 3.96)	2.42 (13.35)	1.27 (-0.39)	2.42 (1.6)
Vorinostat	Lymphocyte Count Decreased	16	10.06 (6.16 - 16.44)	10.03 (130.03)	3.33 (1.66)	10.02 (6.65)
Vorinostat	Back Pain	15	0.73 (0.44 - 1.21)	0.73 (1.52)	-0.46 (-2.12)	0.73 (0.48)
Vorinostat	Device Related Infection	15	9.89 (5.96 - 16.42)	9.87 (119.41)	3.3 (1.63)	9.86 (6.45)

Vorinostat	Dysphagia	15	1.75 (1.05 - 2.9)	1.75 (4.8)	0.8 (-0.86)	1.75 (1.14)
Vorinostat	Myocardial Infarction	15	0.95 (0.58 - 1.59)	0.96 (0.03)	-0.07 (-1.73)	0.96 (0.62)
Vorinostat	Staphylococcal Infection	15	5.19 (3.12 - 8.61)	5.18 (50.54)	2.37 (0.7)	5.17 (3.39)
Vorinostat	Product Use Issue	15	0.94 (0.57 - 1.57)	0.94 (0.05)	-0.08 (-1.75)	0.94 (0.62)
Vorinostat	Cough	14	0.58 (0.35 - 0.99)	0.58 (4.15)	-0.77 (-2.44)	0.58 (0.38)
Vorinostat	Pneumonitis	14	6.49 (3.84 - 10.97)	6.48 (64.83)	2.69 (1.03)	6.47 (4.17)
Vorinostat	Haematocrit Decreased	14	8.25 (4.88 - 13.94)	8.23 (88.89)	3.04 (1.37)	8.22 (5.3)
Vorinostat	Somnolence	13	0.75 (0.43 - 1.29)	0.75 (1.1)	-0.42 (-2.08)	0.75 (0.47)
Vorinostat	Pulmonary Oedema	13	3.36 (1.95 - 5.8)	3.36 (21.54)	1.75 (0.08)	3.36 (2.13)
Vorinostat	Pruritus	13	0.4 (0.23 - 0.7)	0.41 (11.4)	-1.3 (-2.97)	0.41 (0.26)
Vorinostat	Leukopenia	13	3.09 (1.79 - 5.32)	3.08 (18.3)	1.62 (-0.04)	3.08 (1.95)
Vorinostat	Blood Glucose Increased	13	0.76 (0.44 - 1.32)	0.77 (0.94)	-0.39 (-2.05)	0.77 (0.49)
Vorinostat	Insomnia	13	0.55 (0.32 - 0.95)	0.55 (4.71)	-0.85 (-2.52)	0.55 (0.35)
Vorinostat	Product Dose Omission Issue	12	0.28 (0.16 - 0.5)	0.29 (21.69)	-1.81 (-3.48)	0.29 (0.18)
Vorinostat	Neutropenic Sepsis	12	18.83 (10.68 - 33.2)	18.79 (201.78)	4.23 (2.56)	18.76 (11.67)
Vorinostat	Respiratory Distress	12	5.06 (2.87 - 8.92)	5.05 (39.02)	2.34 (0.67)	5.05 (3.14)
Vorinostat	Hypocalcaemia	12	7.44 (4.22 - 13.11)	7.43 (66.7)	2.89 (1.23)	7.42 (4.62)
Vorinostat	Herpes Zoster	11	2.15 (1.19 - 3.88)	2.15 (6.74)	1.1 (-0.56)	2.15 (1.31)
Vorinostat	Abdominal Pain Upper	11	0.62 (0.34 - 1.12)	0.62 (2.53)	-0.68 (-2.35)	0.62 (0.38)
Vorinostat	Stomatitis	11	2.12 (1.17 - 3.83)	2.12 (6.51)	1.08 (-0.58)	2.12 (1.29)
Vorinostat	Adverse Event	11	1.4 (0.77 - 2.53)	1.4 (1.25)	0.48 (-1.18)	1.4 (0.85)
Vorinostat	Haemorrhage	11	1.22 (0.68 - 2.21)	1.22 (0.45)	0.29 (-1.37)	1.22 (0.75)
Vorinostat	Arthralgia	11	0.31 (0.17 - 0.55)	0.31 (17.27)	-1.7 (-3.37)	0.31 (0.19)
Vorinostat	Alopecia	11	0.62 (0.34 - 1.12)	0.62 (2.55)	-0.69 (-2.35)	0.62 (0.38)
Vorinostat	Muscle Spasms	11	0.67 (0.37 - 1.22)	0.68 (1.72)	-0.57 (-2.23)	0.68 (0.41)
Vorinostat	No Adverse Event	10	0.73 (0.39 - 1.36)	0.73 (0.97)	-0.45 (-2.11)	0.73 (0.44)
Vorinostat	Neuropathy Peripheral	10	1.25 (0.67 - 2.32)	1.25 (0.49)	0.32 (-1.35)	1.25 (0.74)

Vorinostat	Sinus Tachycardia	10	8.37 (4.5 - 15.57)	8.36 (64.72)	3.06 (1.4)	8.35 (4.97)
Vorinostat	Gastrointestinal Haemorrhage	10	1.3 (0.7 - 2.41)	1.3 (0.67)	0.37 (-1.29)	1.3 (0.77)
Vorinostat	Haematuria	10	3.27 (1.76 - 6.08)	3.26 (15.7)	1.71 (0.04)	3.26 (1.94)
Vorinostat	Chest Pain	10	0.62 (0.33 - 1.15)	0.62 (2.35)	-0.69 (-2.36)	0.62 (0.37)
Vorinostat	Lower Respiratory Tract Infection	10	2.75 (1.48 - 5.11)	2.75 (11.1)	1.46 (-0.21)	2.75 (1.63)
Vorinostat	Bone Marrow Failure	10	5.06 (2.72 - 9.41)	5.05 (32.48)	2.34 (0.67)	5.05 (3)
Vorinostat	Renal Disorder	10	2.45 (1.32 - 4.56)	2.45 (8.56)	1.29 (-0.38)	2.45 (1.46)
Vorinostat	Hepatic Function Abnormal	10	3.31 (1.78 - 6.15)	3.3 (16.07)	1.72 (0.06)	3.3 (1.97)
Vorinostat	Condition Aggravated	10	0.41 (0.22 - 0.76)	0.41 (8.58)	-1.29 (-2.96)	0.41 (0.24)
Vorinostat	Cardiac Arrest	9	1.26 (0.65 - 2.41)	1.25 (0.47)	0.33 (-1.34)	1.25 (0.73)
Vorinostat	Acute Myeloid Leukaemia	9	6.81 (3.54 - 13.09)	6.8 (44.48)	2.76 (1.1)	6.79 (3.93)
Vorinostat	Haemorrhage Intracranial	9	6.52 (3.39 - 12.54)	6.51 (41.95)	2.7 (1.04)	6.51 (3.76)
Vorinostat	Blood Bilirubin Increased	9	3.95 (2.05 - 7.6)	3.94 (19.78)	1.98 (0.31)	3.94 (2.28)
Vorinostat	Acute Respiratory Distress Syndrome	9	6.06 (3.15 - 11.66)	6.06 (37.97)	2.6 (0.93)	6.05 (3.5)
Vorinostat	Blood Urea Increased	9	6.32 (3.29 - 12.16)	6.31 (40.23)	2.66 (0.99)	6.31 (3.65)
Vorinostat	Blood Albumin Decreased	9	13.69 (7.12 - 26.34)	13.67 (105.53)	3.77 (2.1)	13.65 (7.89)
Vorinostat	Cardiac Failure	9	1.29 (0.67 - 2.48)	1.29 (0.58)	0.37 (-1.3)	1.29 (0.75)
Vorinostat	Lymphopenia	9	7.46 (3.88 - 14.35)	7.45 (50.23)	2.9 (1.23)	7.44 (4.31)
Vorinostat	Blood Sodium Decreased	9	5.75 (2.99 - 11.06)	5.74 (35.25)	2.52 (0.85)	5.74 (3.32)
Vorinostat	Erythema	9	0.48 (0.25 - 0.93)	0.48 (4.96)	-1.05 (-2.71)	0.48 (0.28)
Vorinostat	Lung Infiltration	9	11.95 (6.21 - 22.99)	11.93 (90.03)	3.57 (1.91)	11.92 (6.89)
Vorinostat	Cardio-Respiratory Arrest	9	2.47 (1.28 - 4.74)	2.46 (7.84)	1.3 (-0.37)	2.46 (1.43)
Vorinostat	Pericardial Effusion	8	4.15 (2.07 - 8.3)	4.14 (19.08)	2.05 (0.38)	4.14 (2.32)
Vorinostat	Upper Respiratory Tract Infection	8	2.01 (1 - 4.01)	2 (4.02)	1 (-0.66)	2 (1.12)
Vorinostat	Treatment Failure	8	1.17 (0.58 - 2.34)	1.17 (0.19)	0.22 (-1.44)	1.17 (0.65)
Vorinostat	Hyperkalaemia	8	2.73 (1.36 - 5.46)	2.72 (8.74)	1.45 (-0.22)	2.72 (1.52)

Vorinostat	Septic Shock	8	2.22 (1.11 - 4.45)	2.22 (5.38)	1.15 (-0.51)	2.22 (1.24)
Vorinostat	Fibrin D Dimer Increased	8	26.68 (13.32 - 53.42)	26.64 (196.85)	4.73 (3.06)	26.56 (14.86)
Vorinostat	Delirium	8	2.78 (1.39 - 5.56)	2.78 (9.09)	1.47 (-0.19)	2.78 (1.55)
Vorinostat	Mucosal Inflammation	8	3.64 (1.82 - 7.29)	3.64 (15.3)	1.86 (0.2)	3.64 (2.04)
Vorinostat	Skin Exfoliation	8	1.15 (0.58 - 2.3)	1.15 (0.16)	0.2 (-1.46)	1.15 (0.64)
Vorinostat	Therapy Cessation	8	1.83 (0.91 - 3.66)	1.83 (3.01)	0.87 (-0.8)	1.83 (1.02)
Vorinostat	Myelodysplastic Syndrome	8	6.31 (3.16 - 12.63)	6.31 (35.7)	2.66 (0.99)	6.3 (3.53)
Vorinostat	Hypophosphataemia	8	12.79 (6.39 - 25.6)	12.77 (86.71)	3.67 (2.01)	12.76 (7.14)
Vorinostat	Product Administered To Patient Of Inappropriate Age	8	6.79 (3.4 - 13.6)	6.79 (39.45)	2.76 (1.1)	6.78 (3.8)
Vorinostat	Dyspnoea Exertional	8	2.56 (1.28 - 5.13)	2.56 (7.61)	1.36 (-0.31)	2.56 (1.43)
Vorinostat	Blood Culture Positive	8	31.53 (15.74 - 63.15)	31.48 (235.32)	4.97 (3.3)	31.38 (17.55)
Vorinostat	Electrolyte Imbalance	8	8.45 (4.22 - 16.9)	8.43 (52.39)	3.08 (1.41)	8.43 (4.72)
Vorinostat	Abdominal Distension	8	0.9 (0.45 - 1.8)	0.9 (0.09)	-0.15 (-1.82)	0.9 (0.5)
Vorinostat	Escherichia Infection	7	10.57 (5.03 - 22.19)	10.56 (60.5)	3.4 (1.73)	10.55 (5.67)
Vorinostat	Cerebrovascular Accident	7	0.47 (0.23 - 0.99)	0.47 (4.1)	-1.08 (-2.74)	0.47 (0.25)
Vorinostat	Hypoalbuminaemia	7	11 (5.24 - 23.1)	10.99 (63.5)	3.46 (1.79)	10.98 (5.9)
Vorinostat	Multiple Organ Dysfunction Syndrome	7	1.83 (0.87 - 3.84)	1.83 (2.64)	0.87 (-0.79)	1.83 (0.98)
Vorinostat	Mental Status Changes	7	2.95 (1.4 - 6.19)	2.94 (8.99)	1.56 (-0.11)	2.94 (1.58)
Vorinostat	Thrombosis	7	0.99 (0.47 - 2.08)	0.99 (0)	-0.01 (-1.68)	0.99 (0.53)
Vorinostat	Tachycardia	7	0.92 (0.44 - 1.94)	0.92 (0.04)	-0.11 (-1.78)	0.92 (0.5)
Vorinostat	Skin Lesion	7	2.96 (1.41 - 6.21)	2.96 (9.08)	1.56 (-0.1)	2.96 (1.59)
Vorinostat	Hyperbilirubinaemia	7	8.11 (3.86 - 17.03)	8.1 (43.55)	3.02 (1.35)	8.1 (4.35)
Vorinostat	Balance Disorder	7	0.9 (0.43 - 1.9)	0.9 (0.07)	-0.15 (-1.81)	0.9 (0.49)
Vorinostat	Blood Pressure Decreased	7	1.24 (0.59 - 2.59)	1.24 (0.31)	0.3 (-1.36)	1.23 (0.66)
Vorinostat	Transaminases Increased	7	3.61 (1.72 - 7.57)	3.6 (13.18)	1.85 (0.18)	3.6 (1.94)

Vorinostat	Blood Potassium Decreased	7	2.7 (1.28 - 5.66)	2.69 (7.46)	1.43 (-0.24)	2.69 (1.45)
Vorinostat	Cardiac Failure Congestive	7	0.92 (0.44 - 1.93)	0.92 (0.05)	-0.12 (-1.79)	0.92 (0.49)
Vorinostat	Plasma Cell Myeloma	7	1.78 (0.85 - 3.73)	1.78 (2.38)	0.83 (-0.84)	1.78 (0.96)
Vorinostat	Escherichia Bacteraemia	6	33.91 (15.21 - 75.63)	33.88 (190.75)	5.08 (3.41)	33.76 (17.26)
Vorinostat	Small Intestinal Obstruction	6	5.71 (2.57 - 12.73)	5.71 (23.3)	2.51 (0.85)	5.71 (2.92)
Vorinostat	Sinus Bradycardia	6	7.29 (3.27 - 16.24)	7.28 (32.49)	2.86 (1.2)	7.28 (3.72)
Vorinostat	Lethargy	6	1.21 (0.54 - 2.69)	1.21 (0.21)	0.27 (-1.4)	1.21 (0.62)
Vorinostat	Tumour Lysis Syndrome	6	8.3 (3.73 - 18.49)	8.29 (38.45)	3.05 (1.38)	8.29 (4.24)
Vorinostat	Urinary Retention	6	2.1 (0.95 - 4.69)	2.1 (3.48)	1.07 (-0.59)	2.1 (1.08)
Vorinostat	Failure To Thrive	6	12.86 (5.77 - 28.66)	12.85 (65.49)	3.68 (2.02)	12.83 (6.57)
Vorinostat	Gait Disturbance	6	0.34 (0.15 - 0.76)	0.34 (7.63)	-1.55 (-3.22)	0.34 (0.17)
Vorinostat	Abdominal Discomfort	6	0.4 (0.18 - 0.89)	0.4 (5.38)	-1.32 (-2.98)	0.4 (0.21)
Vorinostat	Neoplasm Progression	6	1.84 (0.82 - 4.09)	1.84 (2.28)	0.88 (-0.79)	1.84 (0.94)
Vorinostat	Dry Mouth	6	0.87 (0.39 - 1.95)	0.87 (0.11)	-0.19 (-1.86)	0.87 (0.45)
Vorinostat	Oral Candidiasis	6	5.66 (2.54 - 12.62)	5.66 (23.01)	2.5 (0.83)	5.66 (2.89)
Vorinostat	Intentional Product Misuse	6	0.61 (0.27 - 1.35)	0.61 (1.52)	-0.72 (-2.38)	0.61 (0.31)
Vorinostat	Haemoptysis	6	2.44 (1.1 - 5.43)	2.44 (5.09)	1.29 (-0.38)	2.44 (1.25)
Vorinostat	Loss Of Consciousness	6	0.55 (0.25 - 1.22)	0.55 (2.25)	-0.87 (-2.54)	0.55 (0.28)
Vorinostat	White Blood Cell Count Increased	6	1.8 (0.81 - 4.01)	1.8 (2.13)	0.85 (-0.82)	1.8 (0.92)
Vorinostat	Drug Interaction	6	0.44 (0.2 - 0.98)	0.44 (4.31)	-1.19 (-2.85)	0.44 (0.22)
Vorinostat	Culture Urine Positive	6	50.57 (22.66 - 112.85)	50.51 (289.65)	5.65 (3.98)	50.25 (25.67)
Vorinostat	Myocardial Ischaemia	6	5.76 (2.59 - 12.83)	5.75 (23.55)	2.52 (0.86)	5.75 (2.94)
Vorinostat	Depressed Level Of Consciousness	6	1.84 (0.83 - 4.1)	1.84 (2.31)	0.88 (-0.79)	1.84 (0.94)
Vorinostat	Hydrocephalus	6	12.82 (5.75 - 28.57)	12.81 (65.23)	3.68 (2.01)	12.79 (6.54)
Vorinostat	C-Reactive Protein Increased	6	1.99 (0.89 - 4.43)	1.99 (2.94)	0.99 (-0.68)	1.99 (1.02)
Vorinostat	Non-Cardiac Chest Pain	5	16.81 (6.99 - 40.44)	16.8 (74.15)	4.07 (2.4)	16.77 (8.04)
Vorinostat	Ejection Fraction Decreased	5	3.76 (1.56 - 9.04)	3.76 (10.11)	1.91 (0.24)	3.75 (1.8)

Vorinostat	Stem Cell Transplant	5	25.87 (10.75 - 62.25)	25.84 (119.09)	4.69 (3.02)	25.78 (12.36)
Vorinostat	Oropharyngeal Pain	5	0.6 (0.25 - 1.45)	0.6 (1.3)	-0.73 (-2.39)	0.6 (0.29)
Vorinostat	Bacterial Infection	5	3.26 (1.35 - 7.83)	3.25 (7.8)	1.7 (0.04)	3.25 (1.56)
Vorinostat	International Normalised Ratio Increased	5	1.93 (0.8 - 4.64)	1.93 (2.25)	0.95 (-0.72)	1.93 (0.93)
Vorinostat	Neoplasm Malignant	5	0.86 (0.36 - 2.08)	0.86 (0.11)	-0.21 (-1.88)	0.86 (0.41)
Vorinostat	Acute Myocardial Infarction	5	1.9 (0.79 - 4.57)	1.9 (2.14)	0.93 (-0.74)	1.9 (0.91)
Vorinostat	Enterocolitis	5	11.19 (4.65 - 26.91)	11.18 (46.29)	3.48 (1.81)	11.17 (5.36)
Vorinostat	Bradycardia	5	1.07 (0.45 - 2.58)	1.07 (0.03)	0.1 (-1.56)	1.07 (0.52)
Vorinostat	Influenza Like Illness	5	0.67 (0.28 - 1.6)	0.67 (0.84)	-0.59 (-2.25)	0.67 (0.32)
Vorinostat	Pulmonary Haemorrhage	5	7.19 (2.99 - 17.29)	7.19 (26.61)	2.84 (1.18)	7.18 (3.45)
Vorinostat	Contusion	5	0.6 (0.25 - 1.43)	0.6 (1.37)	-0.75 (-2.41)	0.6 (0.29)
Vorinostat	Acidosis	5	7.81 (3.25 - 18.78)	7.81 (29.65)	2.96 (1.3)	7.8 (3.74)
Vorinostat	Therapy Non-Responder	5	1.1 (0.46 - 2.63)	1.1 (0.04)	0.13 (-1.53)	1.1 (0.53)
Vorinostat	Oedema	5	1.09 (0.45 - 2.61)	1.09 (0.03)	0.12 (-1.55)	1.09 (0.52)
Vorinostat	Cholangitis	5	10.18 (4.23 - 24.47)	10.17 (41.3)	3.34 (1.68)	10.16 (4.88)
Vorinostat	Pancreatitis	5	1.09 (0.45 - 2.63)	1.09 (0.04)	0.13 (-1.54)	1.09 (0.52)
Vorinostat	Myalgia	5	0.34 (0.14 - 0.82)	0.34 (6.4)	-1.55 (-3.22)	0.34 (0.16)
Vorinostat	Disorientation	5	1.44 (0.6 - 3.45)	1.44 (0.66)	0.52 (-1.15)	1.44 (0.69)
Vorinostat	Arrhythmia	5	1.22 (0.51 - 2.93)	1.22 (0.2)	0.29 (-1.38)	1.22 (0.59)
Vorinostat	Hypoaesthesia	5	0.37 (0.16 - 0.9)	0.37 (5.24)	-1.42 (-3.08)	0.37 (0.18)
Vorinostat	Anxiety	5	0.2 (0.08 - 0.47)	0.2 (16.57)	-2.35 (-4.02)	0.2 (0.09)
Vorinostat	Atelectasis	5	5.93 (2.46 - 14.25)	5.92 (20.44)	2.57 (0.9)	5.92 (2.84)
Vorinostat	Tremor	5	0.34 (0.14 - 0.82)	0.34 (6.31)	-1.54 (-3.21)	0.34 (0.16)
Vorinostat	Cardiac Disorder	5	0.6 (0.25 - 1.44)	0.6 (1.34)	-0.74 (-2.4)	0.6 (0.29)
Vorinostat	Hyperhidrosis	5	0.44 (0.18 - 1.06)	0.44 (3.55)	-1.18 (-2.85)	0.44 (0.21)
Vorinostat	Hypervolaemia	5	2.8 (1.16 - 6.72)	2.8 (5.77)	1.48 (-0.18)	2.8 (1.34)

Vorinostat	Blood Alkaline Phosphatase Increased	5	2.43 (1.01 - 5.83)	2.43 (4.19)	1.28 (-0.39)	2.43 (1.16)
Vorinostat	Malnutrition	5	5.44 (2.26 - 13.09)	5.44 (18.11)	2.44 (0.78)	5.44 (2.61)
Vorinostat	Red Blood Cell Count Decreased	5	2.01 (0.84 - 4.82)	2.01 (2.52)	1 (-0.66)	2.01 (0.96)
Vorinostat	Mitral Valve Incompetence	5	5.34 (2.22 - 12.84)	5.34 (17.62)	2.42 (0.75)	5.34 (2.56)
Vorinostat	Blood Calcium Decreased	5	5.05 (2.1 - 12.14)	5.04 (16.21)	2.33 (0.67)	5.04 (2.42)
Vorinostat	Febrile Bone Marrow Aplasia	5	14.21 (5.91 - 34.18)	14.2 (61.26)	3.83 (2.16)	14.18 (6.8)
Vorinostat	Lower Gastrointestinal Haemorrhage	4	5.76 (2.16 - 15.37)	5.76 (15.73)	2.53 (0.86)	5.76 (2.53)
Vorinostat	Pyelonephritis	4	5.42 (2.03 - 14.46)	5.42 (14.41)	2.44 (0.77)	5.42 (2.38)
Vorinostat	Left Ventricular Dysfunction	4	6.82 (2.56 - 18.2)	6.82 (19.85)	2.77 (1.1)	6.82 (3)
Vorinostat	Chronic Obstructive Pulmonary Disease	4	0.86 (0.32 - 2.3)	0.86 (0.09)	-0.21 (-1.88)	0.86 (0.38)
Vorinostat	Deafness	4	1.8 (0.67 - 4.79)	1.79 (1.41)	0.84 (-0.82)	1.79 (0.79)
Vorinostat	Metabolic Acidosis	4	1.52 (0.57 - 4.05)	1.52 (0.71)	0.6 (-1.06)	1.52 (0.67)
Vorinostat	Hypoglycaemia	4	0.95 (0.36 - 2.53)	0.95 (0.01)	-0.08 (-1.74)	0.95 (0.42)
Vorinostat	Chest Discomfort	4	0.46 (0.17 - 1.23)	0.46 (2.51)	-1.11 (-2.78)	0.46 (0.2)
Vorinostat	Diverticulitis	4	1.67 (0.63 - 4.45)	1.67 (1.07)	0.74 (-0.93)	1.67 (0.73)
Vorinostat	Skin Infection	4	4.2 (1.58 - 11.2)	4.2 (9.74)	2.07 (0.4)	4.2 (1.85)
Vorinostat	Unresponsive To Stimuli	4	1.74 (0.65 - 4.64)	1.74 (1.26)	0.8 (-0.87)	1.74 (0.77)
Vorinostat	Musculoskeletal Chest Pain	4	2.89 (1.08 - 7.71)	2.89 (4.94)	1.53 (-0.14)	2.89 (1.27)
Vorinostat	Brain Oedema	4	3.76 (1.41 - 10.03)	3.76 (8.1)	1.91 (0.24)	3.76 (1.65)
Vorinostat	Haemorrhoids	4	2.32 (0.87 - 6.18)	2.32 (3)	1.21 (-0.45)	2.32 (1.02)
Vorinostat	Escherichia Sepsis	4	16.01 (6 - 42.71)	16 (56.15)	4 (2.33)	15.97 (7.03)
Vorinostat	Respiratory Tract Infection	4	1.86 (0.7 - 4.95)	1.85 (1.58)	0.89 (-0.78)	1.85 (0.82)
Vorinostat	Fibrin Degradation Products Increased	4	166.43 (61.91 - 447.42)	166.3 (645.91)	7.35 (5.68)	163.45 (71.46)

Vorinostat	Wrong Technique In Product Usage Process	4	0.19 (0.07 - 0.51)	0.19 (13.53)	-2.37 (-4.04)	0.19 (0.09)
Vorinostat	Oesophagitis	4	4.42 (1.66 - 11.79)	4.42 (10.58)	2.14 (0.48)	4.42 (1.94)
Vorinostat	Dry Skin	4	0.37 (0.14 - 0.99)	0.37 (4.23)	-1.42 (-3.09)	0.37 (0.16)
Vorinostat	Drug Intolerance	4	0.48 (0.18 - 1.29)	0.48 (2.2)	-1.05 (-2.71)	0.48 (0.21)
Vorinostat	Respiratory Rate Increased	4	5.58 (2.09 - 14.87)	5.57 (15.01)	2.48 (0.81)	5.57 (2.45)
Vorinostat	Endocarditis	4	9.24 (3.47 - 24.64)	9.23 (29.35)	3.21 (1.54)	9.23 (4.06)
Vorinostat	Ventricular Tachycardia	4	2.88 (1.08 - 7.69)	2.88 (4.92)	1.53 (-0.14)	2.88 (1.27)
Vorinostat	Pain In Extremity	4	0.15 (0.06 - 0.4)	0.15 (19.41)	-2.74 (-4.41)	0.15 (0.07)
Vorinostat	Heart Rate Increased	4	0.47 (0.18 - 1.27)	0.48 (2.32)	-1.07 (-2.74)	0.48 (0.21)
Vorinostat	Hospitalisation	4	0.32 (0.12 - 0.86)	0.32 (5.69)	-1.63 (-3.3)	0.32 (0.14)
Vorinostat	Sudden Death	4	4.38 (1.64 - 11.67)	4.38 (10.42)	2.13 (0.46)	4.37 (1.93)
Vorinostat	Therapeutic Product Effect Incomplete	4	0.41 (0.16 - 1.1)	0.41 (3.32)	-1.27 (-2.94)	0.41 (0.18)
Vorinostat	Bacterial Test Positive	4	15.42 (5.78 - 41.13)	15.41 (53.8)	3.94 (2.28)	15.38 (6.77)
Vorinostat	Torsade De Pointes	4	6.14 (2.3 - 16.36)	6.13 (17.18)	2.62 (0.95)	6.13 (2.7)
Vorinostat	Hypomagnesaemia	4	3.45 (1.29 - 9.19)	3.45 (6.94)	1.78 (0.12)	3.45 (1.52)
Vorinostat	Pseudomonas Infection	4	5.95 (2.23 - 15.86)	5.94 (16.44)	2.57 (0.9)	5.94 (2.61)
Vorinostat	Blood Lactate Dehydrogenase Increased	4	2.98 (1.12 - 7.95)	2.98 (5.27)	1.58 (-0.09)	2.98 (1.31)
Vorinostat	Lymphocyte Count Increased	4	14.71 (5.52 - 39.25)	14.7 (51.01)	3.88 (2.21)	14.68 (6.46)
Vorinostat	Benign Prostatic Hyperplasia	4	8.92 (3.35 - 23.8)	8.92 (28.09)	3.16 (1.49)	8.91 (3.92)
Vorinostat	Fungal Infection	4	1.38 (0.52 - 3.67)	1.38 (0.41)	0.46 (-1.21)	1.38 (0.61)
Vorinostat	Aphasia	4	1.48 (0.55 - 3.94)	1.48 (0.62)	0.56 (-1.1)	1.48 (0.65)
Vorinostat	Metastases To Liver	4	2.7 (1.01 - 7.2)	2.7 (4.28)	1.43 (-0.23)	2.7 (1.19)
Vorinostat	Rhinorrhoea	4	0.72 (0.27 - 1.92)	0.72 (0.43)	-0.47 (-2.14)	0.72 (0.32)
Vorinostat	Hypercalcaemia	4	3.78 (1.42 - 10.08)	3.78 (8.17)	1.92 (0.25)	3.78 (1.66)

Vorinostat	Proctalgia	4	7.04 (2.64 - 18.77)	7.04 (20.7)	2.81 (1.15)	7.03 (3.1)
Vorinostat	Vision Blurred	4	0.34 (0.13 - 0.91)	0.34 (5.03)	-1.54 (-3.21)	0.34 (0.15)
Vorinostat	Bacteraemia	4	4.14 (1.55 - 11.04)	4.14 (9.52)	2.05 (0.38)	4.14 (1.82)
Vorinostat	Abdominal Pain Lower	4	1.77 (0.66 - 4.71)	1.77 (1.33)	0.82 (-0.85)	1.77 (0.78)
Vorinostat	Petechiae	4	4.46 (1.67 - 11.89)	4.46 (10.73)	2.16 (0.49)	4.46 (1.96)
Vorinostat	T-Cell Lymphoma	4	44.54 (16.67 - 118.98)	44.5 (169.31)	5.47 (3.8)	44.3 (19.47)
Vorinostat	Agitation	4	0.62 (0.23 - 1.66)	0.62 (0.91)	-0.68 (-2.35)	0.62 (0.27)
Vorinostat	Enterococcal Infection	4	10.28 (3.86 - 27.42)	10.28 (33.46)	3.36 (1.69)	10.27 (4.52)
Vorinostat	Pneumothorax	4	2.76 (1.03 - 7.35)	2.76 (4.48)	1.46 (-0.2)	2.76 (1.21)
Vorinostat	Productive Cough	4	1.02 (0.38 - 2.72)	1.02 (0)	0.03 (-1.64)	1.02 (0.45)
Vorinostat	Staphylococcal Sepsis	4	9.22 (3.46 - 24.59)	9.22 (29.27)	3.2 (1.54)	9.21 (4.05)
Vorinostat	Non-Small Cell Lung Cancer	4	10.41 (3.9 - 27.76)	10.4 (33.96)	3.38 (1.71)	10.39 (4.57)
Vorinostat	Lung Disorder	4	0.97 (0.37 - 2.59)	0.97 (0)	-0.04 (-1.71)	0.97 (0.43)
Vorinostat	Oesophageal Candidiasis	4	12.35 (4.63 - 32.94)	12.34 (41.64)	3.62 (1.96)	12.33 (5.43)
Vorinostat	Fluid Intake Reduced	4	9.9 (3.71 - 26.4)	9.89 (31.94)	3.3 (1.64)	9.88 (4.35)
Vorinostat	Gastrointestinal Disorder	4	0.38 (0.14 - 1.03)	0.39 (3.93)	-1.38 (-3.04)	0.39 (0.17)
Vorinostat	Graft Versus Host Disease	4	6.59 (2.47 - 17.57)	6.59 (18.94)	2.72 (1.05)	6.58 (2.9)
Vorinostat	Leukocytosis	4	2.64 (0.99 - 7.04)	2.64 (4.08)	1.4 (-0.27)	2.64 (1.16)
Vorinostat	Cerebral Ischaemia	4	8.76 (3.28 - 23.35)	8.75 (27.44)	3.13 (1.46)	8.74 (3.85)
Vorinostat	Dysarthria	3	0.92 (0.3 - 2.85)	0.92 (0.02)	-0.12 (-1.79)	0.92 (0.36)
Vorinostat	Diabetes Mellitus	3	0.43 (0.14 - 1.34)	0.43 (2.23)	-1.21 (-2.87)	0.43 (0.17)
Vorinostat	Lymphadenopathy	3	0.98 (0.32 - 3.05)	0.98 (0)	-0.03 (-1.69)	0.98 (0.38)
Vorinostat	Blood Pressure Increased	3	0.22 (0.07 - 0.69)	0.22 (8.09)	-2.16 (-3.83)	0.22 (0.09)
Vorinostat	Localised Oedema	3	7.38 (2.38 - 22.91)	7.38 (16.53)	2.88 (1.22)	7.37 (2.86)
Vorinostat	Cholecystitis	3	2.97 (0.96 - 9.2)	2.96 (3.9)	1.57 (-0.1)	2.96 (1.15)
Vorinostat	Subdural Haematoma	3	2.32 (0.75 - 7.18)	2.31 (2.24)	1.21 (-0.46)	2.31 (0.9)
Vorinostat	Gastric Haemorrhage	3	2.82 (0.91 - 8.75)	2.82 (3.52)	1.49 (-0.17)	2.82 (1.09)

Vorinostat	Nasopharyngitis	3	0.19 (0.06 - 0.58)	0.19 (10.49)	-2.41 (-4.07)	0.19 (0.07)
Vorinostat	Viral Infection	3	1.09 (0.35 - 3.39)	1.09 (0.02)	0.13 (-1.54)	1.09 (0.42)
Vorinostat	Staphylococcal Bacteraemia	3	8.74 (2.82 - 27.12)	8.73 (20.53)	3.13 (1.46)	8.73 (3.38)
Vorinostat	Acute Respiratory Failure	3	1.88 (0.61 - 5.83)	1.88 (1.23)	0.91 (-0.76)	1.88 (0.73)
Vorinostat	Clostridium Difficile Infection	3	1.62 (0.52 - 5.02)	1.62 (0.71)	0.69 (-0.97)	1.62 (0.63)
Vorinostat	Urticaria	3	0.22 (0.07 - 0.67)	0.22 (8.57)	-2.21 (-3.88)	0.22 (0.08)
Vorinostat	Aspiration	3	3.39 (1.09 - 10.5)	3.38 (5.04)	1.76 (0.09)	3.38 (1.31)
Vorinostat	Rash Maculo-Papular	3	1.65 (0.53 - 5.1)	1.64 (0.76)	0.72 (-0.95)	1.64 (0.64)
Vorinostat	Lymphoma	3	2.18 (0.7 - 6.77)	2.18 (1.92)	1.12 (-0.54)	2.18 (0.85)
Vorinostat	Memory Impairment	3	0.24 (0.08 - 0.76)	0.24 (7.04)	-2.03 (-3.7)	0.24 (0.09)
Vorinostat	Urine Output Decreased	3	3.92 (1.27 - 12.18)	3.92 (6.53)	1.97 (0.3)	3.92 (1.52)
Vorinostat	Liver Function Test Abnormal	3	1.17 (0.38 - 3.62)	1.17 (0.07)	0.22 (-1.44)	1.17 (0.45)
Vorinostat	Dermatitis Exfoliative	3	7.14 (2.3 - 22.14)	7.13 (15.81)	2.83 (1.17)	7.13 (2.76)
Vorinostat	Sinusitis	3	0.33 (0.11 - 1.02)	0.33 (4.07)	-1.6 (-3.26)	0.33 (0.13)
Vorinostat	Fracture	3	1.78 (0.57 - 5.51)	1.78 (1.02)	0.83 (-0.84)	1.78 (0.69)
Vorinostat	Bronchitis	3	0.44 (0.14 - 1.37)	0.44 (2.12)	-1.18 (-2.84)	0.44 (0.17)
Vorinostat	Gastritis	3	1.31 (0.42 - 4.05)	1.31 (0.22)	0.39 (-1.28)	1.31 (0.51)
Vorinostat	Dyspepsia	3	0.36 (0.12 - 1.11)	0.36 (3.42)	-1.47 (-3.14)	0.36 (0.14)
Vorinostat	Pneumonia Fungal	3	7.89 (2.54 - 24.47)	7.88 (18.01)	2.98 (1.31)	7.88 (3.05)
Vorinostat	Hyperkeratosis	3	6.2 (2 - 19.23)	6.2 (13.06)	2.63 (0.96)	6.19 (2.4)
Vorinostat	Gastrointestinal Toxicity	3	8.39 (2.7 - 26.04)	8.39 (19.5)	3.07 (1.4)	8.38 (3.25)
Vorinostat	Gastroenteritis	3	2.39 (0.77 - 7.41)	2.39 (2.42)	1.26 (-0.41)	2.39 (0.93)
Vorinostat	Intestinal Perforation	3	3.14 (1.01 - 9.73)	3.14 (4.36)	1.65 (-0.02)	3.13 (1.22)
Vorinostat	Metastases To Bone	3	2.23 (0.72 - 6.9)	2.22 (2.02)	1.15 (-0.51)	2.22 (0.86)
Vorinostat	Unevaluable Event	3	0.43 (0.14 - 1.32)	0.43 (2.3)	-1.23 (-2.89)	0.43 (0.17)
Vorinostat	Metastases To Lung	3	3.13 (1.01 - 9.72)	3.13 (4.35)	1.65 (-0.02)	3.13 (1.21)
Vorinostat	Ascites	3	1.17 (0.38 - 3.64)	1.17 (0.08)	0.23 (-1.44)	1.17 (0.46)

Vorinostat	Flank Pain	3	3.67 (1.18 - 11.39)	3.67 (5.83)	1.88 (0.21)	3.67 (1.42)
Vorinostat	Restlessness	3	0.93 (0.3 - 2.89)	0.93 (0.01)	-0.1 (-1.77)	0.93 (0.36)
Vorinostat	Vena Cava Thrombosis	3	20.71 (6.67 - 64.31)	20.7 (56.12)	4.37 (2.7)	20.65 (8)
Vorinostat	Blood Creatine Phosphokinase Increased	3	1.23 (0.4 - 3.8)	1.23 (0.12)	0.29 (-1.37)	1.23 (0.48)
Vorinostat	Proteinuria	3	1.94 (0.63 - 6.03)	1.94 (1.37)	0.96 (-0.71)	1.94 (0.75)
Vorinostat	Hypoventilation	3	10.6 (3.41 - 32.89)	10.59 (26.03)	3.4 (1.74)	10.58 (4.1)
Vorinostat	Hypovolaemic Shock	3	7.4 (2.39 - 22.97)	7.4 (16.59)	2.89 (1.22)	7.39 (2.87)
Vorinostat	Intra-Abdominal Haemorrhage	3	14.29 (4.6 - 44.36)	14.28 (37.01)	3.83 (2.17)	14.26 (5.53)
Vorinostat	Blood Pressure Systolic Increased	3	1.75 (0.56 - 5.41)	1.74 (0.95)	0.8 (-0.86)	1.74 (0.68)
Vorinostat	Polyneuropathy	3	3.04 (0.98 - 9.44)	3.04 (4.11)	1.6 (-0.06)	3.04 (1.18)
Vorinostat	Herpes Simplex	3	6.27 (2.02 - 19.47)	6.27 (13.28)	2.65 (0.98)	6.27 (2.43)
Vorinostat	Dysuria	3	0.92 (0.3 - 2.85)	0.92 (0.02)	-0.12 (-1.79)	0.92 (0.36)
Vorinostat	Neuralgia	3	1.42 (0.46 - 4.41)	1.42 (0.37)	0.51 (-1.16)	1.42 (0.55)
Vorinostat	Cerebral Atrophy	3	8.74 (2.82 - 27.13)	8.74 (20.54)	3.13 (1.46)	8.73 (3.38)
Vorinostat	Paraesthesia	3	0.21 (0.07 - 0.66)	0.21 (8.72)	-2.23 (-3.89)	0.21 (0.08)
Vorinostat	Hepatic Enzyme Increased	3	0.54 (0.17 - 1.66)	0.54 (1.21)	-0.9 (-2.57)	0.54 (0.21)
Vorinostat	Bone Pain	3	0.58 (0.19 - 1.79)	0.58 (0.92)	-0.79 (-2.46)	0.58 (0.22)
Vorinostat	Complication Associated With Device	3	1.3 (0.42 - 4.04)	1.3 (0.21)	0.38 (-1.29)	1.3 (0.5)
Vorinostat	Clostridial Infection	3	9.71 (3.13 - 30.13)	9.7 (23.39)	3.28 (1.61)	9.69 (3.76)
Vorinostat	Accidental Overdose	3	1.03 (0.33 - 3.2)	1.03 (0)	0.04 (-1.62)	1.03 (0.4)
Vorinostat	Liver Disorder	3	0.81 (0.26 - 2.51)	0.81 (0.13)	-0.3 (-1.97)	0.81 (0.31)
Vorinostat	Hemiparesis	3	1.98 (0.64 - 6.14)	1.98 (1.46)	0.99 (-0.68)	1.98 (0.77)
Vorinostat	B-Cell Lymphoma	3	9.86 (3.18 - 30.59)	9.85 (23.83)	3.3 (1.63)	9.84 (3.82)
Vorinostat	Hypersensitivity	3	0.19 (0.06 - 0.58)	0.19 (10.72)	-2.43 (-4.09)	0.19 (0.07)
Vorinostat	Cardiomegaly	3	2.78 (0.9 - 8.62)	2.78 (3.41)	1.47 (-0.19)	2.78 (1.08)

Vorinostat	Lung Consolidation	3	12.69 (4.09 - 39.39)	12.68 (32.25)	3.66 (2)	12.67 (4.91)
Vorinostat	Skin Ulcer	3	1.32 (0.43 - 4.1)	1.32 (0.23)	0.4 (-1.26)	1.32 (0.51)
Vorinostat	Gastrooesophageal Reflux Disease	3	0.43 (0.14 - 1.34)	0.43 (2.25)	-1.21 (-2.88)	0.43 (0.17)
Vorinostat	Prothrombin Time Prolonged	3	6.2 (2 - 19.23)	6.19 (13.06)	2.63 (0.96)	6.19 (2.4)
Vorinostat	Wound Infection	3	3.74 (1.21 - 11.6)	3.74 (6.02)	1.9 (0.24)	3.74 (1.45)
Vorinostat	Bronchopulmonary Aspergillosis	3	4.63 (1.49 - 14.37)	4.63 (8.53)	2.21 (0.54)	4.63 (1.79)
Vorinostat	Haematemesis	3	1.37 (0.44 - 4.26)	1.37 (0.31)	0.46 (-1.21)	1.37 (0.53)
Vorinostat	Atrial Flutter	3	4.23 (1.36 - 13.14)	4.23 (7.4)	2.08 (0.41)	4.23 (1.64)
Vorinostat	Acute Graft Versus Host Disease In Intestine	3	25.45 (8.19 - 79.06)	25.44 (70.24)	4.67 (3)	25.37 (9.83)
Vorinostat	Treatment Noncompliance	3	0.71 (0.23 - 2.19)	0.71 (0.36)	-0.5 (-2.17)	0.71 (0.27)
Vorinostat	Hallucination	3	0.47 (0.15 - 1.47)	0.47 (1.74)	-1.07 (-2.74)	0.47 (0.18)
Vorinostat	Hyperthermia	3	4.57 (1.47 - 14.19)	4.57 (8.37)	2.19 (0.53)	4.57 (1.77)
Vorinostat	Brain Herniation	3	11.88 (3.83 - 36.87)	11.87 (29.84)	3.57 (1.9)	11.86 (4.6)
Vorinostat	Generalised Tonic-Clonic Seizure	3	1.39 (0.45 - 4.31)	1.39 (0.33)	0.47 (-1.19)	1.39 (0.54)
Vorinostat	Disseminated Intravascular Coagulation	3	2.49 (0.8 - 7.73)	2.49 (2.68)	1.32 (-0.35)	2.49 (0.97)
Vorinostat	Brain Natriuretic Peptide Increased	3	11.34 (3.65 - 35.18)	11.33 (28.22)	3.5 (1.83)	11.32 (4.39)
Vorinostat	Orthostatic Hypotension	3	1.99 (0.64 - 6.19)	1.99 (1.49)	1 (-0.67)	1.99 (0.77)
Vorinostat	Overdose	3	0.15 (0.05 - 0.46)	0.15 (14.61)	-2.74 (-4.41)	0.15 (0.06)
Vorinostat	Pulmonary Mass	3	2.33 (0.75 - 7.22)	2.33 (2.27)	1.22 (-0.45)	2.33 (0.9)
Vorinostat	Hyperuricaemia	3	8.41 (2.71 - 26.11)	8.41 (19.57)	3.07 (1.4)	8.4 (3.26)
Vorinostat	Haemolytic Anaemia	3	3.95 (1.27 - 12.26)	3.95 (6.61)	1.98 (0.32)	3.95 (1.53)
Vorinostat	Influenza	3	0.33 (0.11 - 1.02)	0.33 (4.14)	-1.61 (-3.27)	0.33 (0.13)
Vorinostat	Posterior Reversible Encephalopathy Syndrome	3	3.44 (1.11 - 10.67)	3.44 (5.19)	1.78 (0.11)	3.44 (1.33)
Vorinostat	Parotitis	3	30.96 (9.96 - 96.21)	30.94 (86.65)	4.95 (3.28)	30.85 (11.95)

Vorinostat	Product Packaging Quantity Issue	2	2.65 (0.66 - 10.58)	2.64 (2.05)	1.4 (-0.26)	2.64 (0.83)
Vorinostat	Interstitial Lung Disease	2	0.5 (0.12 - 2)	0.5 (1)	-1 (-2.67)	0.5 (0.16)
Vorinostat	Angina Pectoris	2	0.8 (0.2 - 3.21)	0.8 (0.1)	-0.32 (-1.99)	0.8 (0.25)
Vorinostat	Post Herpetic Neuralgia	2	15.47 (3.86 - 61.94)	15.46 (27.01)	3.95 (2.28)	15.44 (4.84)
Vorinostat	Pneumonia Viral	2	7.63 (1.91 - 30.54)	7.63 (11.51)	2.93 (1.26)	7.62 (2.39)
Vorinostat	Herpes Zoster Disseminated	2	31.04 (7.74 - 124.43)	31.03 (57.93)	4.95 (3.28)	30.93 (9.68)
Vorinostat	Tumour Haemorrhage	2	8.19 (2.05 - 32.76)	8.18 (12.6)	3.03 (1.36)	8.18 (2.56)
Vorinostat	Pneumonia Aspiration	2	0.94 (0.24 - 3.76)	0.94 (0.01)	-0.09 (-1.75)	0.94 (0.29)
Vorinostat	Hypernatraemia	2	4.72 (1.18 - 18.87)	4.71 (5.85)	2.24 (0.57)	4.71 (1.48)
Vorinostat	Colostomy	2	12.3 (3.07 - 49.24)	12.29 (20.73)	3.62 (1.95)	12.28 (3.85)
Vorinostat	Peripheral Ischaemia	2	5.64 (1.41 - 22.58)	5.64 (7.63)	2.5 (0.83)	5.64 (1.77)
Vorinostat	Kidney Infection	2	1.16 (0.29 - 4.64)	1.16 (0.04)	0.21 (-1.45)	1.16 (0.36)
Vorinostat	Pain Of Skin	2	0.81 (0.2 - 3.23)	0.81 (0.09)	-0.31 (-1.97)	0.81 (0.25)
Vorinostat	Cholelithiasis	2	0.7 (0.18 - 2.81)	0.7 (0.25)	-0.51 (-2.18)	0.7 (0.22)
Vorinostat	Progressive Multifocal Leukoencephalopathy	2	2.71 (0.68 - 10.85)	2.71 (2.16)	1.44 (-0.23)	2.71 (0.85)
Vorinostat	Mass	2	1.59 (0.4 - 6.36)	1.59 (0.44)	0.67 (-1)	1.59 (0.5)
Vorinostat	Rectal Haemorrhage	2	0.53 (0.13 - 2.11)	0.53 (0.85)	-0.93 (-2.59)	0.53 (0.17)
Vorinostat	Breast Cancer Metastatic	2	2.11 (0.53 - 8.43)	2.11 (1.16)	1.07 (-0.59)	2.11 (0.66)
Vorinostat	Swelling	2	0.2 (0.05 - 0.81)	0.2 (6.31)	-2.31 (-3.97)	0.2 (0.06)
Vorinostat	Encephalopathy	2	0.97 (0.24 - 3.89)	0.97 (0)	-0.04 (-1.7)	0.97 (0.31)
Vorinostat	Supraventricular Tachycardia	2	2.43 (0.61 - 9.72)	2.43 (1.68)	1.28 (-0.39)	2.43 (0.76)
Vorinostat	Large Intestinal Haemorrhage	2	10.21 (2.55 - 40.86)	10.2 (16.59)	3.35 (1.68)	10.19 (3.19)
Vorinostat	Ataxia	2	1.89 (0.47 - 7.55)	1.89 (0.83)	0.92 (-0.75)	1.89 (0.59)
Vorinostat	Cardiac Tamponade	2	4.87 (1.22 - 19.48)	4.87 (6.14)	2.28 (0.62)	4.87 (1.52)
Vorinostat	Cancer Pain	2	8.79 (2.2 - 35.19)	8.79 (13.79)	3.13 (1.47)	8.78 (2.75)
Vorinostat	Enteritis	2	3.59 (0.9 - 14.34)	3.58 (3.73)	1.84 (0.17)	3.58 (1.12)

Vorinostat	Large Intestine Perforation	2	3.33 (0.83 - 13.32)	3.33 (3.26)	1.73 (0.07)	3.33 (1.04)
Vorinostat	Blood Phosphorus Decreased	2	6.52 (1.63 - 26.1)	6.52 (9.34)	2.7 (1.04)	6.52 (2.04)
Vorinostat	Acute Febrile Neutrophilic Dermatosis	2	10.71 (2.68 - 42.86)	10.71 (17.58)	3.42 (1.75)	10.69 (3.35)
Vorinostat	Gastrointestinal Tube Insertion	2	9.54 (2.38 - 38.2)	9.54 (15.28)	3.25 (1.59)	9.53 (2.99)
Vorinostat	Human Herpesvirus 6 Infection	2	11.59 (2.9 - 46.4)	11.59 (19.33)	3.53 (1.87)	11.58 (3.63)
Vorinostat	Enterobacter Infection	2	14.72 (3.68 - 58.96)	14.72 (25.54)	3.88 (2.21)	14.7 (4.6)
Vorinostat	Tumour Compression	2	68.72 (17.1 - 276.22)	68.69 (132.45)	6.09 (4.42)	68.2 (21.29)
Vorinostat	Appetite Disorder	2	3.69 (0.92 - 14.77)	3.69 (3.92)	1.88 (0.22)	3.69 (1.16)
Vorinostat	Thirst	2	1.3 (0.33 - 5.22)	1.3 (0.14)	0.38 (-1.28)	1.3 (0.41)
Vorinostat	Herpes Virus Infection	2	4.01 (1 - 16.06)	4.01 (4.52)	2 (0.34)	4.01 (1.26)
Vorinostat	Left Ventricular Hypertrophy	2	5.02 (1.25 - 20.09)	5.02 (6.43)	2.33 (0.66)	5.02 (1.57)
Vorinostat	Brain Neoplasm	2	2.56 (0.64 - 10.23)	2.56 (1.9)	1.35 (-0.31)	2.56 (0.8)
Vorinostat	Body Temperature Decreased	2	2.17 (0.54 - 8.67)	2.17 (1.26)	1.12 (-0.55)	2.17 (0.68)
Vorinostat	Pneumonia Bacterial	2	2.77 (0.69 - 11.08)	2.77 (2.26)	1.47 (-0.2)	2.77 (0.87)
Vorinostat	Faeces Discoloured	2	1.05 (0.26 - 4.2)	1.05 (0)	0.07 (-1.59)	1.05 (0.33)
Vorinostat	Intestinal Obstruction	2	0.64 (0.16 - 2.58)	0.64 (0.39)	-0.63 (-2.3)	0.64 (0.2)
Vorinostat	Appendicitis	2	2.55 (0.64 - 10.22)	2.55 (1.89)	1.35 (-0.31)	2.55 (0.8)
Vorinostat	Streptococcal Bacteraemia	2	23.53 (5.87 - 94.27)	23.52 (43.02)	4.55 (2.88)	23.47 (7.35)
Vorinostat	Skin Fissures	2	1.48 (0.37 - 5.94)	1.48 (0.32)	0.57 (-1.1)	1.48 (0.47)
Vorinostat	Frequent Bowel Movements	2	0.91 (0.23 - 3.64)	0.91 (0.02)	-0.14 (-1.8)	0.91 (0.29)
Vorinostat	Pollakiuria	2	0.55 (0.14 - 2.22)	0.55 (0.72)	-0.85 (-2.52)	0.55 (0.17)
Vorinostat	Product Storage Error	2	0.27 (0.07 - 1.08)	0.27 (3.92)	-1.88 (-3.55)	0.27 (0.09)
Vorinostat	Drug Effective For Unapproved Indication	2	1.09 (0.27 - 4.35)	1.09 (0.01)	0.12 (-1.55)	1.09 (0.34)
Vorinostat	Chronic Kidney Disease	2	0.24 (0.06 - 0.96)	0.24 (4.79)	-2.05 (-3.72)	0.24 (0.08)
Vorinostat	Coma	2	0.51 (0.13 - 2.04)	0.51 (0.94)	-0.97 (-2.64)	0.51 (0.16)

Vorinostat	Metastases To Central Nervous System	2	1.93 (0.48 - 7.72)	1.93 (0.9)	0.95 (-0.72)	1.93 (0.6)
Vorinostat	Haematotoxicity	2	2.76 (0.69 - 11.03)	2.76 (2.24)	1.46 (-0.2)	2.76 (0.86)
Vorinostat	Inappropriate Schedule Of Product Administration	2	0.1 (0.02 - 0.39)	0.1 (16.57)	-3.34 (-5.01)	0.1 (0.03)
Vorinostat	Adult T-Cell Lymphoma/Leukaemia	2	46.15 (11.5 - 185.18)	46.13 (87.87)	5.52 (3.85)	45.91 (14.35)
Vorinostat	Transient Ischaemic Attack	2	0.68 (0.17 - 2.7)	0.68 (0.31)	-0.57 (-2.23)	0.68 (0.21)
Vorinostat	Body Temperature Increased	2	1.12 (0.28 - 4.48)	1.12 (0.03)	0.16 (-1.5)	1.12 (0.35)
Vorinostat	Discomfort	2	0.37 (0.09 - 1.47)	0.37 (2.17)	-1.44 (-3.11)	0.37 (0.12)
Vorinostat	Pulse Absent	2	5 (1.25 - 20.02)	5 (6.4)	2.32 (0.65)	5 (1.57)
Vorinostat	Eating Disorder	2	1.05 (0.26 - 4.21)	1.05 (0.01)	0.07 (-1.59)	1.05 (0.33)
Vorinostat	Sputum Culture Positive	2	31.3 (7.81 - 125.46)	31.29 (58.44)	4.96 (3.29)	31.19 (9.76)
Vorinostat	Pericarditis	2	1.67 (0.42 - 6.68)	1.67 (0.54)	0.74 (-0.93)	1.67 (0.52)
Vorinostat	Dermatitis	2	1.21 (0.3 - 4.84)	1.21 (0.07)	0.27 (-1.39)	1.21 (0.38)
Vorinostat	Mean Cell Volume Increased	2	9.55 (2.39 - 38.24)	9.55 (15.3)	3.25 (1.59)	9.54 (2.99)
Vorinostat	Rales	2	3.91 (0.98 - 15.64)	3.91 (4.33)	1.97 (0.3)	3.91 (1.22)
Vorinostat	Ventricular Extrasystoles	2	2.26 (0.57 - 9.05)	2.26 (1.41)	1.18 (-0.49)	2.26 (0.71)
Vorinostat	Coagulopathy	2	1.38 (0.34 - 5.51)	1.38 (0.21)	0.46 (-1.2)	1.38 (0.43)
Vorinostat	Nephrolithiasis	2	0.51 (0.13 - 2.05)	0.51 (0.92)	-0.96 (-2.63)	0.51 (0.16)
Vorinostat	Renal Cyst	2	2.81 (0.7 - 11.26)	2.81 (2.34)	1.49 (-0.17)	2.81 (0.88)
Vorinostat	Activated Partial Thromboplastin Time Shortened	2	61.58 (15.33 - 247.39)	61.55 (118.37)	5.93 (4.26)	61.16 (19.1)
Vorinostat	Refusal Of Treatment By Patient	2	3.84 (0.96 - 15.38)	3.84 (4.21)	1.94 (0.28)	3.84 (1.2)
Vorinostat	Anal Incontinence	2	1.7 (0.43 - 6.82)	1.7 (0.58)	0.77 (-0.9)	1.7 (0.53)
Vorinostat	Nocturia	2	1.97 (0.49 - 7.9)	1.97 (0.96)	0.98 (-0.69)	1.97 (0.62)
Vorinostat	Carbon Dioxide Decreased	2	21.67 (5.41 - 86.83)	21.67 (39.34)	4.43 (2.77)	21.62 (6.77)

Vorinostat	Pallor	2	0.83 (0.21 - 3.34)	0.83 (0.07)	-0.26 (-1.93)	0.83 (0.26)
Vorinostat	Diabetic Ketoacidosis	2	0.95 (0.24 - 3.79)	0.95 (0.01)	-0.08 (-1.75)	0.95 (0.3)
Vorinostat	Lip Swelling	2	0.67 (0.17 - 2.69)	0.67 (0.32)	-0.57 (-2.24)	0.67 (0.21)
Vorinostat	Electrocardiogram St Segment Depression	2	10.62 (2.65 - 42.5)	10.62 (17.4)	3.41 (1.74)	10.6 (3.32)
Vorinostat	Urinary Hesitation	2	9.73 (2.43 - 38.95)	9.73 (15.64)	3.28 (1.61)	9.72 (3.05)
Vorinostat	Conjunctivitis	2	1.33 (0.33 - 5.3)	1.33 (0.16)	0.41 (-1.26)	1.33 (0.42)
Vorinostat	Hypermagnesaemia	2	37.26 (9.29 - 149.43)	37.25 (70.27)	5.21 (3.54)	37.11 (11.61)
Vorinostat	Metastases To Lymph Nodes	2	3.68 (0.92 - 14.72)	3.68 (3.9)	1.88 (0.21)	3.68 (1.15)
Vorinostat	Bacterial Sepsis	2	7.39 (1.85 - 29.55)	7.38 (11.03)	2.88 (1.22)	7.38 (2.31)
Vorinostat	Metastatic Neoplasm	2	5.62 (1.4 - 22.48)	5.62 (7.58)	2.49 (0.82)	5.61 (1.76)
Vorinostat	Bone Metabolism Disorder	2	75.56 (18.79 - 303.88)	75.53 (145.93)	6.23 (4.55)	74.94 (23.39)
Vorinostat	Vaginal Haemorrhage	2	0.51 (0.13 - 2.02)	0.51 (0.97)	-0.98 (-2.65)	0.51 (0.16)
Vorinostat	Drug Level Increased	2	1.44 (0.36 - 5.76)	1.44 (0.27)	0.52 (-1.14)	1.44 (0.45)
Vorinostat	Mood Altered	2	0.85 (0.21 - 3.38)	0.85 (0.06)	-0.24 (-1.91)	0.85 (0.27)
Vorinostat	Ageusia	2	0.91 (0.23 - 3.66)	0.91 (0.02)	-0.13 (-1.8)	0.91 (0.29)
Vorinostat	Cyst	2	1.81 (0.45 - 7.26)	1.81 (0.73)	0.86 (-0.81)	1.81 (0.57)
Vorinostat	Thrombocytosis	2	5.69 (1.42 - 22.77)	5.69 (7.73)	2.51 (0.84)	5.69 (1.78)
Vorinostat	Bundle Branch Block Right	2	5 (1.25 - 19.99)	5 (6.39)	2.32 (0.65)	4.99 (1.57)
Vorinostat	Urinary Tract Obstruction	2	8.09 (2.02 - 32.37)	8.08 (12.41)	3.01 (1.35)	8.08 (2.53)
Vorinostat	Adverse Drug Reaction	2	0.27 (0.07 - 1.1)	0.27 (3.84)	-1.87 (-3.53)	0.27 (0.09)
Vorinostat	Tumour Necrosis	2	21.87 (5.46 - 87.63)	21.87 (39.73)	4.45 (2.78)	21.82 (6.83)
Vorinostat	Granuloma	2	6.91 (1.73 - 27.67)	6.91 (10.11)	2.79 (1.12)	6.91 (2.16)
Vorinostat	Skin Necrosis	2	4.38 (1.09 - 17.52)	4.38 (5.21)	2.13 (0.46)	4.38 (1.37)
Vorinostat	Superinfection Bacterial	2	32.7 (8.16 - 131.1)	32.69 (61.23)	5.03 (3.36)	32.58 (10.19)
Vorinostat	Nail Discolouration	2	5.79 (1.45 - 23.15)	5.78 (7.91)	2.53 (0.86)	5.78 (1.81)
Vorinostat	Acute Pulmonary Oedema	2	4.18 (1.04 - 16.7)	4.17 (4.83)	2.06 (0.39)	4.17 (1.31)

Vorinostat	Blood Pressure Diastolic Increased	2	5 (1.25 - 19.99)	5 (6.39)	2.32 (0.65)	4.99 (1.57)
Vorinostat	Shock	2	1.09 (0.27 - 4.34)	1.09 (0.01)	0.12 (-1.55)	1.09 (0.34)
Vorinostat	Coronary Artery Disease	2	0.71 (0.18 - 2.83)	0.71 (0.24)	-0.5 (-2.17)	0.71 (0.22)
Vorinostat	Mastoiditis	2	25.73 (6.42 - 103.12)	25.72 (47.4)	4.68 (3.01)	25.66 (8.03)
Vorinostat	Staphylococcal Skin Infection	2	17.77 (4.44 - 71.18)	17.77 (31.59)	4.15 (2.48)	17.74 (5.55)
Vorinostat	Abscess	2	1.43 (0.36 - 5.71)	1.43 (0.26)	0.51 (-1.15)	1.43 (0.45)
Vorinostat	Electrocardiogram T Wave Inversion	2	10.61 (2.65 - 42.48)	10.61 (17.39)	3.41 (1.74)	10.6 (3.32)
Vorinostat	Peripheral Motor Neuropathy	2	16.41 (4.1 - 65.7)	16.4 (28.87)	4.03 (2.36)	16.37 (5.13)
Vorinostat	Non-Hodgkin'S Lymphoma	2	3.76 (0.94 - 15.06)	3.76 (4.06)	1.91 (0.24)	3.76 (1.18)
Vorinostat	Phlebitis	2	4.37 (1.09 - 17.47)	4.37 (5.19)	2.13 (0.46)	4.36 (1.37)
Vorinostat	Hiccups	2	2.94 (0.73 - 11.76)	2.94 (2.56)	1.56 (-0.11)	2.94 (0.92)
Vorinostat	Musculoskeletal Pain	2	0.39 (0.1 - 1.56)	0.39 (1.9)	-1.35 (-3.02)	0.39 (0.12)
Vorinostat	Anuria	2	2.68 (0.67 - 10.73)	2.68 (2.11)	1.42 (-0.24)	2.68 (0.84)
Vorinostat	Procedural Complication	2	3.22 (0.8 - 12.86)	3.21 (3.05)	1.68 (0.02)	3.21 (1.01)
Vorinostat	Wheezing	2	0.42 (0.1 - 1.68)	0.42 (1.61)	-1.25 (-2.92)	0.42 (0.13)
Vorinostat	Procedural Pain	2	0.85 (0.21 - 3.4)	0.85 (0.05)	-0.24 (-1.9)	0.85 (0.27)
Vorinostat	Metastases To Meninges	2	11.27 (2.82 - 45.11)	11.26 (18.69)	3.49 (1.82)	11.25 (3.53)
Vorinostat	Wound	2	0.84 (0.21 - 3.38)	0.84 (0.06)	-0.24 (-1.91)	0.84 (0.26)
Vorinostat	Rib Fracture	2	1.1 (0.28 - 4.41)	1.1 (0.02)	0.14 (-1.52)	1.1 (0.35)
Vorinostat	Lung Neoplasm	2	5.32 (1.33 - 21.3)	5.32 (7.02)	2.41 (0.74)	5.32 (1.67)
Vorinostat	Cardiopulmonary Failure	2	5.72 (1.43 - 22.88)	5.72 (7.78)	2.51 (0.85)	5.71 (1.79)
Vorinostat	Blood Urine Present	2	1.16 (0.29 - 4.64)	1.16 (0.04)	0.22 (-1.45)	1.16 (0.36)
Vorinostat	Dialysis	2	1.71 (0.43 - 6.84)	1.71 (0.59)	0.77 (-0.89)	1.71 (0.54)
Vorinostat	Pleurisy	2	3.73 (0.93 - 14.94)	3.73 (4)	1.9 (0.23)	3.73 (1.17)
Vorinostat	Toxic Skin Eruption	2	2.36 (0.59 - 9.46)	2.36 (1.57)	1.24 (-0.43)	2.36 (0.74)
Vorinostat	Diastolic Dysfunction	2	7.56 (1.89 - 30.25)	7.56 (11.37)	2.92 (1.25)	7.55 (2.37)

Vorinostat	Pathological Fracture	2	4.52 (1.13 - 18.07)	4.51 (5.47)	2.17 (0.51)	4.51 (1.41)
Vorinostat	Flatulence	2	0.41 (0.1 - 1.64)	0.41 (1.69)	-1.28 (-2.95)	0.41 (0.13)
Vorinostat	Intestinal Ischaemia	2	4.3 (1.07 - 17.19)	4.29 (5.05)	2.1 (0.43)	4.29 (1.35)
Vorinostat	Transfusion Reaction	2	28.65 (7.15 - 114.82)	28.64 (53.19)	4.84 (3.17)	28.56 (8.94)
Vorinostat	Atrial Tachycardia	2	10.93 (2.73 - 43.75)	10.93 (18.02)	3.45 (1.78)	10.92 (3.42)
Vorinostat	Blood Chloride Increased	2	15.56 (3.89 - 62.3)	15.55 (27.19)	3.96 (2.29)	15.53 (4.86)
Vorinostat	Sialoadenitis	2	17.12 (4.27 - 68.55)	17.11 (30.28)	4.09 (2.43)	17.08 (5.35)
Vorinostat	Underdose	2	0.31 (0.08 - 1.24)	0.31 (3.07)	-1.69 (-3.35)	0.31 (0.1)
Vorinostat	Head Injury	2	0.74 (0.18 - 2.94)	0.74 (0.19)	-0.44 (-2.11)	0.74 (0.23)
Vorinostat	Orthopnoea	2	7.02 (1.75 - 28.09)	7.02 (10.31)	2.81 (1.14)	7.01 (2.2)
Vorinostat	Cytomegalovirus Infection	2	1.42 (0.35 - 5.68)	1.42 (0.25)	0.5 (-1.16)	1.42 (0.44)
Vorinostat	Effusion	2	16.89 (4.22 - 67.63)	16.88 (29.83)	4.08 (2.41)	16.85 (5.28)
Vorinostat	Osteoporosis	2	0.53 (0.13 - 2.13)	0.53 (0.82)	-0.91 (-2.57)	0.53 (0.17)
Vorinostat	Early Satiety	2	12.79 (3.19 - 51.2)	12.78 (21.7)	3.67 (2.01)	12.77 (4)
Vorinostat	Inflammation	2	0.48 (0.12 - 1.9)	0.48 (1.15)	-1.07 (-2.74)	0.48 (0.15)
Vorinostat	Candida Infection	2	1.15 (0.29 - 4.59)	1.15 (0.04)	0.2 (-1.47)	1.15 (0.36)
Vorinostat	Cachexia	2	4.43 (1.11 - 17.73)	4.43 (5.31)	2.15 (0.48)	4.43 (1.39)
Vorinostat	Troponin Increased	2	3.43 (0.86 - 13.7)	3.42 (3.43)	1.78 (0.11)	3.42 (1.07)
Vorinostat	Gastric Ulcer	2	1.2 (0.3 - 4.8)	1.2 (0.07)	0.26 (-1.4)	1.2 (0.38)
Vorinostat	Skin Laceration	2	1.1 (0.28 - 4.4)	1.1 (0.02)	0.14 (-1.53)	1.1 (0.35)
Vorinostat	Oxygen Saturation Decreased	2	0.44 (0.11 - 1.76)	0.44 (1.43)	-1.18 (-2.85)	0.44 (0.14)
Vorinostat	Venous Thrombosis	2	5.62 (1.4 - 22.47)	5.61 (7.58)	2.49 (0.82)	5.61 (1.76)
Vorinostat	Cardiovascular Disorder	2	0.89 (0.22 - 3.57)	0.89 (0.03)	-0.16 (-1.83)	0.89 (0.28)
Vorinostat	Blood Potassium Increased	2	1.45 (0.36 - 5.82)	1.45 (0.28)	0.54 (-1.13)	1.45 (0.46)
Vorinostat	Femur Fracture	2	0.69 (0.17 - 2.78)	0.7 (0.27)	-0.52 (-2.19)	0.7 (0.22)
Vorinostat	Haemothorax	2	7.5 (1.87 - 30.02)	7.5 (11.26)	2.91 (1.24)	7.49 (2.35)
Vorinostat	Polyuria	2	2.83 (0.71 - 11.3)	2.83 (2.36)	1.5 (-0.17)	2.82 (0.89)

Vorinostat	Neuroblastoma	2	63.22 (15.73 - 254.02)	63.2 (121.61)	5.97 (4.3)	62.78 (19.61)
Vorinostat	Febrile Infection	2	20.77 (5.19 - 83.21)	20.77 (37.55)	4.37 (2.7)	20.72 (6.49)
Vorinostat	Cerebral Haemorrhage	2	0.64 (0.16 - 2.56)	0.64 (0.41)	-0.65 (-2.31)	0.64 (0.2)
Vorinostat	Scar	2	1.33 (0.33 - 5.31)	1.33 (0.16)	0.41 (-1.26)	1.33 (0.42)
Vorinostat	Apnoea	2	2.96 (0.74 - 11.84)	2.96 (2.59)	1.56 (-0.1)	2.96 (0.93)
Vorinostat	Immune System Disorder	2	1.76 (0.44 - 7.03)	1.76 (0.65)	0.81 (-0.85)	1.76 (0.55)
Vorinostat	Embolism Venous	2	7.56 (1.89 - 30.27)	7.56 (11.38)	2.92 (1.25)	7.56 (2.37)
Vorinostat	Crohn'S Disease	2	0.36 (0.09 - 1.45)	0.36 (2.24)	-1.46 (-3.13)	0.36 (0.11)
Vorinostat	Drowning	2	23.24 (5.8 - 93.12)	23.23 (42.45)	4.53 (2.87)	23.18 (7.26)
Vorinostat	Lipase Increased	2	3.07 (0.77 - 12.29)	3.07 (2.79)	1.62 (-0.05)	3.07 (0.96)
Vorinostat	Breath Sounds Abnormal	2	4.71 (1.18 - 18.86)	4.71 (5.85)	2.24 (0.57)	4.71 (1.48)
Vorinostat	Hypercreatinaemia	2	67.98 (16.91 - 273.23)	67.95 (131)	6.08 (4.4)	67.48 (21.07)
Vorinostat	Cognitive Disorder	2	0.49 (0.12 - 1.96)	0.49 (1.06)	-1.03 (-2.7)	0.49 (0.15)
Vorinostat	Neck Pain	2	0.41 (0.1 - 1.63)	0.41 (1.71)	-1.29 (-2.96)	0.41 (0.13)
Vorinostat	Superficial Inflammatory Dermatosis	2	50.44 (12.57 - 202.49)	50.42 (96.37)	5.65 (3.98)	50.16 (15.68)
Vorinostat	Enterocolitis Infectious	2	22.96 (5.73 - 91.99)	22.95 (41.89)	4.52 (2.85)	22.9 (7.17)
Vorinostat	Melanocytic Naevus	2	4.97 (1.24 - 19.9)	4.97 (6.34)	2.31 (0.65)	4.97 (1.56)
Vorinostat	Blood Blister	2	7.13 (1.78 - 28.52)	7.12 (10.52)	2.83 (1.16)	7.12 (2.23)
Vorinostat	Tongue Haemorrhage	2	20.68 (5.16 - 82.84)	20.67 (37.37)	4.37 (2.7)	20.63 (6.46)
Vorinostat	Varicella	2	9.76 (2.44 - 39.07)	9.76 (15.7)	3.29 (1.62)	9.75 (3.05)
Vorinostat	Myocardial Necrosis Marker Increased	2	10.62 (2.65 - 42.53)	10.62 (17.41)	3.41 (1.74)	10.61 (3.32)
Vorinostat	Cerebral Infarction	2	0.94 (0.23 - 3.74)	0.94 (0.01)	-0.1 (-1.76)	0.94 (0.29)
Vorinostat	Upper Gastrointestinal Haemorrhage	2	1.32 (0.33 - 5.28)	1.32 (0.15)	0.4 (-1.27)	1.32 (0.41)
Vorinostat	Haematoma	1	0.43 (0.06 - 3.02)	0.43 (0.77)	-1.23 (-2.9)	0.43 (0.08)

Vorinostat	Renal Colic	1	4.93 (0.69 - 35.01)	4.93 (3.13)	2.3 (0.63)	4.92 (0.95)
Vorinostat	Infestation	1	47.17 (6.61 - 336.57)	47.16 (44.96)	5.55 (3.87)	46.93 (9.07)
Vorinostat	Metapneumovirus Infection	1	17.66 (2.48 - 125.59)	17.65 (15.68)	4.14 (2.47)	17.62 (3.41)
Vorinostat	Pneumonia Streptococcal	1	11.14 (1.57 - 79.2)	11.14 (9.22)	3.48 (1.81)	11.13 (2.16)
Vorinostat	Sepsis Syndrome	1	21.35 (3 - 151.96)	21.35 (19.35)	4.41 (2.74)	21.3 (4.12)
Vorinostat	Colon Cancer Metastatic	1	7.34 (1.03 - 52.15)	7.34 (5.47)	2.87 (1.21)	7.33 (1.42)
Vorinostat	Cushingoid	1	3.83 (0.54 - 27.18)	3.83 (2.09)	1.94 (0.27)	3.82 (0.74)
Vorinostat	Parainfluenzae Virus Infection	1	7.59 (1.07 - 53.94)	7.59 (5.72)	2.92 (1.25)	7.58 (1.47)
Vorinostat	Asphyxia	1	1.36 (0.19 - 9.62)	1.36 (0.09)	0.44 (-1.23)	1.36 (0.26)
Vorinostat	Intestinal Dilatation	1	9.16 (1.29 - 65.11)	9.16 (7.26)	3.19 (1.53)	9.15 (1.77)
Vorinostat	Presyncope	1	0.47 (0.07 - 3.33)	0.47 (0.6)	-1.09 (-2.76)	0.47 (0.09)
Vorinostat	Urosepsis	1	1.25 (0.18 - 8.9)	1.25 (0.05)	0.33 (-1.34)	1.25 (0.24)
Vorinostat	Blood Creatine Increased	1	2.73 (0.38 - 19.4)	2.73 (1.1)	1.45 (-0.22)	2.73 (0.53)
Vorinostat	Vulval Ulceration	1	41.95 (5.88 - 299.18)	41.94 (39.79)	5.38 (3.71)	41.76 (8.07)
Vorinostat	Anorectal Infection	1	44.72 (6.27 - 319.03)	44.71 (42.54)	5.48 (3.8)	44.51 (8.6)
Vorinostat	Pleural Mesothelioma Malignant	1	92.95 (12.97 - 666.38)	92.93 (90.06)	6.52 (4.83)	92.04 (17.71)
Vorinostat	Embolism Arterial	1	9.14 (1.29 - 64.98)	9.14 (7.24)	3.19 (1.52)	9.13 (1.77)
Vorinostat	Spinal Cord Compression	1	2.89 (0.41 - 20.54)	2.89 (1.24)	1.53 (-0.14)	2.89 (0.56)
Vorinostat	Ileostomy	1	5.26 (0.74 - 37.4)	5.26 (3.45)	2.4 (0.73)	5.26 (1.02)
Vorinostat	Peripheral Vascular Disorder	1	2.09 (0.29 - 14.85)	2.09 (0.57)	1.06 (-0.6)	2.09 (0.41)
Vorinostat	Sputum Retention	1	13.3 (1.87 - 94.55)	13.3 (11.35)	3.73 (2.06)	13.28 (2.57)
Vorinostat	Eosinophilic Cellulitis	1	55.45 (7.76 - 395.95)	55.43 (53.14)	5.78 (4.1)	55.12 (10.64)
Vorinostat	Heart Rate Irregular	1	0.49 (0.07 - 3.47)	0.49 (0.53)	-1.03 (-2.7)	0.49 (0.09)
Vorinostat	Hepatic Cirrhosis	1	0.66 (0.09 - 4.66)	0.66 (0.18)	-0.61 (-2.28)	0.66 (0.13)
Vorinostat	Partial Seizures	1	2.27 (0.32 - 16.15)	2.27 (0.71)	1.19 (-0.48)	2.27 (0.44)
Vorinostat	Social Stay Hospitalisation	1	185.9 (25.69 - 1345.52)	185.87 (180.34)	7.51 (5.8)	182.31 (34.8)

Vorinostat	Fungaemia	1	7.87 (1.11 - 55.96)	7.87 (6)	2.98 (1.31)	7.87 (1.52)
Vorinostat	Optic Nerve Disorder	1	7.22 (1.02 - 51.31)	7.22 (5.35)	2.85 (1.18)	7.21 (1.4)
Vorinostat	Klebsiella Bacteraemia	1	13.94 (1.96 - 99.14)	13.94 (11.99)	3.8 (2.13)	13.92 (2.7)
Vorinostat	Tardive Dyskinesia	1	0.36 (0.05 - 2.57)	0.36 (1.12)	-1.46 (-3.13)	0.36 (0.07)
Vorinostat	Tongue Movement Disturbance	1	13.06 (1.84 - 92.85)	13.06 (11.12)	3.7 (2.04)	13.04 (2.53)
Vorinostat	Central Venous Catheterisation	1	3.69 (0.52 - 26.23)	3.69 (1.96)	1.88 (0.22)	3.69 (0.72)
Vorinostat	Radiation Necrosis	1	24.56 (3.45 - 174.85)	24.56 (22.54)	4.61 (2.94)	24.5 (4.74)
Vorinostat	Ovarian Epithelial Cancer Stage Iii	1	948.11 (121.34 - 7408)	947.94 (859.94)	9.75 (7.89)	861.85 (154.3)
Vorinostat	Gastroenteritis Salmonella	1	15.37 (2.16 - 109.28)	15.36 (13.41)	3.94 (2.27)	15.34 (2.97)
Vorinostat	Salmonella Sepsis	1	50.97 (7.14 - 363.85)	50.96 (48.72)	5.66 (3.98)	50.7 (9.79)
Vorinostat	Tracheal Stenosis	1	24.12 (3.39 - 171.73)	24.12 (22.11)	4.59 (2.92)	24.06 (4.66)
Vorinostat	Breast Cancer Stage Iv	1	3.47 (0.49 - 24.68)	3.47 (1.76)	1.8 (0.13)	3.47 (0.67)
Vorinostat	Breast Disorder	1	5.44 (0.77 - 38.62)	5.44 (3.62)	2.44 (0.77)	5.43 (1.05)
Vorinostat	Pelvic Pain	1	0.63 (0.09 - 4.44)	0.63 (0.22)	-0.68 (-2.34)	0.63 (0.12)
Vorinostat	Reproductive Tract Disorder	1	26.41 (3.71 - 188.04)	26.4 (24.38)	4.72 (3.05)	26.33 (5.1)
Vorinostat	Acute Myeloid Leukaemia Recurrent	1	6.32 (0.89 - 44.94)	6.32 (4.48)	2.66 (0.99)	6.32 (1.23)
Vorinostat	Intervertebral Discitis	1	6.38 (0.9 - 45.36)	6.38 (4.54)	2.67 (1.01)	6.38 (1.24)
Vorinostat	Cholestasis	1	0.64 (0.09 - 4.52)	0.64 (0.21)	-0.65 (-2.32)	0.64 (0.12)
Vorinostat	Hepatic Steatosis	1	0.64 (0.09 - 4.57)	0.64 (0.2)	-0.63 (-2.3)	0.64 (0.12)
Vorinostat	Systemic Mycosis	1	11 (1.55 - 78.19)	11 (9.08)	3.46 (1.79)	10.99 (2.13)
Vorinostat	Breath Odour	1	4.6 (0.65 - 32.68)	4.6 (2.82)	2.2 (0.53)	4.6 (0.89)
Vorinostat	Epstein-Barr Virus Infection	1	1.95 (0.27 - 13.86)	1.95 (0.46)	0.96 (-0.7)	1.95 (0.38)
Vorinostat	Liver Function Test Increased	1	0.59 (0.08 - 4.22)	0.59 (0.28)	-0.75 (-2.42)	0.59 (0.12)
Vorinostat	Ocular Icterus	1	2.66 (0.37 - 18.9)	2.66 (1.04)	1.41 (-0.26)	2.66 (0.52)
Vorinostat	Alkalosis	1	33.98 (4.77 - 242.14)	33.98 (31.89)	5.08 (3.41)	33.86 (6.55)

Vorinostat	Cellulitis Pharyngeal	1	474.06 (63.61 - 3533.02)	473.97 (449.5)	8.82 (7.04)	451.45 (84.08)
Vorinostat	Monoparesis	1	6.17 (0.87 - 43.86)	6.17 (4.33)	2.62 (0.96)	6.17 (1.2)
Vorinostat	Troponin T Increased	1	9.37 (1.32 - 66.59)	9.37 (7.47)	3.23 (1.56)	9.36 (1.81)
Vorinostat	Pleuritic Pain	1	4.22 (0.59 - 29.99)	4.22 (2.46)	2.08 (0.41)	4.22 (0.82)
Vorinostat	Urinary Tract Infection Enterococcal	1	11.99 (1.69 - 85.21)	11.98 (10.05)	3.58 (1.91)	11.97 (2.32)
Vorinostat	Urinary Tract Infection Pseudomonal	1	19.92 (2.8 - 141.72)	19.91 (17.93)	4.31 (2.64)	19.87 (3.85)
Vorinostat	Hypogammaglobulinaemia	1	2.2 (0.31 - 15.65)	2.2 (0.66)	1.14 (-0.53)	2.2 (0.43)
Vorinostat	Neutropenic Colitis	1	6.17 (0.87 - 43.83)	6.17 (4.33)	2.62 (0.96)	6.16 (1.19)
Vorinostat	Nutritional Condition Abnormal	1	16.49 (2.32 - 117.28)	16.49 (14.52)	4.04 (2.37)	16.46 (3.19)
Vorinostat	Gastrointestinal Pain	1	1.08 (0.15 - 7.65)	1.08 (0.01)	0.11 (-1.56)	1.08 (0.21)
Vorinostat	Nystagmus	1	2.15 (0.3 - 15.3)	2.15 (0.62)	1.11 (-0.56)	2.15 (0.42)
Vorinostat	Erythema Multiforme	1	1.27 (0.18 - 9.03)	1.27 (0.06)	0.35 (-1.32)	1.27 (0.25)
Vorinostat	Amylase Increased	1	2.2 (0.31 - 15.6)	2.2 (0.65)	1.13 (-0.53)	2.2 (0.43)
Vorinostat	Respiratory Arrest	1	0.41 (0.06 - 2.89)	0.41 (0.86)	-1.3 (-2.96)	0.41 (0.08)
Vorinostat	Skin Reaction	1	0.83 (0.12 - 5.91)	0.83 (0.03)	-0.26 (-1.93)	0.83 (0.16)
Vorinostat	Rash Erythematous	1	0.28 (0.04 - 1.98)	0.28 (1.86)	-1.84 (-3.51)	0.28 (0.05)
Vorinostat	Nephropathy Toxic	1	1.12 (0.16 - 7.95)	1.12 (0.01)	0.16 (-1.5)	1.12 (0.22)
Vorinostat	Subarachnoid Haemorrhage	1	1.1 (0.16 - 7.83)	1.1 (0.01)	0.14 (-1.53)	1.1 (0.21)
Vorinostat	Drug Reaction With Eosinophilia And Systemic Symptoms	1	0.41 (0.06 - 2.88)	0.41 (0.87)	-1.3 (-2.97)	0.41 (0.08)
Vorinostat	Myelosuppression	1	0.6 (0.08 - 4.26)	0.6 (0.27)	-0.74 (-2.4)	0.6 (0.12)
Vorinostat	Feeling Drunk	1	1.5 (0.21 - 10.63)	1.5 (0.16)	0.58 (-1.09)	1.5 (0.29)
Vorinostat	Product Preparation Error	1	0.67 (0.1 - 4.79)	0.67 (0.16)	-0.57 (-2.23)	0.67 (0.13)
Vorinostat	Colonic Abscess	1	13.47 (1.89 - 95.76)	13.47 (11.52)	3.75 (2.08)	13.45 (2.61)

Vorinostat	Surgery	1	0.22 (0.03 - 1.56)	0.22 (2.78)	-2.19 (-3.86)	0.22 (0.04)
Vorinostat	Epigastric Discomfort	1	2.89 (0.41 - 20.55)	2.89 (1.24)	1.53 (-0.13)	2.89 (0.56)
Vorinostat	Peripheral T-Cell Lymphoma Unspecified	1	20.79 (2.92 - 147.95)	20.79 (18.8)	4.37 (2.7)	20.74 (4.02)
Vorinostat	Smooth Muscle Cell Neoplasm	1	37.47 (5.26 - 267.13)	37.47 (35.35)	5.22 (3.55)	37.32 (7.22)
Vorinostat	Pneumonia Influenzal	1	11.49 (1.62 - 81.7)	11.49 (9.57)	3.52 (1.85)	11.48 (2.22)
Vorinostat	Peripheral T-Cell Lymphoma Unspecified Recurrent	1	178.89 (24.73 - 1293.82)	178.86 (173.59)	7.46 (5.74)	175.56 (33.53)
Vorinostat	Pharyngeal Haemorrhage	1	9.3 (1.31 - 66.06)	9.29 (7.39)	3.21 (1.55)	9.29 (1.8)
Vorinostat	Epilepsy	1	0.39 (0.06 - 2.8)	0.39 (0.93)	-1.34 (-3.01)	0.39 (0.08)
Vorinostat	Coronavirus Infection	1	1.54 (0.22 - 10.91)	1.54 (0.19)	0.62 (-1.05)	1.54 (0.3)
Vorinostat	Tooth Loss	1	0.51 (0.07 - 3.6)	0.51 (0.48)	-0.98 (-2.65)	0.51 (0.1)
Vorinostat	Peripheral Swelling	1	0.07 (0.01 - 0.53)	0.07 (11.48)	-3.74 (-5.41)	0.07 (0.01)
Vorinostat	Skin Weeping	1	6.48 (0.91 - 46.08)	6.48 (4.64)	2.7 (1.03)	6.48 (1.26)
Vorinostat	Thoracic Haemorrhage	1	54.49 (7.63 - 389.09)	54.48 (52.2)	5.76 (4.08)	54.17 (10.46)
Vorinostat	Eye Ulcer	1	20.39 (2.87 - 145.08)	20.39 (18.4)	4.35 (2.67)	20.34 (3.94)
Vorinostat	Completed Suicide	1	0.14 (0.02 - 0.98)	0.14 (5.41)	-2.86 (-4.53)	0.14 (0.03)
Vorinostat	Malignant Neoplasm Of Spinal Cord	1	64.94 (9.08 - 464.21)	64.93 (62.51)	6.01 (4.33)	64.49 (12.44)
Vorinostat	Hypercalcaemia Of Malignancy	1	46.02 (6.45 - 328.36)	46.02 (43.83)	5.52 (3.84)	45.8 (8.85)
Vorinostat	Product Administration Error	1	0.21 (0.03 - 1.51)	0.21 (2.9)	-2.23 (-3.9)	0.21 (0.04)
Vorinostat	Tubulointerstitial Nephritis	1	0.58 (0.08 - 4.13)	0.58 (0.3)	-0.78 (-2.45)	0.58 (0.11)
Vorinostat	Salivary Gland Cancer	1	21.31 (2.99 - 151.62)	21.3 (19.31)	4.41 (2.74)	21.26 (4.12)
Vorinostat	Product Distribution Issue	1	2.3 (0.32 - 16.36)	2.3 (0.74)	1.2 (-0.46)	2.3 (0.45)
Vorinostat	Gastrointestinal Infection	1	1.3 (0.18 - 9.26)	1.3 (0.07)	0.38 (-1.28)	1.3 (0.25)
Vorinostat	Aplastic Anaemia	1	2.29 (0.32 - 16.28)	2.29 (0.73)	1.2 (-0.47)	2.29 (0.44)
Vorinostat	Migraine	1	0.12 (0.02 - 0.88)	0.12 (6.16)	-3 (-4.67)	0.12 (0.02)

Vorinostat	Drug-Induced Liver Injury	1	0.43 (0.06 - 3.08)	0.43 (0.74)	-1.2 (-2.87)	0.43 (0.08)
Vorinostat	Incorrect Dose Administered	1	0.06 (0.01 - 0.41)	0.06 (15.27)	-4.1 (-5.77)	0.06 (0.01)
Vorinostat	Panic Attack	1	0.32 (0.04 - 2.25)	0.32 (1.47)	-1.66 (-3.32)	0.32 (0.06)
Vorinostat	Rhinitis Atrophic	1	474.06 (63.61 - 3533.02)	473.97 (449.5)	8.82 (7.04)	451.45 (84.08)
Vorinostat	Gastrointestinal Motility Disorder	1	2.27 (0.32 - 16.11)	2.27 (0.71)	1.18 (-0.49)	2.27 (0.44)
Vorinostat	Rhinovirus Infection	1	3.68 (0.52 - 26.16)	3.68 (1.95)	1.88 (0.21)	3.68 (0.71)
Vorinostat	Incorrect Route Of Product Administration	1	0.2 (0.03 - 1.41)	0.2 (3.22)	-2.33 (-3.99)	0.2 (0.04)
Vorinostat	Squamous Cell Carcinoma Of Skin	1	1.97 (0.28 - 13.99)	1.97 (0.48)	0.98 (-0.69)	1.97 (0.38)
Vorinostat	Malignant Dysphagia	1	220.49 (30.36 - 1601.51)	220.45 (213.49)	7.75 (6.03)	215.46 (41)
Vorinostat	Cardiac Dysfunction	1	3.69 (0.52 - 26.2)	3.69 (1.96)	1.88 (0.22)	3.69 (0.71)
Vorinostat	Red Blood Cell Count Increased	1	4.24 (0.6 - 30.11)	4.24 (2.47)	2.08 (0.42)	4.24 (0.82)
Vorinostat	Nervousness	1	0.21 (0.03 - 1.49)	0.21 (2.97)	-2.25 (-3.92)	0.21 (0.04)
Vorinostat	Hepatitis A Antibody Positive	1	65.84 (9.21 - 470.7)	65.83 (63.4)	6.03 (4.35)	65.38 (12.61)
Vorinostat	Hepatitis B Virus Test Positive	1	26.93 (3.78 - 191.79)	26.93 (24.9)	4.75 (3.07)	26.86 (5.2)
Vorinostat	Hepatitis C Virus Test Positive	1	15.49 (2.18 - 110.18)	15.49 (13.53)	3.95 (2.28)	15.47 (3)
Vorinostat	Obstruction Gastric	1	9.42 (1.32 - 66.92)	9.41 (7.51)	3.23 (1.56)	9.41 (1.82)
Vorinostat	Cold Sweat	1	0.66 (0.09 - 4.65)	0.66 (0.18)	-0.61 (-2.28)	0.66 (0.13)
Vorinostat	Heart Rate Abnormal	1	2.13 (0.3 - 15.16)	2.13 (0.6)	1.09 (-0.57)	2.13 (0.41)
Vorinostat	Flushing	1	0.11 (0.02 - 0.76)	0.11 (7.42)	-3.22 (-4.88)	0.11 (0.02)
Vorinostat	Meningioma Malignant	1	215.48 (29.68 - 1564.31)	215.44 (208.7)	7.72 (6)	210.67 (40.11)
Vorinostat	Magnetic Resonance Imaging Head Abnormal	1	5.26 (0.74 - 37.34)	5.25 (3.44)	2.39 (0.73)	5.25 (1.02)
Vorinostat	Gastrointestinal Sounds Abnormal	1	2.39 (0.34 - 16.97)	2.39 (0.81)	1.26 (-0.41)	2.39 (0.46)

Vorinostat	Skin Mass	1	1.6 (0.23 - 11.38)	1.6 (0.23)	0.68 (-0.99)	1.6 (0.31)
Vorinostat	Escherichia Urinary Tract Infection	1	2.7 (0.38 - 19.18)	2.7 (1.07)	1.43 (-0.23)	2.7 (0.52)
Vorinostat	Respiratory Acidosis	1	3.05 (0.43 - 21.67)	3.05 (1.38)	1.61 (-0.06)	3.05 (0.59)
Vorinostat	Right Ventricular Failure	1	1.54 (0.22 - 10.93)	1.54 (0.19)	0.62 (-1.05)	1.54 (0.3)
Vorinostat	Pulmonary Artery Thrombosis	1	15.19 (2.14 - 108.06)	15.19 (13.24)	3.92 (2.25)	15.17 (2.94)
Vorinostat	Carpal Tunnel Syndrome	1	0.81 (0.11 - 5.73)	0.81 (0.05)	-0.31 (-1.98)	0.81 (0.16)
Vorinostat	Dermatomyositis	1	4.85 (0.68 - 34.43)	4.85 (3.05)	2.28 (0.61)	4.84 (0.94)
Vorinostat	Electrocardiogram St Segment Abnormal	1	16.04 (2.26 - 114.1)	16.04 (14.08)	4 (2.33)	16.01 (3.1)
Vorinostat	Eosinophil Count Increased	1	1.44 (0.2 - 10.23)	1.44 (0.13)	0.53 (-1.14)	1.44 (0.28)
Vorinostat	High Density Lipoprotein Decreased	1	3.56 (0.5 - 25.3)	3.56 (1.84)	1.83 (0.16)	3.56 (0.69)
Vorinostat	Monocyte Count Decreased	1	9.99 (1.41 - 71.01)	9.99 (8.08)	3.32 (1.65)	9.98 (1.93)
Vorinostat	Osteoarthritis	1	0.27 (0.04 - 1.92)	0.27 (1.96)	-1.88 (-3.55)	0.27 (0.05)
Vorinostat	Peripheral Coldness	1	0.83 (0.12 - 5.91)	0.83 (0.03)	-0.26 (-1.93)	0.83 (0.16)
Vorinostat	Pharyngeal Erythema	1	5 (0.7 - 35.54)	5 (3.2)	2.32 (0.65)	5 (0.97)
Vorinostat	Sclerodactylia	1	118.51 (16.49 - 851.86)	118.49 (115.06)	6.87 (5.17)	117.04 (22.47)
Vorinostat	Sensory Loss	1	1.41 (0.2 - 10)	1.41 (0.12)	0.49 (-1.17)	1.41 (0.27)
Vorinostat	Skin Tightness	1	2.35 (0.33 - 16.72)	2.35 (0.78)	1.24 (-0.43)	2.35 (0.46)
Vorinostat	Haemorrhage Urinary Tract	1	4.46 (0.63 - 31.66)	4.46 (2.68)	2.16 (0.49)	4.46 (0.86)
Vorinostat	Pyelocaliectasis	1	11.94 (1.68 - 84.89)	11.94 (10.01)	3.58 (1.91)	11.92 (2.31)
Vorinostat	Osteonecrosis	1	0.33 (0.05 - 2.32)	0.33 (1.39)	-1.61 (-3.28)	0.33 (0.06)
Vorinostat	Prothrombin Time Shortened	1	8.65 (1.22 - 61.48)	8.65 (6.76)	3.11 (1.44)	8.64 (1.67)
Vorinostat	Loss Of Personal Independence In Daily Activities	1	0.17 (0.02 - 1.18)	0.17 (4.17)	-2.58 (-4.25)	0.17 (0.03)
Vorinostat	Gastritis Erosive	1	2.47 (0.35 - 17.55)	2.47 (0.88)	1.3 (-0.36)	2.47 (0.48)

Vorinostat	Hiatus Hernia	1	0.86 (0.12 - 6.14)	0.86 (0.02)	-0.21 (-1.88)	0.86 (0.17)
Vorinostat	Carotid Artery Stenosis	1	3.34 (0.47 - 23.75)	3.34 (1.64)	1.74 (0.07)	3.34 (0.65)
Vorinostat	Conjunctival Disorder	1	30.39 (4.27 - 216.45)	30.38 (28.32)	4.92 (3.25)	30.29 (5.86)
Vorinostat	Lumbar Puncture Abnormal	1	110.25 (15.35 - 791.76)	110.23 (106.99)	6.77 (5.07)	108.97 (20.93)
Vorinostat	Anisocoria	1	7.76 (1.09 - 55.18)	7.76 (5.89)	2.96 (1.29)	7.76 (1.5)
Vorinostat	Retroperitoneal Haematoma	1	5.18 (0.73 - 36.79)	5.18 (3.37)	2.37 (0.7)	5.17 (1)
Vorinostat	Cystitis	1	0.34 (0.05 - 2.43)	0.34 (1.26)	-1.55 (-3.21)	0.34 (0.07)
Vorinostat	Depressed Mood	1	0.22 (0.03 - 1.56)	0.22 (2.77)	-2.18 (-3.85)	0.22 (0.04)
Vorinostat	Cheilitis	1	2.13 (0.3 - 15.11)	2.13 (0.6)	1.09 (-0.58)	2.13 (0.41)
Vorinostat	Lip Pain	1	3.97 (0.56 - 28.19)	3.97 (2.22)	1.99 (0.32)	3.97 (0.77)
Vorinostat	Oral Herpes	1	0.6 (0.08 - 4.23)	0.6 (0.27)	-0.75 (-2.41)	0.6 (0.12)
Vorinostat	Tenderness	1	1.06 (0.15 - 7.54)	1.06 (0)	0.09 (-1.58)	1.06 (0.21)
Vorinostat	Abnormal Behaviour	1	0.28 (0.04 - 1.99)	0.28 (1.84)	-1.83 (-3.5)	0.28 (0.05)
Vorinostat	Aggression	1	0.23 (0.03 - 1.63)	0.23 (2.58)	-2.12 (-3.79)	0.23 (0.04)
Vorinostat	Cystitis Bacterial	1	34.86 (4.89 - 248.4)	34.85 (32.76)	5.12 (3.44)	34.73 (6.71)
Vorinostat	Urinary Tract Infection Staphylococcal	1	26.41 (3.71 - 188.04)	26.4 (24.38)	4.72 (3.05)	26.33 (5.1)
Vorinostat	Laboratory Test Abnormal	1	0.39 (0.06 - 2.78)	0.39 (0.95)	-1.35 (-3.02)	0.39 (0.08)
Vorinostat	Skin Burning Sensation	1	0.16 (0.02 - 1.11)	0.16 (4.55)	-2.67 (-4.34)	0.16 (0.03)
Vorinostat	Product Prescribing Error	1	0.26 (0.04 - 1.82)	0.26 (2.15)	-1.96 (-3.63)	0.26 (0.05)
Vorinostat	Pulmonary Hypertension	1	0.54 (0.08 - 3.82)	0.54 (0.4)	-0.89 (-2.56)	0.54 (0.1)
Vorinostat	Blood Thyroid Stimulating Hormone Increased	1	1.22 (0.17 - 8.69)	1.22 (0.04)	0.29 (-1.38)	1.22 (0.24)
Vorinostat	Neurotoxicity	1	0.72 (0.1 - 5.12)	0.72 (0.11)	-0.47 (-2.14)	0.72 (0.14)
Vorinostat	Oral Fungal Infection	1	4.17 (0.59 - 29.6)	4.17 (2.41)	2.06 (0.39)	4.17 (0.81)
Vorinostat	Amnesia	1	0.17 (0.02 - 1.22)	0.17 (4)	-2.54 (-4.21)	0.17 (0.03)

Vorinostat	Metastases To Spleen	1	27.48 (3.86 - 195.69)	27.48 (25.44)	4.78 (3.1)	27.4 (5.3)
Vorinostat	Laryngeal Pain	1	2.55 (0.36 - 18.08)	2.55 (0.94)	1.35 (-0.32)	2.55 (0.49)
Vorinostat	Tumour Pain	1	8.34 (1.17 - 59.26)	8.34 (6.45)	3.06 (1.39)	8.33 (1.61)
Vorinostat	Myelofibrosis	1	3.86 (0.54 - 27.45)	3.86 (2.12)	1.95 (0.28)	3.86 (0.75)
Vorinostat	Biliary Obstruction	1	3.8 (0.54 - 27.02)	3.8 (2.07)	1.93 (0.26)	3.8 (0.74)
Vorinostat	Platelet Count Abnormal	1	1.94 (0.27 - 13.81)	1.94 (0.46)	0.96 (-0.71)	1.94 (0.38)
Vorinostat	Portal Vein Occlusion	1	83.17 (11.61 - 595.63)	83.15 (80.46)	6.37 (4.68)	82.44 (15.87)
Vorinostat	Feeling Abnormal	1	0.05 (0.01 - 0.33)	0.05 (19.86)	-4.44 (-6.11)	0.05 (0.01)
Vorinostat	Oral Disorder	1	1.45 (0.2 - 10.28)	1.45 (0.14)	0.53 (-1.13)	1.45 (0.28)
Vorinostat	Device Related Thrombosis	1	11.09 (1.56 - 78.83)	11.09 (9.17)	3.47 (1.8)	11.08 (2.15)
Vorinostat	Lymphocyte Count Abnormal	1	11.09 (1.56 - 78.83)	11.09 (9.17)	3.47 (1.8)	11.08 (2.15)
Vorinostat	Monocyte Count Abnormal	1	62.79 (8.79 - 448.74)	62.78 (60.39)	5.96 (4.28)	62.37 (12.03)
Vorinostat	Oral Infection	1	2.85 (0.4 - 20.22)	2.85 (1.2)	1.51 (-0.16)	2.85 (0.55)
Vorinostat	Atrioventricular Block First Degree	1	2.59 (0.36 - 18.37)	2.59 (0.97)	1.37 (-0.3)	2.59 (0.5)
Vorinostat	Haemolysis	1	1.5 (0.21 - 10.62)	1.5 (0.16)	0.58 (-1.09)	1.5 (0.29)
Vorinostat	Nervous System Disorder	1	0.35 (0.05 - 2.52)	0.35 (1.18)	-1.5 (-3.16)	0.35 (0.07)
Vorinostat	Osteosclerosis	1	7.01 (0.99 - 49.83)	7.01 (5.15)	2.81 (1.14)	7.01 (1.36)
Vorinostat	Spinal Disorder	1	1.19 (0.17 - 8.43)	1.19 (0.03)	0.25 (-1.42)	1.19 (0.23)
Vorinostat	Blood Sodium Abnormal	1	11.97 (1.68 - 85.11)	11.97 (10.04)	3.58 (1.91)	11.96 (2.32)
Vorinostat	Choking	1	0.61 (0.09 - 4.31)	0.61 (0.26)	-0.72 (-2.39)	0.61 (0.12)
Vorinostat	Hemiplegia	1	1.36 (0.19 - 9.68)	1.36 (0.1)	0.45 (-1.22)	1.36 (0.26)
Vorinostat	Infarction	1	1.52 (0.21 - 10.82)	1.52 (0.18)	0.61 (-1.06)	1.52 (0.3)
Vorinostat	Stenotrophomonas Infection	1	9.51 (1.34 - 67.59)	9.51 (7.61)	3.25 (1.58)	9.5 (1.84)
Vorinostat	Blood Triglycerides Increased	1	0.66 (0.09 - 4.68)	0.66 (0.18)	-0.6 (-2.27)	0.66 (0.13)
Vorinostat	Vascular Occlusion	1	5.58 (0.78 - 39.62)	5.58 (3.75)	2.48 (0.81)	5.57 (1.08)
Vorinostat	Hepatic Failure	1	0.39 (0.06 - 2.8)	0.39 (0.93)	-1.34 (-3.01)	0.39 (0.08)
Vorinostat	Organ Failure	1	2.91 (0.41 - 20.64)	2.91 (1.25)	1.54 (-0.13)	2.91 (0.56)

Vorinostat	Eye Disorder	1	0.36 (0.05 - 2.53)	0.36 (1.16)	-1.49 (-3.15)	0.36 (0.07)
Vorinostat	Post-Traumatic Epilepsy	1	145.86 (20.24 - 1051.4)	145.84 (141.66)	7.17 (5.46)	143.64 (27.51)
Vorinostat	Product Dispensing Error	1	0.31 (0.04 - 2.21)	0.31 (1.53)	-1.69 (-3.35)	0.31 (0.06)
Vorinostat	Culture Wound Positive	1	43.89 (6.15 - 313.09)	43.89 (41.72)	5.45 (3.77)	43.69 (8.44)
Vorinostat	Enterococcal Sepsis	1	13.6 (1.91 - 96.72)	13.6 (11.66)	3.76 (2.09)	13.58 (2.63)
Vorinostat	Epididymitis	1	9.94 (1.4 - 70.64)	9.94 (8.03)	3.31 (1.64)	9.93 (1.92)
Vorinostat	Lung Cancer Metastatic	1	3.81 (0.54 - 27.04)	3.81 (2.07)	1.93 (0.26)	3.8 (0.74)
Vorinostat	Metastatic Carcinoma Of The Bladder	1	30.88 (4.34 - 219.99)	30.88 (28.82)	4.94 (3.27)	30.78 (5.95)
Vorinostat	Electrocardiogram T Wave Abnormal	1	8.07 (1.14 - 57.34)	8.07 (6.19)	3.01 (1.34)	8.06 (1.56)
Vorinostat	Klebsiella Sepsis	1	10.49 (1.48 - 74.55)	10.49 (8.57)	3.39 (1.72)	10.48 (2.03)
Vorinostat	Epstein-Barr Virus Associated Lymphoproliferative Disorder	1	6.8 (0.96 - 48.33)	6.8 (4.94)	2.76 (1.1)	6.8 (1.32)
Vorinostat	Beta Haemolytic Streptococcal Infection	1	10.17 (1.43 - 72.31)	10.17 (8.26)	3.34 (1.68)	10.16 (1.97)
Vorinostat	Glycosylated Haemoglobin Increased	1	0.47 (0.07 - 3.33)	0.47 (0.6)	-1.09 (-2.76)	0.47 (0.09)
Vorinostat	Mucormycosis	1	3.84 (0.54 - 27.29)	3.84 (2.1)	1.94 (0.27)	3.84 (0.74)
Vorinostat	T-Cell Lymphoma Recurrent	1	193.49 (26.71 - 1401.53)	193.46 (187.63)	7.57 (5.85)	189.61 (36.17)
Vorinostat	Pharyngitis	1	0.9 (0.13 - 6.37)	0.9 (0.01)	-0.16 (-1.82)	0.9 (0.17)
Vorinostat	Nasal Congestion	1	0.2 (0.03 - 1.43)	0.2 (3.15)	-2.31 (-3.97)	0.2 (0.04)
Vorinostat	Blood Pressure Diastolic Decreased	1	1.5 (0.21 - 10.64)	1.5 (0.17)	0.58 (-1.08)	1.5 (0.29)
Vorinostat	Troponin I Increased	1	7.17 (1.01 - 50.92)	7.17 (5.3)	2.84 (1.17)	7.16 (1.39)
Vorinostat	Anorectal Discomfort	1	1.97 (0.28 - 14.01)	1.97 (0.48)	0.98 (-0.69)	1.97 (0.38)

Vorinostat	Eye Discharge	1	1.22 (0.17 - 8.65)	1.22 (0.04)	0.28 (-1.38)	1.22 (0.24)
Vorinostat	Eye Pain	1	0.22 (0.03 - 1.59)	0.22 (2.69)	-2.16 (-3.82)	0.22 (0.04)
Vorinostat	Oliguria	1	1.89 (0.27 - 13.45)	1.89 (0.42)	0.92 (-0.75)	1.89 (0.37)
Vorinostat	Mobility Decreased	1	0.16 (0.02 - 1.13)	0.16 (4.44)	-2.65 (-4.32)	0.16 (0.03)
Vorinostat	Hyperpyrexia	1	3.23 (0.45 - 22.94)	3.23 (1.54)	1.69 (0.02)	3.23 (0.63)
Vorinostat	Cyanosis	1	0.76 (0.11 - 5.39)	0.76 (0.08)	-0.4 (-2.06)	0.76 (0.15)
Vorinostat	Hypercapnia	1	4.02 (0.57 - 28.55)	4.02 (2.27)	2.01 (0.34)	4.02 (0.78)
Vorinostat	Non-Small Cell Lung Cancer Stage Iv	1	31.19 (4.38 - 222.17)	31.18 (29.12)	4.96 (3.28)	31.08 (6.01)
Vorinostat	Waist Circumference Increased	1	10.29 (1.45 - 73.17)	10.29 (8.38)	3.36 (1.69)	10.28 (1.99)
Vorinostat	Chest Injury	1	5.64 (0.79 - 40.07)	5.64 (3.81)	2.49 (0.83)	5.64 (1.09)
Vorinostat	Fluid Retention	1	0.22 (0.03 - 1.56)	0.22 (2.76)	-2.18 (-3.85)	0.22 (0.04)
Vorinostat	Crepitations	1	3.76 (0.53 - 26.71)	3.76 (2.03)	1.91 (0.24)	3.76 (0.73)
Vorinostat	Necrosis	1	2.13 (0.3 - 15.15)	2.13 (0.6)	1.09 (-0.57)	2.13 (0.41)
Vorinostat	Procedural Site Reaction	1	22.96 (3.23 - 163.39)	22.95 (20.95)	4.52 (2.84)	22.9 (4.43)
Vorinostat	Rectal Perforation	1	21.07 (2.96 - 149.93)	21.07 (19.07)	4.39 (2.72)	21.02 (4.07)
Vorinostat	Myelitis	1	8.08 (1.14 - 57.44)	8.08 (6.2)	3.01 (1.35)	8.08 (1.57)
Vorinostat	Anaemia Of Malignant Disease	1	40 (5.61 - 285.23)	40 (37.86)	5.32 (3.64)	39.83 (7.7)
Vorinostat	Aortic Aneurysm	1	1.44 (0.2 - 10.23)	1.44 (0.13)	0.53 (-1.14)	1.44 (0.28)
Vorinostat	Incontinence	1	1.09 (0.15 - 7.74)	1.09 (0.01)	0.12 (-1.54)	1.09 (0.21)
Vorinostat	Vertigo	1	0.19 (0.03 - 1.32)	0.19 (3.56)	-2.42 (-4.09)	0.19 (0.04)
Vorinostat	Opportunistic Infection	1	6.13 (0.86 - 43.57)	6.13 (4.29)	2.62 (0.95)	6.13 (1.19)
Vorinostat	Therapeutic Response Decreased	1	0.2 (0.03 - 1.42)	0.2 (3.21)	-2.32 (-3.99)	0.2 (0.04)
Vorinostat	Eye Swelling	1	0.32 (0.04 - 2.24)	0.32 (1.48)	-1.66 (-3.33)	0.32 (0.06)
Vorinostat	Ocular Hyperaemia	1	0.26 (0.04 - 1.87)	0.26 (2.06)	-1.92 (-3.59)	0.26 (0.05)
Vorinostat	Skin Hyperpigmentation	1	1.34 (0.19 - 9.5)	1.34 (0.09)	0.42 (-1.25)	1.34 (0.26)
Vorinostat	Cardiovascular Insufficiency	1	6.4 (0.9 - 45.49)	6.4 (4.55)	2.68 (1.01)	6.4 (1.24)

Vorinostat	Haematochezia	1	0.21 (0.03 - 1.51)	0.21 (2.91)	-2.23 (-3.9)	0.21 (0.04)
Vorinostat	Chromaturia	1	0.49 (0.07 - 3.49)	0.49 (0.52)	-1.02 (-2.69)	0.49 (0.1)
Vorinostat	Blood Creatine Phosphokinase Decreased	1	30.39 (4.27 - 216.45)	30.38 (28.32)	4.92 (3.25)	30.29 (5.86)
Vorinostat	Gastric Cancer	1	0.39 (0.05 - 2.77)	0.39 (0.95)	-1.36 (-3.02)	0.39 (0.08)
Vorinostat	Palpitations	1	0.1 (0.01 - 0.7)	0.1 (8.24)	-3.34 (-5.01)	0.1 (0.02)
Vorinostat	Peripheral Embolism	1	9.29 (1.31 - 66)	9.28 (7.39)	3.21 (1.54)	9.28 (1.8)
Vorinostat	Eye Infection	1	1.05 (0.15 - 7.49)	1.05 (0)	0.08 (-1.59)	1.05 (0.2)
Vorinostat	Hepatosplenomegaly	1	4.59 (0.65 - 32.62)	4.59 (2.81)	2.2 (0.53)	4.59 (0.89)
Vorinostat	Klebsiella Infection	1	2.45 (0.35 - 17.44)	2.45 (0.86)	1.3 (-0.37)	2.45 (0.48)
Vorinostat	Pseudomonal Bacteraemia	1	15.37 (2.16 - 109.28)	15.36 (13.41)	3.94 (2.27)	15.34 (2.97)
Vorinostat	Plasmacytoma	1	5.89 (0.83 - 41.87)	5.89 (4.06)	2.56 (0.89)	5.89 (1.14)
Vorinostat	Pulmonary Granuloma	1	10.46 (1.47 - 74.39)	10.46 (8.55)	3.39 (1.72)	10.45 (2.03)
Vorinostat	Sternal Fracture	1	9.2 (1.3 - 65.42)	9.2 (7.3)	3.2 (1.53)	9.2 (1.78)
Vorinostat	Subclavian Vein Thrombosis	1	9.89 (1.39 - 70.27)	9.88 (7.98)	3.3 (1.63)	9.88 (1.91)
Vorinostat	Bursitis	1	1.09 (0.15 - 7.75)	1.09 (0.01)	0.13 (-1.54)	1.09 (0.21)
Vorinostat	Candida Sepsis	1	13.2 (1.86 - 93.89)	13.2 (11.26)	3.72 (2.05)	13.19 (2.55)
Vorinostat	Chest X-Ray Abnormal	1	3.75 (0.53 - 26.67)	3.75 (2.02)	1.91 (0.24)	3.75 (0.73)
Vorinostat	Splenic Infarction	1	6.73 (0.95 - 47.85)	6.73 (4.88)	2.75 (1.08)	6.73 (1.3)
Vorinostat	Extremity Necrosis	1	6.54 (0.92 - 46.49)	6.54 (4.69)	2.71 (1.04)	6.54 (1.27)
Vorinostat	Perineal Ulceration	1	85.42 (11.92 - 611.87)	85.4 (82.67)	6.4 (4.72)	84.65 (16.3)
Vorinostat	Leukocyturia	1	14.41 (2.03 - 102.46)	14.41 (12.46)	3.85 (2.18)	14.39 (2.79)
Vorinostat	Blood Uric Acid Decreased	1	25.62 (3.6 - 182.43)	25.62 (23.6)	4.68 (3)	25.55 (4.95)
Vorinostat	Furuncle	1	1.38 (0.19 - 9.82)	1.38 (0.11)	0.47 (-1.2)	1.38 (0.27)
Vorinostat	Hypokinesia	1	0.76 (0.11 - 5.4)	0.76 (0.08)	-0.39 (-2.06)	0.76 (0.15)
Vorinostat	Wound Infection Staphylococcal	1	6.19 (0.87 - 44)	6.19 (4.35)	2.63 (0.96)	6.19 (1.2)
Vorinostat	Abdominal Rigidity	1	4.35 (0.61 - 30.88)	4.35 (2.58)	2.12 (0.45)	4.34 (0.84)

Vorinostat	General Symptom	1	1.43 (0.2 - 10.17)	1.43 (0.13)	0.52 (-1.15)	1.43 (0.28)
Vorinostat	Odynophagia	1	2.05 (0.29 - 14.54)	2.05 (0.54)	1.03 (-0.63)	2.05 (0.4)
Vorinostat	Pleural Rub	1	316.04 (43.09 - 2317.96)	315.98 (303.85)	8.26 (6.51)	305.82 (57.73)
Vorinostat	Retching	1	0.56 (0.08 - 3.95)	0.56 (0.35)	-0.85 (-2.51)	0.56 (0.11)
Vorinostat	Alcohol Use	1	3.35 (0.47 - 23.82)	3.35 (1.65)	1.75 (0.08)	3.35 (0.65)
Vorinostat	Metastases To Peritoneum	1	4.85 (0.68 - 34.47)	4.85 (3.06)	2.28 (0.61)	4.85 (0.94)
Vorinostat	Continuous Haemodiafiltration	1	10.17 (1.43 - 72.31)	10.17 (8.26)	3.34 (1.68)	10.16 (1.97)
Vorinostat	Bacillus Infection	1	21.55 (3.03 - 153.34)	21.54 (19.55)	4.43 (2.75)	21.5 (4.16)
Vorinostat	Bone Marrow Transplant	1	6.5 (0.92 - 46.2)	6.5 (4.65)	2.7 (1.03)	6.5 (1.26)
Vorinostat	Bk Virus Infection	1	3.99 (0.56 - 28.37)	3.99 (2.24)	2 (0.33)	3.99 (0.77)
Vorinostat	Cytomegalovirus Viraemia	1	3 (0.42 - 21.31)	3 (1.33)	1.58 (-0.08)	3 (0.58)
Vorinostat	Urinary Tract Infection Viral	1	108.98 (15.18 - 782.56)	108.96 (105.75)	6.75 (5.06)	107.73 (20.7)
Vorinostat	Arteriosclerosis	1	1.18 (0.17 - 8.38)	1.18 (0.03)	0.24 (-1.43)	1.18 (0.23)
Vorinostat	Blood Magnesium Increased	1	13.62 (1.92 - 96.86)	13.62 (11.68)	3.77 (2.1)	13.6 (2.63)
Vorinostat	Diverticulum Intestinal	1	2.86 (0.4 - 20.28)	2.86 (1.21)	1.51 (-0.15)	2.85 (0.55)
Vorinostat	Extradural Haematoma	1	8.36 (1.18 - 59.42)	8.36 (6.47)	3.06 (1.39)	8.35 (1.62)
Vorinostat	Normal Pressure Hydrocephalus	1	28.56 (4.01 - 203.38)	28.55 (26.51)	4.83 (3.16)	28.47 (5.51)
Vorinostat	Supraventricular Extrasystoles	1	3.48 (0.49 - 24.75)	3.48 (1.77)	1.8 (0.13)	3.48 (0.68)
Vorinostat	Anaemia Postoperative	1	7.76 (1.09 - 55.13)	7.76 (5.88)	2.95 (1.29)	7.75 (1.5)
Vorinostat	Mesenteric Vein Thrombosis	1	11.22 (1.58 - 79.76)	11.22 (9.3)	3.49 (1.82)	11.21 (2.17)
Vorinostat	Ecchymosis	1	1.67 (0.24 - 11.87)	1.67 (0.27)	0.74 (-0.93)	1.67 (0.32)
Vorinostat	Exercise Tolerance Decreased	1	2.01 (0.28 - 14.26)	2.01 (0.51)	1.01 (-0.66)	2.01 (0.39)
Vorinostat	Dyskinesia	1	0.28 (0.04 - 1.99)	0.28 (1.85)	-1.83 (-3.5)	0.28 (0.05)
Vorinostat	Peripheral Sensorimotor Neuropathy	1	10.94 (1.54 - 77.74)	10.93 (9.01)	3.45 (1.78)	10.92 (2.12)

Vorinostat	Simple Partial Seizures	1	15.72 (2.21 - 111.83)	15.72 (13.76)	3.97 (2.3)	15.7 (3.04)
Vorinostat	Post Procedural Haemorrhage	1	0.96 (0.14 - 6.85)	0.96 (0)	-0.05 (-1.72)	0.96 (0.19)
Vorinostat	Adenocarcinoma	1	4.98 (0.7 - 35.41)	4.98 (3.18)	2.32 (0.65)	4.98 (0.97)
Vorinostat	Pleurodesis	1	111.54 (15.53 - 801.18)	111.52 (108.26)	6.78 (5.09)	110.24 (21.18)
Vorinostat	Ureteric Obstruction	1	8.24 (1.16 - 58.59)	8.24 (6.36)	3.04 (1.37)	8.24 (1.6)
Vorinostat	Blood Ph Decreased	1	7.87 (1.11 - 55.96)	7.87 (6)	2.98 (1.31)	7.87 (1.52)
Vorinostat	Bundle Branch Block Left	1	2.74 (0.39 - 19.46)	2.74 (1.1)	1.45 (-0.21)	2.74 (0.53)
Vorinostat	Multi-Organ Disorder	1	6.16 (0.87 - 43.8)	6.16 (4.32)	2.62 (0.95)	6.16 (1.19)
Vorinostat	Alpha Haemolytic Streptococcal Infection	1	14.21 (2 - 101.08)	14.21 (12.26)	3.83 (2.16)	14.19 (2.75)
Vorinostat	Vascular Access Device Culture Positive	1	133.54 (18.55 - 961.33)	133.51 (129.69)	7.04 (5.34)	131.67 (25.25)
Vorinostat	Splenomegaly	1	0.98 (0.14 - 6.96)	0.98 (0)	-0.03 (-1.7)	0.98 (0.19)
Vorinostat	Viral Upper Respiratory Tract Infection	1	2.19 (0.31 - 15.55)	2.19 (0.65)	1.13 (-0.54)	2.19 (0.42)
Vorinostat	Apoptosis	1	36.89 (5.18 - 262.95)	36.88 (34.78)	5.2 (3.52)	36.75 (7.1)
Vorinostat	Gastrointestinal Inflammation	1	1.51 (0.21 - 10.69)	1.51 (0.17)	0.59 (-1.08)	1.51 (0.29)
Vorinostat	Signet-Ring Cell Carcinoma	1	137.41 (19.08 - 989.59)	137.38 (133.46)	7.08 (5.38)	135.43 (25.96)
Vorinostat	Hyperviscosity Syndrome	1	42.71 (5.99 - 304.59)	42.7 (40.54)	5.41 (3.73)	42.51 (8.22)
Vorinostat	Decreased Activity	1	1.11 (0.16 - 7.86)	1.11 (0.01)	0.15 (-1.52)	1.11 (0.21)
Vorinostat	Haematology Test Abnormal	1	35.91 (5.04 - 255.95)	35.91 (33.81)	5.16 (3.49)	35.77 (6.92)
Vorinostat	Device Related Sepsis	1	4.99 (0.7 - 35.45)	4.99 (3.19)	2.32 (0.65)	4.99 (0.97)
Vorinostat	Animal Scratch	1	11.78 (1.66 - 83.73)	11.78 (9.85)	3.56 (1.89)	11.76 (2.28)
Vorinostat	Performance Status Decreased	1	2.51 (0.35 - 17.83)	2.51 (0.91)	1.33 (-0.34)	2.51 (0.49)
Vorinostat	Pharyngeal Disorder	1	3.59 (0.5 - 25.47)	3.59 (1.86)	1.84 (0.17)	3.58 (0.69)

Vorinostat	Red Blood Cell Anisocytes Present	1	948.11 (121.34 - 7408)	947.94 (859.94)	9.75 (7.89)	861.85 (154.3)
Vorinostat	Red Blood Cell Macrocytes Present	1	395.05 (53.43 - 2920.77)	394.97 (377.26)	8.57 (6.81)	379.21 (71.1)
Vorinostat	Red Cell Distribution Width Increased	1	2.79 (0.39 - 19.81)	2.79 (1.15)	1.48 (-0.19)	2.79 (0.54)
Vorinostat	Blood Calcium Increased	1	1.34 (0.19 - 9.49)	1.34 (0.08)	0.42 (-1.25)	1.34 (0.26)
Vorinostat	Squamous Cell Carcinoma	1	1.22 (0.17 - 8.69)	1.22 (0.04)	0.29 (-1.38)	1.22 (0.24)
Vorinostat	Diplopia	1	0.46 (0.06 - 3.24)	0.46 (0.65)	-1.13 (-2.8)	0.46 (0.09)
Vorinostat	Vith Nerve Paralysis	1	10.34 (1.45 - 73.49)	10.34 (8.43)	3.37 (1.7)	10.33 (2)
Vorinostat	Abdominal Strangulated Hernia	1	64.94 (9.08 - 464.21)	64.93 (62.51)	6.01 (4.33)	64.49 (12.44)
Vorinostat	Hallucinations, Mixed	1	2.89 (0.41 - 20.5)	2.89 (1.23)	1.53 (-0.14)	2.89 (0.56)
Vorinostat	Candida Pneumonia	1	37.04 (5.2 - 263.98)	37.03 (34.92)	5.21 (3.53)	36.89 (7.13)
Vorinostat	Cardiac Valve Vegetation	1	23.7 (3.33 - 168.72)	23.7 (21.69)	4.56 (2.89)	23.64 (4.58)
Vorinostat	Oesophageal Ulcer	1	3.02 (0.42 - 21.43)	3.02 (1.35)	1.59 (-0.07)	3.02 (0.58)
Vorinostat	Respiratory Moniliasis	1	50.16 (7.03 - 358.05)	50.16 (47.92)	5.64 (3.96)	49.9 (9.64)
Vorinostat	Bicytopenia	1	6.68 (0.94 - 47.47)	6.68 (4.83)	2.74 (1.07)	6.68 (1.29)
Vorinostat	Limb Injury	1	0.43 (0.06 - 3.03)	0.43 (0.77)	-1.23 (-2.9)	0.43 (0.08)
Vorinostat	Pyoderma Gangrenosum	1	3.62 (0.51 - 25.69)	3.62 (1.89)	1.85 (0.19)	3.62 (0.7)
Vorinostat	Anaplastic Large Cell Lymphoma T- And Null-Cell Types Refractory	1	1354.45 (166.61 - 11011)	1354.2 (1183.17)	10.21 (8.29)	1185.05 (205.24)
Vorinostat	Blood Bicarbonate Decreased	1	7.04 (0.99 - 50.05)	7.04 (5.18)	2.82 (1.15)	7.04 (1.36)
Vorinostat	Cholestatic Liver Injury	1	6.41 (0.9 - 45.55)	6.41 (4.56)	2.68 (1.01)	6.41 (1.24)
Vorinostat	Ischaemic Stroke	1	0.63 (0.09 - 4.47)	0.63 (0.22)	-0.67 (-2.33)	0.63 (0.12)
Vorinostat	Device Occlusion	1	0.92 (0.13 - 6.54)	0.92 (0.01)	-0.12 (-1.79)	0.92 (0.18)
Vorinostat	Hypovolaemia	1	2.05 (0.29 - 14.58)	2.05 (0.54)	1.04 (-0.63)	2.05 (0.4)
Vorinostat	Jugular Vein Thrombosis	1	6.49 (0.91 - 46.14)	6.49 (4.64)	2.7 (1.03)	6.49 (1.26)
Vorinostat	Aortic Dissection	1	2.59 (0.37 - 18.43)	2.59 (0.98)	1.38 (-0.29)	2.59 (0.5)

Vorinostat	Hepatic Artery Occlusion	1	145.86 (20.24 - 1051.4)	145.84 (141.66)	7.17 (5.46)	143.64 (27.51)
Vorinostat	Po2 Decreased	1	11.6 (1.63 - 82.5)	11.6 (9.68)	3.53 (1.87)	11.59 (2.25)
Vorinostat	Acute Hepatic Failure	1	0.86 (0.12 - 6.12)	0.86 (0.02)	-0.21 (-1.88)	0.86 (0.17)
Vorinostat	Drug Hypersensitivity	1	0.06 (0.01 - 0.4)	0.06 (15.88)	-4.15 (-5.82)	0.06 (0.01)
Vorinostat	Post Procedural Complication	1	0.67 (0.09 - 4.74)	0.67 (0.17)	-0.58 (-2.25)	0.67 (0.13)
Vorinostat	Purulence	1	5.67 (0.8 - 40.29)	5.67 (3.84)	2.5 (0.83)	5.67 (1.1)
Vorinostat	Renal Tubular Necrosis	1	1.22 (0.17 - 8.67)	1.22 (0.04)	0.29 (-1.38)	1.22 (0.24)
Vorinostat	Oesophageal Disorder	1	3 (0.42 - 21.32)	3 (1.33)	1.59 (-0.08)	3 (0.58)
Vorinostat	Organising Pneumonia	1	2.36 (0.33 - 16.75)	2.36 (0.78)	1.24 (-0.43)	2.36 (0.46)
Vorinostat	Diarrhoea Haemorrhagic	1	1.23 (0.17 - 8.72)	1.23 (0.04)	0.3 (-1.37)	1.23 (0.24)
Vorinostat	Aortic Arteriosclerosis	1	3.44 (0.48 - 24.43)	3.44 (1.73)	1.78 (0.11)	3.44 (0.67)
Vorinostat	Coronary Artery Stenosis	1	2.37 (0.33 - 16.8)	2.37 (0.79)	1.24 (-0.43)	2.36 (0.46)
Vorinostat	Facial Pain	1	1.29 (0.18 - 9.18)	1.29 (0.07)	0.37 (-1.3)	1.29 (0.25)
Vorinostat	Histoplasmosis	1	6.63 (0.93 - 47.14)	6.63 (4.78)	2.73 (1.06)	6.63 (1.29)
Vorinostat	Ischaemic Cardiomyopathy	1	3.44 (0.48 - 24.41)	3.44 (1.73)	1.78 (0.11)	3.43 (0.67)
Vorinostat	Parotid Gland Enlargement	1	18.2 (2.56 - 129.46)	18.19 (16.22)	4.18 (2.51)	18.16 (3.52)
Vorinostat	Salivary Gland Pain	1	52.38 (7.34 - 373.96)	52.37 (50.11)	5.7 (4.02)	52.09 (10.06)
Vorinostat	Serratia Infection	1	16.69 (2.35 - 118.73)	16.69 (14.72)	4.06 (2.39)	16.66 (3.23)
Vorinostat	Splenic Haemorrhage	1	22.68 (3.19 - 161.43)	22.68 (20.67)	4.5 (2.83)	22.63 (4.38)
Vorinostat	Streptococcal Infection	1	2.23 (0.31 - 15.84)	2.23 (0.68)	1.16 (-0.51)	2.23 (0.43)
Vorinostat	Dysstasia	1	0.38 (0.05 - 2.71)	0.38 (1)	-1.39 (-3.06)	0.38 (0.07)
Vorinostat	Delusion	1	0.77 (0.11 - 5.45)	0.77 (0.07)	-0.38 (-2.05)	0.77 (0.15)
Vorinostat	Psychotic Disorder	1	0.4 (0.06 - 2.81)	0.4 (0.92)	-1.33 (-3)	0.4 (0.08)
Vorinostat	Hip Fracture	1	0.36 (0.05 - 2.56)	0.36 (1.14)	-1.47 (-3.14)	0.36 (0.07)
Vorinostat	Micrococcus Test Positive	1	395.05 (53.43 - 2920.77)	394.97 (377.26)	8.57 (6.81)	379.21 (71.1)

Vorinostat	Staphylococcus Test Positive	1	4.74 (0.67 - 33.66)	4.74 (2.95)	2.24 (0.58)	4.74 (0.92)
Vorinostat	Brain Death	1	3.94 (0.55 - 27.99)	3.94 (2.19)	1.98 (0.31)	3.94 (0.76)
Vorinostat	Pupil Fixed	1	7.27 (1.02 - 51.66)	7.27 (5.4)	2.86 (1.19)	7.26 (1.41)
Vorinostat	Acquired Diaphragmatic Eventration	1	33.27 (4.67 - 237.03)	33.26 (31.18)	5.05 (3.38)	33.15 (6.41)
Vorinostat	Vascular Injury	1	8.03 (1.13 - 57.05)	8.03 (6.15)	3 (1.34)	8.02 (1.55)
Vorinostat	Polydipsia	1	3 (0.42 - 21.32)	3 (1.33)	1.59 (-0.08)	3 (0.58)
Vorinostat	Paraesthesia Oral	1	0.82 (0.11 - 5.79)	0.82 (0.04)	-0.29 (-1.96)	0.82 (0.16)
Vorinostat	Liver Injury	1	0.57 (0.08 - 4.02)	0.57 (0.33)	-0.82 (-2.49)	0.57 (0.11)
Vorinostat	Neuroblastoma Recurrent	1	77.08 (10.77 - 551.7)	77.07 (74.48)	6.26 (4.57)	76.45 (14.73)
Vorinostat	Disease Recurrence	1	0.26 (0.04 - 1.87)	0.26 (2.07)	-1.93 (-3.59)	0.26 (0.05)
Vorinostat	Feeling Cold	1	0.41 (0.06 - 2.9)	0.41 (0.86)	-1.29 (-2.96)	0.41 (0.08)
Vorinostat	Pneumococcal Infection	1	21.85 (3.07 - 155.47)	21.84 (19.84)	4.45 (2.77)	21.79 (4.22)
Vorinostat	Skin Bacterial Infection	1	15.54 (2.19 - 110.54)	15.54 (13.58)	3.96 (2.29)	15.52 (3.01)
Vorinostat	Gram Stain Positive	1	65.84 (9.21 - 470.7)	65.83 (63.4)	6.03 (4.35)	65.38 (12.61)
Vorinostat	Device Infusion Issue	1	5.3 (0.75 - 37.65)	5.3 (3.49)	2.4 (0.74)	5.3 (1.03)
Vorinostat	Surgical Procedure Repeated	1	20.79 (2.92 - 147.95)	20.79 (18.8)	4.37 (2.7)	20.74 (4.02)
Vorinostat	Hyperphagia	1	3.12 (0.44 - 22.15)	3.12 (1.44)	1.64 (-0.03)	3.12 (0.6)
Vorinostat	Sensation Of Foreign Body	1	1.44 (0.2 - 10.21)	1.44 (0.13)	0.52 (-1.14)	1.44 (0.28)
Vorinostat	Infusion Related Reaction	1	0.19 (0.03 - 1.33)	0.19 (3.54)	-2.42 (-4.08)	0.19 (0.04)
Vorinostat	Medication Error	1	0.24 (0.03 - 1.68)	0.24 (2.46)	-2.08 (-3.75)	0.24 (0.05)
Vorinostat	Gait Inability	1	0.2 (0.03 - 1.42)	0.2 (3.21)	-2.32 (-3.99)	0.2 (0.04)
Vorinostat	Serratia Test Positive	1	110.25 (15.35 - 791.76)	110.23 (106.99)	6.77 (5.07)	108.97 (20.93)
Vorinostat	Lactobacillus Test Positive	1	526.73 (70.3 - 3946.47)	526.63 (497.02)	8.96 (7.18)	498.97 (92.52)
Vorinostat	Bacillus Test Positive	1	114.23 (15.9 - 820.71)	114.21 (110.88)	6.82 (5.12)	112.86 (21.67)

Vorinostat	Streptococcus Test Positive	1	9.86 (1.39 - 70.05)	9.85 (7.95)	3.3 (1.63)	9.84 (1.91)
Vorinostat	Breast Cancer	1	0.11 (0.02 - 0.78)	0.11 (7.19)	-3.18 (-4.85)	0.11 (0.02)
Vorinostat	Myositis	1	1.45 (0.2 - 10.32)	1.45 (0.14)	0.54 (-1.13)	1.45 (0.28)
Vorinostat	Delusional Disorder, Unspecified Type	1	17.33 (2.44 - 123.29)	17.33 (15.36)	4.11 (2.44)	17.3 (3.35)
Vorinostat	Cerebral Ventricle Dilatation	1	10.11 (1.42 - 71.85)	10.11 (8.2)	3.34 (1.67)	10.1 (1.96)
Vorinostat	Bronchiectasis	1	1.83 (0.26 - 12.99)	1.83 (0.38)	0.87 (-0.8)	1.83 (0.35)
Vorinostat	Bronchial Wall Thickening	1	15.1 (2.12 - 107.37)	15.09 (13.14)	3.91 (2.24)	15.07 (2.92)
Vorinostat	Computerised Tomogram Thorax Abnormal	1	14.74 (2.07 - 104.86)	14.74 (12.79)	3.88 (2.21)	14.72 (2.85)
Vorinostat	Influenza Virus Test Positive	1	61.97 (8.67 - 442.84)	61.96 (59.58)	5.94 (4.26)	61.56 (11.88)
Vorinostat	Blood Magnesium Decreased	1	1.36 (0.19 - 9.68)	1.36 (0.1)	0.45 (-1.22)	1.36 (0.26)
Vorinostat	Salivary Gland Mass	1	67.24 (9.4 - 480.78)	67.23 (64.79)	6.06 (4.38)	66.76 (12.87)
Vorinostat	Vascular Encephalopathy	1	40.17 (5.63 - 286.45)	40.17 (38.03)	5.32 (3.65)	40 (7.73)
Vorinostat	Superinfection	1	8.19 (1.15 - 58.23)	8.19 (6.31)	3.03 (1.36)	8.19 (1.59)
Vorinostat	Infusion Site Erythema	1	1.58 (0.22 - 11.23)	1.58 (0.21)	0.66 (-1.01)	1.58 (0.31)
Vorinostat	Infusion Site Pain	1	0.97 (0.14 - 6.86)	0.97 (0)	-0.05 (-1.72)	0.97 (0.19)
Vorinostat	Cerebral Disorder	1	1.6 (0.23 - 11.38)	1.6 (0.23)	0.68 (-0.99)	1.6 (0.31)
Vorinostat	Occult Blood Positive	1	5.21 (0.73 - 37.05)	5.21 (3.4)	2.38 (0.71)	5.21 (1.01)
Vorinostat	Clostridium Test Positive	1	5.52 (0.78 - 39.23)	5.52 (3.7)	2.46 (0.8)	5.52 (1.07)
Vorinostat	Blood Albumin Increased	1	14.15 (1.99 - 100.63)	14.15 (12.2)	3.82 (2.15)	14.13 (2.74)
Vorinostat	Lung Neoplasm Malignant	1	0.26 (0.04 - 1.84)	0.26 (2.12)	-1.95 (-3.61)	0.26 (0.05)
Vorinostat	Upper Respiratory Tract Inflammation	1	9.03 (1.27 - 64.17)	9.03 (7.13)	3.17 (1.5)	9.02 (1.75)
Vorinostat	Venous Aneurysm	1	225.74 (31.06 - 1640.52)	225.7 (218.5)	7.78 (6.06)	220.47 (41.94)
Vorinostat	Pulmonary Thrombosis	1	1.06 (0.15 - 7.56)	1.06 (0)	0.09 (-1.58)	1.06 (0.21)

Vorinostat	Medical Device Discomfort	1	3.57 (0.5 - 25.36)	3.57 (1.85)	1.84 (0.17)	3.57 (0.69)
Vorinostat	Pulmonary Congestion	1	0.92 (0.13 - 6.52)	0.92 (0.01)	-0.12 (-1.79)	0.92 (0.18)
Vorinostat	Sneezing	1	0.53 (0.07 - 3.78)	0.53 (0.41)	-0.91 (-2.58)	0.53 (0.1)
Vorinostat	Accessory Spleen	1	117.05 (16.29 - 841.22)	117.03 (113.64)	6.85 (5.16)	115.61 (22.2)
Vorinostat	Gallbladder Disorder	1	0.62 (0.09 - 4.39)	0.62 (0.24)	-0.7 (-2.36)	0.62 (0.12)
Vorinostat	Lymphadenitis	1	4.93 (0.69 - 35.04)	4.93 (3.13)	2.3 (0.63)	4.93 (0.96)
Vorinostat	Dizziness Exertional	1	17.21 (2.42 - 122.4)	17.2 (15.23)	4.1 (2.43)	17.17 (3.33)
Vorinostat	Hypotonia	1	1.14 (0.16 - 8.13)	1.14 (0.02)	0.19 (-1.47)	1.14 (0.22)
Vorinostat	Myeloid Leukaemia	1	22.74 (3.19 - 161.82)	22.73 (20.73)	4.5 (2.83)	22.68 (4.39)
Vorinostat	Subdural Haemorrhage	1	4.02 (0.57 - 28.56)	4.02 (2.27)	2.01 (0.34)	4.02 (0.78)
Vorinostat	Bilirubin Conjugated Increased	1	5.34 (0.75 - 37.95)	5.34 (3.53)	2.42 (0.75)	5.34 (1.03)
Vorinostat	Metastases To Pleura	1	12.19 (1.71 - 86.64)	12.18 (10.25)	3.61 (1.94)	12.17 (2.36)
Vorinostat	Engraft Failure	1	37.77 (5.3 - 269.26)	37.77 (35.65)	5.23 (3.56)	37.62 (7.27)
Vorinostat	Blood Fibrinogen Increased	1	16.07 (2.26 - 114.29)	16.07 (14.11)	4 (2.33)	16.04 (3.11)
Vorinostat	Cerebral Thrombosis	1	4.03 (0.57 - 28.65)	4.03 (2.28)	2.01 (0.34)	4.03 (0.78)
Vorinostat	Amylase Abnormal	1	75.85 (10.6 - 542.81)	75.83 (73.26)	6.23 (4.55)	75.24 (14.5)
Vorinostat	Thrombosis In Device	1	3.06 (0.43 - 21.74)	3.06 (1.39)	1.61 (-0.05)	3.06 (0.59)
Vorinostat	Ear Haemorrhage	1	5.64 (0.79 - 40.09)	5.64 (3.82)	2.5 (0.83)	5.64 (1.09)
Vorinostat	Activated Partial Thromboplastin Time Prolonged	1	2.41 (0.34 - 17.14)	2.41 (0.83)	1.27 (-0.4)	2.41 (0.47)
Vorinostat	Lymphocyte Morphology Abnormal	1	19.67 (2.76 - 139.96)	19.67 (17.68)	4.29 (2.62)	19.63 (3.8)
Vorinostat	Respiratory Disorder	1	0.39 (0.05 - 2.76)	0.39 (0.96)	-1.36 (-3.03)	0.39 (0.08)
Vorinostat	Gamma-Glutamyltransferase Increased	1	0.53 (0.08 - 3.78)	0.53 (0.41)	-0.91 (-2.57)	0.53 (0.1)
Vorinostat	Varices Oesophageal	1	3.9 (0.55 - 27.74)	3.9 (2.16)	1.96 (0.3)	3.9 (0.76)

Vorinostat	Haemorrhage Subcutaneous	1	3.09 (0.44 - 21.98)	3.09 (1.42)	1.63 (-0.04)	3.09 (0.6)
Vorinostat	Muscle Haemorrhage	1	2.89 (0.41 - 20.54)	2.89 (1.24)	1.53 (-0.14)	2.89 (0.56)
Vorinostat	Functional Gastrointestinal Disorder	1	2.72 (0.38 - 19.33)	2.72 (1.09)	1.44 (-0.22)	2.72 (0.53)
Vorinostat	Ileus	1	0.99 (0.14 - 7.01)	0.99 (0)	-0.02 (-1.69)	0.99 (0.19)
Vorinostat	Depression	1	0.05 (0.01 - 0.34)	0.05 (18.66)	-4.36 (-6.03)	0.05 (0.01)
Vorinostat	Suicidal Ideation	1	0.13 (0.02 - 0.9)	0.13 (6.05)	-2.98 (-4.65)	0.13 (0.02)
Vorinostat	Blood Lactic Acid Increased	1	2.37 (0.33 - 16.8)	2.37 (0.79)	1.24 (-0.43)	2.36 (0.46)
Vorinostat	Muscle Spasticity	1	0.85 (0.12 - 6.03)	0.85 (0.03)	-0.24 (-1.9)	0.85 (0.16)
Vorinostat	Altered State Of Consciousness	1	0.54 (0.08 - 3.87)	0.54 (0.38)	-0.88 (-2.54)	0.54 (0.11)
Vorinostat	Mucosal Infection	1	44.72 (6.27 - 319.03)	44.71 (42.54)	5.48 (3.8)	44.51 (8.6)
Vorinostat	Ammonia Increased	1	2.23 (0.31 - 15.87)	2.23 (0.68)	1.16 (-0.51)	2.23 (0.43)
Vorinostat	Blood Test Abnormal	1	0.83 (0.12 - 5.91)	0.83 (0.03)	-0.27 (-1.93)	0.83 (0.16)
Vorinostat	Eosinophilic Pneumonia Acute	1	16.9 (2.38 - 120.21)	16.9 (14.93)	4.08 (2.41)	16.87 (3.27)
Vorinostat	Dermatitis Acneiform	1	1.99 (0.28 - 14.14)	1.99 (0.49)	0.99 (-0.67)	1.99 (0.39)
Vorinostat	Product Residue Present	1	0.86 (0.12 - 6.11)	0.86 (0.02)	-0.22 (-1.88)	0.86 (0.17)
Vorinostat	Personality Change	1	1.19 (0.17 - 8.42)	1.19 (0.03)	0.25 (-1.42)	1.19 (0.23)
Vorinostat	Anaplastic Large Cell Lymphoma T- And Null-Cell Types	1	53.57 (7.5 - 382.46)	53.56 (51.28)	5.73 (4.05)	53.26 (10.28)
Vorinostat	Cystitis Noninfective	1	8.29 (1.17 - 58.95)	8.29 (6.41)	3.05 (1.38)	8.29 (1.61)
Vorinostat	Electrocardiogram Pr Prolongation	1	14.47 (2.04 - 102.93)	14.47 (12.52)	3.85 (2.18)	14.45 (2.8)
Vorinostat	Visual Impairment	1	0.09 (0.01 - 0.65)	0.09 (9.06)	-3.45 (-5.12)	0.09 (0.02)
Vorinostat	Female Genital Tract Fistula	1	6.88 (0.97 - 48.85)	6.87 (5.02)	2.78 (1.11)	6.87 (1.33)
Vorinostat	Abdominal Abscess	1	2.68 (0.38 - 19.05)	2.68 (1.05)	1.42 (-0.24)	2.68 (0.52)
Vorinostat	Anastomotic Complication	1	35.51 (4.98 - 253.07)	35.5 (33.41)	5.14 (3.47)	35.37 (6.84)
Vorinostat	Anastomotic Leak	1	16.87 (2.37 - 120)	16.87 (14.9)	4.07 (2.4)	16.84 (3.26)
Vorinostat	Fistula	1	1.01 (0.14 - 7.17)	1.01 (0)	0.01 (-1.65)	1.01 (0.2)

Vorinostat	Urinary Tract Pain	1	23.58 (3.31 - 167.88)	23.58 (21.57)	4.56 (2.88)	23.52 (4.55)
Vorinostat	B-Cell Small Lymphocytic Lymphoma	1	56.44 (7.9 - 403.07)	56.42 (54.12)	5.81 (4.13)	56.1 (10.83)
Vorinostat	Second Primary Malignancy	1	1.28 (0.18 - 9.11)	1.28 (0.06)	0.36 (-1.31)	1.28 (0.25)
Vorinostat	Glucose Tolerance Impaired	1	2 (0.28 - 14.18)	2 (0.5)	1 (-0.67)	2 (0.39)
Vorinostat	Rash Pruritic	1	0.22 (0.03 - 1.58)	0.22 (2.71)	-2.16 (-3.83)	0.22 (0.04)
Vorinostat	Pulmonary Valve Incompetence	1	9.58 (1.35 - 68.07)	9.58 (7.67)	3.26 (1.59)	9.57 (1.85)
Vorinostat	Tricuspid Valve Incompetence	1	1.61 (0.23 - 11.42)	1.61 (0.23)	0.69 (-0.98)	1.61 (0.31)
Vorinostat	Chronic Myeloid Leukaemia	1	2.51 (0.35 - 17.85)	2.51 (0.91)	1.33 (-0.34)	2.51 (0.49)
Vorinostat	Jejunal Perforation	1	46.25 (6.48 - 329.97)	46.24 (44.05)	5.52 (3.85)	46.02 (8.89)
Vorinostat	Blood Creatinine Abnormal	1	3.5 (0.49 - 24.86)	3.5 (1.78)	1.81 (0.14)	3.5 (0.68)
Vorinostat	Pneumocystis Jirovecii Pneumonia	1	0.98 (0.14 - 6.93)	0.98 (0)	-0.03 (-1.7)	0.98 (0.19)
Vorinostat	Hypogeusia	1	5.67 (0.8 - 40.31)	5.67 (3.85)	2.5 (0.84)	5.67 (1.1)
Vorinostat	Folate Deficiency	1	10.86 (1.53 - 77.2)	10.86 (8.94)	3.44 (1.77)	10.85 (2.1)
Vorinostat	Primitive Neuroectodermal Tumour	1	220.49 (30.36 - 1601.51)	220.45 (213.49)	7.75 (6.03)	215.46 (41)
Vorinostat	Immunodeficiency	1	0.86 (0.12 - 6.14)	0.86 (0.02)	-0.21 (-1.88)	0.86 (0.17)
Vorinostat	Inappropriate Antidiuretic Hormone Secretion	1	1.21 (0.17 - 8.58)	1.21 (0.04)	0.27 (-1.39)	1.21 (0.23)
Vorinostat	Immune Thrombocytopenia	1	1.29 (0.18 - 9.15)	1.29 (0.06)	0.37 (-1.3)	1.29 (0.25)
Vorinostat	Platelet Transfusion	1	9.4 (1.32 - 66.79)	9.39 (7.49)	3.23 (1.56)	9.39 (1.82)
Vorinostat	Colon Cancer	1	0.75 (0.11 - 5.35)	0.75 (0.08)	-0.41 (-2.08)	0.75 (0.15)
Vorinostat	Eustachian Tube Dysfunction	1	20.79 (2.92 - 147.95)	20.79 (18.8)	4.37 (2.7)	20.74 (4.02)
Vorinostat	Duodenal Ulcer	1	1.7 (0.24 - 12.07)	1.7 (0.29)	0.77 (-0.9)	1.7 (0.33)
Vorinostat	Full Blood Count Decreased	1	0.58 (0.08 - 4.09)	0.58 (0.31)	-0.79 (-2.46)	0.58 (0.11)
Vorinostat	Subileus	1	5.28 (0.74 - 37.52)	5.28 (3.47)	2.4 (0.73)	5.28 (1.02)
Vorinostat	Nodule	1	0.84 (0.12 - 5.95)	0.84 (0.03)	-0.25 (-1.92)	0.84 (0.16)

Vorinostat	Wernicke'S Encephalopathy	1	21.7 (3.05 - 154.4)	21.69 (19.69)	4.44 (2.76)	21.64 (4.19)
Vorinostat	Eyelid Oedema	1	0.92 (0.13 - 6.55)	0.92 (0.01)	-0.12 (-1.78)	0.92 (0.18)
Vorinostat	Glossodynia	1	0.55 (0.08 - 3.91)	0.55 (0.37)	-0.86 (-2.53)	0.55 (0.11)
Vorinostat	Mucosal Disorder	1	4.91 (0.69 - 34.92)	4.91 (3.12)	2.3 (0.63)	4.91 (0.95)
Vorinostat	Atrioventricular Block	1	1.52 (0.21 - 10.78)	1.52 (0.18)	0.6 (-1.06)	1.52 (0.29)
Vorinostat	Ileal Perforation	1	17.56 (2.47 - 124.9)	17.55 (15.58)	4.13 (2.46)	17.52 (3.39)
Vorinostat	Wound Abscess	1	32.36 (4.54 - 230.54)	32.35 (30.28)	5.01 (3.34)	32.25 (6.24)
Vorinostat	Rhabdomyolysis	1	0.29 (0.04 - 2.08)	0.29 (1.7)	-1.77 (-3.44)	0.29 (0.06)
Vorinostat	Adenoviral Haemorrhagic Cystitis	1	58.17 (8.14 - 415.5)	58.16 (55.83)	5.85 (4.17)	57.81 (11.16)
Vorinostat	Acute Graft Versus Host Disease	1	2.47 (0.35 - 17.55)	2.47 (0.88)	1.3 (-0.36)	2.47 (0.48)
Vorinostat	Post Transplant Lymphoproliferative Disorder	1	2.53 (0.36 - 17.96)	2.53 (0.92)	1.34 (-0.33)	2.53 (0.49)
Vorinostat	Brain Tumour Operation	1	56.77 (7.95 - 405.49)	56.76 (54.45)	5.82 (4.14)	56.43 (10.89)
Vorinostat	Hypothyroidism	1	0.37 (0.05 - 2.64)	0.37 (1.06)	-1.43 (-3.09)	0.37 (0.07)
Vorinostat	Transplant	1	5.04 (0.71 - 35.83)	5.04 (3.24)	2.33 (0.67)	5.04 (0.98)
Vorinostat	Systemic Inflammatory Response Syndrome	1	2.93 (0.41 - 20.8)	2.93 (1.27)	1.55 (-0.12)	2.93 (0.57)
Vorinostat	Pancreatitis Acute	1	0.54 (0.08 - 3.8)	0.54 (0.4)	-0.9 (-2.57)	0.54 (0.1)
Vorinostat	Drug Eruption	1	0.68 (0.1 - 4.86)	0.68 (0.15)	-0.55 (-2.21)	0.68 (0.13)
Vorinostat	Cervical Dysplasia	1	5.26 (0.74 - 37.34)	5.25 (3.44)	2.39 (0.73)	5.25 (1.02)
Vorinostat	Thrombophlebitis	1	3.17 (0.45 - 22.53)	3.17 (1.49)	1.66 (0)	3.17 (0.61)
Vorinostat	Brain Stem Glioma	1	75.85 (10.6 - 542.81)	75.83 (73.26)	6.23 (4.55)	75.24 (14.5)
Vorinostat	Sarcoma Metastatic	1	75.25 (10.52 - 538.46)	75.23 (72.67)	6.22 (4.54)	74.65 (14.38)
Vorinostat	Myasthenic Syndrome	1	18.2 (2.56 - 129.46)	18.19 (16.22)	4.18 (2.51)	18.16 (3.52)
Vorinostat	Hepatic Encephalopathy	1	1.22 (0.17 - 8.67)	1.22 (0.04)	0.29 (-1.38)	1.22 (0.24)
Vorinostat	Jaundice	1	0.43 (0.06 - 3.04)	0.43 (0.76)	-1.22 (-2.89)	0.43 (0.08)
Vorinostat	Melaena	1	0.51 (0.07 - 3.66)	0.51 (0.46)	-0.96 (-2.62)	0.51 (0.1)

Vorinostat	Bile Duct Stone	1	3.6 (0.51 - 25.58)	3.6 (1.88)	1.85 (0.18)	3.6 (0.7)
Vorinostat	Cholecystitis Acute	1	1.99 (0.28 - 14.15)	1.99 (0.49)	0.99 (-0.67)	1.99 (0.39)
Vorinostat	Gallbladder Cancer	1	7.17 (1.01 - 50.92)	7.17 (5.3)	2.84 (1.17)	7.16 (1.39)
Vorinostat	Hepatocellular Carcinoma	1	1.46 (0.2 - 10.33)	1.46 (0.14)	0.54 (-1.13)	1.45 (0.28)
Vorinostat	Leukaemia	1	1.21 (0.17 - 8.6)	1.21 (0.04)	0.28 (-1.39)	1.21 (0.23)
Vorinostat	Cytomegalovirus Gastritis	1	57.81 (8.09 - 412.96)	57.8 (55.48)	5.84 (4.16)	57.46 (11.09)
Vorinostat	Laryngeal Haemorrhage	1	91.16 (12.72 - 653.44)	91.15 (88.31)	6.5 (4.81)	90.29 (17.37)
Vorinostat	Obstructive Airways Disorder	1	1.02 (0.14 - 7.26)	1.02 (0)	0.03 (-1.63)	1.02 (0.2)
Vorinostat	Central Nervous System Neoplasm	1	81.04 (11.32 - 580.23)	81.02 (78.36)	6.33 (4.64)	80.34 (15.47)
Vorinostat	Mental Impairment	1	0.47 (0.07 - 3.33)	0.47 (0.6)	-1.09 (-2.76)	0.47 (0.09)
Vorinostat	White Matter Lesion	1	8.98 (1.26 - 63.81)	8.98 (7.08)	3.16 (1.5)	8.97 (1.74)
Vorinostat	Hot Flush	1	0.16 (0.02 - 1.15)	0.16 (4.35)	-2.63 (-4.29)	0.16 (0.03)
Vorinostat	Weight Increased	1	0.05 (0.01 - 0.37)	0.05 (17.28)	-4.26 (-5.93)	0.05 (0.01)
Vorinostat	Asthma	1	0.11 (0.02 - 0.8)	0.11 (6.99)	-3.15 (-4.81)	0.11 (0.02)
Vorinostat	Female Sterilisation	1	21.02 (2.95 - 149.6)	21.02 (19.02)	4.39 (2.72)	20.97 (4.06)
Vorinostat	Hepatitis C	1	0.87 (0.12 - 6.16)	0.87 (0.02)	-0.2 (-1.87)	0.87 (0.17)
Vorinostat	Hernia Repair	1	4.49 (0.63 - 31.87)	4.49 (2.71)	2.16 (0.5)	4.48 (0.87)
Vorinostat	Radiation Oesophagitis	1	30.39 (4.27 - 216.45)	30.38 (28.32)	4.92 (3.25)	30.29 (5.86)
Vorinostat	Social Problem	1	2.89 (0.41 - 20.5)	2.89 (1.23)	1.53 (-0.14)	2.89 (0.56)
Vorinostat	Peripheral Sensory Neuropathy	1	2.07 (0.29 - 14.71)	2.07 (0.55)	1.05 (-0.62)	2.07 (0.4)
Romidepsin	Peripheral T-Cell Lymphoma Unspecified	113	5540.53 (4459.65 - 6883.4)	5339.32 (447522.77)	11.95 (10.28)	3962.08 (3304.16)
Romidepsin	Death	102	2.32 (1.9 - 2.83)	2.28 (74.08)	1.19 (-0.48)	2.28 (1.93)
Romidepsin	Nausea	94	2.44 (1.99 - 3)	2.4 (77.81)	1.26 (-0.4)	2.4 (2.02)
Romidepsin	Thrombocytopenia	86	16.44 (13.26 - 20.37)	16.01 (1210.99)	4 (2.33)	15.99 (13.37)
Romidepsin	Pyrexia	78	4.59 (3.66 - 5.74)	4.5 (213.1)	2.17 (0.5)	4.49 (3.72)
Romidepsin	Platelet Count Decreased	75	14.33 (11.39 - 18.02)	14 (906.45)	3.81 (2.14)	13.99 (11.55)

Romidepsin	Vomiting	61	2.71 (2.1 - 3.49)	2.67 (64.31)	1.42 (-0.25)	2.67 (2.16)
Romidepsin	Anaemia	54	5.62 (4.3 - 7.36)	5.54 (201.61)	2.47 (0.8)	5.54 (4.42)
Romidepsin	Fatigue	51	1.27 (0.96 - 1.68)	1.27 (2.92)	0.34 (-1.32)	1.27 (1.01)
Romidepsin	Decreased Appetite	44	3.75 (2.78 - 5.05)	3.71 (87.4)	1.89 (0.22)	3.71 (2.89)
Romidepsin	Neutropenia	42	6.34 (4.67 - 8.59)	6.26 (186.1)	2.65 (0.98)	6.26 (4.85)
Romidepsin	Atrial Fibrillation	40	8.29 (6.07 - 11.33)	8.2 (253.09)	3.03 (1.37)	8.19 (6.31)
Romidepsin	Diarrhoea	38	1.18 (0.85 - 1.62)	1.17 (0.99)	0.23 (-1.44)	1.17 (0.9)
Romidepsin	Angioimmunoblastic T-Cell Lymphoma	38	3210.65 (2260.17 - 4560.83)	3171.44 (99823.64)	11.36 (9.68)	2628.75 (1959.7)
Romidepsin	Pneumonia	29	1.67 (1.16 - 2.41)	1.67 (7.8)	0.74 (-0.93)	1.67 (1.23)
Romidepsin	Product Storage Error	28	6.21 (4.28 - 9)	6.16 (121.14)	2.62 (0.96)	6.16 (4.51)
Romidepsin	Electrocardiogram Qt Prolonged	27	15.19 (10.4 - 22.19)	15.07 (354.48)	3.91 (2.25)	15.05 (10.96)
Romidepsin	White Blood Cell Count Decreased	26	4.76 (3.24 - 7.01)	4.73 (76.61)	2.24 (0.58)	4.73 (3.42)
Romidepsin	Asthenia	26	1.39 (0.94 - 2.04)	1.38 (2.78)	0.47 (-1.2)	1.38 (1)
Romidepsin	Neutrophil Count Decreased	24	12.12 (8.11 - 18.11)	12.03 (242.8)	3.59 (1.92)	12.03 (8.59)
Romidepsin	Febrile Neutropenia	23	7.22 (4.79 - 10.88)	7.17 (122.19)	2.84 (1.17)	7.17 (5.08)
Romidepsin	Malaise	23	0.97 (0.64 - 1.46)	0.97 (0.02)	-0.04 (-1.71)	0.97 (0.69)
Romidepsin	Hypotension	22	2.23 (1.46 - 3.39)	2.22 (14.77)	1.15 (-0.52)	2.22 (1.56)
Romidepsin	Sepsis	22	3.95 (2.59 - 6)	3.93 (48.04)	1.97 (0.31)	3.92 (2.76)
Romidepsin	Tumour Lysis Syndrome	22	52.54 (34.52 - 79.97)	52.18 (1100.71)	5.7 (4.03)	52 (36.59)
Romidepsin	Disease Progression	21	3.68 (2.39 - 5.65)	3.66 (40.63)	1.87 (0.2)	3.66 (2.55)
Romidepsin	Headache	21	0.65 (0.42 - 1)	0.65 (3.97)	-0.62 (-2.29)	0.65 (0.45)
Romidepsin	Cutaneous T-Cell Lymphoma	20	232.94 (149.58 - 362.76)	231.45 (4521)	7.83 (6.16)	228.02 (157.4)
Romidepsin	Acute Kidney Injury	20	2.01 (1.29 - 3.12)	2 (10.08)	1 (-0.66)	2 (1.39)
Romidepsin	Rash	19	0.83 (0.53 - 1.31)	0.83 (0.63)	-0.26 (-1.93)	0.83 (0.57)
Romidepsin	Dyspnoea	18	0.63 (0.39 - 1)	0.63 (3.95)	-0.67 (-2.33)	0.63 (0.43)

Romidepsin	Malignant Neoplasm Progression	18	3.58 (2.25 - 5.69)	3.57 (33.31)	1.83 (0.17)	3.57 (2.42)
Romidepsin	General Physical Health Deterioration	16	2.99 (1.83 - 4.89)	2.98 (21.13)	1.58 (-0.09)	2.98 (1.98)
Romidepsin	Pancytopenia	16	6.1 (3.73 - 9.97)	6.07 (67.82)	2.6 (0.93)	6.07 (4.02)
Romidepsin	Dysgeusia	15	4.05 (2.44 - 6.73)	4.04 (34.28)	2.01 (0.35)	4.03 (2.64)
Romidepsin	Respiratory Failure	15	4.22 (2.54 - 7.01)	4.2 (36.66)	2.07 (0.4)	4.2 (2.75)
Romidepsin	Chills	15	2.54 (1.53 - 4.21)	2.53 (13.88)	1.34 (-0.33)	2.53 (1.65)
Romidepsin	Fall	14	0.82 (0.48 - 1.39)	0.82 (0.56)	-0.29 (-1.95)	0.82 (0.53)
Romidepsin	Weight Decreased	14	1.01 (0.6 - 1.71)	1.01 (0)	0.02 (-1.65)	1.01 (0.65)
Romidepsin	Liver Disorder	14	6.7 (3.96 - 11.32)	6.67 (67.52)	2.74 (1.07)	6.67 (4.3)
Romidepsin	Dehydration	13	1.99 (1.15 - 3.43)	1.99 (6.37)	0.99 (-0.68)	1.99 (1.26)
Romidepsin	Pain	13	0.39 (0.22 - 0.67)	0.39 (12.56)	-1.36 (-3.03)	0.39 (0.25)
Romidepsin	T-Cell Lymphoma	13	270.17 (155.96 - 468.03)	269.05 (3411.96)	8.05 (6.38)	264.43 (166.97)
Romidepsin	Leukopenia	13	5.41 (3.14 - 9.33)	5.39 (46.54)	2.43 (0.76)	5.39 (3.42)
Romidepsin	Infection	13	1.8 (1.04 - 3.1)	1.8 (4.61)	0.85 (-0.82)	1.8 (1.14)
Romidepsin	Cardiac Failure	13	3.25 (1.88 - 5.6)	3.24 (20.12)	1.69 (0.03)	3.24 (2.05)
Romidepsin	Lymphocyte Count Decreased	13	13.66 (7.92 - 23.56)	13.61 (151.79)	3.77 (2.1)	13.6 (8.62)
Romidepsin	Pleural Effusion	12	4.01 (2.28 - 7.07)	4 (27.03)	2 (0.33)	4 (2.49)
Romidepsin	Constipation	12	1.14 (0.65 - 2.01)	1.14 (0.21)	0.19 (-1.48)	1.14 (0.71)
Romidepsin	Drug Ineffective	12	0.17 (0.1 - 0.3)	0.18 (47.68)	-2.51 (-4.18)	0.18 (0.11)
Romidepsin	Hepatic Failure	11	7.84 (4.34 - 14.18)	7.82 (65.43)	2.97 (1.3)	7.82 (4.76)
Romidepsin	Bone Marrow Failure	11	9.84 (5.44 - 17.8)	9.81 (87.03)	3.29 (1.63)	9.81 (5.97)
Romidepsin	Blood Lactate Dehydrogenase Increased	11	15.81 (8.75 - 28.59)	15.76 (151.94)	3.98 (2.31)	15.75 (9.59)
Romidepsin	Taste Disorder	11	12.74 (7.05 - 23.03)	12.7 (118.47)	3.67 (2)	12.69 (7.73)
Romidepsin	Septic Shock	10	4.81 (2.59 - 8.95)	4.8 (30.09)	2.26 (0.6)	4.8 (2.85)

Romidepsin	Epistaxis	10	2.58 (1.39 - 4.81)	2.58 (9.68)	1.37 (-0.3)	2.58 (1.53)
Romidepsin	Haematotoxicity	10	23.03 (12.37 - 42.87)	22.96 (209.76)	4.52 (2.85)	22.93 (13.63)
Romidepsin	Cellulitis	10	3.8 (2.04 - 7.07)	3.79 (20.56)	1.92 (0.26)	3.79 (2.25)
Romidepsin	Hyponatraemia	10	3.64 (1.96 - 6.77)	3.63 (19.06)	1.86 (0.19)	3.63 (2.16)
Romidepsin	Stomatitis	10	3.27 (1.76 - 6.08)	3.26 (15.68)	1.7 (0.04)	3.26 (1.94)
Romidepsin	Cytomegalovirus Infection	10	12.22 (6.57 - 22.74)	12.19 (102.62)	3.61 (1.94)	12.18 (7.24)
Romidepsin	Cardiac Failure Congestive	10	2.31 (1.24 - 4.3)	2.31 (7.4)	1.2 (-0.46)	2.31 (1.37)
Romidepsin	Aspartate Aminotransferase Increased	10	4.42 (2.38 - 8.22)	4.41 (26.36)	2.14 (0.47)	4.41 (2.62)
Romidepsin	Myelosuppression	10	9.82 (5.27 - 18.26)	9.79 (78.87)	3.29 (1.62)	9.78 (5.82)
Romidepsin	Muscular Weakness	9	1.54 (0.8 - 2.97)	1.54 (1.71)	0.62 (-1.04)	1.54 (0.89)
Romidepsin	Pulmonary Embolism	9	1.89 (0.98 - 3.64)	1.89 (3.77)	0.92 (-0.75)	1.89 (1.09)
Romidepsin	Lymphoma	9	11.34 (5.89 - 21.82)	11.31 (84.52)	3.5 (1.83)	11.3 (6.53)
Romidepsin	Cough	9	0.63 (0.33 - 1.22)	0.63 (1.91)	-0.66 (-2.32)	0.63 (0.37)
Romidepsin	Lymphopenia	9	12.73 (6.62 - 24.5)	12.7 (96.91)	3.67 (2)	12.69 (7.34)
Romidepsin	Amenorrhoea	9	10.99 (5.71 - 21.14)	10.96 (81.4)	3.45 (1.79)	10.95 (6.33)
Romidepsin	Mental Status Changes	9	6.93 (3.6 - 13.33)	6.91 (45.5)	2.79 (1.12)	6.91 (4)
Romidepsin	Neuropathy Peripheral	9	1.91 (0.99 - 3.67)	1.9 (3.86)	0.93 (-0.74)	1.9 (1.1)
Romidepsin	Cytopenia	9	16.47 (8.56 - 31.69)	16.42 (130.25)	4.04 (2.37)	16.41 (9.49)
Romidepsin	Syncope	8	1.61 (0.8 - 3.21)	1.6 (1.82)	0.68 (-0.99)	1.6 (0.9)
Romidepsin	Epstein-Barr Virus Infection	8	27.39 (13.68 - 54.85)	27.32 (202.5)	4.77 (3.1)	27.27 (15.25)
Romidepsin	Disseminated Intravascular Coagulation	8	12.44 (6.22 - 24.91)	12.42 (83.92)	3.63 (1.97)	12.41 (6.94)
Romidepsin	Erythema	8	0.73 (0.36 - 1.46)	0.73 (0.8)	-0.45 (-2.12)	0.73 (0.41)
Romidepsin	Pruritus	8	0.42 (0.21 - 0.85)	0.42 (6.3)	-1.24 (-2.9)	0.42 (0.24)
Romidepsin	Gastrointestinal Haemorrhage	8	1.77 (0.89 - 3.55)	1.77 (2.68)	0.82 (-0.84)	1.77 (0.99)

Romidepsin	Anaplastic Large-Cell Lymphoma	8	530.9 (262.14 - 1075.19)	529.53 (4079.62)	9 (7.32)	511.92 (283.63)
Romidepsin	Multiple Organ Dysfunction Syndrome	7	3.23 (1.54 - 6.79)	3.23 (10.78)	1.69 (0.02)	3.23 (1.74)
Romidepsin	Alanine Aminotransferase Increased	7	2.59 (1.24 - 5.44)	2.59 (6.84)	1.37 (-0.29)	2.59 (1.39)
Romidepsin	Dyspepsia	7	1.45 (0.69 - 3.05)	1.45 (0.99)	0.54 (-1.13)	1.45 (0.78)
Romidepsin	Hypoalbuminaemia	7	19.49 (9.28 - 40.95)	19.45 (122.39)	4.28 (2.61)	19.43 (10.44)
Romidepsin	Hypokalaemia	7	3.16 (1.5 - 6.63)	3.15 (10.3)	1.66 (-0.01)	3.15 (1.7)
Romidepsin	Mucosal Inflammation	7	5.59 (2.66 - 11.74)	5.58 (26.33)	2.48 (0.81)	5.58 (3)
Romidepsin	Gastrointestinal Disorder	7	1.16 (0.55 - 2.42)	1.15 (0.15)	0.21 (-1.46)	1.15 (0.62)
Romidepsin	Abdominal Pain	7	0.6 (0.29 - 1.26)	0.6 (1.83)	-0.73 (-2.4)	0.6 (0.32)
Romidepsin	Dizziness	7	0.28 (0.13 - 0.59)	0.28 (12.86)	-1.82 (-3.49)	0.28 (0.15)
Romidepsin	Hot Flush	7	1.92 (0.91 - 4.03)	1.92 (3.08)	0.94 (-0.73)	1.92 (1.03)
Romidepsin	Arthralgia	7	0.33 (0.16 - 0.69)	0.33 (9.55)	-1.6 (-3.26)	0.33 (0.18)
Romidepsin	Hepatic Function Abnormal	7	4.09 (1.95 - 8.58)	4.08 (16.28)	2.03 (0.36)	4.08 (2.19)
Romidepsin	Off Label Use	7	0.16 (0.08 - 0.34)	0.17 (29.77)	-2.59 (-4.26)	0.17 (0.09)
Romidepsin	Acute Myeloid Leukaemia	6	7.82 (3.51 - 17.42)	7.8 (35.59)	2.96 (1.3)	7.8 (3.99)
Romidepsin	Skin Exfoliation	6	1.44 (0.65 - 3.22)	1.44 (0.82)	0.53 (-1.14)	1.44 (0.74)
Romidepsin	Adverse Event	6	1.28 (0.57 - 2.85)	1.28 (0.36)	0.35 (-1.31)	1.28 (0.65)
Romidepsin	Clostridium Difficile Colitis	6	11.08 (4.97 - 24.7)	11.07 (54.9)	3.47 (1.8)	11.06 (5.66)
Romidepsin	Toxicity To Various Agents	6	0.65 (0.29 - 1.44)	0.65 (1.15)	-0.63 (-2.29)	0.65 (0.33)
Romidepsin	Deep Vein Thrombosis	6	1.8 (0.81 - 4)	1.79 (2.11)	0.84 (-0.82)	1.79 (0.92)
Romidepsin	Hyperglycaemia	6	3.42 (1.53 - 7.61)	3.41 (10.23)	1.77 (0.1)	3.41 (1.75)
Romidepsin	Hypocalcaemia	6	6.37 (2.86 - 14.2)	6.36 (27.11)	2.67 (1)	6.36 (3.25)
Romidepsin	Electrocardiogram Abnormal	6	15.83 (7.1 - 35.28)	15.8 (83.13)	3.98 (2.31)	15.79 (8.08)
Romidepsin	Blood Creatinine Increased	6	1.91 (0.86 - 4.25)	1.91 (2.6)	0.93 (-0.73)	1.91 (0.98)

Romidepsin	Cardiotoxicity	6	14.39 (6.46 - 32.07)	14.37 (74.55)	3.84 (2.18)	14.35 (7.34)
Romidepsin	Confusional State	6	0.75 (0.34 - 1.68)	0.76 (0.48)	-0.41 (-2.07)	0.76 (0.39)
Romidepsin	Tachycardia	6	1.4 (0.63 - 3.13)	1.4 (0.7)	0.49 (-1.18)	1.4 (0.72)
Romidepsin	Febrile Bone Marrow Aplasia	6	28.87 (12.95 - 64.37)	28.82 (160.84)	4.85 (3.18)	28.77 (14.71)
Romidepsin	Cardiac Tamponade	6	25.93 (11.63 - 57.8)	25.88 (143.29)	4.69 (3.02)	25.84 (13.21)
Romidepsin	Interstitial Lung Disease	6	2.6 (1.17 - 5.79)	2.6 (5.89)	1.38 (-0.29)	2.6 (1.33)
Romidepsin	Urinary Tract Infection	6	0.69 (0.31 - 1.53)	0.69 (0.87)	-0.54 (-2.21)	0.69 (0.35)
Romidepsin	Haemophagocytic Lymphohistiocytosis	6	13.2 (5.92 - 29.41)	13.17 (67.44)	3.72 (2.05)	13.16 (6.73)
Romidepsin	C-Reactive Protein Increased	6	3.47 (1.56 - 7.72)	3.46 (10.5)	1.79 (0.12)	3.46 (1.77)
Romidepsin	Haemoglobin Decreased	6	1.17 (0.53 - 2.61)	1.17 (0.15)	0.23 (-1.44)	1.17 (0.6)
Romidepsin	Ventricular Tachycardia	6	7.95 (3.57 - 17.71)	7.93 (36.35)	2.99 (1.32)	7.93 (4.06)
Romidepsin	Cytomegalovirus Viraemia	6	30.25 (13.57 - 67.43)	30.19 (169.02)	4.91 (3.25)	30.13 (15.41)
Romidepsin	Cytomegalovirus Infection Reactivation	6	40.21 (18.03 - 89.66)	40.13 (228.34)	5.32 (3.66)	40.03 (20.46)
Romidepsin	Hypomagnesaemia	5	7.38 (3.07 - 17.75)	7.37 (27.53)	2.88 (1.21)	7.37 (3.54)
Romidepsin	Seizure	5	0.6 (0.25 - 1.45)	0.61 (1.29)	-0.72 (-2.39)	0.61 (0.29)
Romidepsin	Graft Versus Host Disease	5	14.94 (6.21 - 35.94)	14.92 (64.88)	3.9 (2.23)	14.91 (7.15)
Romidepsin	Arrhythmia	5	2.18 (0.91 - 5.24)	2.18 (3.19)	1.12 (-0.54)	2.18 (1.05)
Romidepsin	Myocardial Infarction	5	0.6 (0.25 - 1.45)	0.6 (1.3)	-0.73 (-2.39)	0.6 (0.29)
Romidepsin	Oedema	5	1.91 (0.8 - 4.6)	1.91 (2.18)	0.94 (-0.73)	1.91 (0.92)
Romidepsin	Full Blood Count Decreased	5	4.73 (1.97 - 11.37)	4.72 (14.66)	2.24 (0.57)	4.72 (2.27)
Romidepsin	Melaena	5	4.51 (1.87 - 10.84)	4.5 (13.62)	2.17 (0.5)	4.5 (2.16)
Romidepsin	Extravasation	5	25.31 (10.52 - 60.9)	25.27 (116.38)	4.66 (2.99)	25.23 (12.1)
Romidepsin	Palpitations	5	0.86 (0.36 - 2.07)	0.86 (0.11)	-0.21 (-1.88)	0.86 (0.41)
Romidepsin	Oxygen Saturation Decreased	5	1.88 (0.78 - 4.53)	1.88 (2.06)	0.91 (-0.76)	1.88 (0.9)
Romidepsin	Lymphadenopathy	5	2.87 (1.19 - 6.9)	2.87 (6.08)	1.52 (-0.15)	2.87 (1.38)

Romidepsin	Myelodysplastic Syndrome	5	6.92 (2.88 - 16.64)	6.91 (25.28)	2.79 (1.12)	6.91 (3.32)
Romidepsin	Leukocytosis	5	5.92 (2.46 - 14.23)	5.91 (20.39)	2.56 (0.9)	5.91 (2.84)
Romidepsin	Acute Respiratory Failure	5	5.3 (2.21 - 12.76)	5.3 (17.43)	2.41 (0.74)	5.3 (2.54)
Romidepsin	Covid-19	5	0.53 (0.22 - 1.29)	0.54 (2.02)	-0.9 (-2.57)	0.54 (0.26)
Romidepsin	Drug Intolerance	5	0.99 (0.41 - 2.39)	0.99 (0)	-0.01 (-1.67)	0.99 (0.48)
Romidepsin	Intentional Product Use Issue	5	1.06 (0.44 - 2.55)	1.06 (0.02)	0.08 (-1.58)	1.06 (0.51)
Romidepsin	Diffuse Large B-Cell Lymphoma	4	11.34 (4.25 - 30.25)	11.33 (37.64)	3.5 (1.83)	11.32 (4.98)
Romidepsin	Cardiomyopathy	4	5.63 (2.11 - 15.01)	5.62 (15.21)	2.49 (0.82)	5.62 (2.47)
Romidepsin	Hyperhidrosis	4	0.62 (0.23 - 1.66)	0.62 (0.93)	-0.69 (-2.35)	0.62 (0.27)
Romidepsin	Acute Myocardial Infarction	4	2.79 (1.05 - 7.44)	2.79 (4.59)	1.48 (-0.19)	2.79 (1.23)
Romidepsin	Ageusia	4	3.14 (1.18 - 8.38)	3.14 (5.83)	1.65 (-0.02)	3.14 (1.38)
Romidepsin	Hypertension	4	0.38 (0.14 - 1.02)	0.38 (4)	-1.39 (-3.05)	0.38 (0.17)
Romidepsin	Cardio-Respiratory Arrest	4	1.97 (0.74 - 5.26)	1.97 (1.91)	0.98 (-0.69)	1.97 (0.87)
Romidepsin	Acute Pulmonary Oedema	4	15.36 (5.76 - 40.98)	15.34 (53.59)	3.94 (2.27)	15.33 (6.75)
Romidepsin	Blood Alkaline Phosphatase Increased	4	3.75 (1.41 - 10.01)	3.75 (8.07)	1.91 (0.24)	3.75 (1.65)
Romidepsin	Blood Bilirubin Increased	4	3.26 (1.22 - 8.7)	3.26 (6.27)	1.7 (0.04)	3.26 (1.43)
Romidepsin	Gamma-Glutamyltransferase Increased	4	4.08 (1.53 - 10.89)	4.08 (9.3)	2.03 (0.36)	4.08 (1.8)
Romidepsin	Hypophosphataemia	4	10.85 (4.07 - 28.94)	10.84 (35.7)	3.44 (1.77)	10.83 (4.77)
Romidepsin	Cytokine Release Syndrome	4	6.18 (2.32 - 16.47)	6.17 (17.32)	2.62 (0.96)	6.17 (2.71)
Romidepsin	Muscle Spasms	4	0.42 (0.16 - 1.12)	0.42 (3.21)	-1.25 (-2.92)	0.42 (0.18)
Romidepsin	Infusion Site Extravasation	4	11.13 (4.17 - 29.7)	11.12 (36.82)	3.47 (1.81)	11.11 (4.89)
Romidepsin	Fracture	4	3.96 (1.48 - 10.55)	3.95 (8.83)	1.98 (0.32)	3.95 (1.74)
Romidepsin	Gastrooesophageal Reflux Disease	4	0.99 (0.37 - 2.64)	0.99 (0)	-0.01 (-1.68)	0.99 (0.44)
Romidepsin	Dry Skin	4	0.61 (0.23 - 1.64)	0.61 (0.97)	-0.7 (-2.37)	0.61 (0.27)
Romidepsin	Sinus Tachycardia	4	6.04 (2.27 - 16.11)	6.04 (16.8)	2.59 (0.93)	6.03 (2.66)

Romidepsin	Alopecia	4	0.38 (0.14 - 1)	0.38 (4.12)	-1.41 (-3.07)	0.38 (0.17)
Romidepsin	Chest Discomfort	4	0.8 (0.3 - 2.13)	0.8 (0.2)	-0.32 (-1.99)	0.8 (0.35)
Romidepsin	Influenza Like Illness	4	0.91 (0.34 - 2.43)	0.91 (0.03)	-0.13 (-1.8)	0.91 (0.4)
Romidepsin	Wound Infection	4	8.53 (3.2 - 22.74)	8.52 (26.53)	3.09 (1.42)	8.51 (3.75)
Romidepsin	Oral Candidiasis	4	6.55 (2.46 - 17.47)	6.54 (18.78)	2.71 (1.04)	6.54 (2.88)
Romidepsin	Pain In Extremity	4	0.25 (0.1 - 0.68)	0.25 (8.77)	-1.97 (-3.64)	0.25 (0.11)
Romidepsin	Pneumonitis	4	3.15 (1.18 - 8.4)	3.15 (5.87)	1.65 (-0.01)	3.15 (1.39)
Romidepsin	Encephalopathy	4	3.43 (1.29 - 9.15)	3.43 (6.88)	1.78 (0.11)	3.43 (1.51)
Romidepsin	Therapy Non-Responder	4	1.52 (0.57 - 4.05)	1.52 (0.7)	0.6 (-1.07)	1.52 (0.67)
Romidepsin	Laboratory Test Abnormal	4	2.61 (0.98 - 6.97)	2.61 (3.98)	1.39 (-0.28)	2.61 (1.15)
Romidepsin	Graft Versus Host Disease In Gastrointestinal Tract	4	33.83 (12.68 - 90.29)	33.79 (126.99)	5.08 (3.41)	33.72 (14.83)
Romidepsin	Acute Graft Versus Host Disease	4	17.07 (6.4 - 45.55)	17.05 (60.39)	4.09 (2.42)	17.04 (7.5)
Romidepsin	Troponin I Increased	4	50.79 (19.02 - 135.62)	50.72 (194.33)	5.66 (3.99)	50.56 (22.23)
Romidepsin	Epstein-Barr Virus Infection Reactivation	4	86.87 (32.49 - 232.25)	86.76 (337.18)	6.43 (4.76)	86.28 (37.89)
Romidepsin	Peripheral Sensory Neuropathy	4	14.24 (5.34 - 37.99)	14.23 (49.14)	3.83 (2.16)	14.21 (6.25)
Romidepsin	Electrolyte Imbalance	3	5.5 (1.77 - 17.07)	5.5 (11.04)	2.46 (0.79)	5.5 (2.13)
Romidepsin	Hodgkin'S Disease	3	15.16 (4.88 - 47.07)	15.15 (39.61)	3.92 (2.25)	15.14 (5.87)
Romidepsin	Non-Hodgkin'S Lymphoma	3	9.94 (3.2 - 30.84)	9.93 (24.08)	3.31 (1.64)	9.92 (3.85)
Romidepsin	Haemolytic Anaemia	3	7.19 (2.32 - 22.3)	7.18 (15.96)	2.84 (1.18)	7.18 (2.78)
Romidepsin	Abdominal Pain Upper	3	0.29 (0.09 - 0.9)	0.29 (5.21)	-1.78 (-3.45)	0.29 (0.11)
Romidepsin	White Blood Cell Count Increased	3	1.6 (0.52 - 4.98)	1.6 (0.68)	0.68 (-0.99)	1.6 (0.62)
Romidepsin	Chronic Graft Versus Host Disease	3	16.53 (5.33 - 51.32)	16.52 (43.69)	4.04 (2.38)	16.5 (6.4)
Romidepsin	Retinal Detachment	3	6.69 (2.16 - 20.76)	6.68 (14.5)	2.74 (1.07)	6.68 (2.59)
Romidepsin	Invasive Ductal Breast Carcinoma	3	20.1 (6.47 - 62.41)	20.08 (54.33)	4.33 (2.66)	20.06 (7.77)
Romidepsin	Escherichia Sepsis	3	21.08 (6.79 - 65.46)	21.07 (57.26)	4.39 (2.73)	21.04 (8.15)

Romidepsin	Depression	3	0.26 (0.08 - 0.8)	0.26 (6.41)	-1.95 (-3.62)	0.26 (0.1)
Romidepsin	Hyperkalaemia	3	1.8 (0.58 - 5.59)	1.8 (1.07)	0.85 (-0.82)	1.8 (0.7)
Romidepsin	Lung Disorder	3	1.25 (0.4 - 3.88)	1.25 (0.15)	0.32 (-1.35)	1.25 (0.48)
Romidepsin	Acute Coronary Syndrome	3	7.1 (2.29 - 22.02)	7.09 (15.69)	2.83 (1.16)	7.09 (2.75)
Romidepsin	Chronic Lymphocytic Leukaemia	3	12.66 (4.08 - 39.3)	12.65 (32.16)	3.66 (1.99)	12.64 (4.9)
Romidepsin	Soft Tissue Infection	3	34.18 (11 - 106.18)	34.15 (96.33)	5.09 (3.42)	34.08 (13.2)
Romidepsin	Plasma Cell Myeloma	3	1.28 (0.41 - 3.96)	1.28 (0.18)	0.35 (-1.32)	1.28 (0.49)
Romidepsin	Pyoderma Gangrenosum	3	18.18 (5.85 - 56.42)	18.16 (48.59)	4.18 (2.51)	18.14 (7.03)
Romidepsin	Bacteraemia	3	5.32 (1.72 - 16.52)	5.32 (10.52)	2.41 (0.74)	5.32 (2.06)
Romidepsin	Myalgia	3	0.36 (0.11 - 1.1)	0.36 (3.49)	-1.49 (-3.15)	0.36 (0.14)
Romidepsin	Injection Site Extravasation	3	4.09 (1.32 - 12.69)	4.09 (6.99)	2.03 (0.36)	4.09 (1.58)
Romidepsin	Gastrointestinal Sounds Abnormal	3	12.79 (4.12 - 39.71)	12.78 (32.56)	3.68 (2.01)	12.77 (4.95)
Romidepsin	Parosmia	3	8.3 (2.68 - 25.77)	8.3 (19.24)	3.05 (1.38)	8.29 (3.21)
Romidepsin	Staphylococcal Sepsis	3	12.87 (4.15 - 39.95)	12.86 (32.79)	3.68 (2.02)	12.85 (4.98)
Romidepsin	Liver Function Test Abnormal	3	2.16 (0.7 - 6.7)	2.16 (1.87)	1.11 (-0.56)	2.16 (0.84)
Romidepsin	Dyspnoea Exertional	3	1.63 (0.53 - 5.06)	1.63 (0.73)	0.71 (-0.96)	1.63 (0.63)
Romidepsin	Haemorrhage Intracranial	3	3.73 (1.2 - 11.58)	3.73 (5.99)	1.9 (0.23)	3.73 (1.45)
Romidepsin	Skin Ulcer	3	2.26 (0.73 - 7.02)	2.26 (2.11)	1.18 (-0.49)	2.26 (0.88)
Romidepsin	Hypercalcaemia	3	4.93 (1.59 - 15.29)	4.92 (9.38)	2.3 (0.63)	4.92 (1.91)
Romidepsin	Acute Respiratory Distress Syndrome	3	3.65 (1.18 - 11.33)	3.65 (5.77)	1.87 (0.2)	3.65 (1.41)
Romidepsin	Transaminases Increased	3	2.71 (0.87 - 8.41)	2.71 (3.24)	1.44 (-0.23)	2.71 (1.05)
Romidepsin	Cardiac Disorder	3	0.62 (0.2 - 1.94)	0.63 (0.67)	-0.68 (-2.34)	0.63 (0.24)
Romidepsin	Orthostatic Hypotension	3	3.55 (1.14 - 11.01)	3.55 (5.48)	1.83 (0.16)	3.55 (1.37)
Romidepsin	Renal Failure	3	0.43 (0.14 - 1.33)	0.43 (2.28)	-1.22 (-2.89)	0.43 (0.17)
Romidepsin	Cerebrovascular Accident	3	0.37 (0.12 - 1.16)	0.37 (3.16)	-1.42 (-3.09)	0.37 (0.14)

Romidepsin	Peripheral T-Cell Lymphoma Unspecified Refractory	3	5123.76 (1386.45 - 18935.3)	5118.82 (11512.85)	11.91 (10.06)	3839.36 (1286.05)
Romidepsin	Back Pain	3	0.25 (0.08 - 0.77)	0.25 (6.83)	-2.01 (-3.67)	0.25 (0.1)
Romidepsin	Urticaria	3	0.37 (0.12 - 1.16)	0.37 (3.14)	-1.42 (-3.08)	0.37 (0.15)
Romidepsin	Herpes Zoster	3	0.98 (0.32 - 3.05)	0.98 (0)	-0.02 (-1.69)	0.98 (0.38)
Romidepsin	Sudden Death	3	6.25 (2.02 - 19.41)	6.25 (13.22)	2.64 (0.98)	6.25 (2.42)
Romidepsin	Mitral Valve Incompetence	3	6.19 (2 - 19.22)	6.19 (13.05)	2.63 (0.96)	6.19 (2.4)
Romidepsin	Pulmonary Oedema	3	1.37 (0.44 - 4.24)	1.37 (0.29)	0.45 (-1.22)	1.37 (0.53)
Romidepsin	Bundle Branch Block Left	3	15.29 (4.93 - 47.46)	15.27 (39.98)	3.93 (2.26)	15.26 (5.92)
Romidepsin	Conjunctivitis	3	3.39 (1.09 - 10.53)	3.39 (5.06)	1.76 (0.1)	3.39 (1.32)
Romidepsin	Mouth Haemorrhage	3	8.45 (2.72 - 26.22)	8.44 (19.67)	3.08 (1.41)	8.44 (3.27)
Romidepsin	Condition Aggravated	3	0.21 (0.07 - 0.65)	0.21 (8.97)	-2.25 (-3.92)	0.21 (0.08)
Romidepsin	Femur Fracture	3	1.74 (0.56 - 5.38)	1.73 (0.93)	0.79 (-0.87)	1.73 (0.67)
Romidepsin	Bradycardia	3	1.15 (0.37 - 3.57)	1.15 (0.06)	0.2 (-1.47)	1.15 (0.45)
Romidepsin	Graft Versus Host Disease In Skin	3	26.49 (8.53 - 82.25)	26.46 (73.37)	4.72 (3.06)	26.42 (10.24)
Romidepsin	Pneumonia Klebsiella	3	34.59 (11.14 - 107.46)	34.56 (97.55)	5.11 (3.44)	34.49 (13.36)
Romidepsin	Staphylococcal Infection	3	1.86 (0.6 - 5.76)	1.86 (1.18)	0.89 (-0.77)	1.86 (0.72)
Romidepsin	Cystitis Haemorrhagic	3	15.55 (5.01 - 48.27)	15.54 (40.77)	3.96 (2.29)	15.52 (6.02)
Romidepsin	Pneumonia Aspiration	3	2.45 (0.79 - 7.61)	2.45 (2.58)	1.29 (-0.37)	2.45 (0.95)
Romidepsin	Vision Blurred	3	0.45 (0.14 - 1.39)	0.45 (2.03)	-1.16 (-2.82)	0.45 (0.17)
Romidepsin	Therapeutic Response Decreased	3	1.02 (0.33 - 3.15)	1.02 (0)	0.02 (-1.64)	1.02 (0.39)
Romidepsin	Pancreatitis	3	1.18 (0.38 - 3.67)	1.18 (0.08)	0.24 (-1.43)	1.18 (0.46)
Romidepsin	Cytomegalovirus Chorioretinitis	3	33.34 (10.73 - 103.57)	33.31 (93.82)	5.05 (3.39)	33.24 (12.88)
Romidepsin	Red Blood Cell Count Decreased	3	2.08 (0.67 - 6.46)	2.08 (1.68)	1.06 (-0.61)	2.08 (0.81)
Romidepsin	Hypersensitivity	3	0.32 (0.1 - 0.98)	0.32 (4.42)	-1.66 (-3.32)	0.32 (0.12)
Romidepsin	Chest Pain	3	0.33 (0.11 - 1.03)	0.33 (4.01)	-1.58 (-3.25)	0.33 (0.13)
Romidepsin	Haemoglobin Abnormal	3	9.43 (3.04 - 29.26)	9.42 (22.57)	3.24 (1.57)	9.42 (3.65)

Romidepsin	Platelet Count Abnormal	3	9.74 (3.14 - 30.24)	9.74 (23.5)	3.28 (1.62)	9.73 (3.77)
Romidepsin	Lung Infiltration	3	7.74 (2.49 - 24.02)	7.73 (17.58)	2.95 (1.28)	7.73 (3)
Romidepsin	Periorbital Oedema	3	11.86 (3.82 - 36.82)	11.85 (29.79)	3.57 (1.9)	11.84 (4.59)
Romidepsin	Electrocardiogram St Segment Depression	3	29.69 (9.56 - 92.22)	29.66 (82.94)	4.89 (3.22)	29.61 (11.47)
Romidepsin	Cardiopulmonary Failure	3	15.31 (4.93 - 47.52)	15.3 (40.04)	3.93 (2.27)	15.28 (5.92)
Romidepsin	Injection Site Reaction	3	0.88 (0.28 - 2.72)	0.88 (0.05)	-0.19 (-1.86)	0.88 (0.34)
Romidepsin	Stevens-Johnson Syndrome	3	3.13 (1.01 - 9.71)	3.13 (4.34)	1.64 (-0.02)	3.13 (1.21)
Romidepsin	Hepatitis B	3	10.05 (3.24 - 31.18)	10.04 (24.4)	3.33 (1.66)	10.03 (3.89)
Romidepsin	Splenomegaly	3	5.18 (1.67 - 16.07)	5.18 (10.11)	2.37 (0.7)	5.17 (2.01)
Romidepsin	Blood Glucose Increased	2	0.22 (0.05 - 0.88)	0.22 (5.54)	-2.18 (-3.85)	0.22 (0.07)
Romidepsin	Product Label Confusion	2	6.81 (1.7 - 27.24)	6.8 (9.9)	2.77 (1.1)	6.8 (2.13)
Romidepsin	Splenic Infarction	2	23.92 (5.97 - 95.77)	23.9 (43.82)	4.58 (2.91)	23.87 (7.47)
Romidepsin	Myocarditis	2	3.99 (1 - 15.97)	3.99 (4.48)	2 (0.33)	3.99 (1.25)
Romidepsin	Blood Magnesium Decreased	2	4.68 (1.17 - 18.71)	4.67 (5.77)	2.22 (0.56)	4.67 (1.46)
Romidepsin	Coma	2	0.91 (0.23 - 3.63)	0.91 (0.02)	-0.14 (-1.81)	0.91 (0.28)
Romidepsin	Metabolic Acidosis	2	1.33 (0.33 - 5.32)	1.33 (0.16)	0.41 (-1.26)	1.33 (0.42)
Romidepsin	Sinus Bradycardia	2	4.37 (1.09 - 17.47)	4.36 (5.19)	2.13 (0.46)	4.36 (1.37)
Romidepsin	Malignant Pleural Effusion	2	23.82 (5.95 - 95.4)	23.81 (43.63)	4.57 (2.9)	23.77 (7.45)
Romidepsin	Lip Swelling	2	1.16 (0.29 - 4.65)	1.16 (0.05)	0.22 (-1.45)	1.16 (0.36)
Romidepsin	International Normalised Ratio Increased	2	1.48 (0.37 - 5.91)	1.48 (0.31)	0.56 (-1.1)	1.48 (0.46)
Romidepsin	Feeling Hot	2	0.63 (0.16 - 2.52)	0.63 (0.43)	-0.67 (-2.33)	0.63 (0.2)
Romidepsin	Mouth Ulceration	2	1.97 (0.49 - 7.9)	1.97 (0.96)	0.98 (-0.69)	1.97 (0.62)
Romidepsin	Rash Macular	2	1.17 (0.29 - 4.68)	1.17 (0.05)	0.23 (-1.44)	1.17 (0.37)
Romidepsin	Non-Hodgkin'S Lymphoma Recurrent	2	74.05 (18.45 - 297.23)	74.01 (143.35)	6.2 (4.53)	73.66 (23.03)

Romidepsin	Cardiac Failure Acute	2	6.28 (1.57 - 25.13)	6.28 (8.87)	2.65 (0.98)	6.27 (1.97)
Romidepsin	Hepatic Enzyme Increased	2	0.62 (0.15 - 2.47)	0.62 (0.47)	-0.69 (-2.36)	0.62 (0.19)
Romidepsin	Paraesthesia	2	0.25 (0.06 - 0.98)	0.25 (4.63)	-2.02 (-3.69)	0.25 (0.08)
Romidepsin	Small Intestinal Obstruction	2	3.3 (0.83 - 13.22)	3.3 (3.21)	1.72 (0.06)	3.3 (1.03)
Romidepsin	Prostate Cancer	2	0.4 (0.1 - 1.62)	0.41 (1.75)	-1.3 (-2.97)	0.41 (0.13)
Romidepsin	Oesophagitis	2	4.01 (1 - 16.04)	4.01 (4.51)	2 (0.34)	4.01 (1.26)
Romidepsin	Upper Respiratory Tract Infection	2	0.85 (0.21 - 3.39)	0.85 (0.05)	-0.24 (-1.9)	0.85 (0.27)
Romidepsin	Peripheral T-Cell Lymphoma Unspecified Stage Iv	2	1024.42 (244.71 - 4288.52)	1023.76 (1915.81)	9.91 (8.17)	959.84 (289.66)
Romidepsin	Systemic Inflammatory Response Syndrome	2	10.09 (2.52 - 40.36)	10.08 (16.35)	3.33 (1.67)	10.07 (3.16)
Romidepsin	Blood Pressure Systolic Decreased	2	9.77 (2.44 - 39.12)	9.77 (15.73)	3.29 (1.62)	9.76 (3.06)
Romidepsin	Non-Small Cell Lung Cancer	2	8.77 (2.19 - 35.08)	8.76 (13.74)	3.13 (1.46)	8.76 (2.74)
Romidepsin	Endocarditis	2	8.01 (2 - 32.04)	8 (12.25)	3 (1.33)	8 (2.51)
Romidepsin	Eye Irritation	2	0.77 (0.19 - 3.07)	0.77 (0.14)	-0.38 (-2.05)	0.77 (0.24)
Romidepsin	Fungal Infection	2	1.16 (0.29 - 4.66)	1.16 (0.05)	0.22 (-1.45)	1.16 (0.36)
Romidepsin	Blood Pressure Diastolic Decreased	2	5.21 (1.3 - 20.84)	5.21 (6.8)	2.38 (0.71)	5.21 (1.63)
Romidepsin	Blood Test Abnormal	2	2.76 (0.69 - 11.04)	2.76 (2.24)	1.46 (-0.2)	2.76 (0.86)
Romidepsin	Blood Urine Present	2	1.99 (0.5 - 7.97)	1.99 (0.99)	0.99 (-0.67)	1.99 (0.62)
Romidepsin	Body Temperature Decreased	2	3.75 (0.94 - 15.01)	3.75 (4.03)	1.91 (0.24)	3.75 (1.17)
Romidepsin	Gingival Bleeding	2	3.08 (0.77 - 12.34)	3.08 (2.81)	1.62 (-0.04)	3.08 (0.97)
Romidepsin	Haematochezia	2	0.72 (0.18 - 2.89)	0.72 (0.21)	-0.47 (-2.14)	0.72 (0.23)
Romidepsin	Scleral Haemorrhage	2	179.72 (44.57 - 724.78)	179.61 (351.12)	7.47 (5.79)	177.54 (55.28)
Romidepsin	Skin Burning Sensation	2	0.51 (0.13 - 2.05)	0.51 (0.93)	-0.96 (-2.63)	0.51 (0.16)
Romidepsin	Subcutaneous Abscess	2	7.91 (1.98 - 31.67)	7.91 (12.07)	2.98 (1.32)	7.91 (2.48)
Romidepsin	Atrioventricular Block Complete	2	6.46 (1.62 - 25.86)	6.46 (9.22)	2.69 (1.02)	6.46 (2.02)

Romidepsin	Klebsiella Infection	2	8.6 (2.15 - 34.42)	8.6 (13.42)	3.1 (1.44)	8.59 (2.69)
Romidepsin	Squamous Cell Carcinoma Of Skin	2	6.55 (1.64 - 26.19)	6.54 (9.39)	2.71 (1.04)	6.54 (2.05)
Romidepsin	Lymphocytosis	2	19.26 (4.81 - 77.09)	19.24 (34.55)	4.26 (2.6)	19.22 (6.02)
Romidepsin	Facial Paralysis	2	2.85 (0.71 - 11.4)	2.85 (2.4)	1.51 (-0.16)	2.85 (0.89)
Romidepsin	Hypoaesthesia	2	0.26 (0.06 - 1.03)	0.26 (4.27)	-1.95 (-3.62)	0.26 (0.08)
Romidepsin	Anxiety	2	0.13 (0.03 - 0.54)	0.13 (11.17)	-2.89 (-4.56)	0.13 (0.04)
Romidepsin	Contusion	2	0.4 (0.1 - 1.62)	0.41 (1.75)	-1.3 (-2.97)	0.41 (0.13)
Romidepsin	Skin Irritation	2	0.78 (0.19 - 3.11)	0.78 (0.13)	-0.36 (-2.03)	0.78 (0.24)
Romidepsin	Loss Of Consciousness	2	0.33 (0.08 - 1.32)	0.33 (2.74)	-1.6 (-3.27)	0.33 (0.1)
Romidepsin	Pericardial Effusion	2	1.81 (0.45 - 7.25)	1.81 (0.73)	0.86 (-0.81)	1.81 (0.57)
Romidepsin	Blood Pressure Decreased	2	0.62 (0.16 - 2.5)	0.62 (0.45)	-0.68 (-2.34)	0.62 (0.2)
Romidepsin	Glomerular Filtration Rate Decreased	2	3.44 (0.86 - 13.76)	3.44 (3.46)	1.78 (0.11)	3.44 (1.08)
Romidepsin	Intentional Overdose	2	0.64 (0.16 - 2.57)	0.64 (0.4)	-0.64 (-2.31)	0.64 (0.2)
Romidepsin	Lymphocytic Leukaemia	2	99.14 (24.67 - 398.35)	99.07 (192.92)	6.62 (4.95)	98.45 (30.75)
Romidepsin	Gastritis	2	1.54 (0.38 - 6.16)	1.54 (0.38)	0.62 (-1.04)	1.54 (0.48)
Romidepsin	Influenza	2	0.36 (0.09 - 1.45)	0.36 (2.24)	-1.46 (-3.13)	0.36 (0.11)
Romidepsin	Respiratory Distress	2	1.51 (0.38 - 6.04)	1.51 (0.34)	0.59 (-1.07)	1.51 (0.47)
Romidepsin	Transient Ischaemic Attack	2	1.25 (0.31 - 5.02)	1.25 (0.1)	0.33 (-1.34)	1.25 (0.39)
Romidepsin	Cholecystitis	2	3.43 (0.86 - 13.71)	3.42 (3.43)	1.78 (0.11)	3.42 (1.07)
Romidepsin	Acute Graft Versus Host Disease In Skin	2	15.2 (3.8 - 60.84)	15.19 (26.49)	3.92 (2.26)	15.18 (4.75)
Romidepsin	Skin Lesion	2	1.45 (0.36 - 5.79)	1.45 (0.28)	0.53 (-1.13)	1.45 (0.45)
Romidepsin	Ejection Fraction Decreased	2	2.66 (0.66 - 10.63)	2.65 (2.06)	1.41 (-0.26)	2.65 (0.83)
Romidepsin	Abscess	2	2.46 (0.61 - 9.84)	2.46 (1.73)	1.3 (-0.37)	2.46 (0.77)
Romidepsin	Cutaneous T-Cell Lymphoma Recurrent	2	472.81 (115.72 - 1931.83)	472.51 (912.93)	8.84 (7.14)	458.43 (141.19)

Romidepsin	Rash Maculo-Papular	2	1.93 (0.48 - 7.72)	1.93 (0.9)	0.95 (-0.72)	1.93 (0.6)
Romidepsin	Swollen Tongue	2	1.29 (0.32 - 5.18)	1.29 (0.13)	0.37 (-1.3)	1.29 (0.41)
Romidepsin	Ascites	2	1.37 (0.34 - 5.5)	1.37 (0.2)	0.46 (-1.21)	1.37 (0.43)
Romidepsin	Liver Function Test Increased	2	1.93 (0.48 - 7.71)	1.93 (0.89)	0.95 (-0.72)	1.93 (0.6)
Romidepsin	Cardiogenic Shock	2	3 (0.75 - 12.02)	3 (2.67)	1.59 (-0.08)	3 (0.94)
Romidepsin	Deafness	2	1.54 (0.38 - 6.15)	1.54 (0.38)	0.62 (-1.05)	1.54 (0.48)
Romidepsin	Hypoxia	2	1.21 (0.3 - 4.84)	1.21 (0.07)	0.27 (-1.39)	1.21 (0.38)
Romidepsin	Haemorrhage	2	0.38 (0.09 - 1.52)	0.38 (2.03)	-1.4 (-3.06)	0.38 (0.12)
Romidepsin	Metastases To Central Nervous System	2	3.27 (0.82 - 13.09)	3.27 (3.15)	1.71 (0.04)	3.27 (1.02)
Romidepsin	Dilated Cardiomyopathy	2	7.98 (2 - 31.95)	7.98 (12.2)	3 (1.33)	7.98 (2.5)
Romidepsin	Tumour Associated Fever	2	103.13 (25.66 - 414.47)	103.06 (200.8)	6.68 (5)	102.38 (31.97)
Romidepsin	Blood Urea Increased	2	2.76 (0.69 - 11.05)	2.76 (2.25)	1.47 (-0.2)	2.76 (0.87)
Romidepsin	Blood Potassium Increased	2	2.61 (0.65 - 10.43)	2.61 (1.98)	1.38 (-0.28)	2.61 (0.82)
Romidepsin	Delirium	2	1.22 (0.31 - 4.88)	1.22 (0.08)	0.29 (-1.38)	1.22 (0.38)
Romidepsin	Adult T-Cell Lymphoma/Leukaemia	2	77.61 (19.33 - 311.54)	77.56 (150.38)	6.27 (4.6)	77.17 (24.12)
Romidepsin	Atrioventricular Block Second Degree	2	13.81 (3.45 - 55.29)	13.8 (23.73)	3.79 (2.12)	13.79 (4.32)
Romidepsin	Lethargy	2	0.71 (0.18 - 2.84)	0.71 (0.24)	-0.49 (-2.16)	0.71 (0.22)
Romidepsin	Inflammation	2	0.81 (0.2 - 3.24)	0.81 (0.09)	-0.3 (-1.97)	0.81 (0.25)
Romidepsin	Hyperuricaemia	2	9.59 (2.4 - 38.4)	9.59 (15.38)	3.26 (1.59)	9.58 (3)
Romidepsin	Squamous Cell Carcinoma	2	4.19 (1.05 - 16.77)	4.19 (4.86)	2.07 (0.4)	4.19 (1.31)
Romidepsin	Conjunctival Haemorrhage	2	10.35 (2.59 - 41.42)	10.34 (16.87)	3.37 (1.7)	10.34 (3.24)
Romidepsin	Amylase Increased	2	8.12 (2.03 - 32.48)	8.11 (12.47)	3.02 (1.35)	8.11 (2.54)
Romidepsin	Pulmonary Nocardiosis	2	60.86 (15.17 - 244.11)	60.82 (117.21)	5.92 (4.25)	60.58 (18.95)

Romidepsin	Hepatic Mass	2	21.28 (5.32 - 85.22)	21.27 (38.58)	4.41 (2.74)	21.24 (6.65)
Romidepsin	Upper Gastrointestinal Haemorrhage	2	2.23 (0.56 - 8.93)	2.23 (1.36)	1.16 (-0.51)	2.23 (0.7)
Romidepsin	Ear Pain	2	2.01 (0.5 - 8.03)	2.01 (1.01)	1 (-0.66)	2.01 (0.63)
Romidepsin	Hepatocellular Injury	2	2.42 (0.6 - 9.67)	2.42 (1.66)	1.27 (-0.39)	2.42 (0.76)
Romidepsin	Blood Magnesium Abnormal	2	74.23 (18.49 - 297.95)	74.19 (143.71)	6.21 (4.53)	73.83 (23.08)
Romidepsin	Peripheral Motor Neuropathy	2	29.02 (7.24 - 116.24)	29 (53.97)	4.86 (3.19)	28.95 (9.06)
Romidepsin	Respiratory Tract Oedema	2	53.92 (13.45 - 216.21)	53.88 (103.44)	5.75 (4.08)	53.7 (16.8)
Romidepsin	Campylobacter Infection	2	50.63 (12.63 - 203)	50.6 (96.92)	5.66 (3.99)	50.44 (15.78)
Romidepsin	Unevaluable Event	2	0.48 (0.12 - 1.91)	0.48 (1.14)	-1.06 (-2.73)	0.48 (0.15)
Romidepsin	Hepatic Cirrhosis	2	2.26 (0.56 - 9.04)	2.26 (1.4)	1.18 (-0.49)	2.26 (0.71)
Romidepsin	Cerebral Haemorrhage	2	1.11 (0.28 - 4.44)	1.11 (0.02)	0.15 (-1.52)	1.11 (0.35)
Romidepsin	Cd34 Cell Count Decreased	2	6146.53 (1192.03 - 31693.73)	6142.58 (8772.26)	12.1 (10.15)	4387.85 (1112.27)
Romidepsin	Ulcer	2	2.59 (0.65 - 10.34)	2.58 (1.94)	1.37 (-0.3)	2.58 (0.81)
Romidepsin	Cancer In Remission	2	156 (38.72 - 628.45)	155.9 (304.73)	7.27 (5.59)	154.35 (48.1)
Romidepsin	Localised Oedema	2	8.57 (2.14 - 34.29)	8.56 (13.36)	3.1 (1.43)	8.56 (2.68)
Romidepsin	Supraventricular Tachycardia	2	4.42 (1.1 - 17.67)	4.41 (5.28)	2.14 (0.47)	4.41 (1.38)
Romidepsin	Cardiac Dysfunction	2	11.96 (2.99 - 47.88)	11.96 (20.06)	3.58 (1.91)	11.95 (3.74)
Romidepsin	Urinary Retention	2	1.22 (0.31 - 4.89)	1.22 (0.08)	0.29 (-1.38)	1.22 (0.38)
Romidepsin	Product Preparation Issue	2	7.99 (2 - 31.98)	7.99 (12.22)	3 (1.33)	7.98 (2.5)
Romidepsin	Drug Resistance	2	1.55 (0.39 - 6.18)	1.54 (0.38)	0.63 (-1.04)	1.54 (0.48)
Romidepsin	Product Preparation Error	2	2.19 (0.55 - 8.75)	2.19 (1.29)	1.13 (-0.54)	2.19 (0.69)
Romidepsin	Organising Pneumonia	2	8.21 (2.05 - 32.86)	8.21 (12.65)	3.04 (1.37)	8.2 (2.57)
Romidepsin	Tremor	2	0.24 (0.06 - 0.96)	0.24 (4.78)	-2.05 (-3.72)	0.24 (0.08)
Romidepsin	Gait Inability	2	0.69 (0.17 - 2.75)	0.69 (0.28)	-0.54 (-2.2)	0.69 (0.22)
Romidepsin	Hypophagia	2	1.5 (0.38 - 6.02)	1.5 (0.34)	0.59 (-1.08)	1.5 (0.47)

Romidepsin	Electrocardiogram T Wave Inversion	2	19.99 (4.99 - 80.06)	19.98 (36.02)	4.32 (2.65)	19.96 (6.25)
Romidepsin	Neoplasm Progression	2	1.01 (0.25 - 4.03)	1.01 (0)	0.01 (-1.66)	1.01 (0.32)
Romidepsin	Oedema Peripheral	2	0.34 (0.08 - 1.36)	0.34 (2.56)	-1.56 (-3.22)	0.34 (0.11)
Romidepsin	Atrioventricular Block	2	5.46 (1.37 - 21.86)	5.46 (7.28)	2.45 (0.78)	5.46 (1.71)
Romidepsin	Oropharyngeal Candidiasis	2	42.8 (10.68 - 171.55)	42.78 (81.37)	5.41 (3.74)	42.66 (13.35)
Romidepsin	Drug Eruption	2	2.4 (0.6 - 9.59)	2.4 (1.63)	1.26 (-0.41)	2.4 (0.75)
Romidepsin	Urine Output Decreased	2	4.77 (1.19 - 19.08)	4.77 (5.95)	2.25 (0.59)	4.76 (1.49)
Romidepsin	Bradyarrhythmia	2	26.2 (6.54 - 104.93)	26.18 (48.36)	4.71 (3.04)	26.14 (8.19)
Romidepsin	Pharyngitis	2	3.06 (0.77 - 12.26)	3.06 (2.78)	1.61 (-0.05)	3.06 (0.96)
Romidepsin	Dry Mouth	2	0.51 (0.13 - 2.03)	0.51 (0.96)	-0.98 (-2.65)	0.51 (0.16)
Romidepsin	Flatulence	2	0.73 (0.18 - 2.91)	0.73 (0.21)	-0.46 (-2.13)	0.73 (0.23)
Romidepsin	Photophobia	2	2.21 (0.55 - 8.84)	2.21 (1.32)	1.14 (-0.52)	2.21 (0.69)
Romidepsin	Atrial Flutter	2	5 (1.25 - 20.01)	5 (6.4)	2.32 (0.65)	5 (1.57)
Romidepsin	Cutaneous T-Cell Lymphoma Stage Iv	2	668.1 (162.11 - 2753.46)	667.67 (1275.88)	9.32 (7.6)	639.89 (195.65)
Romidepsin	Restlessness	2	1.1 (0.27 - 4.39)	1.1 (0.02)	0.13 (-1.53)	1.1 (0.34)
Romidepsin	Feeling Abnormal	2	0.16 (0.04 - 0.63)	0.16 (9.06)	-2.67 (-4.33)	0.16 (0.05)
Romidepsin	Therapeutic Response Unexpected	2	0.79 (0.2 - 3.17)	0.79 (0.11)	-0.33 (-2)	0.79 (0.25)
Romidepsin	Bedridden	1	1.49 (0.21 - 10.58)	1.49 (0.16)	0.57 (-1.09)	1.49 (0.29)
Romidepsin	Memory Impairment	1	0.14 (0.02 - 0.97)	0.14 (5.48)	-2.87 (-4.54)	0.14 (0.03)
Romidepsin	Pulmonary Sepsis	1	13.47 (1.9 - 95.77)	13.47 (11.53)	3.75 (2.08)	13.46 (2.61)
Romidepsin	Disseminated Cytomegaloviral Infection	1	98.47 (13.78 - 703.69)	98.44 (95.83)	6.61 (4.93)	97.82 (18.87)
Romidepsin	Enterococcal Infection	1	4.71 (0.66 - 33.46)	4.71 (2.92)	2.24 (0.57)	4.71 (0.91)
Romidepsin	Intracranial Haematoma	1	35.31 (4.96 - 251.34)	35.3 (33.25)	5.14 (3.47)	35.22 (6.82)
Romidepsin	Brain Natriuretic Peptide Increased	1	6.72 (0.95 - 47.72)	6.71 (4.86)	2.75 (1.08)	6.71 (1.3)

Romidepsin	Device Related Infection	1	1.12 (0.16 - 7.97)	1.12 (0.01)	0.17 (-1.5)	1.12 (0.22)
Romidepsin	Peripheral Swelling	1	0.12 (0.02 - 0.86)	0.12 (6.39)	-3.05 (-4.71)	0.12 (0.02)
Romidepsin	Swelling	1	0.17 (0.02 - 1.21)	0.17 (4.03)	-2.55 (-4.21)	0.17 (0.03)
Romidepsin	Bacterial Infection	1	1.14 (0.16 - 8.07)	1.14 (0.02)	0.18 (-1.48)	1.14 (0.22)
Romidepsin	Discomfort	1	0.31 (0.04 - 2.21)	0.31 (1.53)	-1.69 (-3.35)	0.31 (0.06)
Romidepsin	Treatment Failure	1	0.24 (0.03 - 1.68)	0.24 (2.46)	-2.08 (-3.74)	0.24 (0.05)
Romidepsin	Hypernatraemia	1	4.12 (0.58 - 29.28)	4.12 (2.36)	2.04 (0.38)	4.12 (0.8)
Romidepsin	Peripheral Sensorimotor Neuropathy	1	18.33 (2.58 - 130.33)	18.33 (16.36)	4.19 (2.52)	18.3 (3.55)
Romidepsin	Haematology Test Abnormal	1	63.48 (8.9 - 452.61)	63.46 (61.22)	5.98 (4.3)	63.2 (12.21)
Romidepsin	Clostridial Sepsis	1	57.32 (8.04 - 408.54)	57.3 (55.11)	5.84 (4.16)	57.09 (11.04)
Romidepsin	Infusion Site Cellulitis	1	45.99 (6.46 - 327.57)	45.98 (43.87)	5.52 (3.84)	45.84 (8.87)
Romidepsin	Infusion Site Oedema	1	23.38 (3.29 - 166.29)	23.37 (21.38)	4.54 (2.87)	23.34 (4.52)
Romidepsin	Infusion Site Pruritus	1	6.91 (0.97 - 49.11)	6.91 (5.05)	2.79 (1.12)	6.91 (1.34)
Romidepsin	Infusion Site Ulcer	1	229.27 (31.82 - 1652.1)	229.2 (223.86)	7.82 (6.12)	225.84 (43.27)
Romidepsin	Enteritis	1	3.09 (0.43 - 21.93)	3.09 (1.41)	1.63 (-0.04)	3.09 (0.6)
Romidepsin	Rectal Polyp	1	17.2 (2.42 - 122.29)	17.2 (15.24)	4.1 (2.43)	17.18 (3.33)
Romidepsin	Tongue Oedema	1	5.27 (0.74 - 37.45)	5.27 (3.46)	2.4 (0.73)	5.27 (1.02)
Romidepsin	Non-Cardiac Chest Pain	1	6.38 (0.9 - 45.36)	6.38 (4.54)	2.67 (1.01)	6.38 (1.24)
Romidepsin	Mediastinum Neoplasm	1	124.89 (17.45 - 893.99)	124.85 (121.87)	6.95 (5.27)	123.85 (23.86)
Romidepsin	Product Label Issue	1	2.89 (0.41 - 20.52)	2.89 (1.23)	1.53 (-0.14)	2.89 (0.56)
Romidepsin	Product Packaging Quantity Issue	1	2.14 (0.3 - 15.21)	2.14 (0.61)	1.1 (-0.57)	2.14 (0.42)
Romidepsin	Leukaemia	1	2.05 (0.29 - 14.54)	2.05 (0.54)	1.03 (-0.63)	2.05 (0.4)
Romidepsin	Nasopharyngitis	1	0.1 (0.01 - 0.74)	0.1 (7.69)	-3.26 (-4.92)	0.1 (0.02)

Romidepsin	Palmar-Plantar Erythrodysaesthesia Syndrome	1	0.81 (0.11 - 5.76)	0.81 (0.04)	-0.3 (-1.97)	0.81 (0.16)
Romidepsin	Blood Potassium Decreased	1	0.68 (0.1 - 4.82)	0.68 (0.15)	-0.56 (-2.23)	0.68 (0.13)
Romidepsin	Myocardial Necrosis Marker Increased	1	9.57 (1.35 - 68.01)	9.57 (7.67)	3.26 (1.59)	9.56 (1.85)
Romidepsin	Oral Mucosal Blistering	1	3.03 (0.43 - 21.54)	3.03 (1.36)	1.6 (-0.07)	3.03 (0.59)
Romidepsin	Radiation Necrosis	1	40.11 (5.63 - 285.56)	40.1 (38.02)	5.32 (3.65)	39.99 (7.74)
Romidepsin	Renal Tubular Necrosis	1	2.2 (0.31 - 15.62)	2.2 (0.65)	1.14 (-0.53)	2.2 (0.43)
Romidepsin	Dermatitis Acneiform	1	3.39 (0.48 - 24.08)	3.39 (1.68)	1.76 (0.09)	3.39 (0.66)
Romidepsin	Nephropathy Toxic	1	1.93 (0.27 - 13.71)	1.93 (0.45)	0.95 (-0.72)	1.93 (0.37)
Romidepsin	T-Cell Lymphoma Recurrent	1	349.12 (48.08 - 2534.83)	349.01 (339.3)	8.41 (6.69)	341.28 (64.97)
Romidepsin	Urosepsis	1	2.15 (0.3 - 15.26)	2.15 (0.61)	1.1 (-0.56)	2.15 (0.42)
Romidepsin	Neutropenic Sepsis	1	2.73 (0.38 - 19.39)	2.73 (1.1)	1.45 (-0.22)	2.73 (0.53)
Romidepsin	Visual Impairment	1	0.15 (0.02 - 1.1)	0.15 (4.63)	-2.69 (-4.36)	0.15 (0.03)
Romidepsin	Anisocoria	1	14.04 (1.98 - 99.8)	14.04 (12.1)	3.81 (2.14)	14.03 (2.72)
Romidepsin	Skin Infection	1	1.76 (0.25 - 12.52)	1.76 (0.33)	0.82 (-0.85)	1.76 (0.34)
Romidepsin	Peritonitis Bacterial	1	1.89 (0.27 - 13.44)	1.89 (0.42)	0.92 (-0.75)	1.89 (0.37)
Romidepsin	Eye Haemorrhage	1	1.45 (0.2 - 10.33)	1.45 (0.14)	0.54 (-1.13)	1.45 (0.28)
Romidepsin	Eye Swelling	1	0.54 (0.08 - 3.85)	0.54 (0.39)	-0.88 (-2.55)	0.54 (0.11)
Romidepsin	Neck Pain	1	0.35 (0.05 - 2.49)	0.35 (1.21)	-1.51 (-3.18)	0.35 (0.07)
Romidepsin	Skin Discolouration	1	0.42 (0.06 - 2.99)	0.42 (0.79)	-1.25 (-2.91)	0.42 (0.08)
Romidepsin	Skin Fissures	1	1.23 (0.17 - 8.73)	1.23 (0.04)	0.3 (-1.37)	1.23 (0.24)
Romidepsin	Swelling Face	1	0.31 (0.04 - 2.23)	0.31 (1.5)	-1.67 (-3.34)	0.31 (0.06)
Romidepsin	Cytokine Storm	1	30.78 (4.33 - 219.04)	30.77 (28.75)	4.94 (3.27)	30.71 (5.95)
Romidepsin	Myelodysplastic Syndrome With Multilineage Dysplasia	1	152.09 (21.21 - 1090.6)	152.04 (148.58)	7.23 (5.54)	150.56 (28.96)

Romidepsin	Neuralgia	1	0.8 (0.11 - 5.67)	0.8 (0.05)	-0.33 (-1.99)	0.8 (0.15)
Romidepsin	Liver Injury	1	0.95 (0.13 - 6.76)	0.95 (0)	-0.07 (-1.74)	0.95 (0.18)
Romidepsin	Wound	1	0.71 (0.1 - 5.03)	0.71 (0.12)	-0.5 (-2.16)	0.71 (0.14)
Romidepsin	Injection Site Hypersensitivity	1	7.65 (1.08 - 54.35)	7.65 (5.78)	2.93 (1.27)	7.64 (1.48)
Romidepsin	Escherichia Bacteraemia	1	9.58 (1.35 - 68.05)	9.57 (7.67)	3.26 (1.59)	9.57 (1.85)
Romidepsin	Klebsiella Bacteraemia	1	24 (3.37 - 170.71)	23.99 (22)	4.58 (2.91)	23.96 (4.64)
Romidepsin	Subileus	1	9.3 (1.31 - 66.07)	9.3 (7.4)	3.22 (1.55)	9.29 (1.8)
Romidepsin	Abdominal Discomfort	1	0.11 (0.02 - 0.8)	0.11 (7.01)	-3.15 (-4.82)	0.11 (0.02)
Romidepsin	Tinea Infection	1	17.62 (2.48 - 125.24)	17.61 (15.65)	4.14 (2.47)	17.59 (3.41)
Romidepsin	Endocarditis Bacterial	1	26.35 (3.7 - 187.43)	26.34 (24.34)	4.72 (3.05)	26.3 (5.09)
Romidepsin	Neutrophilic Dermatosis	1	50.87 (7.14 - 362.4)	50.85 (48.71)	5.66 (3.99)	50.68 (9.8)
Romidepsin	Neoplasm Malignant	1	0.29 (0.04 - 2.03)	0.29 (1.79)	-1.81 (-3.47)	0.29 (0.06)
Romidepsin	Breast Cancer Metastatic	1	2.03 (0.29 - 14.45)	2.03 (0.53)	1.02 (-0.64)	2.03 (0.39)
Romidepsin	Aphasia	1	0.65 (0.09 - 4.58)	0.65 (0.19)	-0.63 (-2.3)	0.65 (0.13)
Romidepsin	Transplant Failure	1	8.25 (1.16 - 58.58)	8.24 (6.36)	3.04 (1.37)	8.24 (1.6)
Romidepsin	Staphylococcal Skin Infection	1	15.19 (2.14 - 108.01)	15.19 (13.24)	3.92 (2.25)	15.18 (2.94)
Romidepsin	Ear Haemorrhage	1	9.72 (1.37 - 69.04)	9.71 (7.81)	3.28 (1.61)	9.71 (1.88)
Romidepsin	Otitis Media Acute	1	25.86 (3.64 - 183.95)	25.85 (23.85)	4.69 (3.02)	25.81 (5)
Romidepsin	Pseudomonal Sepsis	1	12.29 (1.73 - 87.34)	12.29 (10.36)	3.62 (1.95)	12.28 (2.38)
Romidepsin	Gastric Haemorrhage	1	1.64 (0.23 - 11.66)	1.64 (0.25)	0.71 (-0.95)	1.64 (0.32)
Romidepsin	Ovarian Cancer	1	2.11 (0.3 - 14.97)	2.11 (0.58)	1.08 (-0.59)	2.11 (0.41)
Romidepsin	Premature Menopause	1	21.73 (3.06 - 154.51)	21.72 (19.74)	4.44 (2.77)	21.69 (4.2)
Romidepsin	Embolism	1	2.54 (0.36 - 18.07)	2.54 (0.94)	1.35 (-0.32)	2.54 (0.49)
Romidepsin	Skin Toxicity	1	3.9 (0.55 - 27.68)	3.9 (2.15)	1.96 (0.29)	3.89 (0.76)
Romidepsin	Viraemia	1	15.5 (2.18 - 110.19)	15.5 (13.55)	3.95 (2.28)	15.48 (3)
Romidepsin	Dermatitis Allergic	1	1.68 (0.24 - 11.95)	1.68 (0.28)	0.75 (-0.92)	1.68 (0.33)
Romidepsin	Cerebral Disorder	1	2.73 (0.38 - 19.37)	2.73 (1.09)	1.45 (-0.22)	2.73 (0.53)

Romidepsin	Left Ventricular Dysfunction	1	2.88 (0.41 - 20.48)	2.88 (1.23)	1.53 (-0.14)	2.88 (0.56)
Romidepsin	Radiation Skin Injury	1	17.22 (2.42 - 122.43)	17.22 (15.26)	4.1 (2.43)	17.2 (3.33)
Romidepsin	Nephrotic Syndrome	1	3.15 (0.44 - 22.39)	3.15 (1.47)	1.66 (-0.01)	3.15 (0.61)
Romidepsin	Clostridium Colitis	1	42.43 (5.96 - 302.17)	42.42 (40.33)	5.4 (3.73)	42.31 (8.19)
Romidepsin	Varicella Zoster Virus Infection	1	9.63 (1.36 - 68.43)	9.63 (7.73)	3.27 (1.6)	9.62 (1.87)
Romidepsin	Bronchospasm	1	1.48 (0.21 - 10.49)	1.48 (0.15)	0.56 (-1.1)	1.48 (0.29)
Romidepsin	Pericarditis	1	1.43 (0.2 - 10.14)	1.43 (0.13)	0.51 (-1.15)	1.43 (0.28)
Romidepsin	Pleuropericarditis	1	59.31 (8.32 - 422.79)	59.29 (57.09)	5.88 (4.21)	59.07 (11.42)
Romidepsin	Clostridium Difficile Infection	1	0.87 (0.12 - 6.2)	0.87 (0.02)	-0.19 (-1.86)	0.87 (0.17)
Romidepsin	Urinary Tract Infection Pseudomonal	1	35.39 (4.97 - 251.92)	35.38 (33.34)	5.14 (3.47)	35.3 (6.83)
Romidepsin	Chronic Myelomonocytic Leukaemia	1	29.6 (4.16 - 210.59)	29.59 (27.57)	4.88 (3.21)	29.53 (5.72)
Romidepsin	Aortic Thrombosis	1	19.15 (2.69 - 136.19)	19.15 (17.18)	4.26 (2.59)	19.13 (3.71)
Romidepsin	Portal Vein Thrombosis	1	6.36 (0.9 - 45.19)	6.36 (4.51)	2.67 (1)	6.36 (1.23)
Romidepsin	Alcohol Withdrawal Syndrome	1	25.69 (3.61 - 182.72)	25.68 (23.68)	4.68 (3.01)	25.64 (4.97)
Romidepsin	Gastrointestinal Pain	1	1.84 (0.26 - 13.06)	1.84 (0.38)	0.88 (-0.79)	1.84 (0.36)
Romidepsin	Injection Site Erythema	1	0.16 (0.02 - 1.14)	0.16 (4.38)	-2.63 (-4.3)	0.16 (0.03)
Romidepsin	Injection Site Pruritus	1	0.29 (0.04 - 2.09)	0.3 (1.68)	-1.76 (-3.43)	0.3 (0.06)
Romidepsin	Viral Load Increased	1	5.38 (0.76 - 38.21)	5.38 (3.56)	2.43 (0.76)	5.38 (1.04)
Romidepsin	Faecaloma	1	3.96 (0.56 - 28.1)	3.95 (2.21)	1.98 (0.32)	3.95 (0.77)
Romidepsin	Pulmonary Alveolar Haemorrhage	1	3.91 (0.55 - 27.76)	3.91 (2.16)	1.97 (0.3)	3.91 (0.76)
Romidepsin	Product Dose Omission Issue	1	0.04 (0.01 - 0.27)	0.04 (24.15)	-4.7 (-6.36)	0.04 (0.01)
Romidepsin	Laryngitis	1	1.85 (0.26 - 13.14)	1.85 (0.39)	0.89 (-0.78)	1.85 (0.36)
Romidepsin	Nasal Congestion	1	0.34 (0.05 - 2.42)	0.34 (1.28)	-1.55 (-3.22)	0.34 (0.07)
Romidepsin	Oropharyngeal Pain	1	0.2 (0.03 - 1.41)	0.2 (3.25)	-2.33 (-4)	0.2 (0.04)
Romidepsin	Rhinitis Allergic	1	4.49 (0.63 - 31.87)	4.48 (2.71)	2.16 (0.5)	4.48 (0.87)

Romidepsin	Motor Dysfunction	1	1.98 (0.28 - 14.07)	1.98 (0.49)	0.99 (-0.68)	1.98 (0.38)
Romidepsin	Polyneuropathy	1	1.74 (0.24 - 12.34)	1.74 (0.31)	0.8 (-0.87)	1.74 (0.34)
Romidepsin	Hiccups	1	2.66 (0.37 - 18.89)	2.66 (1.03)	1.41 (-0.26)	2.66 (0.52)
Romidepsin	Breast Cancer	1	0.19 (0.03 - 1.33)	0.19 (3.51)	-2.41 (-4.08)	0.19 (0.04)
Romidepsin	Dermatomyositis	1	8.49 (1.2 - 60.33)	8.49 (6.6)	3.08 (1.42)	8.48 (1.64)
Romidepsin	Face Oedema	1	1.25 (0.18 - 8.89)	1.25 (0.05)	0.32 (-1.34)	1.25 (0.24)
Romidepsin	Hypoxic-Ischaemic Encephalopathy	1	6.16 (0.87 - 43.74)	6.15 (4.32)	2.62 (0.95)	6.15 (1.19)
Romidepsin	Haematuria	1	0.57 (0.08 - 4.02)	0.57 (0.33)	-0.82 (-2.49)	0.57 (0.11)
Romidepsin	Body Temperature Increased	1	1 (0.14 - 7.07)	1 (0)	-0.01 (-1.67)	1 (0.19)
Romidepsin	Venoocclusive Liver Disease	1	4.36 (0.61 - 30.94)	4.35 (2.58)	2.12 (0.45)	4.35 (0.84)
Romidepsin	Hypertriglyceridaemia	1	3.74 (0.53 - 26.59)	3.74 (2.01)	1.9 (0.24)	3.74 (0.73)
Romidepsin	Bladder Cancer	1	0.27 (0.04 - 1.91)	0.27 (1.99)	-1.89 (-3.56)	0.27 (0.05)
Romidepsin	Bone Pain	1	0.33 (0.05 - 2.33)	0.33 (1.37)	-1.61 (-3.27)	0.33 (0.06)
Romidepsin	Throat Tightness	1	0.76 (0.11 - 5.42)	0.76 (0.07)	-0.39 (-2.06)	0.76 (0.15)
Romidepsin	Blood Calcium Decreased	1	1.77 (0.25 - 12.56)	1.77 (0.33)	0.82 (-0.84)	1.77 (0.34)
Romidepsin	Fibrin D Dimer Increased	1	5.94 (0.84 - 42.23)	5.94 (4.11)	2.57 (0.9)	5.94 (1.15)
Romidepsin	Oral Herpes	1	1.01 (0.14 - 7.19)	1.01 (0)	0.02 (-1.65)	1.01 (0.2)
Romidepsin	Bronchial Carcinoma	1	23.56 (3.31 - 167.57)	23.55 (21.56)	4.56 (2.89)	23.52 (4.56)
Romidepsin	Cardiac Death	1	15.95 (2.24 - 113.39)	15.95 (13.99)	3.99 (2.32)	15.93 (3.09)
Romidepsin	Glomerulonephritis Proliferative	1	86.3 (12.09 - 616.24)	86.27 (83.81)	6.42 (4.74)	85.8 (16.56)
Romidepsin	Glomerulonephritis Acute	1	97.84 (13.69 - 699.18)	97.81 (95.22)	6.6 (4.92)	97.2 (18.75)
Romidepsin	Vascular Device Infection	1	4.63 (0.65 - 32.93)	4.63 (2.85)	2.21 (0.54)	4.63 (0.9)
Romidepsin	Upper Limb Fracture	1	0.91 (0.13 - 6.48)	0.91 (0.01)	-0.13 (-1.8)	0.91 (0.18)
Romidepsin	Lower Gastrointestinal Haemorrhage	1	2.41 (0.34 - 17.14)	2.41 (0.83)	1.27 (-0.4)	2.41 (0.47)
Romidepsin	Overdose	1	0.08 (0.01 - 0.6)	0.08 (9.94)	-3.56 (-5.23)	0.08 (0.02)
Romidepsin	Intestinal Perforation	1	1.81 (0.25 - 12.85)	1.81 (0.36)	0.86 (-0.81)	1.81 (0.35)

Romidepsin	Blood Uric Acid Increased	1	3.6 (0.51 - 25.57)	3.6 (1.88)	1.85 (0.18)	3.6 (0.7)
Romidepsin	Colitis Ischaemic	1	3.18 (0.45 - 22.62)	3.18 (1.5)	1.67 (0)	3.18 (0.62)
Romidepsin	Vasogenic Cerebral Oedema	1	27.93 (3.93 - 198.7)	27.92 (25.91)	4.8 (3.13)	27.87 (5.4)
Romidepsin	Asphyxia	1	2.36 (0.33 - 16.8)	2.36 (0.79)	1.24 (-0.43)	2.36 (0.46)
Romidepsin	Aspiration	1	2.01 (0.28 - 14.3)	2.01 (0.51)	1.01 (-0.66)	2.01 (0.39)
Romidepsin	Enteropathy-Associated T-Cell Lymphoma	1	274.31 (37.96 - 1982.26)	274.22 (267.45)	8.07 (6.36)	269.43 (51.49)
Romidepsin	Bronchitis	1	0.25 (0.04 - 1.78)	0.25 (2.24)	-2 (-3.66)	0.25 (0.05)
Romidepsin	Dysphagia	1	0.2 (0.03 - 1.42)	0.2 (3.19)	-2.32 (-3.98)	0.2 (0.04)
Romidepsin	Mallory-Weiss Syndrome	1	16.66 (2.34 - 118.44)	16.66 (14.7)	4.06 (2.39)	16.64 (3.22)
Romidepsin	Coronary Artery Stenosis	1	5.06 (0.71 - 35.92)	5.05 (3.25)	2.34 (0.67)	5.05 (0.98)
Romidepsin	Interleukin-2 Receptor Increased	1	118.16 (16.51 - 845.49)	118.13 (115.25)	6.87 (5.19)	117.23 (22.59)
Romidepsin	Epstein-Barr Virus Associated Lymphoproliferative Disorder	1	11.65 (1.64 - 82.83)	11.65 (9.73)	3.54 (1.87)	11.64 (2.26)
Romidepsin	Epstein-Barr Virus Test Positive	1	33.91 (4.76 - 241.34)	33.9 (31.86)	5.08 (3.41)	33.83 (6.55)
Romidepsin	Colitis	1	0.55 (0.08 - 3.88)	0.55 (0.38)	-0.87 (-2.54)	0.55 (0.11)
Romidepsin	Atelectasis	1	2.18 (0.31 - 15.45)	2.17 (0.63)	1.12 (-0.55)	2.17 (0.42)
Romidepsin	Large Intestine Infection	1	11.85 (1.67 - 84.24)	11.85 (9.93)	3.57 (1.9)	11.84 (2.29)
Romidepsin	Tubulointerstitial Nephritis	1	1 (0.14 - 7.14)	1 (0)	0.01 (-1.66)	1 (0.19)
Romidepsin	Wolff-Parkinson-White Syndrome	1	44.27 (6.22 - 315.27)	44.25 (42.16)	5.46 (3.79)	44.13 (8.54)
Romidepsin	Transplantation Complication	1	48.92 (6.87 - 348.5)	48.91 (46.78)	5.61 (3.93)	48.75 (9.43)
Romidepsin	Pulmonary Hypertension	1	0.95 (0.13 - 6.72)	0.95 (0)	-0.08 (-1.75)	0.95 (0.18)
Romidepsin	B-Cell Lymphoma	1	5.74 (0.81 - 40.76)	5.74 (3.91)	2.52 (0.85)	5.73 (1.11)
Romidepsin	Hypoaesthesia Oral	1	1.32 (0.19 - 9.38)	1.32 (0.08)	0.4 (-1.27)	1.32 (0.26)
Romidepsin	Infusion Related Reaction	1	0.32 (0.05 - 2.28)	0.32 (1.44)	-1.64 (-3.31)	0.32 (0.06)
Romidepsin	Lung Adenocarcinoma	1	5.96 (0.84 - 42.36)	5.96 (4.13)	2.58 (0.91)	5.96 (1.16)

Romidepsin	Bk Virus Infection	1	6.64 (0.93 - 47.18)	6.64 (4.79)	2.73 (1.06)	6.64 (1.29)
Romidepsin	Epstein-Barr Viraemia	1	20.05 (2.82 - 142.6)	20.05 (18.07)	4.32 (2.65)	20.02 (3.88)
Romidepsin	Skin Disorder	1	0.59 (0.08 - 4.2)	0.59 (0.28)	-0.76 (-2.43)	0.59 (0.11)
Romidepsin	Abdominal Mass	1	7.95 (1.12 - 56.46)	7.94 (6.07)	2.99 (1.32)	7.94 (1.54)
Romidepsin	Hypozincaemia	1	415.17 (56.94 - 3026.96)	415.04 (402.17)	8.66 (6.93)	404.14 (76.67)
Romidepsin	Infective Aortitis	1	404.25 (55.48 - 2945.28)	404.12 (391.81)	8.62 (6.89)	393.78 (74.75)
Romidepsin	Sinus Arrest	1	14.25 (2 - 101.29)	14.25 (12.3)	3.83 (2.16)	14.23 (2.76)
Romidepsin	Cholecystitis Acute	1	3.38 (0.48 - 24.03)	3.38 (1.68)	1.76 (0.09)	3.38 (0.66)
Romidepsin	Microangiopathic Haemolytic Anaemia	1	25.86 (3.64 - 183.95)	25.85 (23.85)	4.69 (3.02)	25.81 (5)
Romidepsin	Plasmablastic Lymphoma	1	60.72 (8.52 - 432.85)	60.7 (58.48)	5.92 (4.24)	60.46 (11.69)
Romidepsin	Quadriparesis	1	12.61 (1.77 - 89.63)	12.61 (10.68)	3.66 (1.99)	12.6 (2.44)
Romidepsin	Retinitis	1	22.36 (3.14 - 159.02)	22.35 (20.37)	4.48 (2.81)	22.32 (4.32)
Romidepsin	Blood Ph Increased	1	61.94 (8.69 - 441.62)	61.92 (59.7)	5.95 (4.27)	61.68 (11.92)
Romidepsin	Protein Total Decreased	1	6 (0.84 - 42.64)	6 (4.17)	2.58 (0.92)	6 (1.16)
Romidepsin	Bronchopulmonary Aspergillosis	1	2.68 (0.38 - 19.05)	2.68 (1.05)	1.42 (-0.24)	2.68 (0.52)
Romidepsin	Enterococcal Bacteraemia	1	21.13 (2.97 - 150.25)	21.12 (19.14)	4.4 (2.73)	21.1 (4.09)
Romidepsin	Pancreatitis Acute	1	0.93 (0.13 - 6.61)	0.93 (0.01)	-0.1 (-1.77)	0.93 (0.18)
Romidepsin	Posterior Reversible Encephalopathy Syndrome	1	1.94 (0.27 - 13.81)	1.94 (0.46)	0.96 (-0.71)	1.94 (0.38)
Romidepsin	Systemic Candida	1	9.01 (1.27 - 64.02)	9.01 (7.11)	3.17 (1.5)	9 (1.74)
Romidepsin	Therapeutic Product Effect Decreased	1	0.19 (0.03 - 1.36)	0.19 (3.43)	-2.39 (-4.05)	0.19 (0.04)
Romidepsin	Pregnancy Of Partner	1	31.8 (4.47 - 226.32)	31.79 (29.76)	4.99 (3.32)	31.73 (6.14)
Romidepsin	Encephalitis Autoimmune	1	13.1 (1.84 - 93.08)	13.09 (11.16)	3.71 (2.04)	13.08 (2.54)

Romidepsin	Platelet Disorder	1	7.75 (1.09 - 55.09)	7.75 (5.88)	2.95 (1.29)	7.75 (1.5)
Romidepsin	Pathological Fracture	1	3.86 (0.54 - 27.42)	3.86 (2.12)	1.95 (0.28)	3.86 (0.75)
Romidepsin	Hypoacusis	1	0.38 (0.05 - 2.72)	0.38 (0.99)	-1.38 (-3.05)	0.38 (0.07)
Romidepsin	Eye Movement Disorder	1	2.77 (0.39 - 19.68)	2.77 (1.13)	1.47 (-0.2)	2.77 (0.54)
Romidepsin	Eyelid Ptosis	1	2.04 (0.29 - 14.48)	2.04 (0.53)	1.03 (-0.64)	2.04 (0.4)
Romidepsin	Blindness	1	0.5 (0.07 - 3.56)	0.5 (0.5)	-1 (-2.66)	0.5 (0.1)
Romidepsin	Intra-Abdominal Haemorrhage	1	8.39 (1.18 - 59.61)	8.39 (6.5)	3.07 (1.4)	8.38 (1.62)
Romidepsin	Idiosyncratic Drug Reaction	1	22.01 (3.09 - 156.51)	22 (20.02)	4.46 (2.79)	21.97 (4.26)
Romidepsin	Parkinsonism	1	2.18 (0.31 - 15.47)	2.18 (0.64)	1.12 (-0.54)	2.18 (0.42)
Romidepsin	Obstructive Airways Disorder	1	1.75 (0.25 - 12.4)	1.75 (0.32)	0.8 (-0.86)	1.75 (0.34)
Romidepsin	Mental Disorder	1	0.45 (0.06 - 3.17)	0.45 (0.69)	-1.16 (-2.83)	0.45 (0.09)
Romidepsin	Cell Death	1	9.86 (1.39 - 70.06)	9.86 (7.95)	3.3 (1.63)	9.85 (1.91)
Romidepsin	Cholestasis	1	1.13 (0.16 - 8.04)	1.13 (0.02)	0.18 (-1.49)	1.13 (0.22)
Romidepsin	Depressed Mood	1	0.37 (0.05 - 2.65)	0.37 (1.06)	-1.42 (-3.09)	0.37 (0.07)
Romidepsin	Cardiac Arrest	1	0.25 (0.04 - 1.77)	0.25 (2.26)	-2 (-3.67)	0.25 (0.05)
Romidepsin	Klebsiella Sepsis	1	18.29 (2.57 - 130.02)	18.28 (16.32)	4.19 (2.52)	18.26 (3.54)
Romidepsin	Neutrophil Count Abnormal	1	7.47 (1.05 - 53.08)	7.47 (5.6)	2.9 (1.23)	7.47 (1.45)
Romidepsin	Tumour Invasion	1	43.76 (6.15 - 311.66)	43.75 (41.66)	5.45 (3.77)	43.63 (8.44)
Romidepsin	Blood Lactic Acid Increased	1	4.13 (0.58 - 29.32)	4.13 (2.37)	2.04 (0.38)	4.13 (0.8)
Romidepsin	Pollakiuria	1	0.48 (0.07 - 3.41)	0.48 (0.56)	-1.06 (-2.73)	0.48 (0.09)
Romidepsin	Urine Odour Abnormal	1	3.28 (0.46 - 23.26)	3.27 (1.58)	1.71 (0.04)	3.27 (0.63)
Romidepsin	Heart Rate Increased	1	0.21 (0.03 - 1.49)	0.21 (2.98)	-2.25 (-3.92)	0.21 (0.04)
Romidepsin	Sinus Headache	1	3.19 (0.45 - 22.69)	3.19 (1.51)	1.68 (0.01)	3.19 (0.62)
Romidepsin	Renal Disorder	1	0.42 (0.06 - 2.99)	0.42 (0.8)	-1.25 (-2.92)	0.42 (0.08)
Romidepsin	Nervous System Disorder	1	0.62 (0.09 - 4.4)	0.62 (0.23)	-0.69 (-2.36)	0.62 (0.12)
Romidepsin	Otitis Externa	1	15.92 (2.24 - 113.16)	15.91 (13.96)	3.99 (2.32)	15.9 (3.08)
Romidepsin	Encephalitis	1	3.09 (0.43 - 21.93)	3.09 (1.41)	1.63 (-0.04)	3.09 (0.6)

Romidepsin	Herpes Zoster Reactivation	1	80.43 (11.27 - 574.08)	80.4 (78)	6.32 (4.64)	79.99 (15.45)
Romidepsin	Gastrointestinal Toxicity	1	4.63 (0.65 - 32.92)	4.63 (2.85)	2.21 (0.54)	4.63 (0.9)
Romidepsin	Hepatotoxicity	1	0.94 (0.13 - 6.67)	0.94 (0)	-0.09 (-1.76)	0.94 (0.18)
Romidepsin	Diabetic Hyperosmolar Coma	1	51.38 (7.21 - 366.04)	51.36 (49.21)	5.68 (4)	51.19 (9.9)
Romidepsin	Oesophagitis Haemorrhagic	1	66.79 (9.36 - 476.32)	66.77 (64.5)	6.05 (4.38)	66.48 (12.85)
Romidepsin	Heart Rate Decreased	1	0.54 (0.08 - 3.83)	0.54 (0.39)	-0.89 (-2.56)	0.54 (0.1)
Romidepsin	Traumatic Haematoma	1	17.82 (2.51 - 126.7)	17.81 (15.85)	4.15 (2.48)	17.8 (3.45)
Romidepsin	Abdominal Wall Wound	1	121.92 (17.03 - 872.54)	121.88 (118.94)	6.92 (5.23)	120.92 (23.3)
Romidepsin	Hypogeusia	1	9.73 (1.37 - 69.17)	9.73 (7.83)	3.28 (1.61)	9.73 (1.89)
Romidepsin	Hypovolaemia	1	3.86 (0.54 - 27.41)	3.86 (2.12)	1.95 (0.28)	3.86 (0.75)
Romidepsin	Blood Disorder	1	2.47 (0.35 - 17.56)	2.47 (0.88)	1.31 (-0.36)	2.47 (0.48)
Romidepsin	Haemangioma	1	9.83 (1.38 - 69.88)	9.83 (7.93)	3.3 (1.63)	9.83 (1.9)
Romidepsin	Diabetes Mellitus	1	0.26 (0.04 - 1.86)	0.26 (2.07)	-1.93 (-3.59)	0.26 (0.05)
Romidepsin	Metastases To Nervous System	1	144.92 (20.22 - 1038.68)	144.87 (141.54)	7.17 (5.48)	143.53 (27.62)
Romidepsin	Device Related Bacteraemia	1	77.19 (10.82 - 550.89)	77.17 (74.81)	6.26 (4.58)	76.79 (14.83)
Romidepsin	Cholelithiasis	1	0.61 (0.09 - 4.37)	0.62 (0.24)	-0.7 (-2.37)	0.62 (0.12)
Romidepsin	Orthopnoea	1	6.32 (0.89 - 44.91)	6.32 (4.48)	2.66 (0.99)	6.32 (1.22)
Romidepsin	Epstein Barr Virus Positive Mucocutaneous Ulcer	1	39.59 (5.56 - 281.87)	39.58 (37.51)	5.3 (3.63)	39.48 (7.64)
Romidepsin	Cerebral Infarction	1	0.83 (0.12 - 5.89)	0.83 (0.03)	-0.27 (-1.94)	0.83 (0.16)
Romidepsin	Monoparesis	1	10.98 (1.55 - 78.03)	10.98 (9.06)	3.46 (1.79)	10.97 (2.13)
Romidepsin	Hospitalisation	1	0.13 (0.02 - 0.94)	0.13 (5.72)	-2.92 (-4.59)	0.13 (0.03)
Romidepsin	Viral Haemorrhagic Cystitis	1	34.37 (4.83 - 244.58)	34.35 (32.31)	5.1 (3.43)	34.28 (6.64)
Romidepsin	Post Transplant Lymphoproliferative Disorder	1	4.11 (0.58 - 29.21)	4.11 (2.35)	2.04 (0.37)	4.11 (0.8)

Romidepsin	T-Cell Prolymphocytic Leukaemia	1	216.36 (30.05 - 1557.75)	216.29 (211.32)	7.74 (6.04)	213.3 (40.89)
Romidepsin	Thrombotic Microangiopathy	1	2.2 (0.31 - 15.66)	2.2 (0.66)	1.14 (-0.53)	2.2 (0.43)
Romidepsin	Cardiac Failure Chronic	1	4.5 (0.63 - 31.99)	4.5 (2.72)	2.17 (0.5)	4.5 (0.87)
Romidepsin	Accidental Exposure To Product	1	0.23 (0.03 - 1.6)	0.23 (2.66)	-2.15 (-3.82)	0.23 (0.04)
Romidepsin	Pneumonia Pseudomonal	1	10.39 (1.46 - 73.86)	10.39 (8.48)	3.38 (1.71)	10.38 (2.01)
Romidepsin	Oesophageal Candidiasis	1	5.4 (0.76 - 38.37)	5.4 (3.58)	2.43 (0.76)	5.4 (1.05)
Romidepsin	Graft Versus Host Disease In Liver	1	26.62 (3.74 - 189.38)	26.61 (24.61)	4.73 (3.06)	26.57 (5.15)
Romidepsin	Presyncope	1	0.79 (0.11 - 5.64)	0.79 (0.05)	-0.33 (-2)	0.79 (0.15)
Romidepsin	Feeling Cold	1	0.71 (0.1 - 5.02)	0.71 (0.12)	-0.5 (-2.17)	0.71 (0.14)
Romidepsin	Streptococcal Bacteraemia	1	20.24 (2.85 - 143.91)	20.23 (18.26)	4.34 (2.67)	20.21 (3.91)
Romidepsin	Therapy Interrupted	1	0.4 (0.06 - 2.82)	0.4 (0.91)	-1.33 (-3)	0.4 (0.08)
Romidepsin	Failure To Thrive	1	3.76 (0.53 - 26.69)	3.76 (2.02)	1.91 (0.24)	3.76 (0.73)
Romidepsin	Meningoencephalitis Herpetic	1	25.64 (3.61 - 182.42)	25.64 (23.64)	4.68 (3.01)	25.6 (4.96)
Romidepsin	Altered State Of Consciousness	1	0.92 (0.13 - 6.57)	0.92 (0.01)	-0.11 (-1.78)	0.92 (0.18)
Romidepsin	Anaphylactoid Reaction	1	5.68 (0.8 - 40.34)	5.68 (3.85)	2.5 (0.84)	5.68 (1.1)
Romidepsin	Therapeutic Product Effect Incomplete	1	0.17 (0.02 - 1.19)	0.17 (4.14)	-2.58 (-4.24)	0.17 (0.03)
Romidepsin	Therapy Change	1	2.17 (0.31 - 15.42)	2.17 (0.63)	1.12 (-0.55)	2.17 (0.42)
Romidepsin	Gastroenteritis Viral	1	1.11 (0.16 - 7.86)	1.11 (0.01)	0.15 (-1.52)	1.11 (0.21)
Romidepsin	Body Mass Index Decreased	1	46.13 (6.48 - 328.56)	46.12 (44.01)	5.52 (3.85)	45.98 (8.9)
Romidepsin	Hypothyroidism	1	0.64 (0.09 - 4.54)	0.64 (0.2)	-0.65 (-2.31)	0.64 (0.12)
Romidepsin	Urinary Incontinence	1	0.68 (0.1 - 4.83)	0.68 (0.15)	-0.55 (-2.22)	0.68 (0.13)
Romidepsin	Sleep Disorder	1	0.29 (0.04 - 2.07)	0.29 (1.71)	-1.77 (-3.44)	0.29 (0.06)
Romidepsin	Long Qt Syndrome	1	7.98 (1.12 - 56.67)	7.97 (6.1)	2.99 (1.33)	7.97 (1.54)
Romidepsin	Prescribed Overdose	1	1.09 (0.15 - 7.75)	1.09 (0.01)	0.13 (-1.54)	1.09 (0.21)

	Electrocardiogram T Wave					
Romidepsin	Abnormal	1	15.03 (2.11 - 106.84)	15.03 (13.08)	3.91 (2.24)	15.01 (2.91)
Romidepsin	Parotitis	1	18.55 (2.61 - 131.9)	18.55 (16.58)	4.21 (2.54)	18.53 (3.59)
Romidepsin	Erythema Multiforme	1	2.32 (0.33 - 16.49)	2.32 (0.75)	1.22 (-0.45)	2.32 (0.45)
Romidepsin	Rash Erythematous	1	0.48 (0.07 - 3.44)	0.48 (0.55)	-1.04 (-2.71)	0.48 (0.09)
Romidepsin	Troponin Increased	1	2.99 (0.42 - 21.25)	2.99 (1.32)	1.58 (-0.09)	2.99 (0.58)
Romidepsin	Parotid Gland Enlargement	1	32.96 (4.63 - 234.59)	32.95 (30.92)	5.04 (3.37)	32.89 (6.37)
Romidepsin	Granulocyte Count Decreased	1	17.07 (2.4 - 121.34)	17.06 (15.1)	4.09 (2.42)	17.04 (3.3)
Romidepsin	Abscess Limb	1	4.6 (0.65 - 32.67)	4.6 (2.81)	2.2 (0.53)	4.6 (0.89)
Romidepsin	Haemodynamic Instability	1	2.95 (0.42 - 20.94)	2.95 (1.29)	1.56 (-0.11)	2.95 (0.57)
Romidepsin	Lymphoproliferative Disorder	1	7.52 (1.06 - 53.42)	7.52 (5.65)	2.91 (1.24)	7.51 (1.46)
Romidepsin	Staphylococcal Abscess	1	33.11 (4.65 - 235.6)	33.1 (31.06)	5.05 (3.37)	33.03 (6.39)
Romidepsin	Anal Incontinence	1	1.5 (0.21 - 10.64)	1.5 (0.17)	0.58 (-1.08)	1.5 (0.29)
Romidepsin	Joint Swelling	1	0.16 (0.02 - 1.14)	0.16 (4.38)	-2.63 (-4.3)	0.16 (0.03)
Romidepsin	Vaginal Haemorrhage	1	0.44 (0.06 - 3.1)	0.44 (0.73)	-1.2 (-2.86)	0.44 (0.08)
Romidepsin	Generalised Oedema	1	1.77 (0.25 - 12.58)	1.77 (0.34)	0.82 (-0.84)	1.77 (0.34)
Romidepsin	Mediastinal Mass	1	59.54 (8.35 - 424.43)	59.52 (57.32)	5.89 (4.21)	59.3 (11.46)
Romidepsin	Acidosis	1	2.81 (0.4 - 19.93)	2.81 (1.16)	1.49 (-0.18)	2.81 (0.54)
Romidepsin	Atrioventricular Block First Degree	1	4.74 (0.67 - 33.65)	4.74 (2.95)	2.24 (0.58)	4.73 (0.92)
Romidepsin	Arrhythmia Supraventricular	1	20.11 (2.83 - 142.97)	20.1 (18.13)	4.33 (2.66)	20.08 (3.89)
Romidepsin	Granulocytopenia	1	3.58 (0.5 - 25.4)	3.57 (1.85)	1.84 (0.17)	3.57 (0.69)
Romidepsin	Insomnia	1	0.07 (0.01 - 0.52)	0.07 (11.65)	-3.76 (-5.42)	0.07 (0.01)
Romidepsin	Thrombosis	1	0.25 (0.04 - 1.78)	0.25 (2.24)	-1.99 (-3.66)	0.25 (0.05)
Romidepsin	Jugular Vein Thrombosis	1	11.48 (1.62 - 81.59)	11.48 (9.56)	3.52 (1.85)	11.47 (2.22)
Romidepsin	Gait Disturbance	1	0.1 (0.01 - 0.68)	0.1 (8.49)	-3.37 (-5.04)	0.1 (0.02)
Romidepsin	Prothrombin Time Prolonged	1	4.31 (0.61 - 30.59)	4.31 (2.54)	2.11 (0.44)	4.3 (0.83)
Romidepsin	Blood Cholesterol Increased	1	0.46 (0.06 - 3.24)	0.46 (0.65)	-1.13 (-2.8)	0.46 (0.09)

Romidepsin	Device Occlusion	1	1.54 (0.22 - 10.94)	1.54 (0.19)	0.62 (-1.04)	1.54 (0.3)
Romidepsin	Ventricular Arrhythmia	1	5.6 (0.79 - 39.75)	5.59 (3.77)	2.48 (0.82)	5.59 (1.08)
Romidepsin	Petechiae	1	1.98 (0.28 - 14.05)	1.98 (0.48)	0.98 (-0.68)	1.98 (0.38)
Romidepsin	Hypermagnesaemia	1	32.48 (4.56 - 231.11)	32.47 (30.43)	5.02 (3.35)	32.4 (6.27)
Romidepsin	Musculoskeletal Disorder	1	0.93 (0.13 - 6.59)	0.93 (0.01)	-0.11 (-1.77)	0.93 (0.18)
Romidepsin	Arthropathy	1	0.34 (0.05 - 2.42)	0.34 (1.27)	-1.55 (-3.22)	0.34 (0.07)
Romidepsin	Hypoglycaemia	1	0.43 (0.06 - 3.06)	0.43 (0.75)	-1.21 (-2.88)	0.43 (0.08)
Romidepsin	Tendon Disorder	1	3.05 (0.43 - 21.66)	3.05 (1.38)	1.61 (-0.06)	3.05 (0.59)
Romidepsin	Periarthritis	1	8.79 (1.24 - 62.48)	8.79 (6.9)	3.14 (1.47)	8.79 (1.7)
Romidepsin	Retching	1	0.97 (0.14 - 6.88)	0.97 (0)	-0.05 (-1.71)	0.97 (0.19)
Romidepsin	Lichenoid Keratosis	1	10.41 (1.47 - 74.01)	10.41 (8.5)	3.38 (1.71)	10.4 (2.02)
Romidepsin	Oral Dysaesthesia	1	97.84 (13.69 - 699.18)	97.81 (95.22)	6.6 (4.92)	97.2 (18.75)
Romidepsin	Immunosuppression	1	2.52 (0.35 - 17.87)	2.52 (0.91)	1.33 (-0.34)	2.52 (0.49)
Romidepsin	Intracardiac Thrombus	1	5.96 (0.84 - 42.35)	5.96 (4.13)	2.57 (0.91)	5.96 (1.15)
Romidepsin	Pulmonary Eosinophilia	1	44.79 (6.29 - 318.95)	44.77 (42.67)	5.48 (3.81)	44.64 (8.64)
Romidepsin	Night Sweats	1	0.64 (0.09 - 4.53)	0.64 (0.2)	-0.65 (-2.31)	0.64 (0.12)
Romidepsin	Product Contamination Microbial	1	16.99 (2.39 - 120.8)	16.99 (15.03)	4.08 (2.42)	16.97 (3.29)
Romidepsin	Adult T-Cell Lymphoma/Leukaemia Refractory	1	3072.28 (358.82 - 26305.54)	3071.29 (2557.74)	11.32 (9.33)	2559.58 (424.46)
Romidepsin	Infusion Site Vesicles	1	37.28 (5.24 - 265.41)	37.27 (35.21)	5.22 (3.54)	37.19 (7.2)
Romidepsin	Treatment Noncompliance	1	0.41 (0.06 - 2.91)	0.41 (0.85)	-1.29 (-2.95)	0.41 (0.08)
Romidepsin	Cerebral Toxoplasmosis	1	23.93 (3.36 - 170.18)	23.92 (21.93)	4.58 (2.91)	23.88 (4.63)
Romidepsin	Central Nervous System Lymphoma	1	16.31 (2.29 - 115.92)	16.3 (14.35)	4.03 (2.36)	16.29 (3.16)
Romidepsin	Vocal Cord Thickening	1	67.37 (9.45 - 480.52)	67.35 (65.08)	6.07 (4.39)	67.06 (12.96)
Romidepsin	Medication Error	1	0.46 (0.06 - 3.24)	0.46 (0.65)	-1.13 (-2.8)	0.46 (0.09)
Romidepsin	Eosinophilia	1	1.22 (0.17 - 8.69)	1.22 (0.04)	0.29 (-1.38)	1.22 (0.24)

Romidepsin	Procedural Complication	1	3.23 (0.45 - 22.92)	3.23 (1.54)	1.69 (0.02)	3.23 (0.63)
Romidepsin	Respiratory Arrest	1	0.76 (0.11 - 5.4)	0.76 (0.08)	-0.4 (-2.06)	0.76 (0.15)
Romidepsin	Incorrect Product Administration Duration	1	0.35 (0.05 - 2.52)	0.35 (1.18)	-1.5 (-3.16)	0.35 (0.07)
Romidepsin	Mucosal Haemorrhage	1	17.68 (2.49 - 125.67)	17.67 (15.71)	4.14 (2.47)	17.65 (3.42)
Romidepsin	Rectal Haemorrhage	1	0.46 (0.06 - 3.24)	0.46 (0.65)	-1.13 (-2.8)	0.46 (0.09)
Romidepsin	Chalazion	1	32.89 (4.62 - 234.09)	32.88 (30.85)	5.04 (3.36)	32.82 (6.35)
Romidepsin	Dry Eye	1	0.46 (0.06 - 3.27)	0.46 (0.63)	-1.12 (-2.79)	0.46 (0.09)
Romidepsin	Meibomianitis	1	232.75 (32.29 - 1677.5)	232.67 (227.24)	7.84 (6.14)	229.22 (43.9)
Romidepsin	Oesophageal Infection	1	43.15 (6.06 - 307.27)	43.14 (41.04)	5.43 (3.75)	43.02 (8.32)
Romidepsin	Sinusitis	1	0.19 (0.03 - 1.32)	0.19 (3.57)	-2.43 (-4.09)	0.19 (0.04)
Romidepsin	Feeling Drunk	1	2.66 (0.37 - 18.91)	2.66 (1.04)	1.41 (-0.26)	2.66 (0.52)
Romidepsin	Infusion Site Erythema	1	2.62 (0.37 - 18.59)	2.62 (1)	1.39 (-0.28)	2.62 (0.51)
Romidepsin	Infusion Site Pain	1	1.58 (0.22 - 11.24)	1.58 (0.21)	0.66 (-1.01)	1.58 (0.31)
Romidepsin	Infusion Site Warmth	1	17.6 (2.47 - 125.1)	17.59 (15.63)	4.14 (2.47)	17.57 (3.4)
Romidepsin	Rash Pruritic	1	0.38 (0.05 - 2.72)	0.38 (1)	-1.38 (-3.05)	0.38 (0.07)
Romidepsin	Anaemia Macrocytic	1	10.4 (1.46 - 73.91)	10.4 (8.49)	3.38 (1.71)	10.39 (2.01)
Romidepsin	Dermatitis Exfoliative	1	4.67 (0.66 - 33.17)	4.67 (2.88)	2.22 (0.55)	4.67 (0.9)
Romidepsin	Herpes Virus Infection	1	3.5 (0.49 - 24.83)	3.49 (1.78)	1.81 (0.14)	3.49 (0.68)
Romidepsin	Pelvi-Ureteric Obstruction	1	85.34 (11.95 - 609.36)	85.31 (82.87)	6.41 (4.73)	84.85 (16.38)
Romidepsin	Cutaneous T-Cell Lymphoma Refractory	1	1024.09 (135.23 - 7755.42)	1023.76 (957.9)	9.91 (8.1)	959.84 (176.4)
Romidepsin	Muscle Haemorrhage	1	5.13 (0.72 - 36.41)	5.12 (3.32)	2.36 (0.69)	5.12 (0.99)
Romidepsin	Metapneumovirus Infection	1	28.98 (4.07 - 206.21)	28.97 (26.96)	4.85 (3.18)	28.92 (5.6)
Romidepsin	Pneumonia Pneumococcal	1	14.66 (2.06 - 104.19)	14.65 (12.71)	3.87 (2.2)	14.64 (2.84)
Romidepsin	Tumour Flare	1	30.24 (4.25 - 215.16)	30.23 (28.21)	4.92 (3.24)	30.17 (5.84)

Romidepsin	Electrocardiogram Qt Interval Abnormal	1	44.79 (6.29 - 318.95)	44.77 (42.67)	5.48 (3.81)	44.64 (8.64)
Romidepsin	Performance Status Decreased	1	4.57 (0.64 - 32.47)	4.57 (2.79)	2.19 (0.52)	4.57 (0.89)
Romidepsin	Blood Creatinine Decreased	1	6.54 (0.92 - 46.48)	6.54 (4.69)	2.71 (1.04)	6.54 (1.27)
Romidepsin	Electrocardiogram Change	1	25.18 (3.54 - 179.12)	25.17 (23.18)	4.65 (2.98)	25.13 (4.87)
Romidepsin	Anaplastic Large Cell Lymphoma T- And Null-Cell Types Recurrent	1	1280.12 (166.4 - 9848.18)	1279.7 (1179.42)	10.21 (8.37)	1181.34 (214.26)
Romidepsin	Hip Fracture	1	0.61 (0.09 - 4.36)	0.61 (0.24)	-0.7 (-2.37)	0.61 (0.12)
Romidepsin	Underdose	1	0.25 (0.04 - 1.8)	0.25 (2.2)	-1.98 (-3.65)	0.25 (0.05)
Romidepsin	H1N1 Influenza	1	12.21 (1.72 - 86.78)	12.21 (10.28)	3.61 (1.94)	12.2 (2.36)
Romidepsin	Cognitive Disorder	1	0.41 (0.06 - 2.94)	0.41 (0.83)	-1.27 (-2.94)	0.41 (0.08)
Romidepsin	Lung Abscess	1	9.92 (1.4 - 70.51)	9.92 (8.02)	3.31 (1.64)	9.91 (1.92)
Romidepsin	Blood Sodium Decreased	1	1.16 (0.16 - 8.23)	1.16 (0.02)	0.21 (-1.45)	1.16 (0.22)
Romidepsin	Respiratory Tract Infection	1	0.78 (0.11 - 5.53)	0.78 (0.06)	-0.36 (-2.03)	0.78 (0.15)
Belinostat	Disease Progression	48	22.49 (16.85 - 30.03)	21.62 (945.15)	4.43 (2.77)	21.61 (16.97)
Belinostat	Nausea	38	2.64 (1.91 - 3.65)	2.59 (37.62)	1.37 (-0.29)	2.59 (1.98)
Belinostat	Death	31	1.83 (1.28 - 2.62)	1.81 (11.43)	0.86 (-0.81)	1.81 (1.34)
Belinostat	Platelet Count Decreased	27	13.46 (9.19 - 19.71)	13.17 (304.16)	3.72 (2.05)	13.17 (9.57)
Belinostat	Anaemia	27	7.9 (5.39 - 11.56)	7.74 (158.86)	2.95 (1.28)	7.74 (5.62)
Belinostat	Vomiting	25	3.03 (2.04 - 4.5)	2.99 (33.24)	1.58 (-0.09)	2.99 (2.14)
Belinostat	Fatigue	25	1.61 (1.08 - 2.39)	1.6 (5.63)	0.67 (-0.99)	1.6 (1.15)
Belinostat	Dyspnoea	23	2.17 (1.44 - 3.28)	2.15 (14.22)	1.1 (-0.57)	2.15 (1.52)
Belinostat	White Blood Cell Count Decreased	22	10.49 (6.88 - 16)	10.32 (185.34)	3.37 (1.7)	10.31 (7.25)
Belinostat	Constipation	19	4.68 (2.98 - 7.37)	4.62 (54.13)	2.21 (0.54)	4.62 (3.16)
Belinostat	Neutrophil Count Decreased	18	23.67 (14.86 - 37.71)	23.33 (384.61)	4.54 (2.87)	23.31 (15.79)
Belinostat	Hyponatraemia	17	17.15 (10.62 - 27.68)	16.92 (254.67)	4.08 (2.41)	16.91 (11.33)
Belinostat	Pain	17	1.34 (0.83 - 2.16)	1.33 (1.43)	0.42 (-1.25)	1.33 (0.89)

Belinostat	Peripheral T-Cell Lymphoma Unspecified Recurrent	15	14465.19 (7878.7 - 26557.89)	14281.79 (149937.99)	13.29 (11.57)	9997.55 (6013.19)
Belinostat	Hypocalcaemia	12	35.47 (20.08 - 62.66)	35.12 (397.49)	5.13 (3.46)	35.09 (21.8)
Belinostat	Lymphocyte Count Decreased	12	30.85 (17.46 - 54.49)	30.54 (342.73)	4.93 (3.26)	30.52 (18.96)
Belinostat	Headache	12	0.98 (0.56 - 1.74)	0.98 (0)	-0.02 (-1.69)	0.98 (0.61)
Belinostat	Sinus Tachycardia	11	49.42 (27.28 - 89.53)	48.97 (516.27)	5.61 (3.94)	48.9 (29.75)
Belinostat	Diarrhoea	11	0.86 (0.48 - 1.56)	0.86 (0.24)	-0.21 (-1.88)	0.86 (0.53)
Belinostat	Non-Cardiac Chest Pain	10	205.92 (110.3 - 384.46)	204.19 (2009.74)	7.67 (6)	202.95 (120.37)
Belinostat	Pneumonia	10	1.53 (0.82 - 2.86)	1.53 (1.85)	0.61 (-1.05)	1.53 (0.91)
Belinostat	Hypomagnesaemia	10	39.22 (21.04 - 73.11)	38.89 (368.84)	5.28 (3.61)	38.85 (23.07)
Belinostat	Pain In Extremity	10	1.73 (0.93 - 3.21)	1.72 (3.02)	0.78 (-0.89)	1.72 (1.02)
Belinostat	Infusion Related Reaction	10	8.22 (4.41 - 15.32)	8.16 (62.87)	3.03 (1.36)	8.16 (4.85)
Belinostat	Blood Bilirubin Increased	9	21.26 (11.03 - 40.97)	21.11 (172.35)	4.4 (2.73)	21.09 (12.18)
Belinostat	Pyrexia	9	1.42 (0.73 - 2.73)	1.41 (1.09)	0.5 (-1.17)	1.41 (0.82)
Belinostat	Hypertension	9	2.33 (1.21 - 4.49)	2.32 (6.78)	1.21 (-0.45)	2.32 (1.34)
Belinostat	Cough	9	1.62 (0.84 - 3.13)	1.62 (2.13)	0.69 (-0.97)	1.62 (0.93)
Belinostat	Dizziness	9	0.97 (0.5 - 1.87)	0.97 (0.01)	-0.04 (-1.71)	0.97 (0.56)
Belinostat	Electrocardiogram Qt Prolonged	9	13.22 (6.86 - 25.47)	13.12 (100.83)	3.71 (2.05)	13.12 (7.58)
Belinostat	Chills	9	4.18 (2.17 - 8.05)	4.15 (21.57)	2.05 (0.39)	4.15 (2.4)
Belinostat	Hypophosphataemia	8	57.47 (28.65 - 115.25)	57.09 (440.13)	5.83 (4.16)	56.99 (31.84)
Belinostat	Embolism	8	56.29 (28.07 - 112.88)	55.91 (430.78)	5.8 (4.13)	55.82 (31.18)
Belinostat	Hypoxia	8	12.95 (6.46 - 25.96)	12.87 (87.59)	3.69 (2.02)	12.87 (7.19)
Belinostat	Hypotension	8	2.15 (1.08 - 4.32)	2.15 (4.92)	1.1 (-0.57)	2.15 (1.2)
Belinostat	Hypokalaemia	8	9.9 (4.94 - 19.84)	9.84 (63.52)	3.3 (1.63)	9.83 (5.49)
Belinostat	Pruritus	8	1.12 (0.56 - 2.25)	1.12 (0.11)	0.16 (-1.5)	1.12 (0.63)
Belinostat	Sepsis	8	3.93 (1.96 - 7.87)	3.91 (17.33)	1.97 (0.3)	3.91 (2.18)

Belinostat	Malignant Neoplasm Progression	8	3.81 (1.9 - 7.64)	3.79 (16.48)	1.92 (0.26)	3.79 (2.12)
Belinostat	Decreased Appetite	8	1.77 (0.88 - 3.54)	1.76 (2.64)	0.82 (-0.85)	1.76 (0.98)
Belinostat	Off Label Use	7	0.37 (0.18 - 0.79)	0.38 (7.28)	-1.4 (-3.07)	0.38 (0.2)
Belinostat	Abdominal Pain	7	1.65 (0.79 - 3.48)	1.65 (1.8)	0.72 (-0.95)	1.65 (0.89)
Belinostat	Skin Lesion	7	13.44 (6.39 - 28.27)	13.37 (80.12)	3.74 (2.07)	13.37 (7.18)
Belinostat	Drug Ineffective	7	0.25 (0.12 - 0.52)	0.25 (15.79)	-1.98 (-3.65)	0.25 (0.14)
Belinostat	Fall	7	1.11 (0.53 - 2.33)	1.11 (0.07)	0.15 (-1.52)	1.11 (0.6)
Belinostat	Alanine Aminotransferase Increased	7	7.64 (3.64 - 16.07)	7.6 (40.17)	2.93 (1.26)	7.6 (4.08)
Belinostat	Insomnia	7	1.42 (0.67 - 2.98)	1.42 (0.86)	0.5 (-1.17)	1.42 (0.76)
Belinostat	Dyspepsia	6	3.46 (1.55 - 7.72)	3.45 (10.44)	1.79 (0.12)	3.45 (1.76)
Belinostat	Back Pain	6	1.32 (0.59 - 2.95)	1.32 (0.47)	0.4 (-1.27)	1.32 (0.67)
Belinostat	Covid-19	6	1.4 (0.63 - 3.12)	1.39 (0.67)	0.48 (-1.19)	1.39 (0.71)
Belinostat	Blood Lactate Dehydrogenase Increased	6	25.49 (11.43 - 56.87)	25.37 (140.36)	4.66 (3)	25.35 (12.95)
Belinostat	Blood Alkaline Phosphatase Increased	6	17.46 (7.83 - 38.95)	17.37 (92.57)	4.12 (2.45)	17.37 (8.87)
Belinostat	Anxiety	6	1.12 (0.5 - 2.51)	1.12 (0.08)	0.17 (-1.5)	1.12 (0.57)
Belinostat	Hyperglycaemia	6	9.73 (4.36 - 21.7)	9.68 (46.72)	3.27 (1.61)	9.68 (4.95)
Belinostat	Blood Creatinine Increased	6	5.39 (2.42 - 12.03)	5.37 (21.36)	2.42 (0.76)	5.37 (2.74)
Belinostat	Injection Site Reaction	6	4.94 (2.21 - 11.01)	4.92 (18.74)	2.3 (0.63)	4.92 (2.51)
Belinostat	Subcutaneous Abscess	5	54.82 (22.76 - 132.05)	54.59 (262.65)	5.77 (4.1)	54.51 (26.12)
Belinostat	Atrial Fibrillation	5	2.78 (1.15 - 6.69)	2.77 (5.67)	1.47 (-0.2)	2.77 (1.33)
Belinostat	Neutropenia	5	1.88 (0.78 - 4.52)	1.87 (2.04)	0.9 (-0.76)	1.87 (0.9)
Belinostat	Dehydration	5	2.14 (0.89 - 5.16)	2.14 (3.04)	1.1 (-0.57)	2.14 (1.03)
Belinostat	Supraventricular Tachycardia	5	32.73 (13.59 - 78.83)	32.6 (153.03)	5.03 (3.36)	32.57 (15.61)
Belinostat	Paraesthesia	5	1.71 (0.71 - 4.11)	1.71 (1.46)	0.77 (-0.9)	1.71 (0.82)

Belinostat	Infusion Site Pain	5	18.57 (7.71 - 44.71)	18.49 (82.72)	4.21 (2.54)	18.49 (8.86)
Belinostat	Gastrooesophageal Reflux Disease	5	3.54 (1.47 - 8.51)	3.53 (9.06)	1.82 (0.15)	3.53 (1.69)
Belinostat	Obesity	5	17.88 (7.43 - 43.05)	17.81 (79.31)	4.15 (2.49)	17.8 (8.53)
Belinostat	Dry Skin	5	1.8 (0.75 - 4.34)	1.8 (1.77)	0.85 (-0.82)	1.8 (0.86)
Belinostat	Febrile Neutropenia	5	4.09 (1.7 - 9.85)	4.08 (11.62)	2.03 (0.36)	4.08 (1.95)
Belinostat	Aspartate Aminotransferase Increased	5	6.62 (2.75 - 15.94)	6.6 (23.75)	2.72 (1.05)	6.6 (3.16)
Belinostat	Phlebitis	4	49.98 (18.71 - 133.48)	49.81 (191.06)	5.64 (3.97)	49.74 (21.86)
Belinostat	Flushing	4	2.56 (0.96 - 6.84)	2.56 (3.8)	1.35 (-0.31)	2.56 (1.12)
Belinostat	Peripheral Sensory Neuropathy	4	37.98 (14.22 - 101.43)	37.86 (143.39)	5.24 (3.57)	37.82 (16.62)
Belinostat	Rash	4	0.46 (0.17 - 1.22)	0.46 (2.56)	-1.12 (-2.79)	0.46 (0.2)
Belinostat	Hyperhidrosis	4	1.71 (0.64 - 4.55)	1.7 (1.17)	0.77 (-0.9)	1.7 (0.75)
Belinostat	Condition Aggravated	4	0.67 (0.25 - 1.79)	0.67 (0.65)	-0.58 (-2.24)	0.67 (0.3)
Belinostat	Pleural Effusion	4	3.71 (1.39 - 9.9)	3.7 (7.89)	1.89 (0.22)	3.7 (1.63)
Belinostat	Epistaxis	4	2.7 (1.01 - 7.19)	2.69 (4.25)	1.43 (-0.24)	2.69 (1.18)
Belinostat	Hypoalbuminaemia	4	31.06 (11.63 - 82.95)	30.96 (115.89)	4.95 (3.28)	30.94 (13.6)
Belinostat	Gait Disturbance	4	1.05 (0.39 - 2.79)	1.05 (0.01)	0.07 (-1.6)	1.05 (0.46)
Belinostat	Weight Decreased	4	0.74 (0.28 - 1.99)	0.75 (0.35)	-0.42 (-2.09)	0.75 (0.33)
Belinostat	Oedema Peripheral	4	2.36 (0.89 - 6.31)	2.36 (3.14)	1.24 (-0.43)	2.36 (1.04)
Belinostat	Dysphagia	3	1.72 (0.55 - 5.33)	1.71 (0.89)	0.78 (-0.89)	1.71 (0.66)
Belinostat	Infusion Site Extravasation	3	20.56 (6.62 - 63.85)	20.51 (55.64)	4.36 (2.69)	20.5 (7.94)
Belinostat	Injection Site Pain	3	0.56 (0.18 - 1.74)	0.56 (1.04)	-0.84 (-2.5)	0.56 (0.22)
Belinostat	Sinus Bradycardia	3	18.65 (6.01 - 57.94)	18.61 (49.97)	4.22 (2.55)	18.6 (7.21)
Belinostat	Hypercalcaemia	3	13.29 (4.28 - 41.28)	13.26 (34)	3.73 (2.06)	13.26 (5.14)
Belinostat	Hypersensitivity	3	0.82 (0.26 - 2.54)	0.82 (0.12)	-0.29 (-1.95)	0.82 (0.32)
Belinostat	Cellulitis	3	3.1 (1 - 9.63)	3.1 (4.26)	1.63 (-0.04)	3.1 (1.2)
Belinostat	Asthenia	3	0.43 (0.14 - 1.32)	0.43 (2.31)	-1.22 (-2.89)	0.43 (0.17)

Belinostat	Gastrointestinal Haemorrhage	3	1.75 (0.56 - 5.43)	1.75 (0.96)	0.8 (-0.86)	1.75 (0.68)
Belinostat	General Physical Health Deterioration	3	1.49 (0.48 - 4.63)	1.49 (0.48)	0.57 (-1.09)	1.49 (0.58)
Belinostat	Anal Incontinence	3	12.29 (3.96 - 38.18)	12.26 (31.03)	3.62 (1.95)	12.26 (4.75)
Belinostat	Lymphadenopathy	3	4.73 (1.52 - 14.7)	4.72 (8.81)	2.24 (0.57)	4.72 (1.83)
Belinostat	Proteinuria	3	8.69 (2.8 - 26.98)	8.67 (20.35)	3.12 (1.45)	8.66 (3.36)
Belinostat	Depression	3	0.75 (0.24 - 2.34)	0.75 (0.24)	-0.41 (-2.08)	0.75 (0.29)
Belinostat	Rash Maculo-Papular	3	7.87 (2.53 - 24.45)	7.85 (17.95)	2.97 (1.31)	7.85 (3.04)
Belinostat	Muscular Weakness	3	1.45 (0.47 - 4.51)	1.45 (0.42)	0.54 (-1.13)	1.45 (0.56)
Belinostat	Eczema	3	4.23 (1.36 - 13.14)	4.22 (7.38)	2.08 (0.41)	4.22 (1.64)
Belinostat	Tremor	3	1.01 (0.33 - 3.15)	1.01 (0)	0.02 (-1.65)	1.01 (0.39)
Belinostat	Pollakiuria	3	3.84 (1.24 - 11.93)	3.83 (6.29)	1.94 (0.27)	3.83 (1.49)
Belinostat	Oral Pain	3	6.82 (2.2 - 21.19)	6.81 (14.87)	2.77 (1.1)	6.81 (2.64)
Belinostat	Transient Ischaemic Attack	3	5.7 (1.83 - 17.69)	5.69 (11.59)	2.51 (0.84)	5.69 (2.2)
Belinostat	Disturbance In Attention	3	2.99 (0.96 - 9.29)	2.99 (3.97)	1.58 (-0.09)	2.99 (1.16)
Belinostat	Swelling Face	2	1.71 (0.43 - 6.85)	1.71 (0.59)	0.77 (-0.89)	1.71 (0.54)
Belinostat	Urinary Tract Infection	2	0.6 (0.15 - 2.4)	0.6 (0.53)	-0.73 (-2.4)	0.6 (0.19)
Belinostat	Seizure	2	0.69 (0.17 - 2.76)	0.69 (0.28)	-0.54 (-2.21)	0.69 (0.22)
Belinostat	Extravasation	2	28.5 (7.12 - 114.18)	28.46 (52.94)	4.83 (3.16)	28.43 (8.9)
Belinostat	Hepatic Enzyme Increased	2	1.63 (0.41 - 6.51)	1.62 (0.48)	0.7 (-0.97)	1.62 (0.51)
Belinostat	Blood Pressure Increased	2	0.68 (0.17 - 2.72)	0.68 (0.3)	-0.55 (-2.22)	0.68 (0.21)
Belinostat	Vision Blurred	2	0.82 (0.21 - 3.29)	0.82 (0.08)	-0.28 (-1.95)	0.82 (0.26)
Belinostat	Muscle Spasms	2	0.56 (0.14 - 2.25)	0.56 (0.68)	-0.83 (-2.5)	0.56 (0.18)
Belinostat	Acute Kidney Injury	2	0.51 (0.13 - 2.06)	0.51 (0.92)	-0.96 (-2.63)	0.51 (0.16)
Belinostat	Tachycardia	2	1.29 (0.32 - 5.17)	1.29 (0.13)	0.37 (-1.3)	1.29 (0.4)
Belinostat	Ascites	2	3.77 (0.94 - 15.09)	3.76 (4.06)	1.91 (0.24)	3.76 (1.18)
Belinostat	Renal Failure	2	0.79 (0.2 - 3.17)	0.79 (0.11)	-0.34 (-2)	0.79 (0.25)

Belinostat	Deep Vein Thrombosis	2	1.91 (0.48 - 7.64)	1.91 (0.86)	0.93 (-0.74)	1.91 (0.6)
Belinostat	Chest Discomfort	2	1.07 (0.27 - 4.3)	1.07 (0.01)	0.1 (-1.57)	1.07 (0.34)
Belinostat	Respiratory Failure	2	1.61 (0.4 - 6.43)	1.6 (0.46)	0.68 (-0.99)	1.6 (0.5)
Belinostat	Drug Intolerance	2	0.94 (0.23 - 3.74)	0.94 (0.01)	-0.1 (-1.76)	0.94 (0.29)
Belinostat	Device Related Infection	2	6.22 (1.55 - 24.89)	6.21 (8.74)	2.63 (0.97)	6.21 (1.94)
Belinostat	Circumstance Or Information Capable Of Leading To Medication Error	2	3.04 (0.76 - 12.18)	3.04 (2.74)	1.6 (-0.06)	3.04 (0.95)
Belinostat	Cardiac Failure	2	1.36 (0.34 - 5.44)	1.36 (0.19)	0.44 (-1.23)	1.36 (0.43)
Belinostat	Chest Pain	2	0.64 (0.16 - 2.55)	0.64 (0.41)	-0.65 (-2.32)	0.64 (0.2)
Belinostat	Pulmonary Embolism	2	1.3 (0.32 - 5.19)	1.3 (0.14)	0.38 (-1.29)	1.3 (0.41)
Belinostat	Restlessness	2	3.1 (0.77 - 12.39)	3.09 (2.83)	1.63 (-0.04)	3.09 (0.97)
Belinostat	Blood Urea Increased	2	9.16 (2.29 - 36.68)	9.15 (14.51)	3.19 (1.52)	9.14 (2.86)
Belinostat	Rhinitis Allergic	2	25.18 (6.29 - 100.86)	25.14 (46.33)	4.65 (2.98)	25.12 (7.87)
Belinostat	Haematuria	2	3.04 (0.76 - 12.19)	3.04 (2.74)	1.6 (-0.06)	3.04 (0.95)
Belinostat	Palpitations	2	0.95 (0.24 - 3.78)	0.95 (0.01)	-0.08 (-1.75)	0.95 (0.3)
Belinostat	Haemorrhoids	2	5.81 (1.45 - 23.26)	5.8 (7.95)	2.54 (0.87)	5.8 (1.82)
Belinostat	Stomatitis	2	1.68 (0.42 - 6.72)	1.68 (0.55)	0.75 (-0.92)	1.68 (0.53)
Belinostat	Gastrointestinal Disorder	2	0.83 (0.21 - 3.32)	0.83 (0.07)	-0.27 (-1.94)	0.83 (0.26)
Belinostat	Arthritis	2	1.26 (0.32 - 5.06)	1.26 (0.11)	0.34 (-1.33)	1.26 (0.4)
Belinostat	Dysgeusia	2	1.48 (0.37 - 5.93)	1.48 (0.31)	0.57 (-1.1)	1.48 (0.46)
Belinostat	Productive Cough	2	2.1 (0.52 - 8.4)	2.1 (1.15)	1.07 (-0.6)	2.1 (0.66)
Belinostat	Urinary Tract Pain	2	211.27 (52.54 - 849.46)	210.91 (415.21)	7.71 (6.04)	209.59 (65.42)
Belinostat	Infection	2	0.71 (0.18 - 2.84)	0.71 (0.24)	-0.49 (-2.16)	0.71 (0.22)
Belinostat	Urinary Incontinence	2	3.87 (0.97 - 15.49)	3.86 (4.25)	1.95 (0.28)	3.86 (1.21)

Belinostat	Infestation	2	379.32 (94.01 - 1530.48)	378.68 (744.91)	8.55 (6.87)	374.44 (116.54)
Belinostat	Wheezing	2	1.81 (0.45 - 7.24)	1.81 (0.72)	0.85 (-0.81)	1.81 (0.57)
Belinostat	Joint Range Of Motion Decreased	2	8.4 (2.1 - 33.62)	8.38 (13)	3.07 (1.4)	8.38 (2.63)
Belinostat	Mucosal Infection	2	414.66 (102.7 - 1674.29)	413.96 (813.83)	8.68 (6.99)	408.9 (127.19)
Belinostat	White Blood Cell Count Increased	2	3.03 (0.76 - 12.14)	3.03 (2.72)	1.6 (-0.07)	3.03 (0.95)
Belinostat	Acute Leukaemia	2	85.48 (21.31 - 342.8)	85.34 (166.28)	6.41 (4.74)	85.12 (26.63)
Belinostat	Thrombocytopenia	2	1.01 (0.25 - 4.04)	1.01 (0)	0.01 (-1.65)	1.01 (0.32)
Belinostat	Sinusitis	2	0.98 (0.25 - 3.93)	0.98 (0)	-0.03 (-1.69)	0.98 (0.31)
Belinostat	Hernia	2	5.28 (1.32 - 21.15)	5.27 (6.93)	2.4 (0.73)	5.27 (1.65)
Belinostat	Activated Partial Thromboplastin Time Prolonged	2	33.89 (8.46 - 135.75)	33.83 (63.66)	5.08 (3.41)	33.8 (10.58)
Belinostat	Blood Thyroid Stimulating Hormone Increased	2	10.79 (2.69 - 43.19)	10.77 (17.72)	3.43 (1.76)	10.77 (3.37)
Belinostat	Tumour Pain	2	67.43 (16.82 - 270.33)	67.32 (130.41)	6.07 (4.4)	67.19 (21.02)
Belinostat	Hot Flush	2	1.44 (0.36 - 5.75)	1.44 (0.27)	0.52 (-1.15)	1.44 (0.45)
Belinostat	Diabetes Mellitus	2	1.58 (0.39 - 6.32)	1.58 (0.42)	0.66 (-1.01)	1.58 (0.49)
Belinostat	Bone Marrow Transplant	2	69.47 (17.33 - 278.5)	69.35 (134.46)	6.11 (4.44)	69.21 (21.66)
Belinostat	Rectal Haemorrhage	2	2.5 (0.62 - 10.02)	2.5 (1.8)	1.32 (-0.35)	2.5 (0.78)
Belinostat	Myalgia	2	0.65 (0.16 - 2.62)	0.65 (0.37)	-0.61 (-2.28)	0.65 (0.2)
Belinostat	Memory Impairment	2	0.71 (0.18 - 2.85)	0.71 (0.23)	-0.49 (-2.16)	0.71 (0.22)
Belinostat	Ear Pain	2	5.42 (1.35 - 21.7)	5.41 (7.19)	2.44 (0.77)	5.41 (1.7)
Belinostat	Tracheal Inflammation	2	914.54 (224.19 - 3730.73)	912.99 (1773.4)	9.8 (8.09)	888.67 (274.05)
Belinostat	Presyncope	2	4.37 (1.09 - 17.48)	4.36 (5.18)	2.12 (0.46)	4.36 (1.37)
Belinostat	Skin Hyperpigmentation	2	11.32 (2.83 - 45.33)	11.3 (18.78)	3.5 (1.83)	11.3 (3.54)

Belinostat	Pancytopenia	2	2.11 (0.53 - 8.43)	2.1 (1.16)	1.07 (-0.6)	2.1 (0.66)
Belinostat	Hepatic Failure	2	4.08 (1.02 - 16.33)	4.07 (4.64)	2.03 (0.36)	4.07 (1.28)
Belinostat	Cytomegalovirus Infection	2	6.46 (1.61 - 25.87)	6.45 (9.21)	2.69 (1.02)	6.45 (2.02)
Belinostat	Breast Cancer Metastatic	2	12 (3 - 48.06)	11.98 (20.13)	3.58 (1.91)	11.98 (3.75)
Belinostat	Colitis	2	2.84 (0.71 - 11.37)	2.84 (2.38)	1.5 (-0.16)	2.84 (0.89)
Belinostat	Small Intestinal Obstruction	2	9.26 (2.31 - 37.08)	9.25 (14.71)	3.21 (1.54)	9.24 (2.9)
Belinostat	Renal Disorder	2	2.22 (0.55 - 8.88)	2.22 (1.33)	1.15 (-0.52)	2.22 (0.69)
Belinostat	Musculoskeletal Chest Pain	1	3.41 (0.48 - 24.2)	3.4 (1.7)	1.77 (0.1)	3.4 (0.66)
Belinostat	Oesophageal Infection	1	117.85 (16.53 - 840.27)	117.75 (115.35)	6.87 (5.2)	117.34 (22.68)
Belinostat	Oesophageal Spasm	1	33.25 (4.68 - 236.49)	33.22 (31.22)	5.05 (3.38)	33.19 (6.43)
Belinostat	Erythema	1	0.24 (0.03 - 1.68)	0.24 (2.46)	-2.08 (-3.74)	0.24 (0.05)
Belinostat	Skin Exfoliation	1	0.58 (0.08 - 4.14)	0.58 (0.3)	-0.78 (-2.45)	0.58 (0.11)
Belinostat	Infusion Site Phlebitis	1	362.52 (50.48 - 2603.32)	362.22 (356.35)	8.49 (6.79)	358.34 (68.85)
Belinostat	Infusion Site Erythema	1	6.06 (0.85 - 43.06)	6.05 (4.22)	2.6 (0.93)	6.05 (1.17)
Belinostat	Meningoencephalitis Viral	1	217.99 (30.48 - 1558.79)	217.81 (214.41)	7.76 (6.07)	216.4 (41.72)
Belinostat	Ejection Fraction Decreased	1	3.51 (0.49 - 24.95)	3.51 (1.79)	1.81 (0.14)	3.51 (0.68)
Belinostat	Enterocolitis Infectious	1	70.07 (9.84 - 498.87)	70.01 (67.88)	6.13 (4.45)	69.86 (13.52)
Belinostat	Hypoacusis	1	0.95 (0.13 - 6.73)	0.95 (0)	-0.08 (-1.75)	0.95 (0.18)
Belinostat	Hypermagnesaemia	1	82.76 (11.62 - 589.45)	82.69 (80.5)	6.37 (4.69)	82.49 (15.96)
Belinostat	Syncope	1	0.56 (0.08 - 3.99)	0.56 (0.34)	-0.83 (-2.5)	0.56 (0.11)
Belinostat	Electrocardiogram St Segment Depression	1	32.07 (4.51 - 228.07)	32.04 (30.05)	5 (3.33)	32.01 (6.2)
Belinostat	Ventricular Extrasystoles	1	6.65 (0.94 - 47.28)	6.65 (4.8)	2.73 (1.06)	6.65 (1.29)
Belinostat	Confusional State	1	0.34 (0.05 - 2.44)	0.34 (1.25)	-1.54 (-3.21)	0.34 (0.07)

Belinostat	Injection Site Irritation	1	8.12 (1.14 - 57.74)	8.12 (6.24)	3.02 (1.35)	8.12 (1.57)
Belinostat	Burning Sensation	1	0.77 (0.11 - 5.46)	0.77 (0.07)	-0.38 (-2.05)	0.77 (0.15)
Belinostat	Blister	1	0.95 (0.13 - 6.78)	0.95 (0)	-0.07 (-1.74)	0.95 (0.19)
Belinostat	Pancreatitis	1	1.25 (0.18 - 8.89)	1.25 (0.05)	0.32 (-1.35)	1.25 (0.24)
Belinostat	Bradycardia	1	1.04 (0.15 - 7.41)	1.04 (0)	0.06 (-1.61)	1.04 (0.2)
Belinostat	Drug Eruption	1	3.37 (0.47 - 23.96)	3.37 (1.67)	1.75 (0.08)	3.37 (0.65)
Belinostat	Conjunctivitis	1	2.87 (0.4 - 20.4)	2.87 (1.22)	1.52 (-0.15)	2.87 (0.56)
Belinostat	Hyperbilirubinaemia	1	5.35 (0.75 - 38.04)	5.35 (3.54)	2.42 (0.75)	5.35 (1.04)
Belinostat	Transaminases Increased	1	2.37 (0.33 - 16.82)	2.37 (0.79)	1.24 (-0.43)	2.37 (0.46)
Belinostat	Septic Shock	1	1.3 (0.18 - 9.21)	1.3 (0.07)	0.37 (-1.29)	1.3 (0.25)
Belinostat	Haematotoxicity	1	5.77 (0.81 - 41.01)	5.77 (3.94)	2.53 (0.86)	5.77 (1.12)
Belinostat	Injection Site Discomfort	1	5 (0.7 - 35.54)	5 (3.2)	2.32 (0.65)	5 (0.97)
Belinostat	Circulatory Collapse	1	3.59 (0.51 - 25.51)	3.59 (1.87)	1.84 (0.17)	3.59 (0.7)
Belinostat	Haemophagocytic Lymphohistiocytosis	1	5.43 (0.76 - 38.61)	5.43 (3.61)	2.44 (0.77)	5.43 (1.05)
Belinostat	Pseudomonas Infection	1	7.03 (0.99 - 49.97)	7.03 (5.17)	2.81 (1.14)	7.02 (1.36)
Belinostat	Pulmonary Sepsis	1	33.76 (4.75 - 240.09)	33.73 (31.73)	5.07 (3.4)	33.7 (6.53)
Belinostat	Peripheral Swelling	1	0.26 (0.04 - 1.86)	0.26 (2.07)	-1.93 (-3.6)	0.26 (0.05)
Belinostat	Physical Product Label Issue	1	43.54 (6.12 - 309.76)	43.5 (41.47)	5.44 (3.77)	43.45 (8.41)
Belinostat	Localised Infection	1	1.99 (0.28 - 14.17)	1.99 (0.49)	0.99 (-0.67)	1.99 (0.39)
Belinostat	Hydrocephalus	1	10.76 (1.51 - 76.51)	10.76 (8.85)	3.43 (1.76)	10.75 (2.08)
Belinostat	Mental Status Changes	1	2.32 (0.33 - 16.48)	2.32 (0.75)	1.21 (-0.46)	2.32 (0.45)
Belinostat	Gamma-Glutamyltransferase Increased	1	3.07 (0.43 - 21.82)	3.07 (1.4)	1.62 (-0.05)	3.07 (0.59)
Belinostat	Salmonella Bacteraemia	1	297.79 (41.55 - 2134.39)	297.54 (292.93)	8.2 (6.51)	294.91 (56.75)
Belinostat	Therapeutic Product Ineffective	1	12.61 (1.77 - 89.62)	12.6 (10.67)	3.65 (1.99)	12.59 (2.44)

Belinostat	Ischaemic Stroke	1	2.84 (0.4 - 20.22)	2.84 (1.2)	1.51 (-0.16)	2.84 (0.55)
Belinostat	Generalised Oedema	1	5.15 (0.73 - 36.63)	5.15 (3.34)	2.36 (0.7)	5.15 (1)
Belinostat	Systemic Candida	1	22.84 (3.21 - 162.42)	22.82 (20.86)	4.51 (2.84)	22.81 (4.42)
Belinostat	Multiple Organ Dysfunction Syndrome	1	1.37 (0.19 - 9.73)	1.37 (0.1)	0.45 (-1.22)	1.37 (0.27)
Belinostat	Musculoskeletal Stiffness	1	0.56 (0.08 - 3.97)	0.56 (0.35)	-0.84 (-2.51)	0.56 (0.11)
Belinostat	Musculoskeletal Discomfort	1	2.85 (0.4 - 20.25)	2.85 (1.2)	1.51 (-0.16)	2.85 (0.55)
Belinostat	Cardiac Disorder	1	0.61 (0.09 - 4.33)	0.61 (0.25)	-0.71 (-2.38)	0.61 (0.12)
Belinostat	Electrocardiogram Qt Interval	1	336.89 (46.95 - 2417.43)	336.61 (331.27)	8.38 (6.69)	333.25 (64.07)
Belinostat	Localised Oedema	1	11.6 (1.63 - 82.42)	11.59 (9.67)	3.53 (1.87)	11.58 (2.24)
Belinostat	Lacunar Stroke	1	140.73 (19.72 - 1004.03)	140.61 (138.03)	7.13 (5.45)	140.02 (27.05)
Belinostat	Acute Respiratory Failure	1	2.75 (0.39 - 19.57)	2.75 (1.12)	1.46 (-0.21)	2.75 (0.53)
Belinostat	Chronic Kidney Disease	1	0.46 (0.06 - 3.25)	0.46 (0.64)	-1.13 (-2.8)	0.46 (0.09)
Belinostat	Interstitial Lung Disease	1	1.15 (0.16 - 8.15)	1.15 (0.02)	0.2 (-1.47)	1.15 (0.22)
Belinostat	Ovarian Cancer Stage Iv	1	91.38 (12.83 - 650.99)	91.3 (89.07)	6.51 (4.83)	91.05 (17.61)
Belinostat	Pleurisy	1	9.89 (1.39 - 70.27)	9.88 (7.98)	3.3 (1.64)	9.88 (1.91)
Belinostat	Pleuritic Pain	1	25.5 (3.59 - 181.3)	25.48 (23.5)	4.67 (3)	25.46 (4.93)
Belinostat	Product Use In Unapproved Indication	1	0.19 (0.03 - 1.34)	0.19 (3.5)	-2.41 (-4.07)	0.19 (0.04)
Belinostat	Pulmonary Oedema	1	1.26 (0.18 - 8.95)	1.26 (0.05)	0.33 (-1.34)	1.26 (0.24)
Belinostat	Pulmonary Toxicity	1	7.86 (1.11 - 55.87)	7.86 (5.98)	2.97 (1.3)	7.85 (1.52)
Belinostat	Hypernatraemia	1	11.62 (1.64 - 82.62)	11.62 (9.7)	3.54 (1.87)	11.61 (2.25)
Belinostat	Nail Disorder	1	6.54 (0.92 - 46.47)	6.53 (4.69)	2.71 (1.04)	6.53 (1.27)
Belinostat	Gingival Pain	1	7.1 (1 - 50.46)	7.09 (5.24)	2.83 (1.16)	7.09 (1.37)
Belinostat	Lipase Increased	1	7.99 (1.12 - 56.75)	7.98 (6.1)	3 (1.33)	7.98 (1.55)

Belinostat	Dysphonia	1	0.88 (0.12 - 6.22)	0.88 (0.02)	-0.19 (-1.86)	0.88 (0.17)
Belinostat	Pain Of Skin	1	1.56 (0.22 - 11.1)	1.56 (0.2)	0.64 (-1.02)	1.56 (0.3)
Belinostat	Periorbital Oedema	1	11.45 (1.61 - 81.4)	11.44 (9.53)	3.52 (1.85)	11.44 (2.22)
Belinostat	Purpura	1	7.24 (1.02 - 51.48)	7.24 (5.38)	2.86 (1.19)	7.24 (1.4)
Belinostat	Oesophagitis	1	6.02 (0.85 - 42.77)	6.01 (4.18)	2.59 (0.92)	6.01 (1.17)
Belinostat	Bone Pain	1	0.87 (0.12 - 6.2)	0.87 (0.02)	-0.2 (-1.86)	0.87 (0.17)
Belinostat	Adrenal Insufficiency	1	4.52 (0.64 - 32.1)	4.51 (2.74)	2.17 (0.51)	4.51 (0.87)
Belinostat	Photosensitivity Reaction	1	3.17 (0.45 - 22.56)	3.17 (1.49)	1.67 (0)	3.17 (0.62)
Belinostat	Mouth Haemorrhage	1	7.61 (1.07 - 54.05)	7.6 (5.73)	2.93 (1.26)	7.6 (1.47)
Belinostat	Vaginal Haemorrhage	1	1.22 (0.17 - 8.64)	1.22 (0.04)	0.28 (-1.39)	1.22 (0.24)
Belinostat	Vulvovaginal Dryness	1	16.78 (2.36 - 119.32)	16.77 (14.82)	4.07 (2.4)	16.76 (3.25)
Belinostat	Pain In Jaw	1	1.79 (0.25 - 12.73)	1.79 (0.35)	0.84 (-0.83)	1.79 (0.35)
Belinostat	Dry Mouth	1	0.69 (0.1 - 4.89)	0.69 (0.14)	-0.54 (-2.21)	0.69 (0.13)
Belinostat	Face Oedema	1	3.67 (0.52 - 26.1)	3.67 (1.94)	1.88 (0.21)	3.67 (0.71)
Belinostat	Fungaemia	1	36.06 (5.07 - 256.46)	36.03 (34.02)	5.17 (3.5)	35.99 (6.97)
Belinostat	Retinopathy Of Prematurity	1	104.55 (14.67 - 745.15)	104.46 (102.15)	6.7 (5.03)	104.14 (20.14)
Belinostat	Scratch	1	5.41 (0.76 - 38.48)	5.41 (3.6)	2.44 (0.77)	5.41 (1.05)
Belinostat	Tinnitus	1	1.18 (0.17 - 8.4)	1.18 (0.03)	0.24 (-1.43)	1.18 (0.23)
Belinostat	Malaise	1	0.11 (0.02 - 0.78)	0.11 (7.2)	-3.18 (-4.84)	0.11 (0.02)
Belinostat	Atrial Flutter	1	7.07 (0.99 - 50.23)	7.06 (5.2)	2.82 (1.15)	7.06 (1.37)
Belinostat	Hypertriglyceridaemia	1	10.19 (1.43 - 72.42)	10.18 (8.28)	3.35 (1.68)	10.18 (1.97)
Belinostat	Thrombotic Microangiopathy	1	5.46 (0.77 - 38.82)	5.46 (3.64)	2.45 (0.78)	5.46 (1.06)
Belinostat	Shock	1	2.77 (0.39 - 19.65)	2.76 (1.13)	1.47 (-0.2)	2.76 (0.54)
Belinostat	Cyanosis	1	4.27 (0.6 - 30.34)	4.27 (2.5)	2.09 (0.42)	4.27 (0.83)
Belinostat	Abdominal Discomfort	1	0.29 (0.04 - 2.03)	0.29 (1.78)	-1.8 (-3.47)	0.29 (0.06)
Belinostat	Lip Swelling	1	1.62 (0.23 - 11.53)	1.62 (0.24)	0.7 (-0.97)	1.62 (0.31)

Belinostat	Mouth Swelling	1	7.01 (0.99 - 49.83)	7.01 (5.15)	2.81 (1.14)	7.01 (1.36)
Belinostat	Oropharyngeal Discomfort	1	5.78 (0.81 - 41.09)	5.78 (3.95)	2.53 (0.86)	5.78 (1.12)
Belinostat	Paraesthesia Oral	1	3.83 (0.54 - 27.19)	3.82 (2.09)	1.93 (0.27)	3.82 (0.74)
Belinostat	Incorrect Dose Administered	1	0.24 (0.03 - 1.71)	0.24 (2.39)	-2.05 (-3.72)	0.24 (0.05)
Belinostat	Medication Error	1	1.28 (0.18 - 9.12)	1.28 (0.06)	0.36 (-1.31)	1.28 (0.25)
Belinostat	Weight Increased	1	0.24 (0.03 - 1.7)	0.24 (2.42)	-2.06 (-3.73)	0.24 (0.05)
Belinostat	Leukocytosis	1	3.36 (0.47 - 23.89)	3.36 (1.66)	1.75 (0.08)	3.36 (0.65)
Belinostat	Nasal Inflammation	1	48.2 (6.77 - 342.93)	48.16 (46.11)	5.59 (3.92)	48.09 (9.31)
Belinostat	Neuralgia	1	2.05 (0.29 - 14.58)	2.05 (0.54)	1.04 (-0.63)	2.05 (0.4)
Belinostat	Clostridium Difficile Colitis	1	5.13 (0.72 - 36.46)	5.13 (3.32)	2.36 (0.69)	5.13 (0.99)
Belinostat	Jugular Vein Thrombosis	1	34.42 (4.84 - 244.8)	34.39 (32.39)	5.1 (3.43)	34.36 (6.65)
Belinostat	Hypoaesthesia	1	0.36 (0.05 - 2.57)	0.36 (1.13)	-1.47 (-3.13)	0.36 (0.07)
Belinostat	Emotional Disorder	1	1.7 (0.24 - 12.11)	1.7 (0.29)	0.77 (-0.9)	1.7 (0.33)
Belinostat	Peripheral Motor Neuropathy	1	40.67 (5.72 - 289.33)	40.64 (38.62)	5.34 (3.67)	40.59 (7.86)
Belinostat	Aspiration Pleural Cavity	1	60.53 (8.5 - 430.84)	60.48 (58.39)	5.92 (4.24)	60.37 (11.69)
Belinostat	Metastases To Pleura	1	51.31 (7.21 - 365.12)	51.27 (49.21)	5.68 (4.01)	51.19 (9.91)
Belinostat	Hepatic Pain	1	12.24 (1.72 - 87.02)	12.23 (10.31)	3.61 (1.94)	12.23 (2.37)
Belinostat	Insurance Issue	1	4.53 (0.64 - 32.21)	4.53 (2.75)	2.18 (0.51)	4.53 (0.88)
Belinostat	Gait Inability	1	0.94 (0.13 - 6.7)	0.94 (0)	-0.08 (-1.75)	0.94 (0.18)
Belinostat	Acute Myeloid Leukaemia	1	3.68 (0.52 - 26.18)	3.68 (1.95)	1.88 (0.21)	3.68 (0.71)
Belinostat	Embolism Venous	1	16.03 (2.25 - 113.92)	16.01 (14.07)	4 (2.33)	16.01 (3.1)
Belinostat	Coronavirus Infection	1	5.41 (0.76 - 38.45)	5.41 (3.59)	2.43 (0.77)	5.41 (1.05)
Belinostat	Urticaria	1	0.33 (0.05 - 2.36)	0.33 (1.35)	-1.59 (-3.26)	0.33 (0.06)
Belinostat	Angina Pectoris	1	2.11 (0.3 - 15)	2.11 (0.58)	1.08 (-0.59)	2.11 (0.41)
Belinostat	Malignant Melanoma	1	3.64 (0.51 - 25.84)	3.63 (1.91)	1.86 (0.19)	3.63 (0.7)
Belinostat	Candida Infection	1	2.68 (0.38 - 19.03)	2.68 (1.05)	1.42 (-0.25)	2.68 (0.52)
Belinostat	Dermatitis Contact	1	4.63 (0.65 - 32.91)	4.63 (2.84)	2.21 (0.54)	4.63 (0.9)

Belinostat	Thrombocytosis	1	14.22 (2 - 101.09)	14.21 (12.28)	3.83 (2.16)	14.21 (2.75)
Belinostat	Hyperkalaemia	1	1.65 (0.23 - 11.72)	1.65 (0.26)	0.72 (-0.95)	1.65 (0.32)
Belinostat	Vascular Access Complication	1	55.59 (7.81 - 395.6)	55.54 (53.47)	5.79 (4.12)	55.45 (10.73)
Belinostat	Cd4 Lymphocytes Decreased	1	28.95 (4.07 - 205.88)	28.93 (26.94)	4.85 (3.18)	28.9 (5.6)
Belinostat	Herpes Zoster	1	0.86 (0.12 - 6.14)	0.86 (0.02)	-0.21 (-1.88)	0.86 (0.17)
Belinostat	Herpes Virus Infection	1	9.69 (1.36 - 68.84)	9.68 (7.78)	3.27 (1.61)	9.68 (1.88)
Belinostat	Fungal Infection	1	1.51 (0.21 - 10.71)	1.51 (0.17)	0.59 (-1.08)	1.51 (0.29)
Belinostat	Neck Pain	1	0.94 (0.13 - 6.69)	0.94 (0)	-0.09 (-1.76)	0.94 (0.18)
Belinostat	Diverticulitis	1	1.85 (0.26 - 13.14)	1.85 (0.39)	0.89 (-0.78)	1.85 (0.36)
Belinostat	Sneezing	1	2.34 (0.33 - 16.63)	2.34 (0.77)	1.23 (-0.44)	2.34 (0.45)
Belinostat	Nocturia	1	4.52 (0.64 - 32.1)	4.51 (2.74)	2.17 (0.51)	4.51 (0.87)
Belinostat	Protein Total Decreased	1	17.74 (2.5 - 126.11)	17.73 (15.77)	4.15 (2.48)	17.72 (3.43)
Belinostat	Platelet Count Increased	1	4.01 (0.56 - 28.5)	4.01 (2.26)	2 (0.33)	4.01 (0.78)
Belinostat	Neutrophil Count Increased	1	5.06 (0.71 - 35.98)	5.06 (3.26)	2.34 (0.67)	5.06 (0.98)
Belinostat	Ovarian Cancer	1	5.36 (0.75 - 38.09)	5.36 (3.54)	2.42 (0.75)	5.36 (1.04)
Belinostat	Culture Positive	1	89.66 (12.59 - 638.7)	89.58 (87.36)	6.48 (4.81)	89.34 (17.28)
Belinostat	Phlebitis Infective	1	641.39 (88.59 - 4643.47)	640.85 (626.8)	9.3 (7.58)	628.78 (119.99)
Belinostat	Arthralgia	1	0.12 (0.02 - 0.86)	0.12 (6.37)	-3.04 (-4.7)	0.12 (0.02)
Belinostat	Administration Site Extravasation	1	46 (6.47 - 327.3)	45.96 (43.93)	5.52 (3.85)	45.9 (8.89)
Belinostat	Liver Function Test Abnormal	1	2.45 (0.34 - 17.41)	2.45 (0.86)	1.29 (-0.38)	2.45 (0.47)
Belinostat	Hospice Care	1	4.51 (0.63 - 32.01)	4.5 (2.72)	2.17 (0.5)	4.5 (0.87)
Belinostat	Full Blood Count Abnormal	1	1.6 (0.22 - 11.35)	1.6 (0.22)	0.67 (-0.99)	1.6 (0.31)
Belinostat	Infusion Site Swelling	1	7.91 (1.11 - 56.22)	7.9 (6.03)	2.98 (1.31)	7.9 (1.53)
Belinostat	Product Dose Omission Issue	1	0.09 (0.01 - 0.64)	0.09 (9.18)	-3.46 (-5.13)	0.09 (0.02)
Belinostat	Aspergillus Infection	1	6.84 (0.96 - 48.64)	6.84 (4.99)	2.77 (1.11)	6.84 (1.33)

Belinostat	Cytomegalovirus Infection Reactivation	1	14.49 (2.04 - 103.03)	14.48 (12.55)	3.86 (2.19)	14.48 (2.81)
Belinostat	Acquired Immunodeficiency Syndrome	1	145.01 (20.32 - 1034.72)	144.89 (142.28)	7.17 (5.49)	144.26 (27.87)
Belinostat	Ovarian Cancer Metastatic	1	89.42 (12.55 - 636.99)	89.34 (87.12)	6.48 (4.8)	89.1 (17.23)
Belinostat	Benign Neoplasm	1	47.31 (6.65 - 336.6)	47.27 (45.23)	5.56 (3.89)	47.2 (9.14)
Belinostat	Disorientation	1	1.54 (0.22 - 10.94)	1.54 (0.19)	0.62 (-1.05)	1.54 (0.3)
Belinostat	Vitreous Haemorrhage	1	20.71 (2.91 - 147.27)	20.7 (18.74)	4.37 (2.7)	20.69 (4.01)
Belinostat	Eye Pruritus	1	1.65 (0.23 - 11.7)	1.65 (0.25)	0.72 (-0.95)	1.65 (0.32)
Belinostat	Eye Disorder	1	1.61 (0.23 - 11.44)	1.61 (0.23)	0.69 (-0.98)	1.61 (0.31)
Belinostat	Vulvovaginal Pain	1	9.95 (1.4 - 70.71)	9.94 (8.04)	3.31 (1.64)	9.94 (1.93)
Belinostat	Neoplasm	1	4.42 (0.62 - 31.4)	4.42 (2.64)	2.14 (0.47)	4.42 (0.86)
Belinostat	Lymphocytic Leukaemia	1	134.48 (18.85 - 959.32)	134.37 (131.85)	7.06 (5.39)	133.84 (25.86)
Belinostat	Therapy Cessation	1	0.82 (0.12 - 5.86)	0.82 (0.04)	-0.28 (-1.95)	0.82 (0.16)
Belinostat	Transplant	1	21 (2.95 - 149.32)	20.98 (19.02)	4.39 (2.72)	20.97 (4.06)
Belinostat	Cardiogenic Shock	1	4.07 (0.57 - 28.89)	4.06 (2.31)	2.02 (0.35)	4.06 (0.79)
Belinostat	Hypovolaemia	1	11.6 (1.63 - 82.48)	11.6 (9.68)	3.53 (1.87)	11.59 (2.25)
Belinostat	Endotracheal Intubation	1	20.12 (2.83 - 143.01)	20.1 (18.14)	4.33 (2.66)	20.09 (3.89)
Belinostat	Scrotal Infection	1	260.56 (36.39 - 1865.56)	260.35 (256.35)	8.01 (6.33)	258.33 (49.76)
Belinostat	Penile Infection	1	297.79 (41.55 - 2134.39)	297.54 (292.93)	8.2 (6.51)	294.91 (56.75)
Belinostat	Erysipelas	1	10.26 (1.44 - 72.91)	10.25 (8.35)	3.36 (1.69)	10.25 (1.99)
Belinostat	Vessel Puncture Site Phlebitis	1	16676.18 (1511.05 - 184040.98)	16662.09 (11106.73)	13.44 (11.23)	11108.39 (1489.67)
Belinostat	Suicide Attempt	1	1 (0.14 - 7.12)	1 (0)	0 (-1.66)	1 (0.19)

Belinostat	Failure To Thrive	1	12.48 (1.76 - 88.72)	12.47 (10.55)	3.64 (1.97)	12.47 (2.42)
Belinostat	Intestinal Perforation	1	5.02 (0.71 - 35.67)	5.02 (3.22)	2.33 (0.66)	5.02 (0.97)
Belinostat	Tracheal Obstruction	1	223.84 (31.3 - 1600.91)	223.65 (220.18)	7.8 (6.11)	222.17 (42.83)
Belinostat	Obstructive Airways Disorder	1	4.25 (0.6 - 30.18)	4.24 (2.48)	2.09 (0.42)	4.24 (0.82)
Belinostat	Disease Recurrence	1	1.03 (0.14 - 7.31)	1.03 (0)	0.04 (-1.63)	1.03 (0.2)
Belinostat	Fluid Retention	1	0.95 (0.13 - 6.73)	0.95 (0)	-0.08 (-1.75)	0.95 (0.18)
Belinostat	Laboratory Test Abnormal	1	1.53 (0.22 - 10.89)	1.53 (0.19)	0.62 (-1.05)	1.53 (0.3)
Belinostat	Dyspnoea At Rest	1	15.92 (2.24 - 113.16)	15.91 (13.96)	3.99 (2.32)	15.9 (3.08)
Belinostat	Sars-Cov-2 Test Positive	1	2.8 (0.39 - 19.88)	2.8 (1.15)	1.48 (-0.18)	2.8 (0.54)
Belinostat	Oxygen Saturation Abnormal	1	17.64 (2.48 - 125.38)	17.62 (15.67)	4.14 (2.47)	17.61 (3.41)
Belinostat	Dyspnoea Exertional	1	1.35 (0.19 - 9.58)	1.35 (0.09)	0.43 (-1.24)	1.35 (0.26)
Belinostat	Leukopenia	1	1.12 (0.16 - 7.98)	1.12 (0.01)	0.17 (-1.5)	1.12 (0.22)
Belinostat	Liver Function Test Increased	1	2.09 (0.29 - 14.85)	2.09 (0.57)	1.06 (-0.61)	2.09 (0.4)
Belinostat	Product Use Complaint	1	2.99 (0.42 - 21.28)	2.99 (1.33)	1.58 (-0.09)	2.99 (0.58)
Belinostat	Anaphylactic Reaction	1	1.04 (0.15 - 7.38)	1.04 (0)	0.05 (-1.61)	1.04 (0.2)
Belinostat	Respiratory Tract Infection	1	1.98 (0.28 - 14.1)	1.98 (0.49)	0.99 (-0.68)	1.98 (0.38)
Belinostat	Loss Of Therapeutic Response	1	22.81 (3.21 - 162.19)	22.79 (20.82)	4.51 (2.84)	22.78 (4.41)
Belinostat	Treatment Failure	1	0.53 (0.07 - 3.76)	0.53 (0.42)	-0.92 (-2.59)	0.53 (0.1)
Belinostat	Flatulence	1	0.98 (0.14 - 6.98)	0.98 (0)	-0.03 (-1.69)	0.98 (0.19)
Belinostat	Abdominal Distension	1	0.52 (0.07 - 3.68)	0.52 (0.45)	-0.95 (-2.62)	0.52 (0.1)
Belinostat	Skin Infection	1	4.46 (0.63 - 31.69)	4.46 (2.68)	2.16 (0.49)	4.46 (0.86)
Panobinostat	Diarrhoea	279	6.37 (5.64 - 7.2)	6.02 (1180.63)	2.59 (0.92)	6.02 (5.44)
Panobinostat	Platelet Count Decreased	171	24.05 (20.63 - 28.03)	23.12 (3616.38)	4.53 (2.86)	23.07 (20.29)
Panobinostat	Death	147	2.44 (2.07 - 2.88)	2.39 (120.94)	1.26 (-0.41)	2.39 (2.09)
Panobinostat	Plasma Cell Myeloma	141	43.79 (37.01 - 51.82)	42.38 (5673.88)	5.4 (3.73)	42.18 (36.64)
Panobinostat	Malignant Neoplasm Progression	141	18.78 (15.88 - 22.22)	18.19 (2290.33)	4.18 (2.52)	18.16 (15.78)

Panobinostat	Nausea	107	2.04 (1.69 - 2.48)	2.02 (55.68)	1.01 (-0.65)	2.02 (1.72)
Panobinostat	Fatigue	96	1.7 (1.39 - 2.08)	1.68 (26.82)	0.75 (-0.92)	1.68 (1.42)
Panobinostat	Vomiting	76	2.56 (2.04 - 3.22)	2.54 (71.16)	1.34 (-0.32)	2.54 (2.1)
Panobinostat	Thrombocytopenia	76	10.85 (8.65 - 13.61)	10.67 (666.63)	3.41 (1.75)	10.66 (8.82)
Panobinostat	White Blood Cell Count Decreased	76	9.9 (7.89 - 12.43)	9.74 (596.81)	3.28 (1.62)	9.73 (8.05)
Panobinostat	Pneumonia	75	3.25 (2.58 - 4.08)	3.21 (114.56)	1.68 (0.01)	3.21 (2.65)
Panobinostat	Anaemia	71	5.86 (4.64 - 7.41)	5.78 (281.4)	2.53 (0.86)	5.78 (4.75)
Panobinostat	Pyrexia	70	3.1 (2.45 - 3.93)	3.07 (97.91)	1.62 (-0.05)	3.06 (2.52)
Panobinostat	Off Label Use	64	0.92 (0.72 - 1.18)	0.93 (0.39)	-0.11 (-1.78)	0.93 (0.75)
Panobinostat	Drug Ineffective	51	0.5 (0.38 - 0.66)	0.51 (25.11)	-0.98 (-2.65)	0.51 (0.4)
Panobinostat	Neutrophil Count Decreased	50	18.02 (13.63 - 23.82)	17.82 (792.78)	4.15 (2.49)	17.79 (14.08)
Panobinostat	Malaise	47	1.45 (1.09 - 1.94)	1.45 (6.58)	0.53 (-1.13)	1.45 (1.14)
Panobinostat	Disease Progression	42	5.32 (3.93 - 7.21)	5.28 (145.89)	2.4 (0.73)	5.28 (4.09)
Panobinostat	Hypokalaemia	40	13.91 (10.18 - 18.99)	13.79 (473.93)	3.78 (2.12)	13.77 (10.61)
Panobinostat	Febrile Neutropenia	38	8.6 (6.25 - 11.84)	8.53 (252.69)	3.09 (1.43)	8.52 (6.52)
Panobinostat	Haemoglobin Decreased	38	5.83 (4.24 - 8.03)	5.79 (150.76)	2.53 (0.87)	5.79 (4.43)
Panobinostat	Constipation	38	2.59 (1.88 - 3.56)	2.57 (36.62)	1.36 (-0.3)	2.57 (1.97)
Panobinostat	Decreased Appetite	37	2.25 (1.63 - 3.11)	2.24 (25.54)	1.16 (-0.5)	2.24 (1.71)
Panobinostat	Dehydration	36	4.42 (3.18 - 6.13)	4.39 (94.3)	2.13 (0.47)	4.39 (3.33)
Panobinostat	Asthenia	34	1.35 (0.97 - 1.9)	1.35 (3.11)	0.43 (-1.23)	1.35 (1.02)
Panobinostat	Neuropathy Peripheral	33	4.87 (3.46 - 6.86)	4.84 (100.67)	2.27 (0.61)	4.84 (3.63)
Panobinostat	Dyspnoea	30	0.78 (0.54 - 1.12)	0.78 (1.85)	-0.36 (-2.02)	0.78 (0.58)
Panobinostat	Hepatic Function Abnormal	28	12.41 (8.56 - 18.01)	12.34 (291.52)	3.62 (1.96)	12.32 (9.03)
Panobinostat	Renal Impairment	28	4.76 (3.28 - 6.9)	4.74 (82.57)	2.24 (0.58)	4.73 (3.47)
Panobinostat	Product Use In Unapproved Indication	27	1.33 (0.91 - 1.94)	1.32 (2.15)	0.4 (-1.26)	1.32 (0.96)
Panobinostat	Neutropenia	25	2.55 (1.72 - 3.77)	2.54 (23.35)	1.34 (-0.32)	2.54 (1.83)

Panobinostat	Bone Marrow Failure	25	17.01 (11.48 - 25.22)	16.92 (373.86)	4.08 (2.41)	16.89 (12.15)
Panobinostat	Concomitant Disease Aggravated	25	47.22 (31.83 - 70.03)	46.95 (1118.27)	5.55 (3.88)	46.7 (33.58)
Panobinostat	General Physical Health Deterioration	24	3.31 (2.22 - 4.95)	3.3 (38.47)	1.72 (0.05)	3.3 (2.36)
Panobinostat	Blood Creatinine Increased	24	6.03 (4.03 - 9)	6 (99.95)	2.58 (0.92)	5.99 (4.28)
Panobinostat	Dizziness	22	0.66 (0.43 - 1)	0.66 (3.91)	-0.6 (-2.27)	0.66 (0.46)
Panobinostat	Infection	22	2.17 (1.43 - 3.3)	2.16 (13.78)	1.11 (-0.55)	2.16 (1.52)
Panobinostat	Pancytopenia	21	6.25 (4.07 - 9.59)	6.22 (92.02)	2.64 (0.97)	6.22 (4.34)
Panobinostat	Sepsis	21	2.87 (1.87 - 4.41)	2.86 (25.48)	1.52 (-0.15)	2.86 (2)
Panobinostat	Hypophosphataemia	19	38.14 (24.28 - 59.92)	37.98 (681.19)	5.24 (3.57)	37.82 (25.92)
Panobinostat	Abdominal Pain	18	1.2 (0.75 - 1.9)	1.2 (0.59)	0.26 (-1.41)	1.2 (0.81)
Panobinostat	Cardiac Failure	18	3.41 (2.15 - 5.42)	3.4 (30.52)	1.77 (0.1)	3.4 (2.31)
Panobinostat	Acute Kidney Injury	18	1.28 (0.8 - 2.03)	1.28 (1.08)	0.35 (-1.31)	1.28 (0.87)
Panobinostat	Hypotension	18	1.35 (0.85 - 2.15)	1.35 (1.65)	0.43 (-1.23)	1.35 (0.92)
Panobinostat	Fall	17	0.75 (0.47 - 1.21)	0.75 (1.4)	-0.41 (-2.08)	0.75 (0.5)
Panobinostat	Atrial Fibrillation	17	2.65 (1.64 - 4.26)	2.64 (17.33)	1.4 (-0.27)	2.64 (1.77)
Panobinostat	Aspartate Aminotransferase Increased	16	5.95 (3.64 - 9.72)	5.93 (65.54)	2.57 (0.9)	5.92 (3.93)
Panobinostat	Confusional State	15	1.44 (0.87 - 2.4)	1.44 (2.05)	0.53 (-1.14)	1.44 (0.94)
Panobinostat	Syncope	15	2.38 (1.43 - 3.95)	2.37 (11.93)	1.25 (-0.42)	2.37 (1.55)
Panobinostat	Chest Pain	15	1.36 (0.82 - 2.25)	1.36 (1.41)	0.44 (-1.23)	1.36 (0.89)
Panobinostat	Renal Failure	15	1.64 (0.99 - 2.72)	1.64 (3.74)	0.71 (-0.95)	1.64 (1.07)
Panobinostat	Alanine Aminotransferase Increased	15	4.56 (2.75 - 7.58)	4.55 (41.59)	2.19 (0.52)	4.55 (2.98)
Panobinostat	Urinary Tract Infection	14	1.17 (0.69 - 1.98)	1.17 (0.35)	0.23 (-1.44)	1.17 (0.75)
Panobinostat	Abdominal Pain Upper	14	1.01 (0.6 - 1.71)	1.01 (0)	0.02 (-1.65)	1.01 (0.65)
Panobinostat	Cytopenia	14	14.59 (8.63 - 24.67)	14.55 (176.38)	3.86 (2.19)	14.53 (9.36)

Panobinostat	Cough	13	0.64 (0.37 - 1.11)	0.64 (2.56)	-0.63 (-2.3)	0.64 (0.41)
Panobinostat	Back Pain	13	0.8 (0.46 - 1.38)	0.8 (0.66)	-0.32 (-1.99)	0.8 (0.51)
Panobinostat	Tumour Lysis Syndrome	13	21.23 (12.31 - 36.61)	21.16 (249.18)	4.4 (2.73)	21.12 (13.38)
Panobinostat	Condition Aggravated	13	0.58 (0.34 - 1.01)	0.59 (3.84)	-0.77 (-2.44)	0.59 (0.37)
Panobinostat	Tachycardia	13	2.34 (1.36 - 4.04)	2.34 (9.97)	1.23 (-0.44)	2.34 (1.48)
Panobinostat	Weight Decreased	12	0.61 (0.35 - 1.08)	0.62 (2.89)	-0.7 (-2.37)	0.62 (0.38)
Panobinostat	Septic Shock	12	4.32 (2.45 - 7.61)	4.31 (30.49)	2.11 (0.44)	4.31 (2.68)
Panobinostat	Haemorrhage	12	1.71 (0.97 - 3.02)	1.71 (3.56)	0.78 (-0.89)	1.71 (1.07)
Panobinostat	Blood Lactate Dehydrogenase Increased	12	14.49 (8.22 - 25.54)	14.45 (150.01)	3.85 (2.18)	14.43 (8.98)
Panobinostat	Somnolence	11	0.82 (0.45 - 1.48)	0.82 (0.44)	-0.29 (-1.95)	0.82 (0.5)
Panobinostat	Epistaxis	11	2.05 (1.14 - 3.71)	2.05 (5.94)	1.04 (-0.63)	2.05 (1.25)
Panobinostat	Loss Of Consciousness	11	1.45 (0.8 - 2.62)	1.45 (1.52)	0.53 (-1.13)	1.45 (0.88)
Panobinostat	Blood Potassium Decreased	11	5.8 (3.21 - 10.48)	5.78 (43.52)	2.53 (0.86)	5.78 (3.52)
Panobinostat	Hyponatraemia	10	2.79 (1.5 - 5.19)	2.78 (11.44)	1.48 (-0.19)	2.78 (1.66)
Panobinostat	Rash	10	0.32 (0.17 - 0.59)	0.32 (14.78)	-1.66 (-3.32)	0.32 (0.19)
Panobinostat	Tremor	10	0.95 (0.51 - 1.76)	0.95 (0.03)	-0.08 (-1.74)	0.95 (0.56)
Panobinostat	Intestinal Obstruction	9	3.62 (1.88 - 6.97)	3.62 (17.06)	1.85 (0.19)	3.62 (2.09)
Panobinostat	Hyperkalaemia	9	4.17 (2.17 - 8.02)	4.16 (21.65)	2.06 (0.39)	4.16 (2.41)
Panobinostat	Electrocardiogram Qt Prolonged	9	3.63 (1.89 - 6.98)	3.62 (17.08)	1.86 (0.19)	3.62 (2.09)
Panobinostat	Gastrointestinal Haemorrhage	9	1.52 (0.79 - 2.92)	1.52 (1.59)	0.6 (-1.06)	1.52 (0.88)
Panobinostat	Pleural Effusion	9	2.38 (1.24 - 4.58)	2.38 (7.2)	1.25 (-0.42)	2.38 (1.38)
Panobinostat	Hypoxia	9	4.03 (2.09 - 7.74)	4.02 (20.41)	2.01 (0.34)	4.02 (2.32)
Panobinostat	Headache	9	0.2 (0.11 - 0.39)	0.2 (28.28)	-2.29 (-3.96)	0.2 (0.12)
Panobinostat	Pain In Extremity	9	0.43 (0.23 - 0.83)	0.43 (6.67)	-1.2 (-2.87)	0.43 (0.25)
Panobinostat	Pulmonary Embolism	9	1.76 (0.92 - 3.39)	1.76 (2.95)	0.81 (-0.85)	1.76 (1.02)

Panobinostat	Disseminated Intravascular Coagulation	9	12.33 (6.41 - 23.72)	12.3 (93.34)	3.62 (1.95)	12.29 (7.11)
Panobinostat	Hypertension	8	0.58 (0.29 - 1.15)	0.58 (2.48)	-0.79 (-2.46)	0.58 (0.32)
Panobinostat	Abdominal Discomfort	8	0.63 (0.31 - 1.25)	0.63 (1.77)	-0.67 (-2.34)	0.63 (0.35)
Panobinostat	Inappropriate Schedule Of Product Administration	8	0.4 (0.2 - 0.8)	0.4 (7.29)	-1.33 (-2.99)	0.4 (0.22)
Panobinostat	Orthostatic Hypotension	8	6.99 (3.49 - 13.99)	6.98 (40.94)	2.8 (1.13)	6.97 (3.9)
Panobinostat	Oedema Peripheral	8	1.33 (0.66 - 2.66)	1.33 (0.66)	0.41 (-1.26)	1.33 (0.74)
Panobinostat	Cerebral Haemorrhage	8	3.4 (1.7 - 6.79)	3.39 (13.49)	1.76 (0.09)	3.39 (1.9)
Panobinostat	Hospitalisation	8	0.65 (0.33 - 1.31)	0.65 (1.47)	-0.61 (-2.28)	0.65 (0.37)
Panobinostat	Disease Recurrence	7	1.92 (0.92 - 4.03)	1.92 (3.09)	0.94 (-0.73)	1.92 (1.03)
Panobinostat	Pneumonia Aspiration	7	4.26 (2.03 - 8.95)	4.26 (17.45)	2.09 (0.42)	4.26 (2.29)
Panobinostat	Dysphagia	7	1.14 (0.54 - 2.39)	1.14 (0.12)	0.19 (-1.48)	1.14 (0.61)
Panobinostat	Bacteraemia	7	9.05 (4.31 - 19.01)	9.04 (50.01)	3.17 (1.51)	9.03 (4.85)
Panobinostat	Peripheral Swelling	7	0.5 (0.24 - 1.05)	0.5 (3.52)	-1 (-2.67)	0.5 (0.27)
Panobinostat	Liver Disorder	7	2.56 (1.22 - 5.37)	2.56 (6.63)	1.35 (-0.31)	2.55 (1.37)
Panobinostat	Therapy Cessation	7	1.53 (0.73 - 3.21)	1.53 (1.27)	0.61 (-1.06)	1.53 (0.82)
Panobinostat	Interstitial Lung Disease	7	2.21 (1.05 - 4.63)	2.21 (4.61)	1.14 (-0.53)	2.21 (1.19)
Panobinostat	Lethargy	7	1.92 (0.91 - 4.03)	1.92 (3.08)	0.94 (-0.73)	1.92 (1.03)
Panobinostat	Ileus	7	9.98 (4.75 - 20.96)	9.97 (56.43)	3.32 (1.65)	9.96 (5.35)
Panobinostat	C-Reactive Protein Increased	7	2.84 (1.35 - 5.97)	2.84 (8.34)	1.51 (-0.16)	2.84 (1.53)
Panobinostat	Palpitations	7	0.92 (0.44 - 1.94)	0.92 (0.05)	-0.12 (-1.78)	0.92 (0.5)
Panobinostat	Chills	7	0.9 (0.43 - 1.89)	0.9 (0.08)	-0.15 (-1.82)	0.9 (0.48)
Panobinostat	Hypocalcaemia	7	5.81 (2.77 - 12.19)	5.8 (27.79)	2.54 (0.87)	5.8 (3.12)
Panobinostat	Depressed Level Of Consciousness	7	2.96 (1.41 - 6.21)	2.96 (9.06)	1.56 (-0.1)	2.96 (1.59)
Panobinostat	Lower Respiratory Tract Infection	7	2.12 (1.01 - 4.46)	2.12 (4.16)	1.09 (-0.58)	2.12 (1.14)
Panobinostat	Pain	7	0.15 (0.07 - 0.32)	0.15 (33.54)	-2.72 (-4.39)	0.15 (0.08)

Panobinostat	Hypercalcaemia	7	8.62 (4.1 - 18.09)	8.6 (47)	3.1 (1.44)	8.6 (4.62)
Panobinostat	Second Primary Malignancy	6	8.3 (3.73 - 18.49)	8.29 (38.43)	3.05 (1.38)	8.28 (4.24)
Panobinostat	Muscular Weakness	6	0.82 (0.37 - 1.82)	0.82 (0.24)	-0.29 (-1.96)	0.82 (0.42)
Panobinostat	Cardiac Arrest	6	1.3 (0.58 - 2.89)	1.3 (0.41)	0.38 (-1.29)	1.3 (0.66)
Panobinostat	Deep Vein Thrombosis	6	1.78 (0.8 - 3.97)	1.78 (2.06)	0.83 (-0.83)	1.78 (0.91)
Panobinostat	Haemoptysis	6	3.2 (1.44 - 7.14)	3.2 (9.08)	1.68 (0.01)	3.2 (1.64)
Panobinostat	Cerebrovascular Accident	6	0.63 (0.28 - 1.39)	0.63 (1.34)	-0.67 (-2.34)	0.63 (0.32)
Panobinostat	Abdominal Distension	6	0.87 (0.39 - 1.95)	0.87 (0.11)	-0.19 (-1.86)	0.87 (0.45)
Panobinostat	Plasmacytoma	6	45.81 (20.52 - 102.23)	45.74 (261.22)	5.51 (3.84)	45.51 (23.25)
Panobinostat	Blood Urea Increased	6	7.89 (3.54 - 17.58)	7.88 (36.02)	2.98 (1.31)	7.87 (4.03)
Panobinostat	Faeces Soft	6	10.22 (4.58 - 22.76)	10.2 (49.75)	3.35 (1.68)	10.19 (5.21)
Panobinostat	Hypophagia	6	3.51 (1.57 - 7.81)	3.5 (10.73)	1.81 (0.14)	3.5 (1.79)
Panobinostat	Gastrointestinal Disorder	6	0.69 (0.31 - 1.53)	0.69 (0.86)	-0.54 (-2.21)	0.69 (0.35)
Panobinostat	Electrolyte Imbalance	6	8.24 (3.7 - 18.35)	8.23 (38.07)	3.04 (1.37)	8.22 (4.21)
Panobinostat	Blood Pressure Decreased	6	1.42 (0.64 - 3.17)	1.42 (0.75)	0.51 (-1.16)	1.42 (0.73)
Panobinostat	Respiratory Tract Infection	6	3.28 (1.47 - 7.31)	3.28 (9.5)	1.71 (0.05)	3.28 (1.68)
Panobinostat	Hyperglycaemia	6	2.71 (1.22 - 6.04)	2.71 (6.47)	1.44 (-0.23)	2.71 (1.39)
Panobinostat	Drug Intolerance	6	0.76 (0.34 - 1.69)	0.76 (0.47)	-0.4 (-2.07)	0.76 (0.39)
Panobinostat	Haematemesis	6	3.83 (1.72 - 8.54)	3.83 (12.55)	1.94 (0.27)	3.83 (1.96)
Panobinostat	Full Blood Count Abnormal	6	2.58 (1.16 - 5.75)	2.58 (5.81)	1.37 (-0.3)	2.58 (1.32)
Panobinostat	Speech Disorder	6	1.8 (0.81 - 4.01)	1.8 (2.12)	0.85 (-0.82)	1.8 (0.92)
Panobinostat	Bronchitis	6	1.15 (0.52 - 2.57)	1.15 (0.12)	0.21 (-1.46)	1.15 (0.59)
Panobinostat	Melaena	6	4.15 (1.86 - 9.25)	4.15 (14.34)	2.05 (0.39)	4.15 (2.12)
Panobinostat	Lung Infiltration	5	12.93 (5.37 - 31.09)	12.91 (54.87)	3.69 (2.02)	12.89 (6.19)
Panobinostat	Stomatitis	5	1.15 (0.48 - 2.76)	1.15 (0.09)	0.2 (-1.47)	1.15 (0.55)
Panobinostat	Therapy Non-Responder	5	1.27 (0.53 - 3.05)	1.27 (0.28)	0.34 (-1.33)	1.27 (0.61)
Panobinostat	Plasma Cell Leukaemia	5	104.63 (43.3 - 252.84)	104.51 (506.47)	6.69 (5.02)	103.27 (49.36)

Panobinostat	Arthralgia	5	0.17 (0.07 - 0.4)	0.17 (20.69)	-2.57 (-4.24)	0.17 (0.08)
Panobinostat	Musculoskeletal Pain	5	1.39 (0.58 - 3.34)	1.39 (0.55)	0.47 (-1.19)	1.39 (0.67)
Panobinostat	Enteritis Infectious	5	38.67 (16.06 - 93.15)	38.63 (182.47)	5.27 (3.6)	38.46 (18.43)
Panobinostat	Eructation	5	4.65 (1.93 - 11.18)	4.64 (14.29)	2.21 (0.55)	4.64 (2.23)
Panobinostat	Pancreatitis	5	1.79 (0.74 - 4.3)	1.79 (1.74)	0.84 (-0.83)	1.79 (0.86)
Panobinostat	Supraventricular Tachycardia	5	9.37 (3.89 - 22.53)	9.36 (37.28)	3.22 (1.56)	9.35 (4.49)
Panobinostat	Oedema	5	1.48 (0.61 - 3.55)	1.48 (0.77)	0.56 (-1.1)	1.48 (0.71)
Panobinostat	Bone Lesion	5	23.35 (9.7 - 56.2)	23.32 (106.55)	4.54 (2.87)	23.26 (11.16)
Panobinostat	Altered State Of Consciousness	5	3.55 (1.48 - 8.54)	3.55 (9.15)	1.83 (0.16)	3.55 (1.7)
Panobinostat	Respiratory Distress	5	3.01 (1.25 - 7.24)	3.01 (6.71)	1.59 (-0.08)	3.01 (1.44)
Panobinostat	Pyelonephritis	5	8.86 (3.68 - 21.31)	8.85 (34.79)	3.14 (1.48)	8.84 (4.24)
Panobinostat	Candida Infection	5	3.7 (1.54 - 8.89)	3.7 (9.83)	1.89 (0.22)	3.69 (1.77)
Panobinostat	Pulmonary Hypertension	4	3.09 (1.16 - 8.24)	3.09 (5.65)	1.63 (-0.04)	3.09 (1.36)
Panobinostat	Lymphocyte Count Decreased	4	2.75 (1.03 - 7.33)	2.75 (4.45)	1.46 (-0.21)	2.75 (1.21)
Panobinostat	Clostridium Difficile Colitis	4	5.79 (2.17 - 15.45)	5.79 (15.84)	2.53 (0.87)	5.79 (2.55)
Panobinostat	Full Blood Count Decreased	4	2.44 (0.91 - 6.5)	2.44 (3.39)	1.29 (-0.38)	2.44 (1.07)
Panobinostat	Insomnia	4	0.22 (0.08 - 0.6)	0.22 (10.73)	-2.15 (-3.82)	0.22 (0.1)
Panobinostat	Cardiomyopathy	4	4.92 (1.84 - 13.11)	4.91 (12.46)	2.3 (0.63)	4.91 (2.16)
Panobinostat	Chest Discomfort	4	0.6 (0.22 - 1.59)	0.6 (1.08)	-0.74 (-2.41)	0.6 (0.26)
Panobinostat	Dyspnoea Exertional	4	1.48 (0.55 - 3.93)	1.47 (0.61)	0.56 (-1.11)	1.47 (0.65)
Panobinostat	Metabolic Acidosis	4	1.96 (0.73 - 5.22)	1.96 (1.87)	0.97 (-0.7)	1.96 (0.86)
Panobinostat	Gastritis	4	2.55 (0.96 - 6.79)	2.55 (3.75)	1.35 (-0.32)	2.55 (1.12)
Panobinostat	Ileus Paralytic	4	17.22 (6.45 - 45.94)	17.2 (60.93)	4.1 (2.43)	17.17 (7.55)
Panobinostat	Cardiac Disorder	4	0.69 (0.26 - 1.85)	0.69 (0.54)	-0.53 (-2.19)	0.69 (0.31)
Panobinostat	Electrocardiogram Abnormal	4	8.7 (3.26 - 23.2)	8.69 (27.2)	3.12 (1.45)	8.68 (3.82)
Panobinostat	Dysuria	4	1.66 (0.62 - 4.43)	1.66 (1.05)	0.73 (-0.94)	1.66 (0.73)
Panobinostat	Herpes Zoster	4	0.96 (0.36 - 2.55)	0.96 (0.01)	-0.06 (-1.73)	0.96 (0.42)

Panobinostat	Neutrophil Percentage Increased	4	59.12 (22.1 - 158.12)	59.07 (226.79)	5.87 (4.2)	58.67 (25.76)
Panobinostat	Klebsiella Infection	4	11.97 (4.49 - 31.93)	11.96 (40.12)	3.58 (1.91)	11.95 (5.26)
Panobinostat	Cardiac Failure Congestive	4	0.89 (0.33 - 2.36)	0.89 (0.06)	-0.17 (-1.84)	0.89 (0.39)
Panobinostat	Myocardial Infarction	4	0.46 (0.17 - 1.24)	0.46 (2.48)	-1.11 (-2.77)	0.46 (0.2)
Panobinostat	Feeling Abnormal	4	0.23 (0.09 - 0.62)	0.23 (10.22)	-2.11 (-3.77)	0.23 (0.1)
Panobinostat	Upper Gastrointestinal Haemorrhage	4	3.09 (1.16 - 8.24)	3.09 (5.65)	1.63 (-0.04)	3.09 (1.36)
Panobinostat	Enteritis	4	8.85 (3.32 - 23.6)	8.84 (27.79)	3.14 (1.48)	8.83 (3.89)
Panobinostat	Delirium	4	1.85 (0.69 - 4.93)	1.85 (1.56)	0.89 (-0.78)	1.85 (0.81)
Panobinostat	Hypoaesthesia	4	0.41 (0.15 - 1.09)	0.41 (3.43)	-1.29 (-2.96)	0.41 (0.18)
Panobinostat	Peripheral Sensory Neuropathy	4	10.42 (3.91 - 27.81)	10.42 (34.01)	3.38 (1.71)	10.4 (4.58)
Panobinostat	Pneumonia Bacterial	4	6.41 (2.4 - 17.1)	6.41 (18.24)	2.68 (1.01)	6.4 (2.82)
Panobinostat	Leukopenia	4	1.25 (0.47 - 3.34)	1.25 (0.2)	0.32 (-1.34)	1.25 (0.55)
Panobinostat	Pulmonary Oedema	4	1.43 (0.54 - 3.81)	1.43 (0.51)	0.51 (-1.15)	1.43 (0.63)
Panobinostat	Blood Phosphorus Decreased	4	15.99 (5.99 - 42.67)	15.98 (56.07)	4 (2.33)	15.95 (7.02)
Panobinostat	Azotaemia	4	19.59 (7.34 - 52.29)	19.58 (70.36)	4.29 (2.62)	19.54 (8.59)
Panobinostat	Renal Disorder	4	1.23 (0.46 - 3.28)	1.23 (0.17)	0.3 (-1.37)	1.23 (0.54)
Panobinostat	Product Use Issue	4	0.23 (0.09 - 0.62)	0.23 (10.22)	-2.11 (-3.78)	0.23 (0.1)
Panobinostat	Seizure	4	0.39 (0.15 - 1.04)	0.39 (3.83)	-1.36 (-3.02)	0.39 (0.17)
Panobinostat	Upper Respiratory Tract Inflammation	4	49.49 (18.51 - 132.3)	49.45 (188.79)	5.62 (3.95)	49.17 (21.6)
Panobinostat	Circulatory Collapse	4	4.1 (1.54 - 10.94)	4.1 (9.37)	2.03 (0.37)	4.1 (1.8)
Panobinostat	Glioma	4	109.98 (41 - 295)	109.88 (426.16)	6.76 (5.09)	108.52 (47.53)
Panobinostat	Muscle Spasms	4	0.31 (0.12 - 0.84)	0.32 (5.96)	-1.66 (-3.33)	0.32 (0.14)
Panobinostat	Multiple Organ Dysfunction Syndrome	4	1.54 (0.58 - 4.1)	1.54 (0.75)	0.62 (-1.05)	1.54 (0.68)

	Therapeutic Product Effect					
Panobinostat	Incomplete	4	0.4 (0.15 - 1.06)	0.4 (3.62)	-1.32 (-2.99)	0.4 (0.18)
Panobinostat	Cerebral Infarction	4	2.62 (0.98 - 6.98)	2.61 (3.99)	1.39 (-0.28)	2.61 (1.15)
Panobinostat	Concomitant Disease Progression	4	43.8 (16.39 - 117.04)	43.76 (166.29)	5.44 (3.77)	43.54 (19.13)
Panobinostat	Influenza	4	0.5 (0.19 - 1.32)	0.5 (2.05)	-1.01 (-2.68)	0.5 (0.22)
Panobinostat	Respiratory Failure	4	0.9 (0.34 - 2.41)	0.9 (0.04)	-0.15 (-1.81)	0.9 (0.4)
Panobinostat	Influenza Like Illness	4	0.77 (0.29 - 2.06)	0.77 (0.27)	-0.37 (-2.04)	0.77 (0.34)
Panobinostat	Contusion	4	0.6 (0.22 - 1.6)	0.6 (1.07)	-0.74 (-2.4)	0.6 (0.26)
Panobinostat	Gastroenteritis Viral	4	3.21 (1.21 - 8.57)	3.21 (6.09)	1.68 (0.02)	3.21 (1.41)
Panobinostat	Purpura	4	8.2 (3.07 - 21.86)	8.19 (25.23)	3.03 (1.37)	8.18 (3.6)
Panobinostat	Productive Cough	4	1.15 (0.43 - 3.05)	1.14 (0.07)	0.2 (-1.47)	1.14 (0.5)
Panobinostat	Large Intestine Perforation	4	9.22 (3.46 - 24.58)	9.21 (29.24)	3.2 (1.53)	9.2 (4.05)
Panobinostat	Subdural Haematoma	3	3.01 (0.97 - 9.34)	3.01 (4.02)	1.59 (-0.08)	3.01 (1.17)
Panobinostat	Clostridium Difficile Infection	3	1.7 (0.55 - 5.28)	1.7 (0.87)	0.77 (-0.9)	1.7 (0.66)
Panobinostat	Mental Status Changes	3	2.01 (0.65 - 6.24)	2.01 (1.53)	1.01 (-0.66)	2.01 (0.78)
Panobinostat	Dry Mouth	3	0.58 (0.19 - 1.8)	0.58 (0.91)	-0.78 (-2.45)	0.58 (0.23)
Panobinostat	Oesophagitis	3	5.13 (1.65 - 15.93)	5.13 (9.97)	2.36 (0.69)	5.13 (1.99)
Panobinostat	Toxicity To Various Agents	3	0.23 (0.07 - 0.71)	0.23 (7.75)	-2.12 (-3.79)	0.23 (0.09)
Panobinostat	Pneumonia Viral	3	12.95 (4.17 - 40.2)	12.94 (33.01)	3.69 (2.02)	12.92 (5.01)
Panobinostat	Subarachnoid Haemorrhage	3	4.29 (1.38 - 13.3)	4.28 (7.55)	2.1 (0.43)	4.28 (1.66)
Panobinostat	Gastrooesophageal Reflux Disease	3	0.59 (0.19 - 1.84)	0.59 (0.84)	-0.75 (-2.42)	0.59 (0.23)
Panobinostat	Enterocolitis	3	7.81 (2.52 - 24.24)	7.81 (17.79)	2.96 (1.3)	7.8 (3.02)
Panobinostat	Hypertriglyceridaemia	3	8.38 (2.7 - 26.02)	8.38 (19.48)	3.07 (1.4)	8.37 (3.25)
Panobinostat	Increased Tendency To Bruise	3	4.69 (1.51 - 14.55)	4.69 (8.7)	2.23 (0.56)	4.68 (1.82)
Panobinostat	Acute Myeloid Leukaemia	3	3.07 (0.99 - 9.52)	3.07 (4.18)	1.62 (-0.05)	3.07 (1.19)
Panobinostat	Blood Glucose Increased	3	0.23 (0.08 - 0.73)	0.24 (7.49)	-2.09 (-3.76)	0.24 (0.09)
Panobinostat	Rectal Haemorrhage	3	1.05 (0.34 - 3.26)	1.05 (0.01)	0.07 (-1.6)	1.05 (0.41)

Panobinostat	Cellulitis	3	0.87 (0.28 - 2.7)	0.87 (0.06)	-0.2 (-1.87)	0.87 (0.34)
Panobinostat	Erythema	3	0.2 (0.06 - 0.61)	0.2 (9.91)	-2.35 (-4.02)	0.2 (0.08)
Panobinostat	Cardiotoxicity	3	4.76 (1.54 - 14.78)	4.76 (8.91)	2.25 (0.58)	4.76 (1.85)
Panobinostat	Pruritus	3	0.12 (0.04 - 0.36)	0.12 (20.28)	-3.1 (-4.77)	0.12 (0.05)
Panobinostat	International Normalised Ratio Increased	3	2.31 (0.74 - 7.17)	2.31 (2.23)	1.21 (-0.46)	2.31 (0.9)
Panobinostat	Myalgia	3	0.27 (0.09 - 0.85)	0.27 (5.8)	-1.87 (-3.54)	0.27 (0.11)
Panobinostat	Joint Swelling	3	0.34 (0.11 - 1.05)	0.34 (3.89)	-1.56 (-3.23)	0.34 (0.13)
Panobinostat	Blood Magnesium Decreased	3	4.97 (1.6 - 15.41)	4.96 (9.49)	2.31 (0.64)	4.96 (1.92)
Panobinostat	Blood Calcium Decreased	3	4.01 (1.29 - 12.43)	4.01 (6.76)	2 (0.33)	4 (1.55)
Panobinostat	Blood Immunoglobulin G Increased	3	39.48 (12.69 - 122.78)	39.45 (111.92)	5.3 (3.63)	39.28 (15.2)
Panobinostat	Coagulopathy	3	3.17 (1.02 - 9.82)	3.17 (4.44)	1.66 (0)	3.16 (1.23)
Panobinostat	Graft Versus Host Disease	3	7.18 (2.31 - 22.27)	7.17 (15.92)	2.84 (1.17)	7.17 (2.78)
Panobinostat	Faeces Discoloured	3	2.12 (0.68 - 6.58)	2.12 (1.78)	1.08 (-0.58)	2.12 (0.82)
Panobinostat	Subileus	3	23.92 (7.7 - 74.31)	23.9 (65.66)	4.58 (2.91)	23.84 (9.23)
Panobinostat	Thrombosis	3	0.56 (0.18 - 1.75)	0.56 (1.02)	-0.83 (-2.5)	0.56 (0.22)
Panobinostat	Bone Pain	3	0.72 (0.23 - 2.24)	0.72 (0.32)	-0.47 (-2.13)	0.72 (0.28)
Panobinostat	N-Terminal Prohormone Brain Natriuretic Peptide Increased	3	27.29 (8.78 - 84.79)	27.27 (75.68)	4.76 (3.1)	27.19 (10.53)
Panobinostat	Incorrect Dose Administered	3	0.19 (0.06 - 0.6)	0.19 (10.01)	-2.36 (-4.02)	0.2 (0.08)
Panobinostat	Pneumonitis	3	1.58 (0.51 - 4.9)	1.58 (0.64)	0.66 (-1.01)	1.58 (0.61)
Panobinostat	Heart Rate Increased	3	0.47 (0.15 - 1.46)	0.47 (1.8)	-1.09 (-2.76)	0.47 (0.18)
Panobinostat	Unevaluable Event	3	0.53 (0.17 - 1.64)	0.53 (1.25)	-0.92 (-2.58)	0.53 (0.21)
Panobinostat	Abnormal Behaviour	3	1.4 (0.45 - 4.35)	1.4 (0.35)	0.49 (-1.18)	1.4 (0.54)
Panobinostat	Neoplasm Progression	3	0.96 (0.31 - 2.96)	0.96 (0.01)	-0.07 (-1.73)	0.96 (0.37)
Panobinostat	Therapeutic Response Decreased	3	0.77 (0.25 - 2.39)	0.77 (0.21)	-0.38 (-2.04)	0.77 (0.3)
Panobinostat	Drug Interaction	3	0.3 (0.1 - 0.92)	0.3 (5.05)	-1.76 (-3.42)	0.3 (0.11)

Panobinostat	Presyncope	3	1.85 (0.6 - 5.74)	1.85 (1.17)	0.89 (-0.78)	1.85 (0.72)
Panobinostat	Neutrophil Percentage Decreased	3	122.54 (39.19 - 383.13)	122.45 (356.34)	6.92 (5.24)	120.76 (46.52)
Panobinostat	Crepitations	3	14.95 (4.82 - 46.43)	14.95 (38.97)	3.9 (2.23)	14.92 (5.78)
Panobinostat	Brain Neoplasm	3	4.8 (1.55 - 14.88)	4.79 (9)	2.26 (0.59)	4.79 (1.86)
Panobinostat	Arrhythmia	3	1.04 (0.34 - 3.24)	1.04 (0.01)	0.06 (-1.6)	1.04 (0.4)
Panobinostat	Bacterial Sepsis	3	15.63 (5.03 - 48.53)	15.62 (40.98)	3.96 (2.29)	15.59 (6.04)
Panobinostat	Lymphadenopathy	3	1.32 (0.43 - 4.11)	1.32 (0.24)	0.4 (-1.26)	1.32 (0.51)
Panobinostat	Blood Calcium Increased	3	5.01 (1.61 - 15.54)	5 (9.61)	2.32 (0.66)	5 (1.94)
Panobinostat	Blood Magnesium Increased	3	53.02 (17.03 - 165.02)	52.98 (152.06)	5.72 (4.05)	52.66 (20.37)
Panobinostat	Disorientation	3	1.31 (0.42 - 4.06)	1.31 (0.22)	0.39 (-1.28)	1.31 (0.51)
Panobinostat	Lung Consolidation	3	19.74 (6.36 - 61.31)	19.73 (53.21)	4.3 (2.63)	19.68 (7.63)
Panobinostat	Upper Limb Fracture	3	2.12 (0.68 - 6.57)	2.12 (1.77)	1.08 (-0.58)	2.12 (0.82)
Panobinostat	Pneumonia Fungal	3	9.6 (3.09 - 29.79)	9.59 (23.07)	3.26 (1.59)	9.58 (3.71)
Panobinostat	Diabetes Mellitus	3	0.66 (0.21 - 2.06)	0.66 (0.51)	-0.59 (-2.26)	0.66 (0.26)
Panobinostat	Hyperuricaemia	3	12.24 (3.94 - 38)	12.23 (30.9)	3.61 (1.94)	12.22 (4.74)
Panobinostat	Inflammation	3	0.84 (0.27 - 2.6)	0.84 (0.09)	-0.25 (-1.92)	0.84 (0.33)
Panobinostat	Recurrent Cancer	3	9.75 (3.14 - 30.26)	9.74 (23.52)	3.28 (1.62)	9.73 (3.77)
Panobinostat	Hiccups	3	5.94 (1.91 - 18.44)	5.94 (12.31)	2.57 (0.9)	5.93 (2.3)
Panobinostat	Neck Pain	3	0.79 (0.25 - 2.45)	0.79 (0.17)	-0.34 (-2.01)	0.79 (0.31)
Panobinostat	Petechiae	3	4.55 (1.47 - 14.1)	4.54 (8.29)	2.18 (0.52)	4.54 (1.76)
Panobinostat	Autonomic Neuropathy	3	47.75 (15.35 - 148.58)	47.72 (136.47)	5.57 (3.9)	47.46 (18.36)
Panobinostat	Morganella Infection	3	149.3 (47.67 - 467.62)	149.19 (434.12)	7.2 (5.52)	146.68 (56.43)
Panobinostat	Hepatitis E	3	22.3 (7.18 - 69.27)	22.28 (60.83)	4.47 (2.81)	22.23 (8.61)
Panobinostat	Covid-19	3	0.18 (0.06 - 0.56)	0.18 (11.15)	-2.47 (-4.13)	0.18 (0.07)
Panobinostat	Neutropenic Sepsis	2	4.25 (1.06 - 16.99)	4.24 (4.96)	2.08 (0.42)	4.24 (1.33)
Panobinostat	Thrombocytosis	2	7.91 (1.98 - 31.64)	7.9 (12.05)	2.98 (1.31)	7.9 (2.47)

Panobinostat	Myelodysplastic Syndrome	2	2.31 (0.58 - 9.25)	2.31 (1.49)	1.21 (-0.46)	2.31 (0.72)
Panobinostat	Encephalopathy	2	1.28 (0.32 - 5.13)	1.28 (0.12)	0.36 (-1.31)	1.28 (0.4)
Panobinostat	Parainfluenzae Virus Infection	2	15.99 (3.99 - 64.03)	15.98 (28.03)	4 (2.33)	15.95 (5)
Panobinostat	Cognitive Disorder	2	0.62 (0.16 - 2.49)	0.62 (0.46)	-0.68 (-2.35)	0.62 (0.2)
Panobinostat	Cardio-Respiratory Arrest	2	0.85 (0.21 - 3.41)	0.85 (0.05)	-0.23 (-1.9)	0.85 (0.27)
Panobinostat	Gastric Ulcer	2	1.7 (0.43 - 6.82)	1.7 (0.58)	0.77 (-0.9)	1.7 (0.53)
Panobinostat	Myocardial Ischaemia	2	3.76 (0.94 - 15.05)	3.76 (4.05)	1.91 (0.24)	3.76 (1.18)
Panobinostat	Bundle Branch Block Left	2	8.84 (2.21 - 35.39)	8.84 (13.89)	3.14 (1.47)	8.83 (2.77)
Panobinostat	Hip Fracture	2	0.95 (0.24 - 3.81)	0.95 (0)	-0.07 (-1.74)	0.95 (0.3)
Panobinostat	Metastases To Bone	2	1.77 (0.44 - 7.09)	1.77 (0.67)	0.83 (-0.84)	1.77 (0.56)
Panobinostat	Paraesthesia	2	0.19 (0.05 - 0.76)	0.19 (6.89)	-2.39 (-4.06)	0.19 (0.06)
Panobinostat	Urosepsis	2	3.26 (0.81 - 13.04)	3.26 (3.13)	1.7 (0.04)	3.26 (1.02)
Panobinostat	Lower Limb Fracture	2	1.62 (0.4 - 6.48)	1.62 (0.47)	0.69 (-0.97)	1.62 (0.51)
Panobinostat	Acute Myocardial Infarction	2	1.15 (0.29 - 4.6)	1.15 (0.04)	0.2 (-1.47)	1.15 (0.36)
Panobinostat	Hypothyroidism	2	0.99 (0.25 - 3.96)	0.99 (0)	-0.02 (-1.68)	0.99 (0.31)
Panobinostat	Myocardial Necrosis Marker Increased	2	15.77 (3.94 - 63.15)	15.76 (27.6)	3.98 (2.31)	15.73 (4.93)
Panobinostat	Hypertensive Crisis	2	2.61 (0.65 - 10.44)	2.61 (1.99)	1.38 (-0.28)	2.61 (0.82)
Panobinostat	Heart Rate Decreased	2	0.76 (0.19 - 3.04)	0.76 (0.15)	-0.4 (-2.06)	0.76 (0.24)
Panobinostat	Sedation	2	1.3 (0.32 - 5.19)	1.3 (0.14)	0.37 (-1.29)	1.3 (0.41)
Panobinostat	Anxiety	2	0.1 (0.03 - 0.42)	0.1 (15.44)	-3.26 (-4.93)	0.1 (0.03)
Panobinostat	Pharyngitis	2	2.27 (0.57 - 9.09)	2.27 (1.42)	1.18 (-0.48)	2.27 (0.71)
Panobinostat	Red Blood Cell Count Decreased	2	0.99 (0.25 - 3.97)	0.99 (0)	-0.01 (-1.68)	0.99 (0.31)
Panobinostat	Tachyarrhythmia	2	13.42 (3.35 - 53.74)	13.42 (22.95)	3.74 (2.08)	13.4 (4.2)
Panobinostat	Cardiac Failure Acute	2	4.39 (1.1 - 17.57)	4.39 (5.23)	2.13 (0.47)	4.39 (1.38)
Panobinostat	Tumour Rupture	2	51.07 (12.72 - 205.13)	51.05 (97.57)	5.67 (3.99)	50.76 (15.86)
Panobinostat	Thirst	2	1.64 (0.41 - 6.56)	1.64 (0.5)	0.71 (-0.95)	1.64 (0.51)

Panobinostat	Pneumonia Pseudomonal	2	15.25 (3.81 - 61.09)	15.25 (26.58)	3.93 (2.26)	15.22 (4.77)
Panobinostat	Blood Fibrinogen Increased	2	60.12 (14.96 - 241.62)	60.09 (115.42)	5.9 (4.22)	59.68 (18.64)
Panobinostat	Electrocardiogram St Segment Depression	2	18.68 (4.66 - 74.82)	18.67 (33.37)	4.22 (2.55)	18.63 (5.83)
Panobinostat	Hypersomnia	2	1.02 (0.26 - 4.09)	1.02 (0)	0.03 (-1.63)	1.02 (0.32)
Panobinostat	Ammonia Increased	2	6.16 (1.54 - 24.64)	6.15 (8.63)	2.62 (0.95)	6.15 (1.93)
Panobinostat	Gait Disturbance	2	0.15 (0.04 - 0.58)	0.15 (9.97)	-2.77 (-4.44)	0.15 (0.05)
Panobinostat	Oropharyngeal Pain	2	0.29 (0.07 - 1.18)	0.3 (3.37)	-1.76 (-3.43)	0.3 (0.09)
Panobinostat	Sinus Disorder	2	1.36 (0.34 - 5.45)	1.36 (0.19)	0.45 (-1.22)	1.36 (0.43)
Panobinostat	Escherichia Sepsis	2	11.06 (2.76 - 44.29)	11.06 (18.28)	3.47 (1.8)	11.05 (3.46)
Panobinostat	Haematoma	2	1.15 (0.29 - 4.6)	1.15 (0.04)	0.2 (-1.47)	1.15 (0.36)
Panobinostat	Adverse Drug Reaction	2	0.29 (0.07 - 1.17)	0.29 (3.42)	-1.77 (-3.44)	0.29 (0.09)
Panobinostat	Urinary Tract Infection Viral	2	270.54 (66.2 - 1105.61)	270.41 (520.56)	8.03 (6.33)	262.25 (80.75)
Panobinostat	Decreased Activity	2	2.55 (0.64 - 10.19)	2.55 (1.88)	1.35 (-0.32)	2.55 (0.8)
Panobinostat	Intestinal Dilatation	2	28.57 (7.13 - 114.54)	28.56 (53.01)	4.83 (3.16)	28.47 (8.91)
Panobinostat	Staphylococcal Infection	2	1.03 (0.26 - 4.1)	1.03 (0)	0.04 (-1.63)	1.03 (0.32)
Panobinostat	Hyperthermia	2	4.18 (1.04 - 16.72)	4.18 (4.83)	2.06 (0.39)	4.18 (1.31)
Panobinostat	Drug Ineffective For Unapproved Indication	2	0.45 (0.11 - 1.8)	0.45 (1.34)	-1.15 (-2.81)	0.45 (0.14)
Panobinostat	Asthma	2	0.27 (0.07 - 1.07)	0.27 (4.01)	-1.9 (-3.57)	0.27 (0.08)
Panobinostat	Upper Respiratory Tract Infection	2	0.62 (0.16 - 2.48)	0.62 (0.47)	-0.69 (-2.36)	0.62 (0.19)
Panobinostat	Atrial Flutter	2	4 (1 - 16.01)	4 (4.5)	2 (0.33)	4 (1.25)
Panobinostat	Stress Cardiomyopathy	2	5.04 (1.26 - 20.17)	5.04 (6.47)	2.33 (0.67)	5.04 (1.58)
Panobinostat	Immunoglobulins Increased	2	73.68 (18.31 - 296.44)	73.64 (142.11)	6.19 (4.51)	73.03 (22.78)
Panobinostat	Left Ventricular Dysfunction	2	4.72 (1.18 - 18.88)	4.72 (5.86)	2.24 (0.57)	4.71 (1.48)
Panobinostat	Fibrin D Dimer Increased	2	9.09 (2.27 - 36.38)	9.08 (14.38)	3.18 (1.51)	9.08 (2.84)

Panobinostat	Fibrin Degradation Products Increased	2	174.89 (43.12 - 709.35)	174.81 (338.79)	7.42 (5.73)	171.37 (53.1)
Panobinostat	Acute Pulmonary Oedema	2	6.67 (1.67 - 26.7)	6.67 (9.63)	2.74 (1.07)	6.66 (2.09)
Panobinostat	Brain Stem Glioma	2	199.02 (48.97 - 808.74)	198.92 (385.01)	7.6 (5.91)	194.48 (60.17)
Panobinostat	Nasopharyngitis	2	0.15 (0.04 - 0.59)	0.15 (9.87)	-2.76 (-4.43)	0.15 (0.05)
Panobinostat	Memory Impairment	2	0.2 (0.05 - 0.79)	0.2 (6.53)	-2.34 (-4.01)	0.2 (0.06)
Panobinostat	Meningitis	2	4.89 (1.22 - 19.56)	4.89 (6.18)	2.29 (0.62)	4.89 (1.53)
Panobinostat	Cancer Pain	2	9.94 (2.48 - 39.79)	9.93 (16.05)	3.31 (1.64)	9.92 (3.11)
Panobinostat	Adrenal Insufficiency	2	2.44 (0.61 - 9.76)	2.44 (1.7)	1.29 (-0.38)	2.44 (0.76)
Panobinostat	Paralysis	2	2.25 (0.56 - 9)	2.25 (1.39)	1.17 (-0.5)	2.25 (0.7)
Panobinostat	Pneumocystis Jirovecii Pneumonia	2	2.48 (0.62 - 9.94)	2.48 (1.77)	1.31 (-0.35)	2.48 (0.78)
Panobinostat	Hypertransaminasaemia	2	4.39 (1.1 - 17.58)	4.39 (5.24)	2.13 (0.47)	4.39 (1.38)
Panobinostat	Sinusitis	2	0.27 (0.07 - 1.09)	0.27 (3.87)	-1.87 (-3.54)	0.27 (0.09)
Panobinostat	Staphylococcal Sepsis	2	7.49 (1.87 - 29.96)	7.48 (11.22)	2.9 (1.23)	7.48 (2.34)
Panobinostat	Bone Swelling	2	32.24 (8.04 - 129.3)	32.23 (60.29)	5.01 (3.33)	32.11 (10.05)
Panobinostat	Creatinine Renal Clearance Abnormal	2	59.3 (14.75 - 238.3)	59.27 (113.79)	5.88 (4.2)	58.87 (18.38)
Panobinostat	Blast Cell Count Increased	2	24.95 (6.22 - 99.99)	24.94 (45.82)	4.64 (2.97)	24.87 (7.78)
Panobinostat	Hepatic Failure	2	1.16 (0.29 - 4.63)	1.16 (0.04)	0.21 (-1.46)	1.16 (0.36)
Panobinostat	Diverticulitis	2	1.02 (0.26 - 4.09)	1.02 (0)	0.03 (-1.63)	1.02 (0.32)
Panobinostat	Subdural Haemorrhage	2	10.61 (2.65 - 42.47)	10.6 (17.38)	3.4 (1.74)	10.59 (3.32)
Panobinostat	Retinal Haemorrhage	2	5.2 (1.3 - 20.79)	5.19 (6.77)	2.38 (0.71)	5.19 (1.63)
Panobinostat	Posterior Reversible Encephalopathy Syndrome	2	2.83 (0.71 - 11.34)	2.83 (2.37)	1.5 (-0.16)	2.83 (0.89)
Panobinostat	Haematuria	2	0.86 (0.21 - 3.43)	0.86 (0.05)	-0.22 (-1.89)	0.86 (0.27)
Panobinostat	Prothrombin Time Prolonged	2	9.1 (2.27 - 36.42)	9.09 (14.39)	3.18 (1.52)	9.09 (2.85)

Panobinostat	Depression	2	0.14 (0.04 - 0.57)	0.14 (10.39)	-2.81 (-4.48)	0.14 (0.04)
Panobinostat	Metastases To Liver	2	1.66 (0.41 - 6.64)	1.66 (0.52)	0.73 (-0.94)	1.66 (0.52)
Panobinostat	Peripheral Coldness	2	2.27 (0.57 - 9.08)	2.27 (1.42)	1.18 (-0.48)	2.27 (0.71)
Panobinostat	Bradycardia	2	0.58 (0.14 - 2.31)	0.58 (0.62)	-0.79 (-2.46)	0.58 (0.18)
Panobinostat	Large Intestinal Ulcer	2	11.88 (2.97 - 47.54)	11.87 (19.88)	3.57 (1.9)	11.86 (3.71)
Panobinostat	Rash Maculo-Papular	2	1.45 (0.36 - 5.8)	1.45 (0.28)	0.54 (-1.13)	1.45 (0.45)
Panobinostat	Device Related Infection	2	1.78 (0.45 - 7.14)	1.78 (0.69)	0.83 (-0.83)	1.78 (0.56)
Panobinostat	Respiratory Arrest	2	1.6 (0.4 - 6.39)	1.6 (0.45)	0.67 (-0.99)	1.6 (0.5)
Panobinostat	Liver Injury	2	1.35 (0.34 - 5.39)	1.35 (0.18)	0.43 (-1.24)	1.35 (0.42)
Panobinostat	Osteonecrosis Of Jaw	2	1.2 (0.3 - 4.78)	1.2 (0.06)	0.26 (-1.41)	1.2 (0.37)
Panobinostat	Pleocytosis	2	50.92 (12.68 - 204.52)	50.9 (97.27)	5.66 (3.99)	50.61 (15.81)
Panobinostat	Dysgeusia	2	0.41 (0.1 - 1.64)	0.41 (1.69)	-1.28 (-2.95)	0.41 (0.13)
Panobinostat	Encephalitis	2	4.57 (1.14 - 18.28)	4.57 (5.57)	2.19 (0.52)	4.57 (1.43)
Panobinostat	Ocular Hyperaemia	2	0.64 (0.16 - 2.56)	0.64 (0.41)	-0.64 (-2.31)	0.64 (0.2)
Panobinostat	Chronic Obstructive Pulmonary Disease	2	0.59 (0.15 - 2.35)	0.59 (0.58)	-0.76 (-2.43)	0.59 (0.18)
Panobinostat	Bronchial Haemorrhage	2	83.24 (20.67 - 335.18)	83.2 (160.89)	6.36 (4.69)	82.42 (25.7)
Panobinostat	Escherichia Infection	2	3.85 (0.96 - 15.4)	3.85 (4.21)	1.94 (0.28)	3.85 (1.21)
Panobinostat	Pericarditis	2	1.88 (0.47 - 7.52)	1.88 (0.82)	0.91 (-0.76)	1.88 (0.59)
Panobinostat	Spinal Pain	2	2 (0.5 - 8)	2 (1)	1 (-0.67)	2 (0.63)
Panobinostat	Thrombotic Microangiopathy	2	3.04 (0.76 - 12.14)	3.03 (2.73)	1.6 (-0.07)	3.03 (0.95)
Panobinostat	Pneumonia Pneumococcal	2	22.14 (5.53 - 88.72)	22.13 (40.25)	4.46 (2.79)	22.08 (6.91)
Panobinostat	Rhinorrhoea	2	0.42 (0.1 - 1.66)	0.42 (1.64)	-1.26 (-2.93)	0.42 (0.13)
Panobinostat	Diastolic Dysfunction	2	11.38 (2.84 - 45.54)	11.37 (18.89)	3.51 (1.84)	11.36 (3.56)
Panobinostat	Obstructive Airways Disorder	2	2.28 (0.57 - 9.12)	2.28 (1.44)	1.19 (-0.48)	2.28 (0.71)
Panobinostat	Angioedema	2	0.69 (0.17 - 2.77)	0.69 (0.27)	-0.53 (-2.2)	0.69 (0.22)

Panobinostat	Product Administered To Patient Of Inappropriate Age	2	1.74 (0.43 - 6.95)	1.74 (0.62)	0.8 (-0.87)	1.74 (0.54)
Panobinostat	Hypoaesthesia Oral	2	2.14 (0.53 - 8.54)	2.14 (1.21)	1.09 (-0.57)	2.13 (0.67)
Panobinostat	Pneumatosis Intestinalis	2	10.12 (2.53 - 40.53)	10.12 (16.42)	3.34 (1.67)	10.11 (3.17)
Panobinostat	Pneumomediastinum	2	17.51 (4.37 - 70.12)	17.5 (31.05)	4.13 (2.46)	17.47 (5.47)
Panobinostat	Pneumoperitoneum	2	16.66 (4.16 - 66.74)	16.66 (29.38)	4.06 (2.39)	16.63 (5.21)
Panobinostat	Hepatosplenic Candidiasis	2	203.7 (50.11 - 828.08)	203.6 (393.95)	7.64 (5.94)	198.95 (61.53)
Panobinostat	Hodgkin'S Disease	2	7.6 (1.9 - 30.42)	7.6 (11.45)	2.92 (1.26)	7.59 (2.38)
Panobinostat	Blood Creatinine Decreased	2	9.2 (2.3 - 36.82)	9.2 (14.59)	3.2 (1.53)	9.19 (2.88)
Panobinostat	Diplopia	2	1.28 (0.32 - 5.13)	1.28 (0.13)	0.36 (-1.31)	1.28 (0.4)
Panobinostat	Dry Skin	2	0.19 (0.05 - 0.77)	0.19 (6.83)	-2.38 (-4.05)	0.19 (0.06)
Panobinostat	Eczema	2	0.76 (0.19 - 3.03)	0.76 (0.15)	-0.4 (-2.06)	0.76 (0.24)
Panobinostat	Electrocardiogram St Segment Abnormal	2	67.37 (16.75 - 270.93)	67.34 (129.7)	6.06 (4.39)	66.83 (20.86)
Panobinostat	Escherichia Urinary Tract Infection	2	7.13 (1.78 - 28.53)	7.12 (10.52)	2.83 (1.16)	7.12 (2.23)
Panobinostat	Eye Pain	2	0.54 (0.14 - 2.18)	0.54 (0.76)	-0.88 (-2.54)	0.54 (0.17)
Panobinostat	Impaired Gastric Emptying	2	3.76 (0.94 - 15.03)	3.76 (4.04)	1.91 (0.24)	3.75 (1.18)
Panobinostat	Odynophagia	2	5.66 (1.41 - 22.65)	5.66 (7.66)	2.5 (0.83)	5.65 (1.77)
Panobinostat	Pelvic Venous Thrombosis	2	30.11 (7.51 - 120.73)	30.1 (56.07)	4.91 (3.24)	30 (9.39)
Panobinostat	Vena Cava Thrombosis	2	24.05 (6 - 96.37)	24.04 (44.03)	4.58 (2.91)	23.97 (7.5)
Panobinostat	Haemorrhagic Stroke	2	3.68 (0.92 - 14.71)	3.67 (3.89)	1.88 (0.21)	3.67 (1.15)
Panobinostat	Therapy Interrupted	2	0.45 (0.11 - 1.79)	0.45 (1.36)	-1.16 (-2.83)	0.45 (0.14)
Panobinostat	Myelosuppression	2	1.12 (0.28 - 4.49)	1.12 (0.03)	0.17 (-1.5)	1.12 (0.35)
Panobinostat	Blood Pressure Increased	2	0.19 (0.05 - 0.75)	0.19 (7.05)	-2.41 (-4.08)	0.19 (0.06)
Panobinostat	Adverse Event	2	0.31 (0.08 - 1.25)	0.31 (3.04)	-1.68 (-3.35)	0.31 (0.1)
Panobinostat	Hypervolaemia	1	0.76 (0.11 - 5.37)	0.76 (0.08)	-0.4 (-2.07)	0.76 (0.15)
Panobinostat	Bicytopenia	1	7.15 (1.01 - 50.79)	7.15 (5.28)	2.84 (1.17)	7.14 (1.38)

Panobinostat	Gastrointestinal Viral Infection	1	13.57 (1.91 - 96.48)	13.56 (11.62)	3.76 (2.09)	13.54 (2.62)
Panobinostat	Intervertebral Disc Disorder	1	3.94 (0.55 - 28)	3.94 (2.19)	1.98 (0.31)	3.94 (0.76)
Panobinostat	Hypokinesia	1	0.93 (0.13 - 6.62)	0.93 (0)	-0.1 (-1.77)	0.93 (0.18)
Panobinostat	Neutrophil Count Abnormal	1	5 (0.7 - 35.5)	5 (3.19)	2.32 (0.65)	4.99 (0.97)
Panobinostat	Pelvic Mass	1	21.37 (3 - 152.12)	21.37 (19.37)	4.41 (2.74)	21.32 (4.13)
Panobinostat	Malignant Pleural Effusion	1	9.16 (1.29 - 65.1)	9.16 (7.26)	3.19 (1.52)	9.15 (1.77)
Panobinostat	Neurogenic Bladder	1	8.1 (1.14 - 57.54)	8.09 (6.21)	3.02 (1.35)	8.09 (1.57)
Panobinostat	Drug Hypersensitivity	1	0.06 (0.01 - 0.43)	0.06 (14.61)	-4.04 (-5.71)	0.06 (0.01)
Panobinostat	Oropharyngeal Discomfort	1	1.54 (0.22 - 10.96)	1.54 (0.19)	0.63 (-1.04)	1.54 (0.3)
Panobinostat	Skin Exfoliation	1	0.16 (0.02 - 1.11)	0.16 (4.53)	-2.67 (-4.34)	0.16 (0.03)
Panobinostat	Systemic Inflammatory Response Syndrome	1	3.88 (0.55 - 27.58)	3.88 (2.14)	1.96 (0.29)	3.88 (0.75)
Panobinostat	Chronic Gastritis	1	5.36 (0.75 - 38.1)	5.36 (3.55)	2.42 (0.75)	5.36 (1.04)
Panobinostat	Blood Triglycerides Increased	1	1.17 (0.16 - 8.3)	1.17 (0.02)	0.22 (-1.44)	1.17 (0.23)
Panobinostat	Organising Pneumonia	1	2.91 (0.41 - 20.67)	2.91 (1.25)	1.54 (-0.13)	2.91 (0.56)
Panobinostat	Pleural Disorder	1	29.95 (4.2 - 213.39)	29.94 (27.88)	4.9 (3.22)	29.84 (5.77)
Panobinostat	Urinary Bladder Haemorrhage	1	6.95 (0.98 - 49.36)	6.94 (5.08)	2.79 (1.13)	6.94 (1.35)
Panobinostat	Coronary Artery Disease	1	0.77 (0.11 - 5.46)	0.77 (0.07)	-0.38 (-2.05)	0.77 (0.15)
Panobinostat	Electrocardiogram T Wave Inversion	1	9.74 (1.37 - 69.21)	9.73 (7.83)	3.28 (1.61)	9.72 (1.88)
Panobinostat	Otitis Media	1	5.03 (0.71 - 35.71)	5.03 (3.22)	2.33 (0.66)	5.02 (0.97)
Panobinostat	International Normalised Ratio Abnormal	1	5.09 (0.72 - 36.15)	5.09 (3.28)	2.35 (0.68)	5.08 (0.99)
Panobinostat	Bone Marrow Transplant Rejection	1	87.43 (12.19 - 626.97)	87.41 (84.56)	6.44 (4.74)	86.54 (16.65)
Panobinostat	Diffuse Large B-Cell Lymphoma	1	1.82 (0.26 - 12.92)	1.82 (0.37)	0.86 (-0.8)	1.82 (0.35)
Panobinostat	Pulmonary Fibrosis	1	0.86 (0.12 - 6.14)	0.86 (0.02)	-0.21 (-1.88)	0.86 (0.17)
Panobinostat	Bradyphrenia	1	2.07 (0.29 - 14.7)	2.07 (0.55)	1.05 (-0.62)	2.07 (0.4)

Panobinostat	Conjunctivitis	1	0.78 (0.11 - 5.53)	0.78 (0.06)	-0.36 (-2.03)	0.78 (0.15)
Panobinostat	Ketoacidosis	1	1.95 (0.27 - 13.86)	1.95 (0.46)	0.96 (-0.7)	1.95 (0.38)
Panobinostat	Fluid Retention	1	0.26 (0.04 - 1.88)	0.26 (2.04)	-1.92 (-3.58)	0.26 (0.05)
Panobinostat	Hyperthyroidism	1	1.02 (0.14 - 7.26)	1.02 (0)	0.03 (-1.64)	1.02 (0.2)
Panobinostat	Troponin T Increased	1	12.73 (1.79 - 90.51)	12.73 (10.79)	3.67 (2)	12.71 (2.46)
Panobinostat	Blood Creatine Phosphokinase Increased	1	0.7 (0.1 - 4.96)	0.7 (0.13)	-0.52 (-2.18)	0.7 (0.14)
Panobinostat	Flatulence	1	0.27 (0.04 - 1.94)	0.27 (1.93)	-1.87 (-3.54)	0.27 (0.05)
Panobinostat	Human Rhinovirus Test Positive	1	47.3 (6.62 - 337.65)	47.29 (45.06)	5.56 (3.88)	47.03 (9.08)
Panobinostat	Heart Rate Irregular	1	0.69 (0.1 - 4.93)	0.69 (0.13)	-0.53 (-2.19)	0.69 (0.13)
Panobinostat	Gastrointestinal Pain	1	1.3 (0.18 - 9.22)	1.3 (0.07)	0.38 (-1.29)	1.3 (0.25)
Panobinostat	Bundle Branch Block	1	21.11 (2.97 - 150.26)	21.11 (19.11)	4.4 (2.72)	21.06 (4.08)
Panobinostat	Actinic Keratosis	1	5.18 (0.73 - 36.78)	5.18 (3.37)	2.37 (0.7)	5.17 (1)
Panobinostat	Dermatitis Exfoliative	1	5.18 (0.73 - 36.82)	5.18 (3.37)	2.37 (0.7)	5.18 (1)
Panobinostat	Pericardial Effusion	1	0.69 (0.1 - 4.9)	0.69 (0.14)	-0.54 (-2.2)	0.69 (0.13)
Panobinostat	Skin Hypertrophy	1	5.61 (0.79 - 39.83)	5.6 (3.78)	2.49 (0.82)	5.6 (1.09)
Panobinostat	Vertigo Positional	1	9.15 (1.29 - 65.03)	9.15 (7.25)	3.19 (1.52)	9.14 (1.77)
Panobinostat	Pyelonephritis Acute	1	7.74 (1.09 - 55.02)	7.74 (5.86)	2.95 (1.28)	7.73 (1.5)
Panobinostat	Non-Cardiac Chest Pain	1	5.77 (0.81 - 41.03)	5.77 (3.94)	2.53 (0.86)	5.77 (1.12)
Panobinostat	Pelvic Pain	1	1.02 (0.14 - 7.25)	1.02 (0)	0.03 (-1.64)	1.02 (0.2)
Panobinostat	Autonomic Nervous System Imbalance	1	5.56 (0.78 - 39.5)	5.56 (3.74)	2.47 (0.81)	5.55 (1.08)
Panobinostat	Myopathy	1	1.99 (0.28 - 14.16)	1.99 (0.49)	1 (-0.67)	1.99 (0.39)
Panobinostat	Renal Function Test Abnormal	1	3.08 (0.43 - 21.85)	3.08 (1.4)	1.62 (-0.05)	3.07 (0.6)
Panobinostat	Anger	1	0.48 (0.07 - 3.39)	0.48 (0.57)	-1.07 (-2.73)	0.48 (0.09)
Panobinostat	Prostatic Specific Antigen Decreased	1	37.63 (5.28 - 268.36)	37.62 (35.49)	5.23 (3.55)	37.46 (7.24)

Panobinostat	Albumin Globulin Ratio Decreased	1	81.65 (11.39 - 585.18)	81.63 (78.9)	6.34 (4.65)	80.88 (15.57)
Panobinostat	Blood Immunoglobulin A Decreased	1	24.04 (3.38 - 171.19)	24.04 (22.02)	4.58 (2.91)	23.97 (4.64)
Panobinostat	Blood Immunoglobulin M Decreased	1	14.62 (2.06 - 103.99)	14.62 (12.66)	3.87 (2.2)	14.59 (2.83)
Panobinostat	Carbon Dioxide Decreased	1	16.21 (2.28 - 115.3)	16.2 (14.24)	4.02 (2.34)	16.18 (3.13)
Panobinostat	Glomerular Filtration Rate Decreased	1	1.18 (0.17 - 8.36)	1.18 (0.03)	0.24 (-1.43)	1.18 (0.23)
Panobinostat	Monocytosis	1	30.26 (4.25 - 215.63)	30.26 (28.19)	4.91 (3.24)	30.15 (5.83)
Panobinostat	Neutrophilia	1	2.35 (0.33 - 16.69)	2.35 (0.77)	1.23 (-0.44)	2.35 (0.46)
Panobinostat	Failure To Thrive	1	3.71 (0.52 - 26.33)	3.71 (1.97)	1.89 (0.22)	3.7 (0.72)
Panobinostat	Helicobacter Infection	1	1.56 (0.22 - 11.07)	1.56 (0.2)	0.64 (-1.03)	1.56 (0.3)
Panobinostat	Cardiac Operation	1	1.83 (0.26 - 13)	1.83 (0.38)	0.87 (-0.8)	1.83 (0.35)
Panobinostat	Product Prescribing Error	1	0.29 (0.04 - 2.06)	0.29 (1.74)	-1.79 (-3.45)	0.29 (0.06)
Panobinostat	Venous Thrombosis Limb	1	7.37 (1.04 - 52.35)	7.36 (5.5)	2.88 (1.21)	7.36 (1.43)
Panobinostat	Pneumonia Parainfluenzae Viral	1	99.48 (13.85 - 714.42)	99.46 (96.36)	6.62 (4.93)	98.34 (18.89)
Panobinostat	Abdominal Tenderness	1	3.4 (0.48 - 24.15)	3.4 (1.69)	1.76 (0.1)	3.4 (0.66)
Panobinostat	Bacterial Test Positive	1	6.28 (0.88 - 44.6)	6.27 (4.43)	2.65 (0.98)	6.27 (1.22)
Panobinostat	Blood Chloride Increased	1	11.76 (1.65 - 83.62)	11.76 (9.83)	3.55 (1.88)	11.74 (2.27)
Panobinostat	Blood Phosphorus Increased	1	5.81 (0.82 - 41.3)	5.81 (3.98)	2.54 (0.87)	5.81 (1.13)
Panobinostat	Duodenal Ulcer Haemorrhage	1	5.48 (0.77 - 38.92)	5.48 (3.66)	2.45 (0.78)	5.47 (1.06)
Panobinostat	Glycosylated Haemoglobin Increased	1	0.54 (0.08 - 3.81)	0.54 (0.4)	-0.9 (-2.57)	0.54 (0.1)
Panobinostat	Monocyte Percentage Increased	1	21.06 (2.96 - 149.89)	21.05 (19.06)	4.39 (2.72)	21.01 (4.07)
Panobinostat	Occult Blood Positive	1	9.1 (1.28 - 64.69)	9.1 (7.2)	3.18 (1.52)	9.09 (1.76)
Panobinostat	Urine Ketone Body Present	1	8.76 (1.23 - 62.27)	8.76 (6.87)	3.13 (1.46)	8.75 (1.7)
Panobinostat	Acidosis	1	2.23 (0.31 - 15.87)	2.23 (0.68)	1.16 (-0.51)	2.23 (0.43)

Panobinostat	Hepatic Encephalopathy	1	1.66 (0.23 - 11.78)	1.66 (0.26)	0.73 (-0.94)	1.66 (0.32)
Panobinostat	Shock	1	0.77 (0.11 - 5.5)	0.78 (0.07)	-0.37 (-2.03)	0.78 (0.15)
Panobinostat	Neoplasm	1	1.23 (0.17 - 8.76)	1.23 (0.04)	0.3 (-1.36)	1.23 (0.24)
Panobinostat	Lymphopenia	1	0.97 (0.14 - 6.9)	0.97 (0)	-0.04 (-1.71)	0.97 (0.19)
Panobinostat	Radiation Oesophagitis	1	46.53 (6.52 - 332.17)	46.52 (44.31)	5.53 (3.85)	46.28 (8.94)
Panobinostat	Performance Status Decreased	1	3.49 (0.49 - 24.8)	3.49 (1.78)	1.8 (0.14)	3.49 (0.68)
Panobinostat	Quality Of Life Decreased	1	1.33 (0.19 - 9.46)	1.33 (0.08)	0.41 (-1.25)	1.33 (0.26)
Panobinostat	Respiratory Tract Congestion	1	0.95 (0.13 - 6.74)	0.95 (0)	-0.08 (-1.74)	0.95 (0.18)
Panobinostat	Secretion Discharge	1	1.05 (0.15 - 7.46)	1.05 (0)	0.07 (-1.6)	1.05 (0.2)
Panobinostat	Plasma Cell Myeloma Recurrent	1	3.78 (0.53 - 26.87)	3.78 (2.05)	1.92 (0.25)	3.78 (0.73)
Panobinostat	Gastrointestinal Perforation	1	3.6 (0.51 - 25.59)	3.6 (1.88)	1.85 (0.18)	3.6 (0.7)
Panobinostat	Peritonitis	1	0.8 (0.11 - 5.68)	0.8 (0.05)	-0.32 (-1.99)	0.8 (0.16)
Panobinostat	Incorrect Dosage Administered	1	2.27 (0.32 - 16.15)	2.27 (0.71)	1.18 (-0.48)	2.27 (0.44)
Panobinostat	Platelet Transfusion	1	10.34 (1.45 - 73.51)	10.34 (8.43)	3.37 (1.7)	10.33 (2)
Panobinostat	Transfusion	1	1.23 (0.17 - 8.74)	1.23 (0.04)	0.3 (-1.37)	1.23 (0.24)
Panobinostat	Metastasis	1	2.1 (0.3 - 14.93)	2.1 (0.58)	1.07 (-0.6)	2.1 (0.41)
Panobinostat	Parotid Gland Enlargement	1	28.95 (4.06 - 206.23)	28.94 (26.89)	4.85 (3.18)	28.85 (5.58)
Panobinostat	Sputum Abnormal	1	22.9 (3.22 - 163.01)	22.89 (20.88)	4.51 (2.84)	22.83 (4.42)
Panobinostat	Cardiogenic Shock	1	1.13 (0.16 - 8)	1.13 (0.01)	0.17 (-1.5)	1.13 (0.22)
Panobinostat	Cardiomegaly	1	1.71 (0.24 - 12.13)	1.71 (0.29)	0.77 (-0.9)	1.71 (0.33)
Panobinostat	Paresis	1	7.46 (1.05 - 53.03)	7.46 (5.59)	2.9 (1.23)	7.45 (1.44)
Panobinostat	Subcutaneous Abscess	1	3.05 (0.43 - 21.65)	3.05 (1.37)	1.61 (-0.06)	3.05 (0.59)
Panobinostat	Dialysis	1	1.25 (0.18 - 8.91)	1.25 (0.05)	0.33 (-1.34)	1.25 (0.24)
Panobinostat	Oesophageal Ulcer	1	4.71 (0.66 - 33.45)	4.71 (2.92)	2.23 (0.57)	4.71 (0.91)
Panobinostat	Wheezing	1	0.25 (0.03 - 1.76)	0.25 (2.28)	-2.01 (-3.68)	0.25 (0.05)
Panobinostat	Corynebacterium Infection	1	29.64 (4.16 - 211.19)	29.63 (27.57)	4.88 (3.21)	29.54 (5.71)
Panobinostat	Myoclonus	1	1.4 (0.2 - 9.95)	1.4 (0.11)	0.49 (-1.18)	1.4 (0.27)

Panobinostat	Groin Pain	1	1.68 (0.24 - 11.91)	1.68 (0.27)	0.75 (-0.92)	1.68 (0.33)
Panobinostat	Hepatotoxicity	1	0.66 (0.09 - 4.66)	0.66 (0.18)	-0.61 (-2.28)	0.66 (0.13)
Panobinostat	Blood Immunoglobulin A Increased	1	31.7 (4.45 - 225.94)	31.7 (29.62)	4.98 (3.31)	31.58 (6.11)
Panobinostat	Citrobacter Infection	1	40.44 (5.67 - 288.51)	40.44 (38.28)	5.33 (3.65)	40.25 (7.78)
Panobinostat	Subgaleal Haematoma	1	65.08 (9.1 - 465.52)	65.06 (62.61)	6.01 (4.33)	64.58 (12.45)
Panobinostat	Wound Dehiscence	1	5.39 (0.76 - 38.29)	5.39 (3.57)	2.43 (0.76)	5.39 (1.04)
Panobinostat	Meniscus Injury	1	1.98 (0.28 - 14.09)	1.98 (0.49)	0.99 (-0.68)	1.98 (0.38)
Panobinostat	Agranulocytosis	1	0.88 (0.12 - 6.22)	0.88 (0.02)	-0.19 (-1.86)	0.88 (0.17)
Panobinostat	Hypermagnesaemia	1	22.9 (3.22 - 163.01)	22.89 (20.88)	4.51 (2.84)	22.83 (4.42)
Panobinostat	Malignant Melanoma	1	1 (0.14 - 7.07)	1 (0)	-0.01 (-1.67)	1 (0.19)
Panobinostat	Aphasia	1	0.51 (0.07 - 3.66)	0.51 (0.46)	-0.96 (-2.62)	0.51 (0.1)
Panobinostat	Cranial Nerve Injury	1	157.37 (21.77 - 1137.45)	157.33 (152.56)	7.27 (5.56)	154.54 (29.53)
Panobinostat	Monoparesis	1	8.71 (1.22 - 61.89)	8.71 (6.81)	3.12 (1.45)	8.7 (1.69)
Panobinostat	Disturbance In Attention	1	0.28 (0.04 - 1.97)	0.28 (1.89)	-1.85 (-3.52)	0.28 (0.05)
Panobinostat	Fungal Cystitis	1	188.16 (25.94 - 1364.7)	188.11 (182.16)	7.52 (5.81)	184.13 (35.08)
Panobinostat	Localised Infection	1	0.56 (0.08 - 3.95)	0.56 (0.35)	-0.85 (-2.51)	0.56 (0.11)
Panobinostat	H1N1 Influenza	1	14.14 (1.99 - 100.58)	14.14 (12.19)	3.82 (2.15)	14.12 (2.73)
Panobinostat	Type 1 Diabetes Mellitus	1	2.72 (0.38 - 19.36)	2.72 (1.09)	1.45 (-0.22)	2.72 (0.53)
Panobinostat	Cold Sweat	1	0.92 (0.13 - 6.56)	0.92 (0.01)	-0.11 (-1.78)	0.92 (0.18)
Panobinostat	Staring	1	7.68 (1.08 - 54.58)	7.68 (5.8)	2.94 (1.27)	7.67 (1.49)
Panobinostat	Bone Neoplasm	1	13.78 (1.94 - 98.02)	13.78 (11.83)	3.78 (2.11)	13.76 (2.66)
Panobinostat	Phlebitis	1	3.64 (0.51 - 25.87)	3.64 (1.91)	1.86 (0.2)	3.64 (0.71)
Panobinostat	Kussmaul Respiration	1	81.65 (11.39 - 585.18)	81.63 (78.9)	6.34 (4.65)	80.88 (15.57)
Panobinostat	Bacterial Infection	1	0.83 (0.12 - 5.92)	0.83 (0.03)	-0.26 (-1.93)	0.83 (0.16)
Panobinostat	Csf Cell Count Increased	1	131.14 (18.2 - 945.09)	131.11 (127.19)	7.01 (5.31)	129.17 (24.74)

Panobinostat	Serratia Sepsis	1	169.71 (23.45 - 1228.36)	169.67 (164.45)	7.38 (5.67)	166.43 (31.76)
Panobinostat	Swelling Face	1	0.24 (0.03 - 1.7)	0.24 (2.41)	-2.06 (-3.73)	0.24 (0.05)
Panobinostat	Atrioventricular Block	1	2.16 (0.3 - 15.32)	2.16 (0.62)	1.11 (-0.56)	2.16 (0.42)
Panobinostat	Bifascicular Block	1	108.19 (15.05 - 777.69)	108.16 (104.86)	6.74 (5.04)	106.84 (20.51)
Panobinostat	Pseudomonas Infection	1	1.94 (0.27 - 13.81)	1.94 (0.46)	0.96 (-0.71)	1.94 (0.38)
Panobinostat	Wound Complication	1	3.8 (0.54 - 27.03)	3.8 (2.07)	1.93 (0.26)	3.8 (0.74)
Panobinostat	Drug-Induced Liver Injury	1	0.44 (0.06 - 3.15)	0.44 (0.7)	-1.17 (-2.84)	0.44 (0.09)
Panobinostat	Immunosuppression	1	1.68 (0.24 - 11.9)	1.68 (0.27)	0.74 (-0.92)	1.68 (0.32)
Panobinostat	Metastatic Malignant Melanoma	1	6.2 (0.87 - 44.02)	6.19 (4.35)	2.63 (0.96)	6.19 (1.2)
Panobinostat	Acute Psychosis	1	6.64 (0.93 - 47.2)	6.64 (4.79)	2.73 (1.06)	6.64 (1.29)
Panobinostat	Acute Lymphocytic Leukaemia	1	4.96 (0.7 - 35.24)	4.96 (3.16)	2.31 (0.64)	4.96 (0.96)
Panobinostat	Facial Paresis	1	4.53 (0.64 - 32.19)	4.53 (2.75)	2.18 (0.51)	4.53 (0.88)
Panobinostat	Protein Total Increased	1	5.73 (0.81 - 40.73)	5.73 (3.9)	2.52 (0.85)	5.73 (1.11)
Panobinostat	Enterobacter Infection	1	10.1 (1.42 - 71.8)	10.1 (8.19)	3.33 (1.66)	10.09 (1.95)
Panobinostat	Hypoalbuminaemia	1	2.16 (0.3 - 15.33)	2.16 (0.62)	1.11 (-0.56)	2.16 (0.42)
Panobinostat	Squamous Cell Carcinoma	1	1.55 (0.22 - 11.04)	1.55 (0.2)	0.64 (-1.03)	1.55 (0.3)
Panobinostat	Colitis	1	0.39 (0.05 - 2.76)	0.39 (0.96)	-1.36 (-3.03)	0.39 (0.08)
Panobinostat	Abscess	1	0.94 (0.13 - 6.68)	0.94 (0)	-0.09 (-1.75)	0.94 (0.18)
Panobinostat	Intervertebral Discitis	1	8.18 (1.15 - 58.14)	8.18 (6.3)	3.03 (1.36)	8.17 (1.58)
Panobinostat	Cardiac Amyloidosis	1	13.27 (1.87 - 94.4)	13.27 (11.33)	3.73 (2.06)	13.25 (2.57)
Panobinostat	Clostridial Infection	1	15.32 (2.15 - 108.97)	15.32 (13.36)	3.93 (2.26)	15.29 (2.96)
Panobinostat	Regurgitation	1	4.99 (0.7 - 35.46)	4.99 (3.19)	2.32 (0.65)	4.99 (0.97)
Panobinostat	Acute Respiratory Distress Syndrome	1	0.95 (0.13 - 6.78)	0.95 (0)	-0.07 (-1.73)	0.95 (0.19)

Panobinostat	International Normalised Ratio Fluctuation	1	8.49 (1.19 - 60.37)	8.49 (6.6)	3.08 (1.42)	8.48 (1.64)
Panobinostat	Deafness	1	0.56 (0.08 - 3.98)	0.56 (0.34)	-0.83 (-2.5)	0.56 (0.11)
Panobinostat	Atypical Mycobacterial Infection	1	11.57 (1.63 - 82.27)	11.57 (9.64)	3.53 (1.86)	11.55 (2.24)
Panobinostat	Epilepsy	1	0.51 (0.07 - 3.63)	0.51 (0.47)	-0.97 (-2.63)	0.51 (0.1)
Panobinostat	Candida Pneumonia	1	62.72 (8.77 - 448.53)	62.7 (60.28)	5.96 (4.28)	62.26 (12)
Panobinostat	Apnoea	1	2.3 (0.32 - 16.31)	2.3 (0.73)	1.2 (-0.47)	2.3 (0.45)
Panobinostat	Metastases To Central Nervous System	1	1.17 (0.16 - 8.31)	1.17 (0.02)	0.23 (-1.44)	1.17 (0.23)
Panobinostat	Nutritional Condition Abnormal	1	22.9 (3.22 - 163.01)	22.89 (20.88)	4.51 (2.84)	22.83 (4.42)
Panobinostat	Breast Cancer Metastatic	1	1.64 (0.23 - 11.62)	1.64 (0.25)	0.71 (-0.96)	1.64 (0.32)
Panobinostat	Nocturia	1	1.25 (0.18 - 8.88)	1.25 (0.05)	0.32 (-1.35)	1.25 (0.24)
Panobinostat	Lung Disorder	1	0.31 (0.04 - 2.17)	0.31 (1.57)	-1.71 (-3.37)	0.31 (0.06)
Panobinostat	Mental Impairment	1	0.64 (0.09 - 4.55)	0.64 (0.2)	-0.64 (-2.31)	0.64 (0.12)
Panobinostat	Movement Disorder	1	0.52 (0.07 - 3.7)	0.52 (0.44)	-0.94 (-2.61)	0.52 (0.1)
Panobinostat	Hemiparesis	1	0.96 (0.14 - 6.85)	0.96 (0)	-0.05 (-1.72)	0.96 (0.19)
Panobinostat	Gastrointestinal Toxicity	1	3.17 (0.45 - 22.54)	3.17 (1.49)	1.67 (0)	3.17 (0.61)
Panobinostat	Blood Bilirubin Increased	1	0.66 (0.09 - 4.71)	0.66 (0.17)	-0.59 (-2.26)	0.66 (0.13)
Panobinostat	Gamma-Glutamyltransferase Increased	1	0.86 (0.12 - 6.14)	0.86 (0.02)	-0.21 (-1.88)	0.86 (0.17)
Panobinostat	Enterococcal Infection	1	3.46 (0.49 - 24.58)	3.46 (1.75)	1.79 (0.12)	3.46 (0.67)
Panobinostat	Hyperphosphataemia	1	8.02 (1.13 - 57.01)	8.02 (6.14)	3 (1.33)	8.01 (1.55)
Panobinostat	Oxygen Saturation Decreased	1	0.25 (0.04 - 1.8)	0.25 (2.2)	-1.98 (-3.64)	0.25 (0.05)
Panobinostat	Sinus Arrest	1	12.07 (1.7 - 85.83)	12.07 (10.14)	3.59 (1.92)	12.05 (2.33)
Panobinostat	Systemic Mycosis	1	13.42 (1.89 - 95.43)	13.42 (11.47)	3.74 (2.07)	13.4 (2.59)
Panobinostat	Compression Fracture	1	3.8 (0.54 - 27.03)	3.8 (2.07)	1.93 (0.26)	3.8 (0.74)
Panobinostat	Tooth Disorder	1	0.62 (0.09 - 4.41)	0.62 (0.23)	-0.69 (-2.35)	0.62 (0.12)

Panobinostat	Basal Cell Carcinoma	1	0.89 (0.13 - 6.32)	0.89 (0.01)	-0.17 (-1.83)	0.89 (0.17)
Panobinostat	Hypersensitivity	1	0.08 (0.01 - 0.53)	0.08 (11.37)	-3.73 (-5.39)	0.08 (0.01)
Panobinostat	Periorbital Oedema	1	3.24 (0.46 - 22.98)	3.23 (1.54)	1.69 (0.03)	3.23 (0.63)
Panobinostat	Lymphocyte Count Increased	1	4.41 (0.62 - 31.31)	4.41 (2.63)	2.14 (0.47)	4.4 (0.85)
Panobinostat	Splenomegaly	1	1.3 (0.18 - 9.25)	1.3 (0.07)	0.38 (-1.29)	1.3 (0.25)
Panobinostat	T-Cell Prolymphocytic Leukaemia	1	157.37 (21.77 - 1137.45)	157.33 (152.56)	7.27 (5.56)	154.54 (29.53)
Panobinostat	Neutrophil Count Increased	1	1.47 (0.21 - 10.47)	1.47 (0.15)	0.56 (-1.11)	1.47 (0.29)
Panobinostat	Viral Infection	1	0.46 (0.06 - 3.25)	0.46 (0.64)	-1.13 (-2.79)	0.46 (0.09)
Panobinostat	Cardiopulmonary Failure	1	4.74 (0.67 - 33.69)	4.74 (2.95)	2.24 (0.58)	4.74 (0.92)
Panobinostat	Leukaemia Monocytic	1	618.23 (81.28 - 4702.61)	618.08 (575.01)	9.17 (7.36)	576.95 (105.64)
Panobinostat	Vitamin B6 Deficiency	1	68.69 (9.6 - 491.58)	68.68 (66.17)	6.09 (4.4)	68.14 (13.13)
Panobinostat	Disease Complication	1	3.1 (0.44 - 22.01)	3.1 (1.42)	1.63 (-0.04)	3.1 (0.6)
Panobinostat	Haemorrhage Intracranial	1	0.95 (0.13 - 6.75)	0.95 (0)	-0.07 (-1.74)	0.95 (0.18)
Panobinostat	Internal Haemorrhage	1	0.88 (0.12 - 6.24)	0.88 (0.02)	-0.19 (-1.85)	0.88 (0.17)
Panobinostat	Spinal Compression Fracture	1	1.62 (0.23 - 11.5)	1.62 (0.24)	0.7 (-0.97)	1.62 (0.31)
Panobinostat	Gastrointestinal Motility Disorder	1	2.65 (0.37 - 18.79)	2.65 (1.02)	1.4 (-0.26)	2.64 (0.51)
Panobinostat	Dysstasia	1	0.49 (0.07 - 3.48)	0.49 (0.53)	-1.03 (-2.7)	0.49 (0.1)
Panobinostat	Feeding Disorder	1	0.63 (0.09 - 4.49)	0.63 (0.21)	-0.66 (-2.33)	0.63 (0.12)
Panobinostat	Tooth Abscess	1	1.47 (0.21 - 10.42)	1.47 (0.15)	0.55 (-1.11)	1.47 (0.28)
Panobinostat	Nightmare	1	0.46 (0.06 - 3.23)	0.46 (0.65)	-1.14 (-2.8)	0.46 (0.09)
Panobinostat	Breath Sounds Abnormal	1	2.91 (0.41 - 20.67)	2.91 (1.25)	1.54 (-0.13)	2.91 (0.56)
Panobinostat	Cytomegalovirus Infection	1	0.88 (0.12 - 6.24)	0.88 (0.02)	-0.19 (-1.85)	0.88 (0.17)
Panobinostat	Hepatic Cirrhosis	1	0.84 (0.12 - 5.95)	0.84 (0.03)	-0.25 (-1.92)	0.84 (0.16)
Panobinostat	Pneumonia Cytomegaloviral	1	8.25 (1.16 - 58.64)	8.25 (6.36)	3.04 (1.37)	8.24 (1.6)
Panobinostat	Hydronephrosis	1	2.19 (0.31 - 15.58)	2.19 (0.65)	1.13 (-0.53)	2.19 (0.43)

Panobinostat	Therapy Partial Responder	1	1.06 (0.15 - 7.51)	1.06 (0)	0.08 (-1.59)	1.06 (0.2)
Panobinostat	Urinary Retention	1	0.46 (0.07 - 3.3)	0.46 (0.62)	-1.1 (-2.77)	0.46 (0.09)
Panobinostat	Polyneuropathy	1	1.19 (0.17 - 8.44)	1.19 (0.03)	0.25 (-1.42)	1.19 (0.23)
Panobinostat	Extrasystoles	1	2.5 (0.35 - 17.73)	2.5 (0.9)	1.32 (-0.35)	2.49 (0.48)
Panobinostat	Dyspepsia	1	0.16 (0.02 - 1.14)	0.16 (4.4)	-2.64 (-4.31)	0.16 (0.03)
Panobinostat	Hypophysitis	1	5.63 (0.79 - 40.04)	5.63 (3.81)	2.49 (0.83)	5.63 (1.09)
Panobinostat	Nephropathy Toxic	1	1.42 (0.2 - 10.05)	1.42 (0.12)	0.5 (-1.17)	1.42 (0.27)
Panobinostat	White Blood Cell Count Increased	1	0.43 (0.06 - 3.06)	0.43 (0.75)	-1.21 (-2.88)	0.43 (0.08)
Panobinostat	Sputum Discoloured	1	1.33 (0.19 - 9.44)	1.33 (0.08)	0.41 (-1.26)	1.33 (0.26)
Panobinostat	Gastrointestinal Infection	1	1.58 (0.22 - 11.22)	1.58 (0.21)	0.66 (-1.01)	1.58 (0.31)
Panobinostat	Encephalitis Toxic	1	184.15 (25.4 - 1335.07)	184.11 (178.32)	7.49 (5.78)	180.3 (34.37)
Panobinostat	Orchitis	1	15.57 (2.19 - 110.73)	15.56 (13.6)	3.96 (2.29)	15.54 (3.01)
Panobinostat	Neurological Decompensation	1	6.54 (0.92 - 46.49)	6.54 (4.69)	2.71 (1.04)	6.54 (1.27)
Panobinostat	Bone Marrow Infiltration	1	26.71 (3.75 - 190.26)	26.71 (24.67)	4.73 (3.06)	26.63 (5.15)
Panobinostat	Plasma Cells Present	1	721.27 (93.76 - 5548.38)	721.1 (663.78)	9.38 (7.54)	665.71 (120.75)
Panobinostat	Mass	1	0.96 (0.14 - 6.83)	0.96 (0)	-0.06 (-1.72)	0.96 (0.19)
Panobinostat	Restlessness	1	0.43 (0.06 - 3.06)	0.43 (0.75)	-1.21 (-2.88)	0.43 (0.08)
Panobinostat	Cholangitis Infective	1	45.08 (6.32 - 321.74)	45.07 (42.87)	5.49 (3.81)	44.84 (8.66)
Panobinostat	Gastroenteritis	1	1.1 (0.16 - 7.82)	1.1 (0.01)	0.14 (-1.53)	1.1 (0.21)
Panobinostat	Stomach Mass	1	16.12 (2.27 - 114.66)	16.11 (14.15)	4.01 (2.34)	16.09 (3.11)
Panobinostat	Terminal State	1	1.96 (0.28 - 13.93)	1.96 (0.47)	0.97 (-0.7)	1.96 (0.38)
Panobinostat	Infrequent Bowel Movements	1	5.74 (0.81 - 40.75)	5.73 (3.91)	2.52 (0.85)	5.73 (1.11)
Panobinostat	Nasal Disorder	1	4.97 (0.7 - 35.3)	4.97 (3.17)	2.31 (0.64)	4.97 (0.96)
Panobinostat	Sphincter Of Oddi Dysfunction	1	31.13 (4.37 - 221.86)	31.13 (29.05)	4.96 (3.28)	31.02 (6)

Panobinostat	Acute Lymphocytic Leukaemia Recurrent	1	4.98 (0.7 - 35.4)	4.98 (3.18)	2.32 (0.65)	4.98 (0.97)
Panobinostat	Anion Gap Increased	1	11.84 (1.67 - 84.19)	11.84 (9.91)	3.56 (1.89)	11.82 (2.29)
Panobinostat	Anisochromia	1	721.27 (93.76 - 5548.38)	721.1 (663.78)	9.38 (7.54)	665.71 (120.75)
Panobinostat	Ascites	1	0.52 (0.07 - 3.73)	0.52 (0.43)	-0.93 (-2.6)	0.52 (0.1)
Panobinostat	Blood Pressure Abnormal	1	0.68 (0.1 - 4.84)	0.68 (0.15)	-0.55 (-2.22)	0.68 (0.13)
Panobinostat	Hyperalbuminaemia	1	254.56 (34.84 - 1860.12)	254.51 (245.29)	7.95 (6.22)	247.26 (46.82)
Panobinostat	Hyperbilirubinaemia	1	1.49 (0.21 - 10.55)	1.49 (0.16)	0.57 (-1.1)	1.49 (0.29)
Panobinostat	Hypernatraemia	1	3.27 (0.46 - 23.22)	3.27 (1.57)	1.71 (0.04)	3.27 (0.63)
Panobinostat	Hypochromasia	1	75.26 (10.51 - 539)	75.25 (72.63)	6.22 (4.53)	74.6 (14.37)
Panobinostat	Jaundice	1	0.69 (0.1 - 4.89)	0.69 (0.14)	-0.54 (-2.21)	0.69 (0.13)
Panobinostat	Klebsiella Bacteraemia	1	16.45 (2.31 - 117.06)	16.45 (14.48)	4.04 (2.37)	16.42 (3.18)
Panobinostat	Lacrimation Increased	1	0.48 (0.07 - 3.39)	0.48 (0.57)	-1.07 (-2.73)	0.48 (0.09)
Panobinostat	Lactic Acidosis	1	0.47 (0.07 - 3.31)	0.47 (0.61)	-1.1 (-2.77)	0.47 (0.09)
Panobinostat	Mastoid Effusion	1	332.89 (45.16 - 2453.77)	332.81 (318.57)	8.32 (6.57)	320.53 (60.25)
Panobinostat	Meningitis Bacterial	1	10.58 (1.49 - 75.22)	10.58 (8.66)	3.4 (1.73)	10.57 (2.05)
Panobinostat	Ocular Icterus	1	4.04 (0.57 - 28.73)	4.04 (2.29)	2.02 (0.35)	4.04 (0.78)
Panobinostat	Pachymeningitis	1	53.76 (7.52 - 384.07)	53.75 (51.45)	5.74 (4.06)	53.42 (10.31)
Panobinostat	Pneumonia Klebsiella	1	8.38 (1.18 - 59.55)	8.38 (6.49)	3.07 (1.4)	8.37 (1.62)
Panobinostat	Poikilocytosis	1	58.48 (8.18 - 418.02)	58.47 (56.11)	5.86 (4.18)	58.08 (11.2)
Panobinostat	Restrictive Pulmonary Disease	1	13 (1.83 - 92.42)	12.99 (11.05)	3.7 (2.03)	12.97 (2.51)
Panobinostat	Scleral Oedema	1	184.15 (25.4 - 1335.07)	184.11 (178.32)	7.49 (5.78)	180.3 (34.37)
Panobinostat	Sinus Tachycardia	1	1.27 (0.18 - 9.01)	1.27 (0.06)	0.34 (-1.32)	1.27 (0.25)

Panobinostat	Status Epilepticus	1	1.4 (0.2 - 9.94)	1.4 (0.11)	0.49 (-1.18)	1.4 (0.27)
Panobinostat	Subdural Effusion	1	221.93 (30.48 - 1615.74)	221.88 (214.38)	7.76 (6.03)	216.35 (41.09)
Panobinostat	Embolism	1	1.99 (0.28 - 14.11)	1.99 (0.49)	0.99 (-0.68)	1.99 (0.39)
Panobinostat	Choking	1	0.76 (0.11 - 5.41)	0.76 (0.07)	-0.39 (-2.06)	0.76 (0.15)
Panobinostat	Colon Cancer	1	0.97 (0.14 - 6.85)	0.97 (0)	-0.05 (-1.72)	0.97 (0.19)
Panobinostat	Drug Eruption	1	0.94 (0.13 - 6.68)	0.94 (0)	-0.09 (-1.76)	0.94 (0.18)
Panobinostat	Streptococcal Sepsis	1	15.1 (2.12 - 107.44)	15.1 (13.15)	3.91 (2.24)	15.08 (2.92)
Panobinostat	Diabetic Ketoacidosis	1	0.54 (0.08 - 3.82)	0.54 (0.4)	-0.89 (-2.56)	0.54 (0.1)
Panobinostat	Rhinovirus Infection	1	3.53 (0.5 - 25.09)	3.53 (1.81)	1.82 (0.15)	3.53 (0.68)
Panobinostat	Small Intestinal Obstruction	1	1.28 (0.18 - 9.12)	1.28 (0.06)	0.36 (-1.31)	1.28 (0.25)
Panobinostat	Gait Inability	1	0.26 (0.04 - 1.88)	0.27 (2.04)	-1.92 (-3.58)	0.27 (0.05)
Panobinostat	Dysphonia	1	0.24 (0.03 - 1.73)	0.24 (2.35)	-2.04 (-3.7)	0.24 (0.05)
Panobinostat	Stress	1	0.2 (0.03 - 1.42)	0.2 (3.19)	-2.32 (-3.98)	0.2 (0.04)
Panobinostat	Spinal Cord Compression	1	3.76 (0.53 - 26.73)	3.76 (2.03)	1.91 (0.24)	3.76 (0.73)
Panobinostat	Pneumonia Streptococcal	1	15.82 (2.22 - 112.56)	15.82 (13.86)	3.98 (2.31)	15.79 (3.06)
Panobinostat	Haemolytic Uraemic Syndrome	1	7.65 (1.08 - 54.34)	7.64 (5.77)	2.93 (1.26)	7.64 (1.48)
Panobinostat	Thrombotic Thrombocytopenic Purpura	1	5.57 (0.78 - 39.57)	5.57 (3.75)	2.48 (0.81)	5.57 (1.08)
Panobinostat	Blood Uric Acid Increased	1	2.78 (0.39 - 19.74)	2.78 (1.14)	1.47 (-0.19)	2.78 (0.54)
Panobinostat	Cell Death	1	7.09 (1 - 50.37)	7.09 (5.22)	2.82 (1.16)	7.08 (1.37)
Panobinostat	Adenocarcinoma	1	6.53 (0.92 - 46.38)	6.53 (4.68)	2.71 (1.04)	6.52 (1.26)
Panobinostat	Acute Respiratory Failure	1	0.77 (0.11 - 5.44)	0.77 (0.07)	-0.38 (-2.05)	0.77 (0.15)
Panobinostat	Blood Sodium Decreased	1	0.91 (0.13 - 6.5)	0.91 (0.01)	-0.13 (-1.8)	0.91 (0.18)
Panobinostat	Burkitt'S Lymphoma	1	29.85 (4.19 - 212.65)	29.84 (27.78)	4.89 (3.22)	29.74 (5.75)
Panobinostat	Drug Resistance	1	0.48 (0.07 - 3.42)	0.48 (0.56)	-1.05 (-2.72)	0.48 (0.09)

Panobinostat	Complications Of Bone Marrow Transplant	1	53.76 (7.52 - 384.07)	53.75 (51.45)	5.74 (4.06)	53.42 (10.31)
Panobinostat	Bacillus Infection	1	32.42 (4.55 - 231.03)	32.41 (30.33)	5.01 (3.34)	32.29 (6.24)
Panobinostat	Pulmonary Haemorrhage	1	2.13 (0.3 - 15.15)	2.13 (0.6)	1.09 (-0.57)	2.13 (0.41)
Panobinostat	Retinopathy	1	4.28 (0.6 - 30.44)	4.28 (2.52)	2.1 (0.43)	4.28 (0.83)
Panobinostat	Peripheral Sensorimotor Neuropathy	1	14.85 (2.09 - 105.6)	14.84 (12.89)	3.89 (2.22)	14.82 (2.87)
Panobinostat	Moaning	1	14.69 (2.07 - 104.52)	14.69 (12.74)	3.87 (2.2)	14.67 (2.84)
Panobinostat	Skin Discolouration	1	0.31 (0.04 - 2.19)	0.31 (1.54)	-1.69 (-3.36)	0.31 (0.06)
Panobinostat	Aspiration	1	1.61 (0.23 - 11.43)	1.61 (0.23)	0.69 (-0.98)	1.61 (0.31)
Panobinostat	Cytomegalovirus Viraemia	1	3.3 (0.47 - 23.47)	3.3 (1.61)	1.72 (0.06)	3.3 (0.64)
Panobinostat	No Adverse Event	1	0.08 (0.01 - 0.59)	0.08 (10.07)	-3.58 (-5.25)	0.08 (0.02)
Panobinostat	Cardiac Tamponade	1	3.22 (0.45 - 22.88)	3.22 (1.53)	1.69 (0.02)	3.22 (0.62)
Panobinostat	Intra-Abdominal Haematoma	1	12.28 (1.73 - 87.3)	12.27 (10.34)	3.62 (1.95)	12.26 (2.37)
Panobinostat	Haematotoxicity	1	1.62 (0.23 - 11.5)	1.62 (0.24)	0.7 (-0.97)	1.62 (0.31)
Panobinostat	Rhabdomyolysis	1	0.45 (0.06 - 3.18)	0.45 (0.68)	-1.16 (-2.82)	0.45 (0.09)
Panobinostat	Dementia	1	0.52 (0.07 - 3.71)	0.52 (0.44)	-0.94 (-2.6)	0.52 (0.1)
Panobinostat	Appendicitis Perforated	1	6.7 (0.94 - 47.6)	6.7 (4.84)	2.74 (1.07)	6.69 (1.3)
Panobinostat	Hallucination, Visual	1	0.79 (0.11 - 5.6)	0.79 (0.06)	-0.34 (-2.01)	0.79 (0.15)
Panobinostat	Transaminases Increased	1	0.66 (0.09 - 4.66)	0.66 (0.18)	-0.61 (-2.27)	0.66 (0.13)
Panobinostat	Balance Disorder	1	0.17 (0.02 - 1.21)	0.17 (4.04)	-2.55 (-4.22)	0.17 (0.03)
Panobinostat	Vertigo	1	0.23 (0.03 - 1.64)	0.23 (2.55)	-2.11 (-3.78)	0.23 (0.04)
Panobinostat	Clostridium Test Positive	1	7.22 (1.02 - 51.3)	7.22 (5.35)	2.85 (1.18)	7.21 (1.4)
Panobinostat	Blood Albumin Decreased	1	2.53 (0.36 - 17.96)	2.53 (0.92)	1.34 (-0.33)	2.53 (0.49)
Panobinostat	Surgery	1	0.26 (0.04 - 1.87)	0.26 (2.06)	-1.93 (-3.59)	0.26 (0.05)
Panobinostat	Hypovolaemia	1	3.4 (0.48 - 24.14)	3.4 (1.69)	1.76 (0.1)	3.4 (0.66)
Panobinostat	Malnutrition	1	1.57 (0.22 - 11.13)	1.57 (0.21)	0.65 (-1.02)	1.57 (0.3)

Panobinostat	Sinus Bradycardia	1	1.76 (0.25 - 12.51)	1.76 (0.33)	0.82 (-0.85)	1.76 (0.34)
Panobinostat	Haematochezia	1	0.25 (0.04 - 1.78)	0.25 (2.24)	-2 (-3.66)	0.25 (0.05)
Panobinostat	Muscle Haemorrhage	1	4.04 (0.57 - 28.73)	4.04 (2.29)	2.02 (0.35)	4.04 (0.78)
Panobinostat	Pulmonary Alveolar Haemorrhage	1	2.84 (0.4 - 20.19)	2.84 (1.19)	1.51 (-0.16)	2.84 (0.55)
Panobinostat	Cataract	1	0.25 (0.03 - 1.74)	0.25 (2.32)	-2.03 (-3.69)	0.25 (0.05)
Panobinostat	Vision Blurred	1	0.11 (0.02 - 0.81)	0.11 (6.85)	-3.12 (-4.79)	0.11 (0.02)
Panobinostat	Hospice Care	1	1.21 (0.17 - 8.62)	1.21 (0.04)	0.28 (-1.39)	1.21 (0.24)
Panobinostat	Rales	1	2.64 (0.37 - 18.76)	2.64 (1.02)	1.4 (-0.27)	2.64 (0.51)
Panobinostat	Pulmonary Thrombosis	1	1.26 (0.18 - 8.93)	1.26 (0.05)	0.33 (-1.34)	1.26 (0.24)
Panobinostat	Sudden Death	1	1.92 (0.27 - 13.6)	1.92 (0.44)	0.94 (-0.73)	1.92 (0.37)
Panobinostat	Troponin Increased	1	2.21 (0.31 - 15.73)	2.21 (0.67)	1.15 (-0.52)	2.21 (0.43)
Panobinostat	Hepatic Enzyme Increased	1	0.22 (0.03 - 1.58)	0.22 (2.71)	-2.16 (-3.83)	0.22 (0.04)
Panobinostat	Diabetes Insipidus	1	6.32 (0.89 - 44.89)	6.32 (4.47)	2.66 (0.99)	6.31 (1.22)
Panobinostat	Otitis Media Acute	1	18.45 (2.59 - 131.32)	18.45 (16.47)	4.2 (2.53)	18.41 (3.56)
Panobinostat	Biliary Colic	1	5.58 (0.78 - 39.62)	5.58 (3.75)	2.48 (0.81)	5.57 (1.08)
Panobinostat	Diarrhoea Haemorrhagic	1	1.57 (0.22 - 11.15)	1.57 (0.21)	0.65 (-1.02)	1.57 (0.3)
Panobinostat	Blood Cholesterol Increased	1	0.38 (0.05 - 2.73)	0.38 (0.99)	-1.38 (-3.05)	0.38 (0.07)
Panobinostat	Pneumocystis Jirovecii Infection	1	12.09 (1.7 - 85.95)	12.09 (10.15)	3.59 (1.92)	12.07 (2.34)
Panobinostat	Varicella Zoster Virus Infection	1	5.43 (0.76 - 38.56)	5.43 (3.61)	2.44 (0.77)	5.42 (1.05)
Panobinostat	Dizziness Postural	1	1.51 (0.21 - 10.74)	1.51 (0.17)	0.6 (-1.07)	1.51 (0.29)
Panobinostat	Mucosal Haemorrhage	1	12.98 (1.82 - 92.28)	12.97 (11.03)	3.7 (2.03)	12.96 (2.51)

HDACi, histone deacetylase inhibitors; PT, preferred term; ROR, reporting odds ratio; CI, confidence interval; PRR, proportional reporting ratio; χ^2, chi-squared; IC, information component; IC025, the lower limit of the 95% CI of the IC; EBGM, empirical Bayesian geometric mean; EBGM05, the lower limit of the 95% CI of EBGM.
